# Metal-free oxidative cross-coupling enabled practical synthesis of atropisomeric QUINOL and its derivatives

**DOI:** 10.1038/s41467-021-22621-2

**Published:** 2021-04-22

**Authors:** Peng-Ying Jiang, Kai-Fang Fan, Shaoyu Li, Shao-Hua Xiang, Bin Tan

**Affiliations:** 1 Department of Chemistry and Shenzhen Grubbs Institute, Guangdong Provincial Key Laboratory of Catalysis, Southern University of Science and Technology, Shenzhen, China; 2 Academy for Advanced Interdisciplinary Studies, Southern University of Science and Technology, Shenzhen, China

**Keywords:** Organocatalysis, Synthetic chemistry methodology

## Abstract

As an important platform molecule, atropisomeric QUINOL plays a crucial role in the development of chiral ligands and catalysts in asymmetric catalysis. However, efficient approaches towards QUINOL remain scarce, and the resulting high production costs greatly impede the related academic research as well as downstream industrial applications. Here we report a direct oxidative cross-coupling reaction between isoquinolines and 2-naphthols, providing a straightforward and scalable route to acquire the privileged QUINOL scaffolds in a metal-free manner. Moreover, a NHC-catalyzed kinetic resolution of QUINOL N-oxides with high selectivity factor is established to access two types of promising axially chiral Lewis base catalysts in optically pure forms. The utility of this methodology is further illustrated by facile transformations of the products into QUINAP, an iconic ligand in asymmetric catalysis.

## Abstract (web summary)

1-(Isoquinolin-1-yl)naphthalen-2-ol (QUINOL) is an atropisomeric heterobiaryl that serves as a platform for the synthesis of other biaryl ligands useful in asymmetric catalysis. Here, the authors report a straightforward oxidative cross-coupling reaction between isoquinolines and 2-naphthols to efficiently access the QUINOL scaffolds in a metal-free manner.

## Introduction

**Fig. 1:**
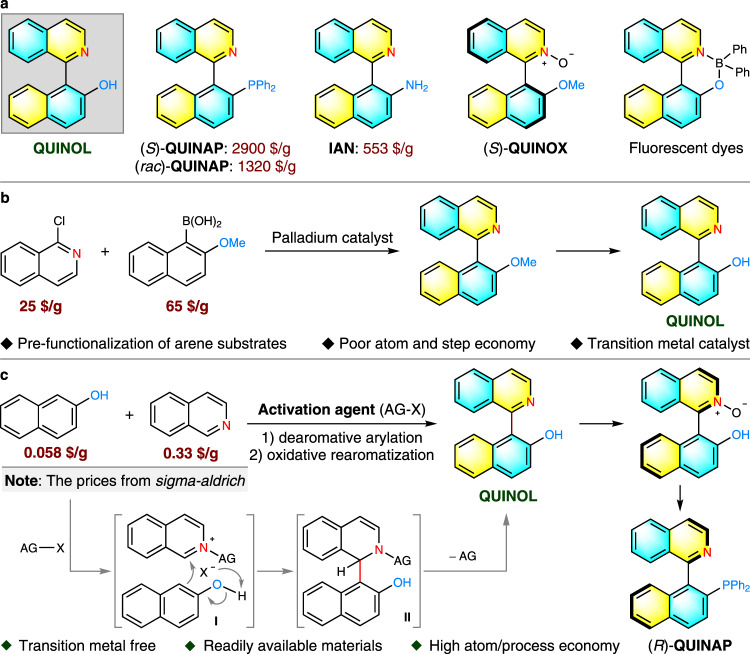
The significance of QUINOL and its synthetic approaches. **a** QUINOL and representative biaryl isoquinoline structures. **b** Current strategy: transition-metal-catalyzed cross-coupling. **c** Our strategy: metal-free oxidative cross-coupling.

The capability to procure axially chiral biaryl skeletons with facility and efficiency has a major impact on advancing catalytic synthesis and material sciences owing to the widespread occurrence of these scaffolds in organocatalysts, ligands, and functional materials^1–7^. In particular, a streamlined access to a core framework from which modular installation of diversity is feasible for divergent construction of functional molecules, will greatly accelerate the development of these fields. 1-(Isoquinolin-1-yl)naphthalen-2-ol (QUINOL), a representative atropisomeric heterobiaryl, was described to function as reliable N–O chelating ligand in the controlled polymerization of cyclic esters^8^ while itself has been synthesized as a platform molecule to access other iconic ligands such as QUINAP^9–13^, QUINOX^14,15^ and IAN^16,17^. A novel organic dye derived from QUINOL has also shown potential as vesicle stains in confocal fluorescence microscopy imaging (Fig. 1a)^18^. In view of these apparent utilities, concise and practical preparation of QUINOL and its derivatives becomes imperative, which remains an unaddressed synthetic challenge. The venerable Suzuki–Miyaura coupling reaction in the transition metal catalysis domain provides a reliable entry^19,20^ but the necessity to pre-functionalize the coupling partners has elongated step counts, hampering overall efficiency which culminates in the high production costs (Fig. 1b). This is reflected in the prices of some commercially available ligands derived from QUINOL. As displayed in Fig. 1a, enantiopure (*S*)-QUINAP costs about 2900 USD/g and even the racemic analog is sold at 1320 USD/g. The cost factor coupled with a lack of modularity has immensely restricted the related academic research as well as downstream industrial applications. These factors collectively illuminate the practical appeal to devise a unified strategy that constructs QUINOLs concisely, particularly in a cost-effective and metal-free manner.

One of the most convergent retrosynthetic disconnections of QUINOL frameworks would trace back to cross-dehydrogenative coupling of isoquinolines and 2-naphthols under oxidative conditions. It is remarkable that related method has never been accomplished, albeit the atom- and step-economy, probably due to the great disparity in redox potential between the two participating reactants. As an exceptional workaround for the limitations encountered in traditional oxidative-coupling, nucleophilic C–H functionalization of electron-deficient *N*-heteroarenes founded on *N*-activation mode has been realized, which encompasses a dearomatizing nucleophilic addition and a sequential oxidative rearomatization. Transition metals and organic reagents with strong Lewis acidity, inherent electron-deficient property as well as Lewis basicity of azaarenes are the key control elements that have been exploited for the success of this reaction type^21–30^. For instance, ZnMe_2_ was utilized by Cho and Baik in a highly regioselective alkylation of *N*-heteroarenes with 1,1-diborylalkanes^23^, while Kanai and Kuninobu developed a site-selective perfluoroalkylation of pyridines and quinolines with perfluoroalkyltrimethylsilane using a sterically bulky borane as the activator^24^. In addition, the *N*-acyl activation mode in Reissert reaction has been demonstrated to be one of the most efficient protocols for direct C–H functionalization of six-membered heteroarenes by Fier^25,26^ and McNally’s group^27–30^. Building on these literature precedents, we envisaged the suitability of this activation mode to direct selective arylation of isoquinoline with 2-naphthol which will occur through a sequence of nucleophilic addition and oxidative re-aromatization to construct QUINOLs (Fig. 1c). The challenging aspect of this reaction design belongs to that of chemoselectivity; differentiation of multiple nucleophilic centers in 2-naphthol and several electrophilic sites in activated acyl isoquinolinium intermediate must be efficient to establish the correct point of attachment. Consequently, the pursuit of an appropriate activation system becomes essential to realize this transformation.

Here, we show our successful endeavor to overcome the above-mentioned challenges, opening up a practical synthetic pathway to structurally diversified QUINOLs by means of a metal-free oxidative cross-coupling reaction between isoquinoline and 2-naphthol. On further development of a chiral NHC-catalyzed kinetic resolution of QUINOL N-oxides, two types of axially chiral QUINOL N-oxides, which are promising Lewis base catalysts can be readily accessible in optically pure forms. Importantly, scalable synthesis and useful transformation have demonstrated the utility of this methodology.

## Results

### Reaction condition optimization

**Fig. 2:**
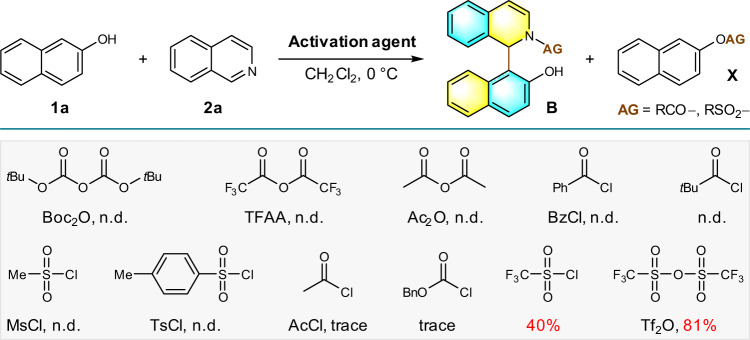
Initial screening of activation agents. Conditions: acylation agent (0.5 mmol) and **2a** (1.25 mmol) were stirred in CH_2_Cl_2_ (4 mL) for 0.5 h at 0 °C. The solution of **1a** (0.50 mmol) in CH_2_Cl_2_ (4 mL) was then added and stirred for another 2 h. Isolated yield was provided.

Initiating our investigations, the influence of the activation reagents on dearomative arylation step was examined in a model coupling reaction of 2-naphthol **1a** and isoquinoline **2a** (Fig. 2). In the excess of isoquinoline, the first attempt with Boc_2_O in CH_2_Cl_2_ at 0 °C only afforded the byproduct from the esterification of 2-naphthol. Evaluation of a panel of acyl chloride and anhydride including TFAA, Ac_2_O, BzCl, MsCl, TsCl, and pivaloyl chloride has resulted in similar reaction outcomes. The first hint of success came when a trace amount of target compound **B** was detected by LCMS in reactions that employed AcCl or benzyl chloroformate. Following investigations revealed that trifluoromethanesulfonyl chloride could impart major improvement in reaction efficiency to provide **B** in 40% isolated yield. Trifluoromethanesulfonic anhydride (Tf_2_O) proved to be the optimal activator for selective formation of the C–C bond under this set of conditions and delivered the desired product in 81% yield, along with 10% yield of esterification by-product.

**Fig. 3:**
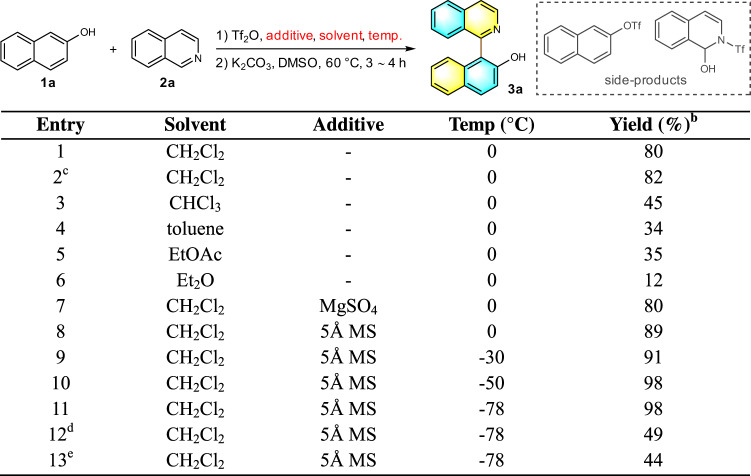
Optimization of the conditions^a^. ^a^Reaction conditions: To a Schlenk tube was added additive (0 or 250 mg), **2a** (1.25 mmol), CH_2_Cl_2_ (4 mL) and Tf_2_O (0.5 mmol) successively. After stirring at specified temperature for 0.5 h, a solution of **1a** (0.5 mmol) in CH_2_Cl_2_ (4 mL) was added. The reaction mixture slowly increased to room temperature, and then continued to stir for 2 h. ^b^Isolated yield. ^c^**1a** (0.55 mmol) was used. ^d^**2a** (0.5 mmol) and Et_3_N (1.5 eq.) were used. ^e^**2a** (0.5 mmol) and NaHCO_3_ (1.5 eq.) were used.

After a simple workup, intermediate **B** could be readily converted to the target QUINOL **3a** in nearly quantitative yield by treatment with K_2_CO_3_ in DMSO at 60 °C for 3 h (Fig. 3, entry 1). The identity of **3a** was confirmed by single-crystal X-ray diffraction analysis (CCDC 2005905). This achievement encouraged us to investigate other reaction parameters of the first arylation step to further enhance the overall effectiveness of this cross-coupling reaction. An increased loading of 2-naphthol (Fig. 3, entry 2) failed to suppress the side reactions, whereas switching the reaction medium to chloroform, toluene, ethyl acetate or diethyl ether did not improve the outcomes as well (Fig. 3, entries 3–6). Given H_2_O adduct was identified in LCMS during the initial optimization process, we reckoned that the moisture in the reaction system could be detrimental to the productive reaction pathway. As such, a series of additives known to sequester moisture were included. The addition of Mg_2_SO_4_ did not bring about any positive effect in terms of product yield (Fig. 3, entry 7) while 5 Å molecular sieve successfully obstructed the *O-*acylation thus raised the product yield to 89% (Fig. 3, entry 8). Interestingly, competitive esterification of 2-naphthol was inhibited altogether when at lower reaction temperatures (Fig. 3, entries 9–11) with quantitative yield being secured at −50 or −78 °C. The introduction of an exogenous base such as Et_3_N or NaHCO_3_ has eroded the chemoselectivity instead (Fig. 3, entries 12 and 13).

### Substrate scope

**Fig. 4:**
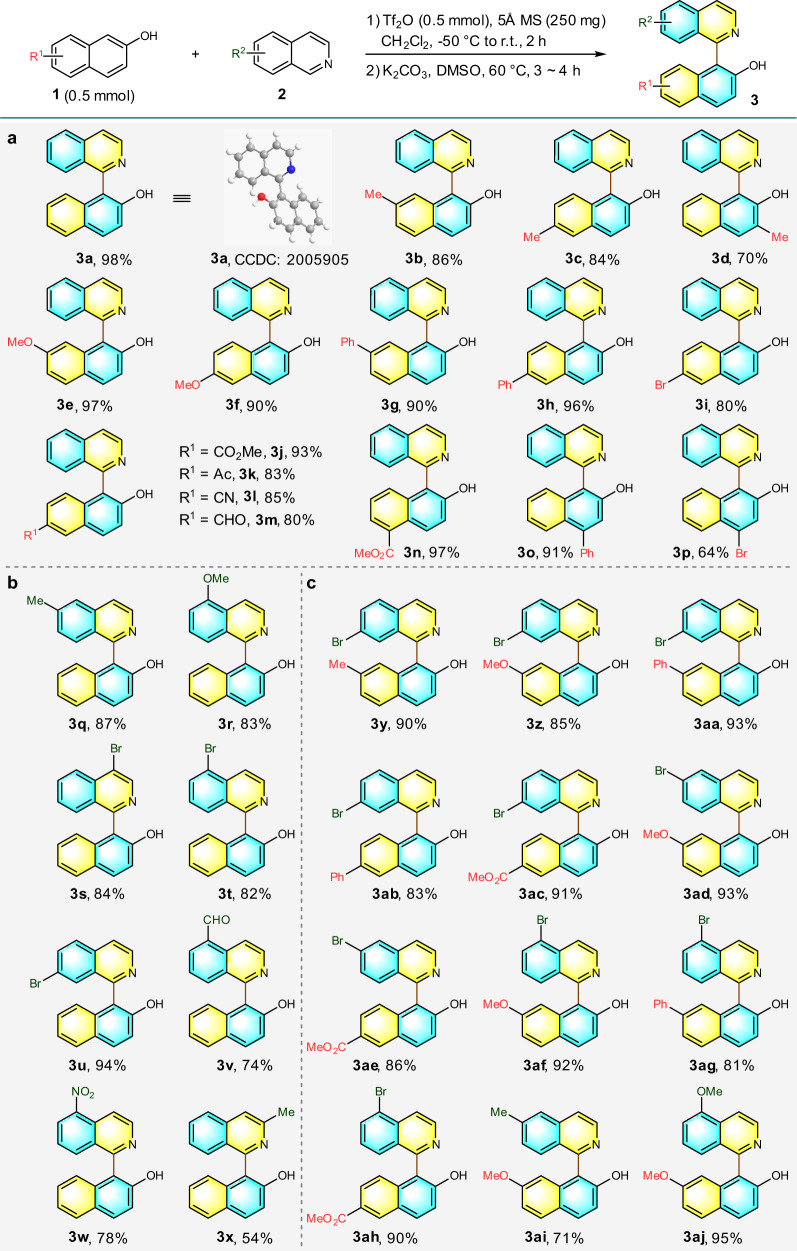
Substrate scope of the oxidative cross-coupling of 2-naphthols and isoquinolines. **a** Scope of 2-naphthalenol. **b** Scope of isoquinoline. **c** Modification of both coupling partners.

Having identified the optimal reaction parameters for the model coupling, we turned to probe the generality of this set of conditions. As shown in Fig. 4, a wide range of substrates with different substituents attached in varied patterns were well tolerated to form target products in high to excellent yields without chromatographic purification of the intermediates. Firstly, electronic variations on 2-naphthol substrates posed limited influence on the reactivity. Substrates with electron-donating and electron-withdrawing groups furnished the corresponding QUINOLs (**3b**–**3p**) in more than 80% yields within similar reaction duration, except **3d** and **3p**. The slight decrease in the yields of **3k**, **3l** and **3m** may be ascribed to the poor solubility of reacting substrates. Notably, the tolerance of ester, cyano, aldehyde, and acetyl groups signified the potential chemical manipulations to introduce other useful functionalities onto product scaffold (Fig. 4b). Next, the conditions were studied with respect to variations on isoquinoline where all selected substrates generally furnished the expected products **3q–3x** in high efficiency (Fig. 4b). It could be discerned that the coupling efficacy of electron-poor isoquinolines (**3v** and **3w**) was lower compared to the more electron-rich analogs (**3q** and **3r**) or those carrying halide moiety (**3s–3u**). Moreover, the 3-position substituent of isoquinoline posed a negative effect on the transformation (**3x**). To enrich the structural diversity of QUINOL framework, a wide spectrum of di-substituted QUINOL derivatives were synthesized smoothly using the same set of conditions (**3y**–**3aj**). It is worth pointing out that the ability to introduce substituents at both 7,7′-positions of QUINOL backbone provides the flexibility to adjust the dihedral angle of the biaryl motifs, which is a strong asset in the design and fine-tuning of ligands or organocatalysts. More importantly, bromo substituent which was commonly incompatible with conventional palladium-catalyzed Suzuki-Miyaura cross-couplings was left unscathed on both coupling partners under our metal-free reaction conditions (**3i**, **3s–3u**, and **3y–3ah**). The orthogonality of the present method to classic cross-coupling chemistry would permit downstream functionalization and further diversification of QUINOL scaffolds via these robust coupling protocols.

The untapped utilitarian potential of these structurally diverse QUINOL analogs in asymmetric catalysis provides a strong incentive to resolve the racemates using kinetic resolution. Initial efforts to isolate the individual enantiomers by preparative chiral HPLC revealed the low configurational stability of the stereogenic axis in these scaffolds. We sought to continue the endeavor through synthesizing the more stable QUINOL N-oxide derivatives, which could be easily achieved from the oxidation of prepared QUINOLs with DMDO. Several relevant works have served as important precedents for our approach. The preparation of axially chiral QUINOL scaffolds via kinetic resolution is pioneered by You and Gu in 2014, where Pd(II)-catalyzed direct C–H iodination forged axially chiral isoquinoline N-oxides with moderate enantioselectivity^31^. A biocatalytic dynamic kinetic resolution enabled by configurational lability of precursors was then prominently disclosed by Clayden and Turner^32^. Diversely, Matsubara and Asano attained a highly enantioselective aromatic electrophilic bromination of 1-(3-hydroxyphenyl)isoquinoline 2-oxides to construct axially chiral isoquinoline N-oxides by using a bifunctional catalyst^33^. Recognizing the structural characteristic of our QUINOL N-oxide products, the contributions from Zhao^34^ and Wang^35^ on enantioselective kinetic resolution of NOBIN analogs via chiral *N*-heterocyclic carbene (NHC) catalyzed acylation reactions inspired our formulation of an analogous strategy to obtain the axially chiral QUINOL derivatives in enantioenriched forms.

### Kinetic resolution of QUINOL N-oxides

**Fig. 5:**
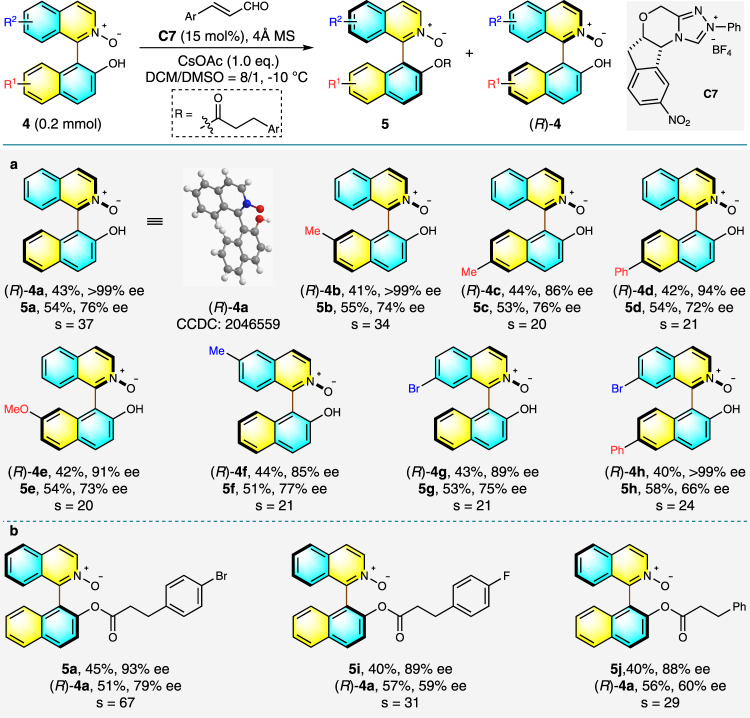
Substrate scope of NHC-catalyzed kinetic resolution of QUINOL N-oxides. **a** Ar = 4-Br-Ph (0.65 eq. aldehyde). **b** with 0.5 eq. aldehydes.

An interrogation of different NHC precatalysts for this kinetic resolution process has demonstrated the sensitivity of enantioselectivity outcome towards the identity of aryl group on the triazolium moiety where sterically hindered ones were found unfavorable. **C7** which possesses a nitro group on the other aryl group, was subsequently identified as the lead precatalyst. The poor solubility of QUINOL N-oxide in most common solvents put an additional constraint on stereochemical control. Eventually, this was effectively addressed through employing a binary mixture of CH_2_Cl_2_ and DMSO (for more detailed reaction optimization of the kinetic resolution, see Supplementary Table 3). We also investigated the generality of this NHC-catalyzed kinetic resolution strategy (Fig. 5) and found it to be pleasingly broad. Most racemic QUINOL N-oxides could be stereochemically enriched to 85->99% ee and isolated in 40–44% yield. Particularly noteworthy is that the acylation products **5** and the remaining (*R*)-**4** could be obtained with high yields and good ee values simply by adjusting the loading of aldehydes. Under the standard conditions (0.65 equiv of aldehyde), (*R*)-**4a** was produced in 43% yield with >99% ee while the acylation product **5a** was afforded in 45% yield and 93% ee using 0.5 equiv. of aldehyde (Fig. 5). The absolute configuration of (*R*)-**4a** was determined by X-ray crystallographic analysis (CCDC 2046559), and those of other QUINOL N-oxides displayed in Fig. 5 were assigned by analogy.

### Preparative synthesis and transformations

**Fig. 6:**
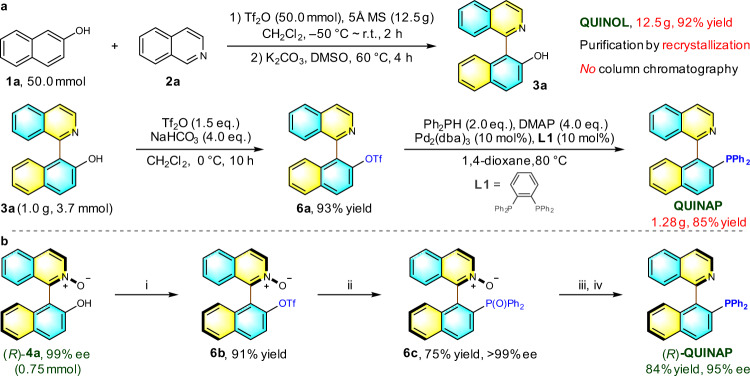
Preparative synthesis and transformations. **a** Gram-scale synthesis of (*rac*)-QUINOL and (*rac*)-QUINAP. **b** Synthesis of (*R*)-QUINAP from (*R*)-QUINOL N-oxide. Reaction conditions: i Tf_2_O (1.05 eq.), DMAP (2.0 eq.), CH_2_Cl_2_, r.t., 10 h; ii diphenylphosphine oxide (2.0 eq.), Pd(OAc)_2_ (12 mol%), **L1** (24 mol%), DIPEA (5.5 eq.), DMSO, 110 °C, 24 h; iii *bis*(pinacolato)diboron (1.0 eq.), diglyme, 70 °C, 12 h; iv HSiCl_3_ (10.0 eq.), DIPEA (40.0 eq.), toluene, 70 °C, 4 h.

The practicality of the methodology in terms of scalability was first testified with a decagram-scale synthesis of QUINOL product performed under the optimal reaction conditions (Fig. 6a). QUINOL **3a** was effortlessly generated in 92% yield and 12.5 gram when the standard reaction was performed on a 50 mmol scale. The procedural simplicity (i.e., post-reaction treatment and rapid recrystallization of product) holds promises for industrial-scale production. The streamlined synthesis of QUINAP from QUINOL **3a** was also scalable and an overall yield of 79% was obtained over two steps. Subsequently, optically active (*R*)-QUINAP was efficiently synthesized in four steps using enantiopure (*R*)-QUINOL N-oxide as the starting material with routine transformations (Fig. 6b).

### Controlled experiments and proposed reaction processes

**Fig. 7:**
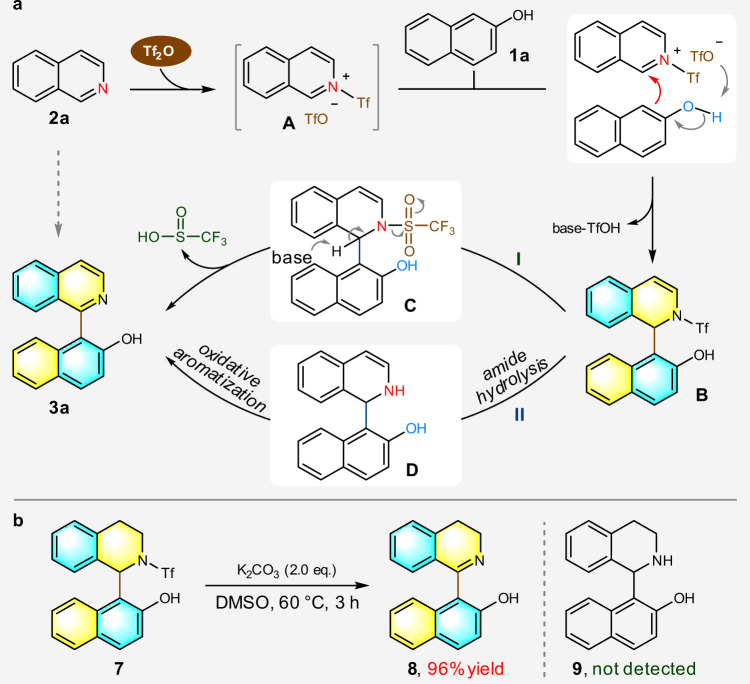
Proposed reaction mechanism and control experiment. **a** Proposed reaction pathway. **b** Control experiment.

Guided by our continuous understanding of the construction of axially chiral compounds with 2-naphthol as the nucleophile^36^, a plausible mechanistic pathway was rationalized as outlined in Fig. 7. This coupling reaction is initiated when Tf_2_O activates the isoquinoline **2a** in the form acyl isoquinolinium **A**. The nucleophilic 2-naphthol **1a** then adds to the iminium carbon of activated species **A** selectively from C1 position, as simultaneously promoted by the proton abstraction from hydroxyl group by the proximal triflate anion; tautomerization at naphthol ring then gives rise to dearomatized intermediate **B**. Such intermolecular synergistic activation-addition events may play a crucial role to overcome the otherwise challenging chemoselectivity issue resulting from competitive nucleophilic and electrophilic sites present on these reacting partners. Finally, an oxidative aromatization process delivers the target product QUINOL **3a** along with the removal of Tf group. Noteworthily, there are two possible pathways for the last step, namely, base-promoted redox process (Fig. 7a, pathway **I**) or amide hydrolysis-aromatization-driven oxidation cascade process (Fig. 7a, pathway **II**). To obtain more insights into this step, a control experiment was designed. In light of the powerful driving force for aromatization in intermediate **D**, it should be infeasible to capture such highly active species. Therefore, the hydrogenation product **7** of intermediate **B** was prepared as the starting material for mechanistic investigation. As displayed in Fig. 7b, the treatment of **7** under the standard conditions provided product **8** in 96% yield with no formation of product **9**. This lent indirect evidence to rule out the possibility of pathway **II** and indicated that Tf_2_O is not only an activator of isoquinoline but also an oxidant for the oxidative aromatization process.

## Discussion

In conclusion, a metal-free oxidative cross-coupling reaction between isoquinoline and 2-naphthol has been discovered to deliver structurally diversified QUINOLs. This protocol features desirable blend of high yield, exclusive chemoselectivity, robust functional group tolerance, and step economy for the construction of privileged QUINOL derivatives. Moreover, the low cost of raw materials, ease of purification, and decagram-scale preparation portend the adaptability to process settings. For illustration, a streamlined and large-scale synthesis of QUINAP from QUINOL product was demonstrated which presents superiority in terms of production cost. Following a chiral NHC-catalyzed kinetic resolution, two types of axially chiral QUINOL N-oxides which are promising Lewis base catalysts were readily accessible in enantioenriched forms. Derivatization into (*R*)-QUINAP was also accomplished with standard chemical transformations, thus providing a practical method to access these important frameworks.

## Methods

### Procedure for the enantioselective synthesis of 3

To a dried Schlenk tube was added 5 Å molecular sieve (250 mg), isoquinoline **2** (1.25 mmol), and CH_2_Cl_2_ (4 mL) under argon atmosphere. After the solution was cooled to −50 °C, Tf_2_O (0.5 mmol) was added through a microsyringe and stirred for half an hour until white solid salts appeared. After adding the solution of 2-naphthol **1** (0.5 mmol) in CH_2_Cl_2_ (4 mL), the reaction was slowly raised to room temperature and then continued to stir for 2 h. The molecular sieve was removed by filtration, and the resulting filtrate was washed successively with 3 M HCl, saturated sodium bicarbonate, and brine, and then dried over anhydrous Na_2_SO_4_. After evaporating the solvent under reduced pressure, the resulting intermediate **B** was dissolved in 4 mL of DMSO, and K_2_CO_3_ (138 mg, 1.0 mmol) was then added. The mixture was stirred at 60 °C for about 3–4 h until the intermediate was consumed completely. The reaction system was adjusted to weakly acidic with 3 M HCl, and then to weakly basic with saturated sodium bicarbonate. The mixture was diluted with 20 mL of water, and extracted with EA (20 mL **×** 3). The combined organic phase was washed with brine (20 mL **×** 2), dried over anhydrous Na_2_SO_4_, and concentrated to afford the crude product, which was purified by column chromatography eluted with PE/EA (10/1 to 3/1) to afford the pure product.

### Procedure for the kinetic resolution of 4

Under argon atmosphere, substrate **4** (0.2 mmol), cinnamaldehyde (0.13 mmol, 0.65 eq.), **C7** (13 mg, 0.03 mmol), activated 4 Å MS (400 mg), solvent (18 mL, CH_2_Cl_2_/DMSO = 8/1), and CsOAc (0.2 mmol, 1.0 eq.) were added sequentially to a dried Schlenk flask. The mixture was stirred at −10 °C until the aldehyde was completely consumed (monitored by TLC). After removing the molecular sieve by filtration, the obtained filtrate was diluted with 30 mL of water, and then extracted with DCM (30 mL × 3). The combined organic phase was washed with 30 mL brine, dried over anhydrous Na_2_SO_4_, and concentrated to afford crude products, which were purified by column chromatography eluted with DCM/EA (20/1 to 1/1) to afford the pure products **5** and (*R*)-**4**.

## Supplementary information

Supplementary Information

## Data Availability

The X-ray crystallographic coordinates for structures reported in this Article have been deposited at the Cambridge Crystallographic Data Centre (CCDC), under deposition numbers CCDC 2005905, CCDC 2046559. These data can be obtained free of charge from The Cambridge Crystallographic Data Centre via http://www.ccdc.cam.ac.uk/ data_request/cif.
