# Supplementary material for: Metal-free oxidative cross-coupling enabled practical synthesis of atropisomeric QUINOL and its derivatives
Source: Nat Commun. 2021 Apr 22;12:2384. doi: 10.1038/s41467-021-22621-2 (PMC8062545; doi:10.1038/s41467-021-22621-2)
Supplement: Supplementary file 1 — Supplementary Information [file 41467_2021_22621_MOESM1_ESM.pdf]

Supplementary information for

Metal-Free Oxidative Cross-Coupling Enabled Practical Synthesis of Atropisomeric QUINOL and Its Derivatives

Peng-Ying Jiang[1,3], Kai-Fang Fan[1,3], Shaoyu Li[1,2]*, Shao-Hua Xiang[1,2] & Bin Tan[1]*

[1]Department of Chemistry and Shenzhen Grubbs Institute, Guangdong Provincial Key Laboratory of Catalysis, Southern University of Science and Technology, Shenzhen 518055, China. [2]Academy for Advanced Interdisciplinary Studies, Southern University of Science and Technology, Shenzhen 518055, China. [3]These authors contributed equally to this work: Peng-Ying Jiang, Kai-Fang Fan.

*email: *lisy@sustech.edu.cn; tanb@sustech.edu.cn*

Table of Contents

1. General information 2
2. Optimization of the reaction conditions 3
3. Procedure for the synthesis of QUINOL derivatives 5
4. Procedure for the preparation of racemic QUINOL N-oxides 24
5. Procedure for the kinetic resolution of QUINOL N-oxides 29
6. Gram-scale preparation of QUINOL and (*rac*)-QUINAP 52
7. Synthesis of (*R*)-QUINAP from (*R*)-QUINOL N-oxide 55
8. Control experiment for mechanism investigation 61
9. Crystal data and structure of compounds 3a and (*R*)-4a 65
10. Copies of ^{1}H and ^{13}C NMR spectra of related compounds 89
11. Supplementary References 155

1. General information

Chemicals were purchased from commercial suppliers and used without further purification unless otherwise stated. Analytical thin layer chromatography (TLC) was performed on precoated silica gel 60 GF254 plates. Flash column chromatography was performed using Tsingdao silica gel (60, particle size 0.040-0.063 mm). Visualization on TLC was achieved by use of UV light (254 nm). ^{1}H and ^{13}C NMR spectra were recorded on Bruker 400 MHz or 500 MHz spectrometer in $CDCl_3$ or DMSO-d_6 with tetramethylsilane (TMS) as internal standard. The chemical shifts are expressed in ppm and coupling constants are given in Hz. Data for 1H NMR are recorded as follows: chemical shift (δ, ppm), multiplicity (s = singlet; d = doublet; t = triplet; q = quartet; p = pentet; m = multiplet; brs = broad singlet), coupling constant (Hz), integration. Data for ^{13}C NMR, ^{19}F NMR and ^{31}P NMR, are reported in terms of chemical shift (δ, ppm). The enantiomeric excess values were determined by chiral HPLC with an Agilent instrument and a Daicel CHIRALCEL and CHIRALPAK column. High resolution mass spectroscopy (HRMS) analyses were performed at a Q-Exactive (Thermo Scientific) Inc mass instrument (HESI).

2. Optimization of the reaction conditions

2.1 Optimization of re-aromatization conditions to form QUINOL

Supplementary Table 1 Optimization of the Reaction Conditions[a]

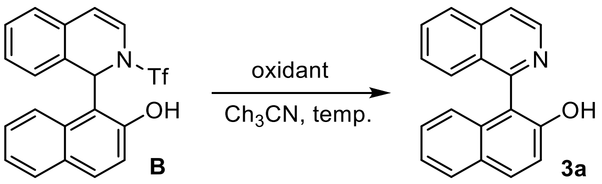

entry	oxidant	T [℃]	yield [%]
1	IBX	rt~60	n.d.
2	DMP	rt~60	n.d.
3	DDQ	rt~60	n.d.
4	PCC	rt~60	n.d.
5[*b*]	Pd-C/O_2	rt~60	n.d.

[a]Reaction conditions: Substrate **B** (0.2 mol) and oxidant (2.0 eq.) was stirred in CH_3CN (4 mL). [b]Performed with Pd-C (10 mol%) under O_2 atmosphere.

Supplementary Table 2 Optimization of the reaction conditions[a]

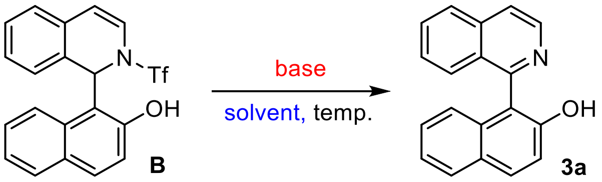

entry	base	solvent	**T** [℃]	**yield** [%][b]
1	Cs_2CO_3	CH_3CN	60	70
2	Cs_2CO_3	DCE	60	n.d.
3	Cs_2CO_3	1,4-dioxane	60	trace
4	Cs_2CO_3	toluene	60	n.d.
5	Cs_2CO_3	DMSO	60	83
6	Na_2CO_3	DMSO	60	75
7	K_2CO_3	DMSO	60	98
8	K_3PO_4	DMSO	60	92
9	CsOAc	DMSO	60	75
10	NaO*t*Bu	DMSO	60	n.d.
11[c]	K_2CO_3	DMSO	60	30
12[d]	K_2CO_3	DMSO	r.t.	90

[a]Reaction conditions: Substrate **B** (0.2 mol) and base (2.0 eq.) was stirred in solvent (4 mL) at 60 ℃ for about 3 h. [b]NMR yield was provided using 1,3,5-trimethoxybenzene as internal standard. [c]K_2CO_3 (0.2 eq.) was used. [d]Reaction time is 24 h.

2.2 Optimization of conditions for NHC-catalyzed kinetic resolution of QUINOL N-oxide

Supplementary Table 3 Screening of parameters for the kinetic resolution of QUINOL N-oxide[a]

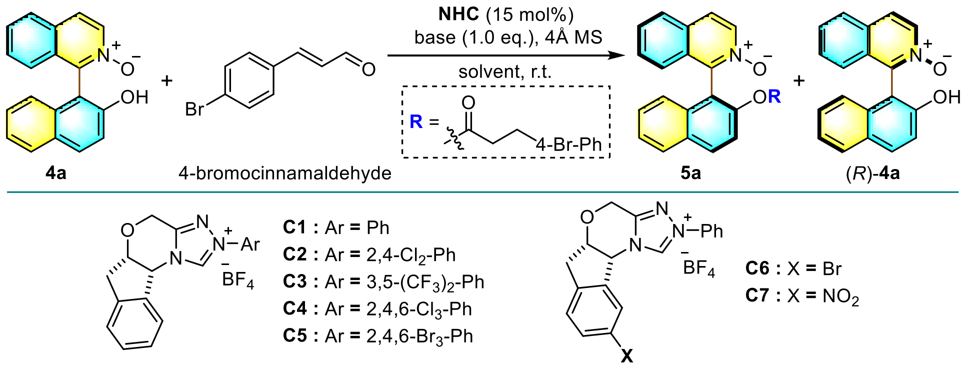

entry	NHC	base	solvent	5a[b] yield, *ee* [%]	(*R*)-4a[b] yield, *ee* [%]	conv [%][c]	s[d]
1	**C1**	CsOAc	CH_2Cl_2	44, 55	54, 46	46	5
2	**C2**	CsOAc	CH_2Cl_2	42, 45	50, 37	45	4
3	**C3**	CsOAc	CH_2Cl_2	30, 43	68, 21	33	3
4	**C4**	CsOAc	CH_2Cl_2	35, -26	62, -16	38	2
5	**C5**	CsOAc	CH_2Cl_2	48, -13	46, -13	50	2
6	**C6**	CsOAc	CH_2Cl_2	48, 59	45, 56	49	7
7	**C7**	CsOAc	CH_2Cl_2	47, 68	45, 65	49	10
8	**C7**	CsOAc	THF	39, 22	58, 16	42	2
9	**C7**	CsOAc	EA	35, 19	62, 11	37	2
10	**C7**	CsOAc	toluene	12, 28	90, 5	15	2
11	**C7**	CsOAc	DMSO	/	/	trace	/
12	**C7**	CsOAc	CH_2Cl_2/DMSO = 8/1	43, 75	52, 64	46	13
13	**C7**	NaOAc	CH_2Cl_2/DMSO = 8/1	25, 70	70, 24	26	7
14	**C7**	LiOAc	CH_2Cl_2/DMSO = 8/1	38, 56	54, 36	39	5
15	**C7**	Na_2CO_3	CH_2Cl_2/DMSO = 8/1	47, 52	46, 50	49	5
16	**C7**	K_2CO_3	CH_2Cl_2/DMSO = 8/1	25, 27	72, 11	29	2
17	**C7**	Et_3N	CH_2Cl_2/DMSO = 8/1	40, 70	55, 53	43	10
18	**C7**	DIPEA	CH_2Cl_2/DMSO = 8/1	9, 85	86, 12	12	14
19[e]	**C7**	CsOAc	CH_2Cl_2/DMSO = 8/1	38, 87	60, 66	43	28
20[f]	**C7**	CsOAc	CH_2Cl_2/DMSO = 8/1	45, 93	51, 79	46	67
21[g]	**C7**	CsOAc	CH_2Cl_2/DMSO = 8/1	37, 91	58, 60	40	39
22[f,h]	**C7**	CsOAc	CH_2Cl_2/DMSO = 8/1	54, 76	43, >99	57	37

[a]Unless otherwise stated, all reactions were performed with **4a** (0.2 mmol), 4-bromocinnamaldehyde (0.1 mmol, 0.5 eq.), 4Å MS (400 mg), base (0.2 mmol, 1.0 eq.), NHC (15 mol%) and solvent (18.0 mL) at 25 ℃ for 24 h. [b]Isolated yield, and ee value determined by chiral HPLC. [c]conv = *ee*(**4a**)/(*ee*(**4a**) + *ee*(**5a**)). [d]**s** = ln[(1-conv)(1-*ee*(**4a**))]/ln[(1-conv)(1 + *ee*(**4a**))]. [e]The reaction was performed at 0 ℃ for 2 days. [f]The reaction was performed at -10 ℃ for 3 days. [g]The reaction was performed at -20 ℃ for 5 days. [h]With 0.65 equiv

4-bromocinnamaldehyde.

3. Procedure for the synthesis of QUINOL derivatives

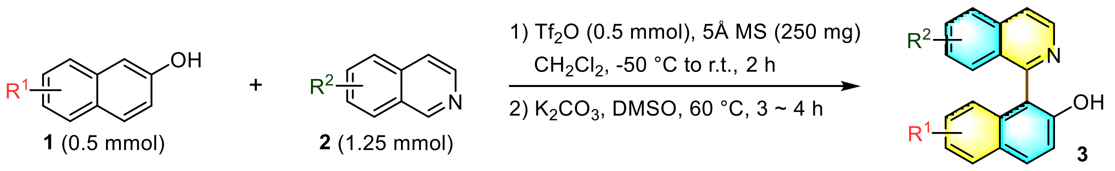

To a dried Schlenk tube was added 5Å molecular sieve (250 mg), isoquinoline **2** (1.25 mmol) and CH_2Cl_2 (4 mL) under argon atmosphere. After the solution was cooled to -50 ℃, Tf_2O (0.5 mmol) was added through a microsyringe and stirred for half an hour until white solid salts appeared. After adding the solution of 2-naphthol **1** (0.5 mmol) in CH_2Cl_2 (4 mL), the reaction was slowly raised to room temperature, and then continued to stir for 2 hours. The molecular sieve was removed by filtration, and the resulting filtrate was washed successively with 3M HCl, saturated sodium bicarbonate and brine, and then dried over anhydrous Na_2SO_4. After evaporating the solvent under reduced pressure, the resulting intermediate **B** was dissolved in 4 mL of DMSO, and K_2CO_3 (138 mg, 1.0 mmol) was then added. The mixture was stirred at 60 ℃ for about 3 ~ 4 hours until the intermediate was consumed completely. The reaction system was adjusted to weakly acidic with 3M HCl, and then to weakly basic with saturated sodium bicarbonate. The mixture was diluted with 20 mL of water, and extracted with EA (20 mL × 3). The combined organic phase was washed with brine (20 mL × 2), dried over anhydrous Na_2SO_4 and concentrated to afford the crude product, which was purified by column chromatography eluted with PE/EA (10/1 to 3/1) to afford the pure product **3**.

Characterizations of products 3:

1-(Isoquinolin-1-yl)naphthalen-2-ol (3a)

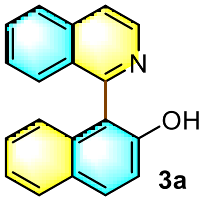

3a

According to the general procedure, **3a** was obtained in **98% yield** as a white solid. **^{1}H NMR (400 MHz, DMSO-d_6)** δ 9.72 (s, 1H), 8.67 (d, J = 5.7 Hz, 1H), 8.05 (d, J = 8.3 Hz, 1H), 7.96 (d, J = 8.9 Hz, 1H), 7.90-7.88 (m, 2H), 7.75-7.71 (m, 1H), 7.48-7.43 (m, 2H), 7.38 (d, J = 8.9 Hz, 1H), 7.28-7.25 (m, 1H), 7.22-7.18 (m, 1H), 6.85 (d, J = 8.4 Hz, 1H). **^{13}C NMR (100 MHz, DMSO-d_6)** δ 157.9, 152.7, 142.5, 135.8, 133.8, 130.2, 129.8, 128.0, 128.0, 127.8, 127.4, 127.0, 126.9, 126.4, 123.9, 122.6, 119.9, 118.4, 118.3. **HRMS (ESI)** calcd for $[M+H]^+$ $C_{19}H_{14}NO^+$, m/z: 272.1070, found: 272.1068.

1-(Isoquinolin-1-yl)-7-methylnaphthalen-2-ol (3b)

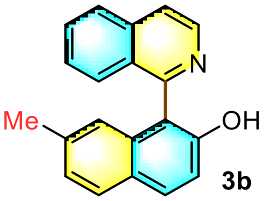

3b

According to the general procedure, **3b was obtained in 86% yield** as a white solid. **^{1}H NMR (400 MHz, DMSO-d_6)** δ 9.70 (s, 1H), 8.67 (d, J = 5.7 Hz, 1H), 8.03 (d, J = 8.2 Hz, 1H), 7.90-7.87 (m, 2H), 7.78 (d, J = 8.3 Hz, 1H), 7.74-7.70 (m, 1H), 7.46-7.45 (m, 2H), 7.31 (d, J = 8.8 Hz, 1H), 7.10 (dd, J = 8.3, 1.6 Hz, 1H), 6.64 (s, 1H), 2.14 (s, 3H). **^{13}C NMR (100 MHz, DMSO-d_6)** δ 158.2, 152.9, 142.6, 135.9, 135.6, 134.1, 130.3, 129.5, 128.1, 128.0, 127.4, 127.0, 127.0, 126.2, 124.9, 122.8, 112.0, 118.0, 117.3, 21.6. **HRMS (ESI)** calcd for $[M+H]^+$ $C_{20}H_{16}NO^+$, m/z: 286.1226, found: 286.1225.

1-(Isoquinolin-1-yl)-6-methylnaphthalen-2-ol (3c)

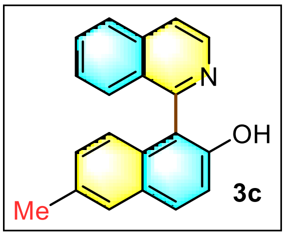

According to the general procedure, **3c** was obtained in **84% yield** as a white solid. **^{1}H NMR (400 MHz, $CDCl_3$)** δ 8.62 (d, *J* = 5.7 Hz, 1H), 7.92 (d, *J* = 8.3 Hz, 1H), 7.76-7.73 (m, 2H), 7.72-7.68 (m, 1H), 7.61-7.58 (m, 2H), 7.41-7.37 (m, 1H), 7.22 (d, *J* = 8.9 Hz, 1H), 7.07-7.00 (m, 2H), 2.45 (s, 3H). **^{13}C NMR (100 MHz, $CDCl_3$)** δ 157.5, 152.7, 141.8, 137.1, 132.7, 131.1, 130.7, 130.4, 129.0, 128.6, 128.3, 128.2, 127.3, 127.2, 127.1, 124.9, 120.8, 119.0, 117.1, 21.3. **HRMS (ESI)** calcd for $[M+H]^+$ $C_{20}H_{16}NO^+$, m/z: 286.1226, found: 286.1225.

1-(Isoquinolin-1-yl)-3-methylnaphthalen-2-ol (3d)

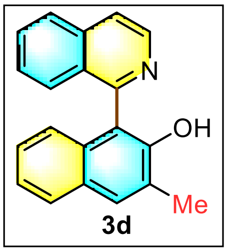

According to the general procedure, **3d was obtained in 70% yield** as a yellow solid. **^{1}H NMR (400 MHz, DMSO-*d*$_6$)** δ 8.68 (d, *J* = 5.7 Hz, 2H), 8.07 (dd, *J* = 8.4, 1.1 Hz, 1H), 7.92 (dd, *J* = 5.7, 0.8 Hz, 1H), 7.83-7.80 (m, 2H), 7.76-7.72 (m, 1H), 7.48-7.44 (m, 1H), 7.39 (dd, *J* = 8.4, 1.2 Hz, 1H), 7.27-7.23 (m, 1H), 7.15-7.10 (m, 1H), 6.72 (dd, *J* = 8.5, 1.1 Hz, 1H), 2.45 (s, 3H). **^{13}C NMR (100 MHz, DMSO-*d*$_6$)** δ 157.7, 151.6, 142.6, 136.0, 132.5, 130.2, 129.2, 128.3, 128.1, 127.7, 127.4, 127.2, 127.1, 126.8, 125.4, 123.8, 122.9, 120.2, 119.3, 17.4. **HRMS (ESI)** calcd for $[M+H]^+$ $C_{20}H_{16}NO^+$, m/z: 286.1226, found: 286.1224.

1-(Isoquinolin-1-yl)-7-methoxynaphthalen-2-ol (3e)

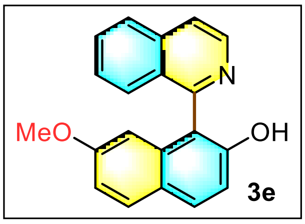

According to the general procedure, **3e** was obtained in **97% yield** as a white solid. **^{1}H NMR (400 MHz, DMSO-d_6)** δ 9.61 (s, 1H), 8.66 (d, J = 5.7 Hz, 1H), 8.05 (d, J = 8.3 Hz, 1H), 7.90 (d, J = 5.8 Hz, 1H), 7.86 (d, J = 8.8 Hz, 1H), 7.82 (d, J = 8.9 Hz, 1H), 7.77-7.73 (m, 1H), 7.52-7.44 (m, 2H), 7.18 (d, J = 8.8 Hz, 1H), 6.96 (dd, J = 9.0, 2.4 Hz, 1H), 6.17 (d, J = 2.3 Hz, 1H), 3.43 (s, 3H). **^{13}C NMR (100 MHz, DMSO-d_6)** δ 158.0, 157.6, 153.4, 142.4, 135.9, 135.1, 130.3, 129.7, 129.6, 127.9, 127.4, 127.0, 127.0, 123.3, 120.0, 117.6, 115.7, 114.3, 103.4, 54.6. **HRMS (ESI)** calcd for $[M+H]^+$ $C_{20}H_{16}NO_2^+$, m/z: 302.1176, found: 302.1173.

1-(Isoquinolin-1-yl)-6-methoxynaphthalen-2-ol (3f)

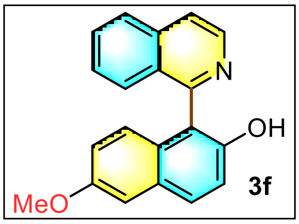

According to the general procedure, **3f** was obtained in **90% yield** as a white solid. **^{1}H NMR (400 MHz, DMSO-d_6)** δ 9.46 (s, 1H), 8.66 (d, J = 5.7 Hz, 1H), 8.04 (d, J = 8.2 Hz, 1H), 7.87 (dd, J = 10.2, 7.3 Hz, 2H), 7.75-7.71 (m, 1H), 7.47-7.42 (m, 2H), 7.35-7.32 (m, 2H), 6.91 (dd, J = 9.2, 2.6 Hz, 1H), 6.77 (d, J = 9.2 Hz, 1H), 3.82 (s, 3H). **^{13}C NMR (100 MHz, DMSO-d_6)** δ 158.1, 155.0, 151.0, 142.5, 135.8, 130.3, 129.0, 128.8, 128.5, 128.1, 127.4, 127.0, 125.5, 119.9, 118.8, 118.7, 118.7, 106.5, 55.1. **HRMS (ESI)** calcd for $[M+H]^+$ $C_{20}H_{16}NO_2^+$, m/z: 302.1176, found: 302.1173.

1-(Isoquinolin-1-yl)-7-phenylnaphthalen-2-ol (3g)

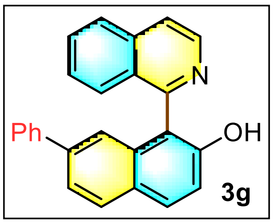

According to the general procedure, **3g** was obtained in **90% yield** as a yellow solid. **^{1}H NMR (400 MHz, DMSO-d_6)** δ 9.79 (s, 1H), 8.69 (d, *J* = 5.7 Hz, 1H), 8.05 (d, *J* = 8.3 Hz, 1H), 8.00 (d, *J* = 8.6 Hz, 2H), 7.91 (d, *J* = 5.7 Hz, 1H), 7.76-7.72 (m, 1H), 7.58 (dd, *J* = 8.4, 1.8 Hz, 1H), 7.51-7.48 (m, 2H), 7.38 (d, *J* = 8.9 Hz, 1H), 7.33-7.30 (m, 4H), 7.26-7.23 (m, 1H), 7.06 (d, *J* = 1.8 Hz, 1H). **^{13}C NMR (100 MHz, DMSO-d_6)** δ 157.8, 153.2, 142.5, 140.4, 138.1, 135.8, 134.1, 130.3, 129.7, 129.0, 128.8, 128.0, 127.5, 127.4, 127.1, 127.0, 126.9, 126.7, 122.1, 121.4, 120.1, 118.7, 118.5. **HRMS (ESI)** calcd for $[M+H]^+$ $C_{25}H_{18}NO^+$, m/z: 348.1383, found: 348.1380.

1-(Isoquinolin-1-yl)-6-phenylnaphthalen-2-ol (3h)

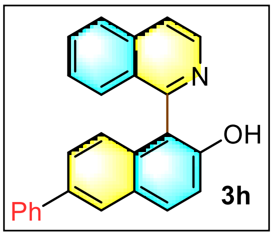

According to the general procedure, **3h** was obtained in **96% yield** as a white solid. **^{1}H NMR (400 MHz, DMSO-d_6)** δ 9.81 (s, 1H), 8.69 (d, *J* = 5.6 Hz, 1H), 8.21 (d, *J* = 1.9 Hz, 1H), 8.06 (dd, *J* = 8.6, 3.6 Hz, 2H), 7.92 (d, *J* = 5.7 Hz, 1H), 7.76-7.72 (m, 3H), 7.56 (dd, *J* = 8.8, 2.0 Hz, 1H), 7.49-7.41 (m, 5H), 7.35-7.31 (m, 1H), 6.95 (d, *J* = 8.8 Hz, 1H). **^{13}C NMR (100 MHz, DMSO-d_6)** δ 157.8, 153.0, 142.5, 140.1, 135.9, 134.4, 133.1, 130.3, 129.0, 128.1, 128.1, 127.5, 127.2, 127.0, 126.9, 126.7, 125.6, 125.5, 124.7, 120.0, 118.8, 118.4. **HRMS (ESI)** calcd for $[M+H]^+$ $C_{25}H_{18}NO^+$, m/z: 348.1383, found: 348.1381.

6-Bromo-1-(isoquinolin-1-yl)naphthalen-2-ol (3i)

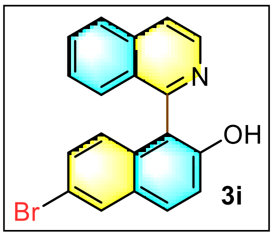

According to the general procedure, **3i** was obtained in **80% yield** as a white solid. **^{1}H NMR (400 MHz, DMSO-d_6)** δ 9.92 (s, 1H), 8.66 (d, *J* = 5.7 Hz, 1H), 8.17 (s, 1H), 8.05 (d, *J* = 8.2 Hz, 1H), 7.96 (d, *J* = 9.0 Hz, 1H), 7.91 (d, *J* = 5.7 Hz, 1H), 7.76-7.73 (m, 1H), 7.50-7.46 (m, 1H), 7.43-7.40 (m, 2H), 7.34 (dd, *J* = 9.1, 2.0 Hz, 1H), 6.80 (d, *J* = 9.0 Hz, 1H). **^{13}C NMR (100 MHz, DMSO-d_6)** δ 157.2, 153.2, 142.5, 135.8, 132.4, 130.3, 129.7, 129.3, 129.1, 129.0, 127.9, 127.5, 127.0, 126.7, 126.2, 120.1, 119.5, 118.6, 115.5. **HRMS (ESI)** calcd for $[M+H]^+$ $C_{19}H_{13}BrNO^+$, m/z: 350.0175, found: 350.0173.

Methyl 6-hydroxy-5-(isoquinolin-1-yl)-2-naphthoate (3j)

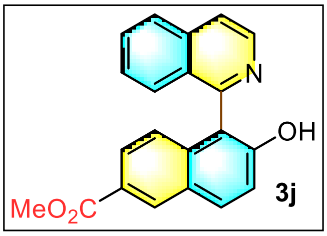

According to the general procedure, **3j** was obtained in **93% yield** as a white solid. **^{1}H NMR (400 MHz, DMSO-d_6)** δ 10.24 (s, 1H), 8.68 (d, *J* = 5.7 Hz, 1H), 8.64 (d, *J* = 1.9 Hz, 1H), 8.19 (d, *J* = 8.9 Hz, 1H), 8.06 (d, *J* = 8.3 Hz, 1H), 7.91 (d, *J* = 5.7 Hz, 1H), 7.76-7.71 (m, 2H), 7.50-7.42 (m, 3H), 6.96 (d, *J* = 8.9 Hz, 1H), 3.86 (s, 3H). **^{13}C NMR (100 MHz, DMSO-d_6)** δ 166.5, 157.2, 155.2, 142.6, 136.2, 135.9, 131.7, 131.0, 130.4, 128.0, 127.6, 127.1, 126.8, 126.7, 125.5, 124.3, 123.7, 120.2, 119.3, 118.7, 52.0. **HRMS (ESI)** calcd for $[M+H]^+$ $C_{21}H_{16}NO_3^+$, m/z: 330.1125, found: 330.1121.

1-(6-Hydroxy-5-(isoquinolin-1-yl)naphthalen-2-yl)ethan-1-one (3k)

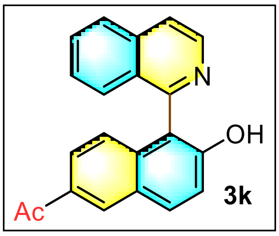

According to the general procedure, **3k** was obtained in **83% yield** as a yellow solid. **^{1}H NMR (400 MHz, DMSO-*d*$_6$)** δ 10.17 (s, 1H), 8.68-8.66 (m, 2H), 8.18 (d, *J* = 8.9 Hz, 1H), 8.08 (d, *J* = 8.2 Hz, 1H), 7.93 (dd, *J* = 5.8, 0.9 Hz, 1H), 7.79-7.75 (m, 1H), 7.71 (dd, *J* = 8.9, 1.9 Hz, 1H), 7.52-7.47 (m, 1H), 7.45 (d, *J* = 8.9 Hz, 1H), 7.40 (dd, *J* = 8.4, 1.1 Hz, 1H), 6.90 (d, *J* = 8.9 Hz, 1H), 2.64 (s, 3H). **^{13}C NMR (100 MHz, DMSO-*d*$_6$)** δ 197.9, 157.7, 155.7, 143.0, 136.6, 136.3, 132.4, 131.8, 131.3, 130.8, 128.4, 128.0, 127.5, 127.2, 127.1, 124.7, 124.6, 120.7, 119.6, 119.2, 27.0. **HRMS (ESI)** calcd for $[M+H]^+$ $C_{21}H_{16}NO_2^+$, m/z: 314.1176, found: 314.1172.

6-Hydroxy-5-(isoquinolin-1-yl)-2-naphthonitrile (3l)

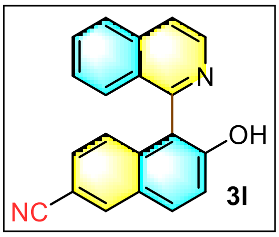

According to the general procedure, **3l** was obtained in **85% yield** as a white solid. **^{1}H NMR (400 MHz, DMSO-*d*$_6$)** δ 10.44 (s, 1H), 8.67 (d, *J* = 5.7 Hz, 1H), 8.54 (d, *J* = 1.7 Hz, 1H), 8.12 (d, *J* = 9.0 Hz, 1H), 8.06 (d, *J* = 8.2 Hz, 1H), 7.93 (d, *J* = 5.8 Hz, 1H), 7.77-7.73 (m, 1H), 7.53-7.46 (m, 3H), 7.41 (d, *J* = 8.4 Hz, 1H), 6.97 (d, *J* = 8.8 Hz, 1H). **^{13}C NMR (100 MHz, DMSO-*d*$_6$)** δ 156.7, 155.8, 142.5, 135.9, 135.5, 134.5, 130.9, 130.5, 127.9, 127.7, 127.1, 127.0, 126.7, 126.6, 125.2, 120.4, 120.0, 119.6, 118.9, 104.8. **HRMS (ESI)** calcd for $[M+H]^+$ $C_{20}H_{13}N_2O^+$, m/z: 297.1022, found: 297.1020.

6-Hydroxy-5-(isoquinolin-1-yl)-2-naphthaldehyde (3m)

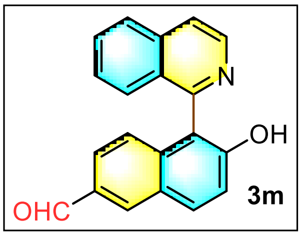

According to the general procedure, **3m** was obtained in **80% yield** as a white solid. **^{1}H NMR (400 MHz, DMSO-d_6)** δ 10.35 (s, 1H), 10.05 (s, 1H), 8.68 (d, J = 5.7 Hz, 1H), 8.56 (d, J = 1.7 Hz, 1H), 8.21 (d, J = 8.9 Hz, 1H), 8.07 (d, J = 8.2 Hz, 1H), 7.93 (d, J = 5.7 Hz, 1H), 7.78-7.74 (m, 1H), 7.63 (dd, J = 8.8, 1.7 Hz, 1H), 7.51-7.47 (m, 2H), 7.43-7.41 (m, 1H), 6.97 (d, J = 8.8 Hz, 1H). **^{13}C NMR (100 MHz, DMSO-d_6)** δ 192.4, 157.1, 155.9, 142.6, 137.0, 135.9, 135.1, 132.0, 131.2, 130.4, 127.9, 127.6, 127.1, 126.8, 126.7, 124.9, 123.1, 120.3, 119.4, 119.2. **HRMS (ESI)** calcd for $[M+H]^+$ $C_{20}H_{14}NO_2^+$, m/z: 300.1019, found: 300.1017.

Methyl 6-hydroxy-5-(isoquinolin-1-yl)-1-naphthoate (3n)

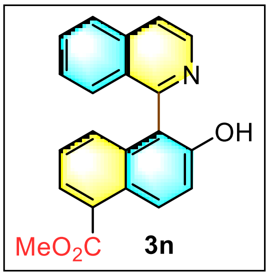

According to the general procedure, **3n** was obtained in **97% yield** as a white solid. **^{1}H NMR (400 MHz, DMSO-d_6)** δ 9.98 (s, 1H), 8.80 (d, J = 9.4 Hz, 1H), 8.67 (d, J = 5.7 Hz, 1H), 8.06 (d, J = 8.2 Hz, 1H), 7.91 (d, J = 5.7 Hz, 1H), 7.87 (dd, J = 7.2, 1.3 Hz, 1H), 7.76-7.72 (m, 1H), 7.51 (d, J = 9.4 Hz, 1H), 7.49-7.40 (m, 2H), 7.29 (dd, J = 8.6, 7.2 Hz, 1H), 7.09 (d, J = 8.6 Hz, 1H), 3.95 (s, 3H). **^{13}C NMR (100 MHz, DMSO-d_6)** δ 167.7, 157.5, 153.0, 142.5, 135.9, 134.4, 130.4, 129.0, 128.1, 127.6, 127.3, 127.2, 127.0, 126.8, 126.4, 125.4, 125.0, 120.2, 119.9, 119.1, 52.3. **HRMS (ESI)** calcd for $[M+H]^+$ $C_{21}H_{16}NO_3^+$, m/z: 330.1125, found: 330.1121.

1-(isoquinolin-1-yl)-4-phenylnaphthalen-2-ol (3o)

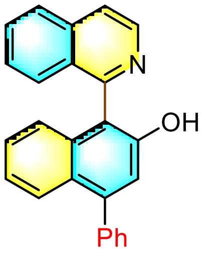

3o

According to the general procedure, **3o** was obtained in **91% yield** as a yellow solid. **^{1}H NMR (400 MHz, DMSO-d_6)** δ 9.81 (s, 1H), 8.69 (d, J = 5.6 Hz, 1H), 8.07 (d, J = 8.4 Hz, 1H), 7.92 (d, J = 5.6 Hz, 1H), 7.78-7.74 (m, 2H), 7.61-7.58 (m, 4H), 7.55-7.49 (m, 3H), 7.29 (s, 1H), 7.24-7.20 (m, 2H), 6.94-6.90 (m, 1H). **^{13}C NMR (100 MHz, DMSO-d_6)** δ 157.8, 152.0, 142.6, 141.5, 139.8, 135.8, 134.4, 130.3, 129.7, 128.6, 128.1, 127.7, 127.5, 127.0, 127.0, 126.4, 125.8, 125.6, 124.5, 123.0, 120.0, 119.0, 118.2. **HRMS (ESI)** calcd for $[M+H]^+$ $C_{25}H_{18}NO^+$, m/z: 348.1383, found: 348.1378.

4-bromo-1-(isoquinolin-1-yl)naphthalen-2-ol (3p)

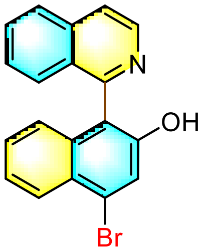

3p

According to the general procedure, **3p** was obtained in **64% yield** as a yellow solid. **^{1}H NMR (400 MHz, DMSO-d_6)** δ 10.10 (s, 1H), 8.67 (d, J = 5.6 Hz, 1H), 8.11 (dd, J = 8.4, 1.2 Hz, 1H), 8.06 (d, J = 8.4 Hz, 1H), 7.92 (d, J = 5.6 Hz, 1H), 7.78-7.73 (m, 2H), 7.50-7.46 (m, 1H), 7.45-7.41 (m, 2H), 7.32-7.28 (m, 1H), 6.89 (d, J = 8.4 Hz, 1H). **^{13}C NMR (100 MHz, DMSO-d_6)** δ 156.9, 152.6, 142.5, 135.8, 134.5, 130.4, 127.9, 127.6, 127.4, 127.0, 126.7, 126.5, 125.9, 124.7, 124.4, 122.6, 122.0, 120.2, 119.0. **HRMS (ESI)** calcd for $[M+H]^+$ $C_{19}H_{13}BrNO^+$, m/z: 350.0175, found: 350.0170.

1-(6-Methylisoquinolin-1-yl)naphthalen-2-ol (3q)

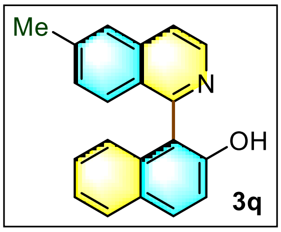

According to the general procedure, **3q** was obtained in **87% yield** as a white solid. **^{1}H NMR (400 MHz, DMSO-d_6)** δ 9.69 (s, 1H), 8.61 (d, J = 5.8 Hz, 1H), 7.94 (d, J = 8.9 Hz, 1H), 7.88 (d, J = 8.0 Hz, 1H), 7.80-7.78 (m, 2H), 7.38-7.28 (m, 3H), 7.29-7.24 (m, 1H), 7.22-7.18 (m, 1H), 6.85 (d, J = 8.4 Hz, 1H), 2.47 (s, 3H). **^{13}C NMR (100 MHz, DMSO-d_6)** δ 157.5, 152.7, 142.5, 140.2, 136.1, 133.8, 129.7, 129.5, 127.9, 127.8, 126.8, 126.5, 126.3, 125.6, 123.9, 122.6, 119.5, 118.5, 118.3, 21.4. **HRMS (ESI)** calcd for $[M+H]^+$ $C_{20}H_{16}NO^+$, m/z: 286.1226, found: 286.1224.

1-(5-Methoxyisoquinolin-1-yl)naphthalen-2-ol (3r)

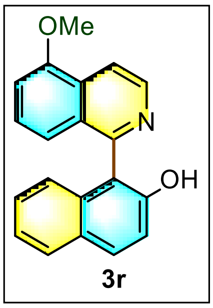

According to the general procedure, **3r** was obtained in **83% yield** as a white solid. **^{1}H NMR (400 MHz, DMSO-d_6)** δ 9.67 (s, 1H), 8.65 (d, J = 5.9 Hz, 1H), 8.05 (d, J = 5.8 Hz, 1H), 7.94 (d, J = 8.8 Hz, 1H), 7.88 (d, J = 8.1 Hz, 1H), 7.39-7.34 (m, 2H), 7.28-7.17 (m, 3H), 6.97 (d, J = 8.4 Hz, 1H), 6.81 (d, J = 8.4 Hz, 1H), 4.02 (s, 3H). **^{13}C NMR (100 MHz, DMSO-d_6)** δ 157.4, 154.2, 152.6, 142.2, 133.7, 129.7, 128.8, 128.1, 127.9, 127.7, 127.5, 126.3, 123.9, 122.6, 118.7, 118.6, 118.3, 113.8, 108.1, 55.9. **HRMS (ESI)** calcd for $[M+H]^+$ $C_{20}H_{16}NO_2^+$, m/z: 302.1176, found: 302.1174.

1-(4-Bromoisoquinolin-1-yl)naphthalen-2-ol (3s)

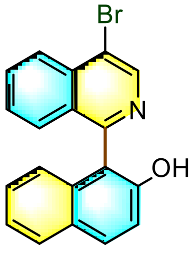

According to the general procedure, **3s** was obtained in **84% yield** as a yellow solid. **^{1}H NMR (400 MHz, DMSO-*d*$_6$)** δ 9.79 (s, 1H), 8.93 (s, 1H), 8.20 (d, *J* = 8.4 Hz, 1H), 7.97 (d, *J* = 8.8 Hz, 1H), 7.94-7.88 (m, 2H), 7.62-7.57 (m, 1H), 7.51-7.48 (m, 1H), 7.37 (d, *J* = 8.8 Hz, 1H), 7.30-7.26 (m, 1H), 7.23-7.19 (m, 1H), δ 6.89 (dd, J = 8.4, 1.2 Hz, 1H). **^{13}C NMR (100 MHz, DMSO-*d*$_6$)** δ 157.9, 152.7, 144.0, 134.2, 133.5, 132.1, 130.2, 129.4, 128.7, 128.0, 127.7, 127.7, 126.6, 125.6, 123.8, 122.8, 118.2, 118.2, 117.4. **HRMS (ESI)** calcd for $[M+H]^+$ $C_{19}H_{13}BrNO^+$, m/z: 350.0175, found: 350.0172.

1-(5-Bromoisoquinolin-1-yl)naphthalen-2-ol (3t)

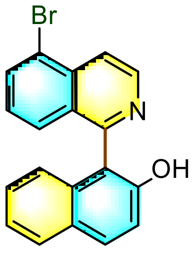

According to the general procedure, **3t** was obtained in **82% yield** as a yellow solid. **^{1}H NMR (400 MHz, DMSO-*d*$_6$)** δ 9.80 (s, 1H), 8.82 (d, *J* = 6.0 Hz, 1H), 8.10 (d, *J* = 7.0 Hz, 1H), 8.04 (d, *J* = 6.0 Hz, 1H), 7.97 (d, *J* = 8.9 Hz, 1H), 7.89 (d, *J* = 7.7 Hz, 1H), 7.49 (d, *J* = 8.4 Hz, 1H), 7.41-7.36 (m, 2H), 7.29-7.25 (m, 1H), 7.22-7.18 (m, 1H), 6.84 (d, *J* = 8.4 Hz, 1H). **^{13}C NMR (100 MHz, DMSO-*d*$_6$)** δ 159.2, 153.2, 144.7, 135.1, 134.6, 134.1, 130.6, 129.7, 128.7, 128.5, 128.2, 127.6, 127.0, 124.3, 123.2, 121.4, 118.7, 118.3. **HRMS (ESI)** calcd for $[M+H]^+$ $C_{19}H_{13}BrNO^+$, m/z: 350.0175, found: 350.0172.

1-(7-Bromoisoquinolin-1-yl)naphthalen-2-ol (3u)

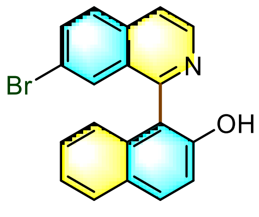

3u

According to the general procedure, **3u** was obtained in **94% yield** as a yellow solid. **^{1}H NMR (400 MHz, DMSO-*d*$_6$)** δ 9.86 (s, 1H), 8.73 (d, *J* = 5.7 Hz, 1H), 8.05 (d, *J* = 8.8 Hz, 1H), 7.98 (d, *J* = 8.9 Hz, 1H), 7.94 (d, *J* = 5.5 Hz, 1H), 7.91-7.86 (m, 2H), 7.56 (d, *J* = 2.0 Hz, 1H), 7.38 (d, *J* = 8.9 Hz, 1H), 7.30-7.21 (m, 2H), 6.89 (dd, *J* = 8.3, 1.3 Hz, 1H). **^{13}C NMR (100 MHz, DMSO-*d*$_6$)** δ 157.0, 152.7, 143.2, 134.5, 133.6, 133.4, 130.3, 129.7, 129.0, 128.6, 128.1, 127.8, 126.7, 123.8, 122.9, 120.3, 119.9, 118.3, 117.6. **HRMS (ESI)** calcd for $[M+H]^+$ $C_{19}H_{13}BrNO^+$, m/z: 350.0175, found: 350.0172.

1-(2-Hydroxynaphthalen-1-yl)isoquinoline-5-carbaldehyde (3v)

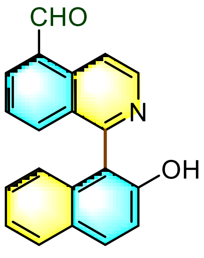

3v

According to the general procedure, **3v** was obtained in **74% yield** as a yellow solid. **^{1}H NMR (400 MHz, DMSO-*d*$_6$)** δ 10.49 (s, 1H), 9.82 (s, 1H), 9.03 (dd, *J* = 6.0, 0.9 Hz, 1H), 8.87 (d, *J* = 6.0 Hz, 1H), 8.42 (dd, *J* = 7.0, 1.4 Hz, 1H), 7.98 (d, *J* = 8.9 Hz, 1H), 7.91 (dd, *J* = 8.1, 1.4 Hz, 1H), 7.79 (d, *J* = 8.4 Hz, 1H), 7.71 (dd, *J* = 8.5, 6.9 Hz, 1H), 7.38 (d, *J* = 8.9 Hz, 1H), 7.31-7.27 (m, 1H), 7.25-7.21 (m, 1H), 6.86 (dd, *J* = 8.3, 1.2 Hz, 1H). **^{13}C NMR (100 MHz, DMSO-*d*$_6$)** δ 193.9, 158.8, 152.8, 145.4, 139.6, 134.0, 133.7, 133.2, 130.4, 130.2, 128.1, 127.8, 127.0, 126.6, 123.8, 122.8, 118.3, 118.1, 116.3. **HRMS (ESI)** calcd for $[M+H]^+$ $C_{20}H_{14}NO_2^+$, m/z: 300.1019, found: 300.1017.

1-(5-Nitroisoquinolin-1-yl)naphthalen-2-ol (3w)

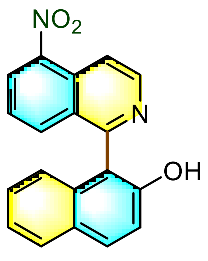

According to the general procedure, **3w** was obtained in **78% yield** as a yellow solid. **^{1}H NMR (400 MHz, DMSO-d_6)** δ 9.92 (s, 1H), 8.91 (d, J = 6.7 Hz, 1H), 8.60 (d, J = 7.5 Hz, 1H), 8.38 (d, J = 6.5 Hz, 1H), 8.02-7.87 (m, 3H), 7.69 (d, J = 7.9 Hz, 1H), 7.41-7.23 (m, 3H), 6.88 (d, J = 8.1 Hz, 1H). **^{13}C NMR (100 MHz, DMSO-d_6)** δ 159.1, 152.9, 145.8, 145.0, 134.2, 133.6, 130.4, 128.5, 128.5, 128.1, 127.9, 127.8, 126.8, 126.6, 123.7, 122.9, 118.3, 117.6, 114.5. **HRMS (ESI)** calcd for $[M+H]^+$ $C_{19}H_{13}N_2O_3^+$, m/z: 317.0921, found: 317.0924.

1-(3-methylisoquinolin-1-yl)-naphthalen-2-ol (3x)

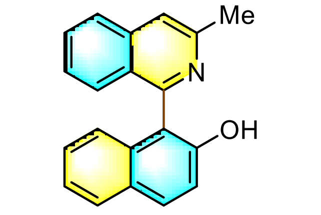

According to the general procedure, **3x** was obtained in **54% yield** as a white solid**.** **^{1}H NMR (400 MHz, DMSO-d_6)** δ 9.65 (s, 1H), 7.94 (dd, J = 8.4, 2.4 Hz, 2H), 7.88 (d, J = 7.6 Hz, 1H), 7.71 (s, 1H), 7.70-7.65 (m, 1H), 7.38-7.36 (m, 3H), 7.28-7.24 (m, 1H), 7.22-7.18 (m, 1H),6.84 (d, J = 1.2 Hz, 1H), 2.66 (s, 3H) **^{13}C NMR (100 MHz, DMSO-d_6)** δ 157.3, 152.6, 150.7, 136.6, 133.8, 130.2, 129.6, 127.9, 127.8, 126.7, 126.4, 126.4, 126.3, 126.3, 124.0, 122.6, 118.5, 118.3, 117.7, 24.0. **HRMS (ESI)** calcd for $[M+H]^{+:}$ $C_{20}H_{16}NO^+$, m/z: 286.1226, found: 286.1223.

1-(7-Bromoisoquinolin-1-yl)-7-methylnaphthalen-2-ol (3y)

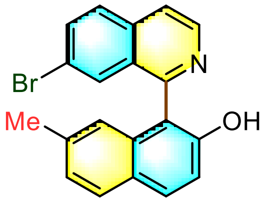

3y

According to the general procedure, **3y** was obtained in **90% yield** as a yellow solid. **^{1}H NMR (400 MHz, DMSO-d_6)** δ 9.74 (s, 1H), 8.73 (d, J = 5.7 Hz, 1H), 8.07 (d, J = 8.8 Hz, 1H), 7.95 (dd, J = 5.8, 0.9 Hz, 1H), 7.92-7.88 (m, 2H), 7.80 (d, J = 8.3 Hz, 1H), 7.54 (d, J = 2.1 Hz, 1H), 7.28 (d, J = 8.8 Hz, 1H), 7.13 (dd, J = 8.4, 1.7 Hz, 1H), 6.64 (s, 1H), 2.19 (s, 3H). **^{13}C NMR (100 MHz, DMSO-d_6)** δ 157.2, 152.8, 143.2, 135.8, 134.5, 133.8, 133.3, 130.0, 129.7, 129.0, 128.5, 128.0, 126.1, 125.0, 122.6, 120.2, 119.8, 117.2, 117.0, 21.5. **HRMS (ESI)** calcd for $[M+H]^+$ $C_{20}H_{15}BrNO^+$, m/z: 364.0332, found: 364.0329.

1-(7-Bromoisoquinolin-1-yl)-7-methoxynaphthalen-2-ol (3z)

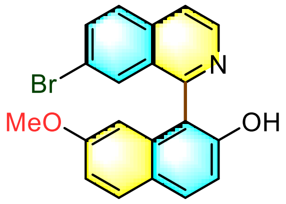

3z

According to the general procedure, **3z** was obtained in **85% yield** as a white solid. **^{1}H NMR (400 MHz, DMSO-d_6)** δ 9.77 (s, 1H), 8.74 (d, J = 5.7 Hz, 1H), 8.07 (d, J = 8.8 Hz, 1H), 7.95 (d, J = 5.7 Hz, 1H), 7.89 (dd, J = 8.8, 3.5 Hz, 2H), 7.83 (d, J = 9.0 Hz, 1H), 7.58 (s, 1H), 7.19 (d, J = 8.8 Hz, 1H), 6.99 (dd, J = 8.9, 2.5 Hz, 1H), 6.21 (d, J = 2.5 Hz, 1H), 3.47 (s, 3H). **^{13}C NMR (100 MHz, DMSO-d_6)** δ 157.8, 157.1, 153.3, 143.2, 134.9, 134.5, 133.3, 130.0, 129.8, 129.7, 128.8, 128.6, 123.3, 120.2, 119.8, 116.7, 115.6, 114.5, 103.1, 54.7. **HRMS (ESI)** calcd for $[M+H]^+$ $C_{20}H_{15}BrNO_2^+$, m/z: 380.0281, found: 380.0277.

1-(7-Bromoisoquinolin-1-yl)-7-phenylnaphthalen-2-ol (3aa)

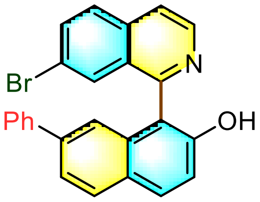

3aa

According to the general procedure, **3aa** was obtained in **93% yield** as a white solid. **^{1}H NMR (400 MHz, DMSO-d_6)** δ 9.91 (s, 1H), 8.76 (d, *J* = 5.7 Hz, 1H), 8.07 (dd, *J* = 8.8, 4.8 Hz, 1H), 8.04-8.01 (m, 2H), 7.98-7.96 (m, 1H), 7.92-7.88 (m, 1H), 7.63-7.60 (m, 2H), 7.40-7.32(m, 5H), 7.29-7.25 (m, 1H), 7.09 (d, *J* = 5.5 Hz, 1H). **^{13}C NMR (100 MHz, DMSO-d_6)** δ 156.8, 153.1, 143.2, 140.3, 138.3, 134.5, 133.9, 133.4, 130.1, 129.7, 129.0, 128.9, 128.6, 127.4, 127.1, 126.7, 122.2, 121.2, 120.3, 120.0, 118.4, 117.8. **HRMS (ESI)** calcd for $[M+H]^+$ $C_{25}H_{17}BrNO^+$, m/z: 426.0488, found: 426.0485.

1-(7-Bromoisoquinolin-1-yl)-6-phenylnaphthalen-2-ol (3ab)

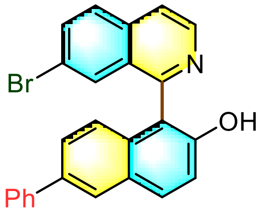

3ab

According to the general procedure, **3ab** was obtained in **83% yield** as a white solid. **^{1}H NMR (400 MHz, DMSO-d_6)** δ 9.90 (s, 1H), 8.75 (d, *J* = 5.7 Hz, 1H), 8.23 (s, 1H), 8.08 (dd, *J* = 8.9, 4.9 Hz, 2H), 7.98 (d, *J* = 5.8 Hz, 1H), 7.91 (d, *J* = 7.8 Hz, 1H), 7.75-7.73 (m, 2H), 7.61-7.57 (m, 2H), 7.49-7.33 (m, 4H), 6.97 (d, *J* = 8.6 Hz, 1H). **^{13}C NMR (100 MHz, DMSO-d_6)** δ 156.8, 152.9, 143.2, 140.0, 134.5, 134.5, 133.4, 132.9, 130.7, 129.7, 128.9, 128.5, 128.1, 127.2, 126.7, 125.8, 125.5, 124.5, 120.3, 119.9, 118.7, 117.5. **HRMS (ESI)** calcd for $[M+H]^+$ $C_{25}H_{17}BrNO^+$, m/z: 426.0488, found: 426.0484.

Methyl 5-(7-bromoisoquinolin-1-yl)-6-hydroxy-2-naphthoate (3ac)

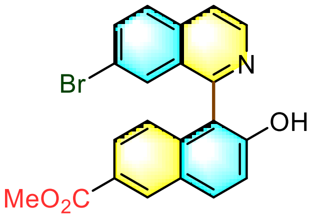

3ac

According to the general procedure, **3ac** was obtained in **91% yield** as a white solid. **^{1}H NMR (400 MHz, DMSO-d_6)** δ 10.31 (s, 1H), 8.74 (d, J = 5.6 Hz, 1H), 8.63 (d, J = 1.8 Hz, 1H), 8.20 (d, J = 8.8 Hz, 1H), 8.06 (d, J = 8.8 Hz, 1H), 7.96 (d, J = 6.0 Hz, 1H), 7.89 (dd, J = 8.8, 2.0 Hz, 1H), 7.74 (dd, J = 8.8, 2.0 Hz, 1H), 7.55 (d, J = 2.0 Hz, 1H), 7.47 (d, J = 9.2 Hz, 1H), 6.99 (d, J = 8.8 Hz, 1H), 3.86 (s, 3H). **^{13}C NMR (100 MHz, DMSO-d_6)** δ 166.3, 156.3, 155.1, 143.2, 136.0, 134.5, 133.4, 132.1, 131.0, 129.7, 128.9, 128.3, 126.8, 125.6, 124.2, 123.8, 120.4, 120.1, 119.2, 117.8, 52.0.**HRMS (ESI)** calcd for $[M+H]^+$ $C_{21}H_{15}BrNO_3^+$, m/z: 408.0230, found: 408.0228.

1-(6-Bromoisoquinolin-1-yl)-7-methoxynaphthalen-2-ol (3ad)

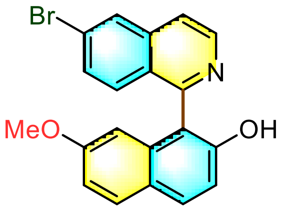

3ad

According to the general procedure, **3ad** was obtained in **93% yield** as a yellow solid. **^{1}H NMR (400 MHz, DMSO-d_6)** δ 9.70 (d, J = 1.8 Hz, 1H), 8.71 (d, J = 5.8 Hz, 1H), 8.37 (s, 1H), 7.88-7.80 (m, 3H), 7.63 (d, J = 9.0 Hz, 1H), 7.39 (d, J = 9.0 Hz, 1H), 7.18 (d, J = 8.8 Hz, 1H), 6.97 (d, J = 9.0 Hz, 1H), 6.19 (s, 1H), 3.45 (s, 3H). **^{13}C NMR (100 MHz, DMSO-d_6)** δ 158.2, 157.7, 153.3, 143.7, 137.2, 135.0, 130.5, 129.8, 129.7, 129.3, 129.0, 126.4, 124.0, 123.3, 119.1, 117.0, 115.6, 114.4, 103.2, 54.7. **HRMS (ESI)** calcd for $[M+H]^+$ $C_{20}H_{15}BrNO_2^+$, m/z: 380.0281, found: 380.0278.

Methyl 5-(6-bromoisoquinolin-1-yl)-6-hydroxy-2-naphthoate (3ae)

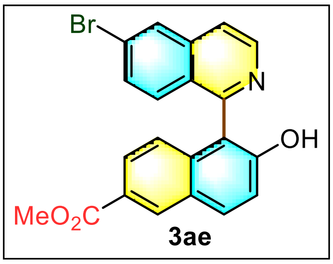

According to the general procedure, **3ae** was obtained in **86% yield** as a white solid. **^{1}H NMR (400 MHz, DMSO-d_6)** δ 10.25 (s, 1H), 8.72 (d, J = 5.6 Hz, 1H), 8.63 (d, J = 1.6 Hz, 1H), 8.39 (d, J = 2.0 Hz, 1H), 8.19 (d, J = 8.8 Hz, 1H), 7.91 (dd, J =6.0, 0.8 Hz, 1H), 7.72 (dd, J = 8.8, 1.6 Hz, 1H), 7.62 (dd, J = 8.8, 2.0 Hz, 1H), 7.45 (d, J = 8.8 Hz, 1H), 7.36 (d, J = 8.8 Hz, 1H), 6.96 (d, J = 8.8 Hz, 1H), 3.87 (s, 3H). **^{13}C NMR (100 MHz, DMSO-d_6)** δ 166.3, 157.4, 155.2, 143.7, 137.1, 136.0, 131.9, 130.9, 130.7, 129.1, 129.1, 126.8, 126.4, 125.5, 124.2, 124.1, 123.7, 119.3, 119.2, 118.0, 52.0. **HRMS (ESI)** calcd for $[M+H]^+$ $C_{21}H_{15}BrNO_3^+$, m/z: 408.0230, found: 408.0225.

1-(5-Bromoisoquinolin-1-yl)-7-methoxynaphthalen-2-ol (3af)

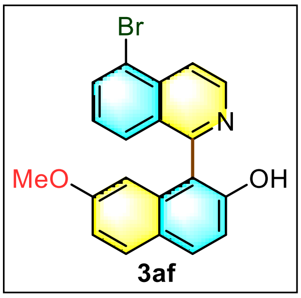

According to the general procedure, **3af** was obtained in **92% yield** as a white solid. **^{1}H NMR (400 MHz, DMSO-d_6)** δ 9.70 (s, 1H), 8.82 (d, J = 6.0 Hz, 1H), 8.13 (d, J = 7.3 Hz, 1H), 8.04 (d, J = 6.0 Hz, 1H), 7.88 (d, J = 8.8 Hz, 1H), 7.82 (d, J = 9.0 Hz, 1H), 7.52 (d, J = 8.4 Hz, 1H), 7.45-7.41 (m, 1H), 7.17 (d, J = 8.9 Hz, 1H), 6.97 (dd, J = 8.9, 2.5 Hz, 1H), 6.16 (s, 1H), 3.45 (s, 3H). **^{13}C NMR (100 MHz, DMSO-d_6)** δ 158.8, 157.8, 153.4, 144.3, 134.9, 134.6, 134.1, 129.9, 129.7, 129.1, 128.2, 127.2, 123.3, 120.9, 118.2, 117.1, 115.6, 114.4, 103.1, 54.7. **HRMS (ESI)** calcd for $[M+H]^+$ $C_{20}H_{15}BrNO_2^+$, m/z: 380.0281, found: 380.0278.

1-(5-Bromoisoquinolin-1-yl)-7-phenylnaphthalen-2-ol (3ag)

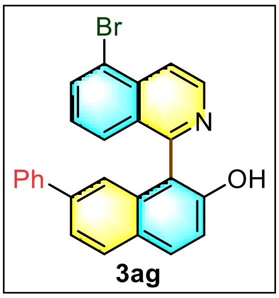

According to the general procedure, **3ag** was obtained in **81% yield** as a white solid. **^{1}H NMR (400 MHz, DMSO-d_6)** δ 9.84 (s, 1H), 8.84 (d, J = 6.0 Hz, 1H), 8.13 (dd, J = 7.4, 1.1 Hz, 1H), 8.06 (dd, J = 6.0, 0.9 Hz, 1H), 8.01 (d, J = 8.6 Hz, 2H), 7.60 (dd, J = 8.5, 1.8 Hz, 1H), 7.55 (d, J = 8.4 Hz, 1H), 7.44 (dd, J = 8.5, 7.4 Hz, 1H), 7.38-7.31 (m, 5H), 7.28-7.24 (m, 1H), 7.04 (s, 1H). **^{13}C NMR (100 MHz, DMSO-d_6)** δ 158.5, 153.3, 144.3, 140.3, 138.3, 134.6, 134.2, 133.9, 129.9, 129.2, 129.0, 128.9, 128.4, 127.4, 127.2, 127.1, 126.7, 122.2, 121.2, 120.9, 118.4, 118.3, 118.1. **HRMS (ESI)** calcd for $[M+H]^+$ $C_{25}H_{17}BrNO^+$, m/z: 426.0488, found: 426.0484.

Methyl 5-(5-bromoisoquinolin-1-yl)-6-hydroxy-2-naphthoate (3ah)

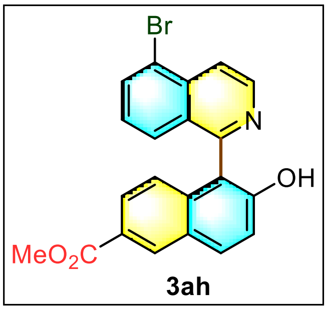

According to the general procedure, **3ah** was obtained in **90% yield** as a white solid. **^{1}H NMR (400 MHz, DMSO-d_6)** δ 10.24 (s, 1H), 8.83 (d, J = 6.0 Hz, 1H), 8.63 (d, J = 1.9 Hz, 1H), 8.20 (d, J = 8.9 Hz, 1H), 8.14 (dd, J = 7.0, 1.5 Hz, 1H), 8.06 (dd, J = 6.0, 0.9 Hz, 1H), 7.72 (dd, J = 8.9, 1.8 Hz, 1H), 7.48-7.41 (m, 3H), 6.93 (d, J = 8.9 Hz, 1H), 3.87 (s, 3H). **^{13}C NMR (100 MHz, DMSO)** δ 166.3, 158.0, 155.2, 144.3, 136.0, 134.6, 134.2, 131.9, 130.9, 129.1, 128.5, 127.0, 126.7, 125.6, 124.2, 123.7, 121.0, 119.2, 118.4, 118.1, 52.0. **HRMS (ESI)** calcd for $[M+H]^+$ $C_{21}H_{15}BrNO_3^+$, m/z: 408.024, found: 408.0226.

7-Methoxy-1-(6-methylisoquinolin-1-yl)naphthalen-2-ol (3ai)

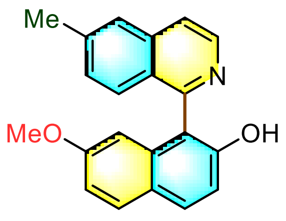

3ai

According to the general procedure, **3ai** was obtained in **71% yield** as a white solid. **^{1}H NMR (400 MHz, DMSO-d_6)** δ 9.59 (s, 1H), 8.60 (d, J = 5.7 Hz, 1H), 7.85 (d, J = 8.8 Hz, 1H), 7.82-7.79 (m, 2H), 7.78 (d, J = 0.7 Hz, 1H), 7.36-7.30 (m, 2H), 7.17 (d, J = 8.8 Hz, 1H), 6.96 (dd, J = 8.9, 2.5 Hz, 1H), 6.18 (d, J = 2.5 Hz, 1H), 3.43 (s, 3H), 2.49 (s, 3H). **^{13}C NMR (100 MHz, DMSO-d_6)** δ 157.6, 157.6, 153.2, 142.6, 140.1, 136.1, 135.1, 129.6, 129.5, 129.4, 126.8, 126.4, 125.7, 123.3, 119.4, 117.7, 115.7, 114.2, 103.4, 54.6, 21.5. **HRMS (ESI)** calcd for $[M+H]^+$ $C_{21}H_{18}NO_2^+$, m/z: 316.1332, found: 316.1330.

7-Methoxy-1-(5-methoxyisoquinolin-1-yl)naphthalen-2-ol (3aj)

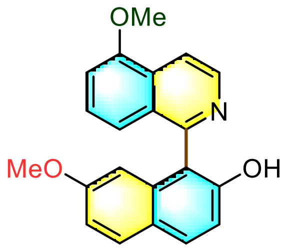

3aj

According to the general procedure, **3aj** was obtained in **95% yield** as a yellow solid. **^{1}H NMR (400 MHz, DMSO-d_6)** δ 9.59 (s, 1H), 8.66 (d, J = 5.8 Hz, 1H), 8.04 (d, J = 5.8 Hz, 1H), 7.85 (d, J = 8.8 Hz, 1H), 7.81 (d, J = 8.9 Hz, 1H), 7.38 (t, J = 8.1 Hz, 1H), 7.18 (dd, J = 8.3, 3.4 Hz, 2H), 7.01 (d, J = 8.4 Hz, 1H), 6.96 (dd, J = 8.9, 2.5 Hz, 1H), 6.16 (d, J = 2.5 Hz, 1H), 4.02 (s, 3H), 3.43 (s, 3H). **^{13}C NMR (100 MHz, DMSO-d_6)** δ 157.6, 157.5, 154.2, 153.3, 142.2, 135.0, 129.6, 129.5, 128.7, 128.1, 127.4, 123.3, 118.6, 117.9, 115.7, 114.3, 113.8, 108.0, 103.3, 55.9, 54.6. **HRMS (ESI)** calcd for $[M+H]^+$ $C_{21}H_{18}NO_3^+$, m/z: 332. 1281, found: 332.1278.

4. Procedure for the preparation of racemic QUINOL N-oxides

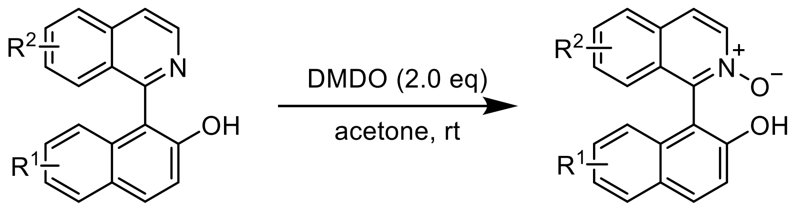

3 4

To a solution of compound **3** (0.5 mmol) in acetone (8 mL) was added the freshly prepared DMDO[1] (1.0 mmol, 0.06 mol/L in acetone). The reaction was complete after stirring for 2 hours, and the mixture was then concentrated under reduced pressure. The residue was purified by flash chromatography eluted with DCM/EA (5/1 to 1/1) to afford **4.**

1-(2-hydroxynaphthalen-1-yl)isoquinoline 2-oxide (4a)

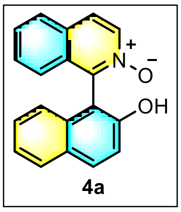

According to the general procedure, **4a** was obtained in **92% yield** as a white solid. **^{1}H NMR (400 MHz, DMSO-*d*$_6$)** δ 9.97 (s, 1H), 8.35 (d, *J* = 6.8 Hz, 1H), 8.06-8.03 (m, 2H), 7.99 (d, *J* = 8.8 Hz, 1H), 7.91 (d, *J* = 7.6 Hz, 1H), 7.58 (t, *J* = 7.2 Hz, 1H), 7.46 (t, *J* = 7.2 Hz, 1H), 7.36 (d, *J* = 8.8 Hz, 1H), 7.32-7.24 (m, 2H), 7.05 (d, *J* = 8.4 Hz, 1H), 6.83 (d, *J* = 8.0 Hz, 1H). **^{13}C NMR (100 MHz, DMSO-*d*$_6$)** δ 153.7, 142.0, 137.6, 132.7, 130.9, 129.8, 129.3, 128.2, 128.1, 127.9, 127.8, 127.2, 126.8, 124.6, 124.0, 123.4, 122.9, 118.6, 110.7. **HRMS (ESI)** calcd for [M+H] $C_{19}H_{14}NO_2^+$, m/z: 288.1019; found: 288.1019.

1-(2-hydroxy-7-methylnaphthalen-1-yl)isoquinoline 2-oxide (4b)

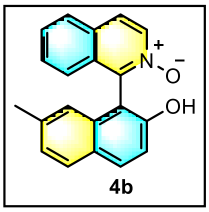

According to the general procedure, **4b** was obtained in **90% yield** as a white solid. **^{1}H NMR (400 MHz, DMSO-*d*$_6$)** δ 9.87 (s, 1H), 8.35 (d, *J* = 7.1 Hz, 1H), 8.03 (dd, *J* = 7.2, 4.0 Hz, 2H), 7.92 (d, *J* = 8.8 Hz, 1H), 7.81 (d, *J* = 8.3 Hz, 1H), 7.57 (t, *J* = 7.3 Hz, 1H), 7.46 (t, *J* = 7.7 Hz, 1H), 7.27 (d, *J* = 8.8 Hz, 1H), 7.14 (dd, *J* = 8.3, 1.6 Hz, 1H), 7.06 (d, *J* = 8.4 Hz, 1H), 6.61 (s, 1H), 2.20 (s, 3H). **^{13}C NMR (100 MHz, DMSO-*d*$_6$)** δ 153.8, 142.2, 137.6, 136.1, 132.9, 130.6, 129.8, 129.2, 128.2, 128.1, 127.8, 127.2, 126.2, 125.1, 124.7, 123.9, 122.2, 117.6, 110.2, 21.5. **HRMS (ESI)** calcd for [M+H] $C_{20}H_{16}NO_2^+$, m/z: 302.1176; found: 302.1173.

1-(2-hydroxy-6-methylnaphthalen-1-yl)isoquinoline 2-oxide (4c)

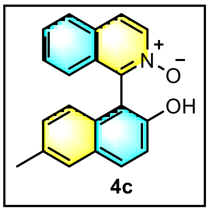

According to the general procedure, **4c** was obtained in **82% yield** as a white solid. **^{1}H NMR (400 MHz, DMSO-d_6)** δ 9.82 (s, 1H), 8.35 (d, *J* = 7.2 Hz, 1H), 8.04 (d, *J* = 7.2 Hz, 1H), 8.03 (d, *J* = 8.4 Hz, 1H), 7.89 (d, *J* = 9.2 Hz, 1H), 7.68 (s, 1H), 7.59-7.55 (m, 1H), 7.47-7.43 (m, 1H), 7.31 (d, *J* = 9.2 Hz, 1H), 7.10 (dd, *J* = 8.4, 1.6 Hz, 1H), 7.04 (d, *J* = 8.4 Hz, 1H), 6.75 (d, *J* = 8.4 Hz, 1H), 2.39 (s, 3H). **^{13}C NMR (100 MHz, DMSO-d_6)** δ 153.1, 142.2, 137.5, 131.9, 130.8, 130.2, 129.8, 129.2, 128.9, 128.1, 128.1, 127.8, 127.2, 127.1, 124.7, 123.9, 123.4, 118.6, 110.7, 20.9. **HRMS (ESI)** calcd for [M+H] $C_{20}H_{16}NO_2^+$, m/z: 302.1176; found: 302.1173.

1-(2-hydroxy-6-phenylnaphthalen-1-yl)isoquinoline 2-oxide (4d)

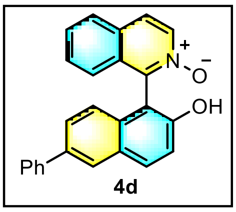

According to the general procedure, **4d** was obtained in **95% yield** as a white solid. **^{1}H NMR (400 MHz, DMSO-d_6)** δ 10.03 (s, 1H), 8.37 (d, *J* = 7.2 Hz, 1H), 8.23 (d, *J* = 2.0 Hz, 1H), 8.10-8.04 (m, 3H), 7.75-7.72 (m, 2H), 7.63-7.57 (m, 2H), 7.51-7.45 (m, 3H), 7.39 (d, *J* = 8.8 Hz, 1H), 7.37-7.33 (m, 1H), 7.11 (d, *J* = 8.4 Hz, 1H), 6.93 (d, *J* = 8.8 Hz, 1H). **^{13}C NMR (100 MHz, DMSO-d_6)** δ 154.0, 141.9, 140.1, 137.6, 134.8, 132.0, 131.4, 129.8, 129.3, 129.0, 128.3, 128.1, 127.9, 127.3, 127.2, 126.7, 126.0, 125.8, 124.7, 124.2, 124.1, 119.1, 110.6. **HRMS (ESI)** calcd for [M+H] $C_{25}H_{18}NO_2^+$, m/z: 364.1332; found: 364.1328.

1-(2-hydroxy-7-methoxynaphthalen-1-yl)isoquinoline 2-oxide (4e)

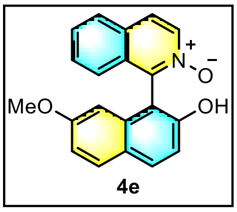

According to the general procedure, **4e** was obtained in **78% yield** as a white solid. **^{1}H NMR (400 MHz, DMSO-d_6)** δ 9.69 (s, 1H), 8.35 (d, J = 7.2 Hz, 1H), 8.03 (d, J = 7.2, 1H), 8.02 (d, J = 8.4, 1H), 7.90 (d, J = 8.8 Hz, 1H), 7.59-7.55 (m, 1H), 7.49-7.44 (m, 1H), 7.36 (d, J = 2.8 Hz, 1H), 7.32 (d, J = 8.8 Hz, 1H), 7.05 (d, J = 8.4 Hz, 1H), 6.96 (dd, J = 9.2, 2.8 Hz, 1H), 6.76 (d, J = 9.2 Hz, 1H), 3.83 (s, 3H). **^{13}C NMR (100 MHz, DMSO-d_6)** δ 155.3, 152.0, 142.1, 137.5, 129.8, 129.7, 129.2, 128.9, 128.1, 127.8, 127.8, 127.2, 125.0, 124.7, 124.0, 119.2, 119.0, 111.0, 106.9, 55.2. **HRMS (ESI)** calcd for [M+H] $C_{20}H_{16}NO_3^+$, m/z: 318.1125; found: 318.1121.

1-(2-hydroxynaphthalen-1-yl)-6-methylisoquinoline 2-oxide (4f)

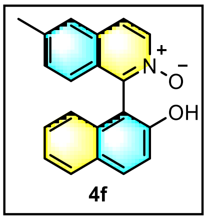

According to the general procedure, **4f** was obtained in **86% yield** as a white solid. **^{1}H NMR (400 MHz, DMSO-d_6)** δ 9.97 (s, 1H), 8.32 (d, J = 6.4 Hz, 1H), 7.99-7.90 (m, 3H), 7.81 (s, 1H), 7.36-7.23 (m, 4H), 6.96 (d, J = 8.4 Hz, 1H), 6.82 (d, J = 8.0 Hz, 1H), 2.43 (s, 3H). **^{13}C NMR (100 MHz, DMSO-d_6)** δ 153.8, 141.9, 137.8, 137.5, 132.7, 131.3, 130.8, 128.4, 128.2, 128.0, 127.9, 126.8, 126.2, 124.7, 123.4, 123.4, 122.9, 118.7, 110.8, 21.1. **HRMS (ESI)** calcd for [M+H] $C_{20}H_{16}NO_2^+$, m/z: 302.1176; found: 302.1174.

7-bromo-1-(2-hydroxynaphthalen-1-yl)isoquinoline 2-oxide (4g)

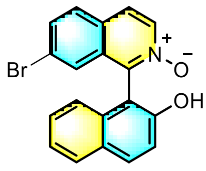

4g

According to the general procedure, **4g** was obtained in **78% yield** as a white solid. **^{1}H NMR (400 MHz, DMSO-d_6)** δ 10.03 (s, 1H), 8.40 (d, *J* = 7.2 Hz, 1H), 8.08 (d, *J* = 7.2 Hz, 1H), 8.03-8.00 (m, 2H), 7.92 (dd, *J* = 8.0, 1.5 Hz, 1H), 7.72 (dd, *J* = 8.6, 2.0 Hz, 1H), 7.36 (d, *J* = 9.0 Hz, 1H), 7.33-7.27 (m, 2H), 7.13 (d, *J* = 1.9 Hz, 1H), 6.86 (dd, *J* = 7.8, 1.6 Hz, 1H). **^{13}C NMR (100 MHz, DMSO-d_6)** δ 153.7, 141.1, 138.3, 132.4, 131.3, 131.1, 130.6, 129.7, 128.4, 127.9, 127.1, 126.7, 125.9, 124.0, 123.2, 123.1, 122.7, 118.6, 109.9. **HRMS (ESI)** calcd for [M+H] $C_{19}H_{13}BrNO_2^+$, m/z: 366.0124; found: 366.0121.

7-bromo-1-(2-hydroxy-6-phenylnaphthalen-1-yl)isoquinoline 2-oxide (4h)

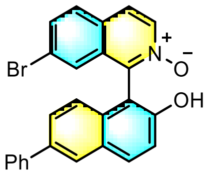

4h

According to the general procedure, **4h** was obtained in **86% yield** as a white solid. **^{1}H NMR (400 MHz, DMSO-d_6)** δ 10.12 (s, 1H), 8.42 (d, *J* = 7.2 Hz, 1H), 8.23 (s, 1H), 8.11-8.09 (m, 2H), 8.03 (d, *J* = 8.4 Hz, 1H), 7.75-7.71 (m, 3H), 7.64 (d, *J* = 8.4 Hz, 1H), 7.49-7.45 (m, 2H), 7.41 (d, *J* = 8.8 Hz, 1H), 7.35 (t, *J* = 7.2 Hz, 1H), 7.20 (d, *J* = 2.0 Hz, 1H), 6.96 (d, *J* = 8.8 Hz, 1H). **^{13}C NMR (100 MHz, DMSO-d_6)** δ 154.4, 141.5, 140.5, 138.8, 135.4, 132.2, 132.2, 131.6, 131.1, 130.2, 129.2, 129.4 128.7, 127.7, 127.2, 126.7, 126.4, 124.5, 124.5, 123.3, 119.5, 110.3. **HRMS (ESI)** calcd for [M+H] $C_{25}H_{17}BrNO_2^+$, m/z: 442.0437; found: 442.0432.

5. Procedure for the kinetic resolution of QUINOL N-oxides

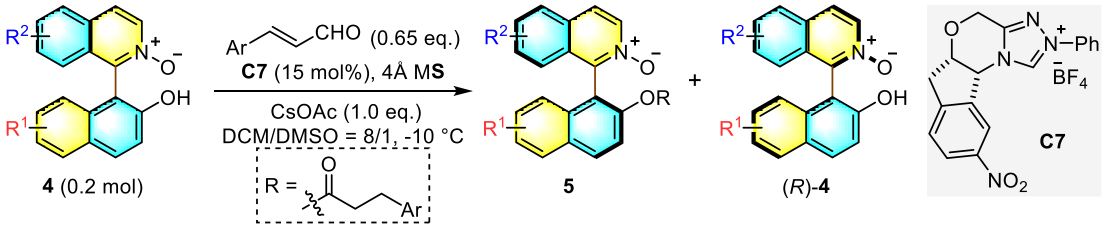

Under argon atmosphere, substrate **4** (0.2 mmol), cinnamaldehyde (0.13 mmol, 0.65 eq.), **C7** (13 mg, 0.03 mmol), activated 4Å MS (400 mg), solvent (18 mL, CH_2Cl_2/DMSO = 8/1), and CsOAc (0.2 mmol, 1.0 eq.) were added sequentially to a dried Schlenk flask. The mixture was stirred at -10 ℃ untill the aldehyde was completely consumed (monitored by TLC). After removing the molecular sieve by filtration, the obtained filtrate was diluted with 30 mL of water, and then extracted with DCM (30 mL × 3). The combined organic phase was washed with 30 mL brine, dried over anhydrous Na_2SO_4 and concentrated to afford crude products, which were purified by column chromatography eluted with DCM/EA (20/1 to 1/1) to afford the pure products **5** and (*R*)-**4**.

The racemic products **5** were prepared according to the following route.

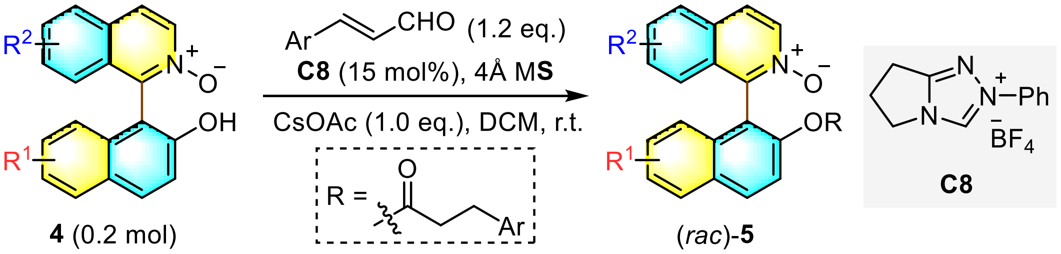

(1) Kinetic resolution of 4a with 4-bromocinnamaldehyde (3 days)

(*R*)-1-(2-hydroxynaphthalen-1-yl)isoquinoline 2-oxide ((*R*)-4a)

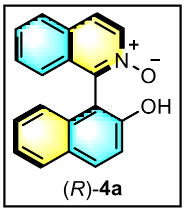

(*R*)-4a was obtained in **43% yield** and **>99% ee** as a white solid. **^{1}H NMR (400 MHz, DMSO-d_6)** δ 9.97 (s, 1H), 8.35 (d, *J* = 6.8 Hz, 1H), 8.06-8.03 (m, 2H), 7.99 (d, *J* = 8.8 Hz, 1H), 7.91 (d, *J* = 7.6 Hz, 1H), 7.58 (t, *J* = 7.2 Hz, 1H), 7.46 (t, *J* = 7.2 Hz, 1H), 7.36 (d, *J* = 8.8 Hz, 1H), 7.32-7.24 (m, 2H), 7.05 (d, *J* = 8.4 Hz, 1H), 6.83 (d, *J* = 8.0 Hz, 1H). **^{13}C NMR (100 MHz, DMSO-d_6)** δ 153.7, 142.0, 137.6, 132.7, 130.9, 129.8, 129.3, 128.2, 128.1, 127.9, 127.8, 127.2, 126.8, 124.6, 124.0, 123.4, 122.9, 118.6, 110.7. **HRMS (ESI)** calcd for [M+H] $C_{19}H_{14}NO_2^+$, m/z: 288.1019; found: 288.1019. **HPLC analysis:** HPLC DAICEL CHIRALCEL AD-H, *n*-hexane/isopropanol = 75/25, 1.0 mL/min, λ = 254 nm, t_R (major) = 9.9 min, t_R (minor) = 17.7 min, ee = >99%.

Chiral HPLC spectrum of (*rac*)-**4a**

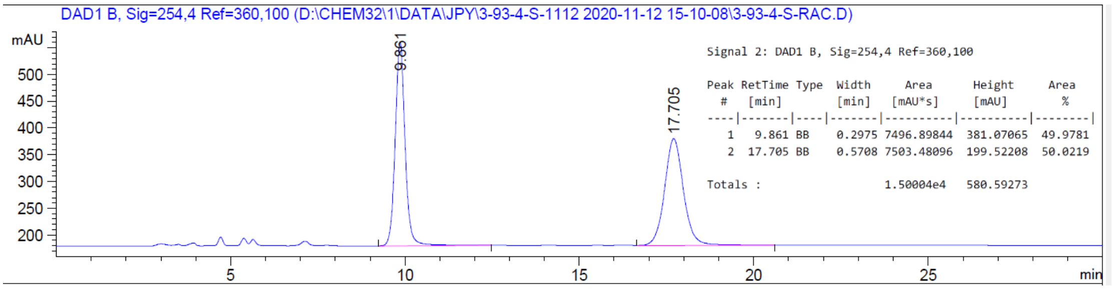

Chiral HPLC spectrum of (*R*)-**4a**

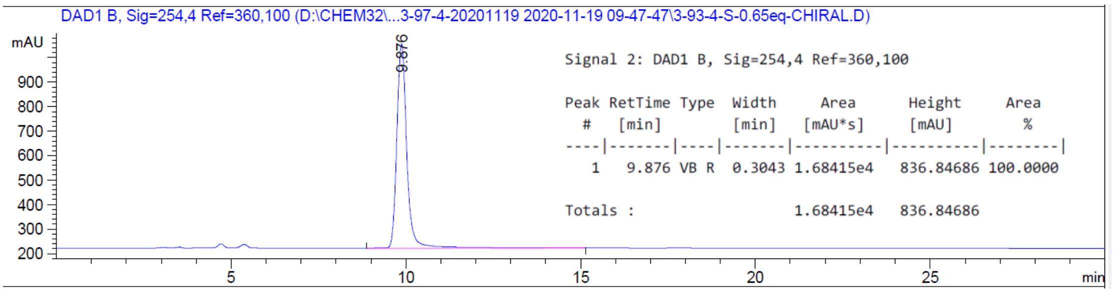

(*S*)-1-(2-((3-(4-bromophenyl)propanoyl)oxy)naphthalen-1-yl)isoquinoline 2-oxide (5a)

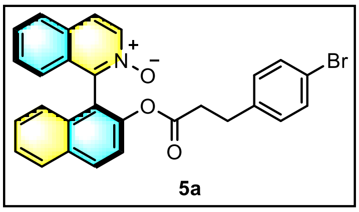

5a was obtained in **54% yield** and **76% ee** as a yellow solid. **^{1}H NMR (400 MHz, DMSO-d_6)** δ 8.40 (d, *J* = 7.2 Hz, 1H), 8.21 (d, *J* = 8.8 Hz, 1H), 8.10 (d, *J* = 7.2 Hz, 2H), 8.05 (d, *J* = 8.0 Hz, 1H), 7.60-7.53 (m, 3H), 7.43-7.38 (m, 2H), 7.34-7.31 (m, 2H), 7.07 (d, *J* = 8.6 Hz, 1H), 6.92(d, *J* = 8.6 Hz, 1H), 6.89(d, *J* = 8.4 Hz, 2H), 2.57-2.36 (m, 4H). **^{13}C NMR (100 MHz, DMSO-d_6)** δ 170.0, 147.1, 139.4, 139.1, 137.5, 131.6, 131.2, 131.1, 130.7, 130.1, 129.5, 129.3, 128.5, 128.0, 127.8, 127.5, 127.3, 126.1, 124.7, 123.9, 122.3, 119.7, 119.2, 34.4, 29.0. **HRMS (ESI)** calcd for [M+H] $C_{28}H_{21}BrNO_3^+$, m/z: 498.0699; found: 498.0693. **HPLC analysis:** HPLC DAICEL CHIRALCEL AD-H, *n*-hexane/isopropanol = 60/40, 1.0 mL/min, λ = 254 nm, t_R (major) = 13.4 min, t_R (minor) = 29.7 min, ee = 76%.

Chiral HPLC spectrum of (*rac*)-**5a**

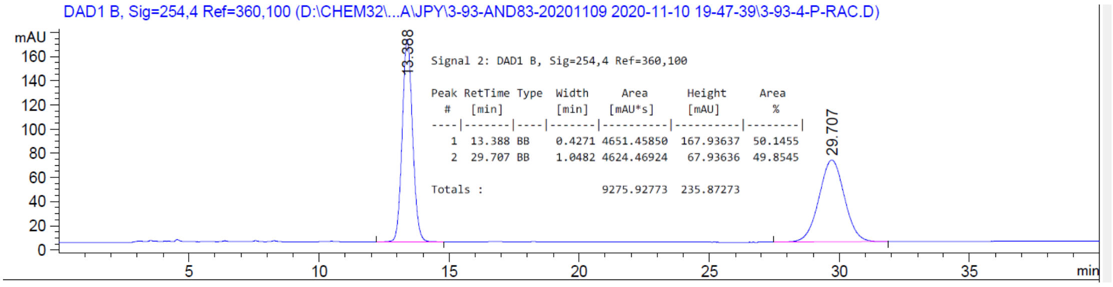

Chiral HPLC spectrum of (*S*)-**5a**

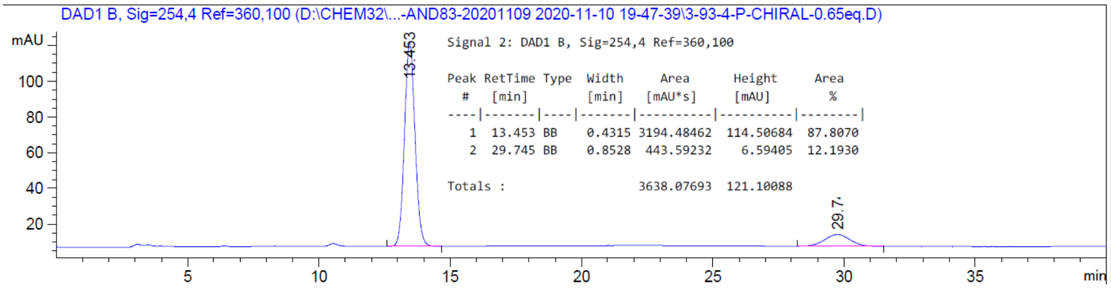

(2) Kinetic resolution of 4b with 4-bromocinnamaldehyde (3 days)

(*R*)-1-(2-hydroxy-7-methylnaphthalen-1-yl)isoquinoline 2-oxide ((*R*)-4b)

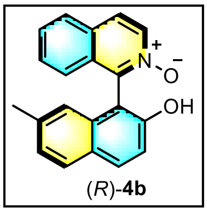

(*R*)-4b was obtained in **41% yield** and > **99% ee** as a white solid. **^{1}H NMR (400 MHz, DMSO-d_6)** δ 9.87 (s, 1H), 8.35 (d, *J* = 7.1 Hz, 1H), 8.04 (d, *J* = 7.2, Hz, 1H), 8.03 (d, *J* = 8.4, Hz, 1H), 7.92 (d, *J* = 8.8 Hz, 1H), 7.81 (d, *J* = 8.3 Hz, 1H), 7.57 (t, *J* = 7.3 Hz, 1H), 7.46 (t, *J* = 7.7 Hz, 1H), 7.27 (d, *J* = 8.8 Hz, 1H), 7.14 (dd, *J* = 8.3, 1.6 Hz, 1H), 7.06 (d, *J* = 8.4 Hz, 1H), 6.61 (s, 1H), 2.20 (s, 3H). **^{13}C NMR (100 MHz, DMSO-d_6)** δ 153.8, 142.2, 137.6, 136.1, 132.9, 130.6, 129.8, 129.2, 128.2, 128.1, 127.8, 127.2, 126.2, 125.1, 124.7, 123.9, 122.2, 117.6, 110.2, 21.5. **HRMS (ESI)** calcd for [M+H] $C_{20}H_{16}NO_2^+$, m/z: 302.1176; found: 302.1173. **HPLC analysis:** HPLC DAICEL CHIRALCEL AD-H, *n*-hexane/isopropanol = 75/25, 1.0 mL/min, λ = 254 nm, t_R (major) = 7.6 min, t_R (minor) = 15.6 min, ee > 99%.

Chiral HPLC spectrum of (*rac*)-**4b**

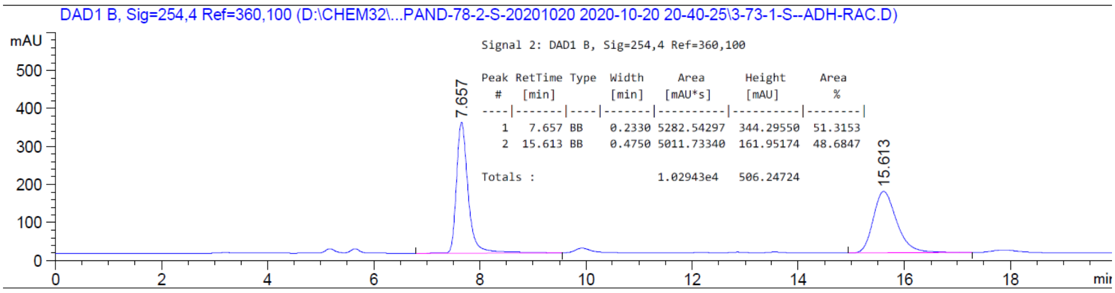

Chiral HPLC spectrum of (*R*)-**4b**

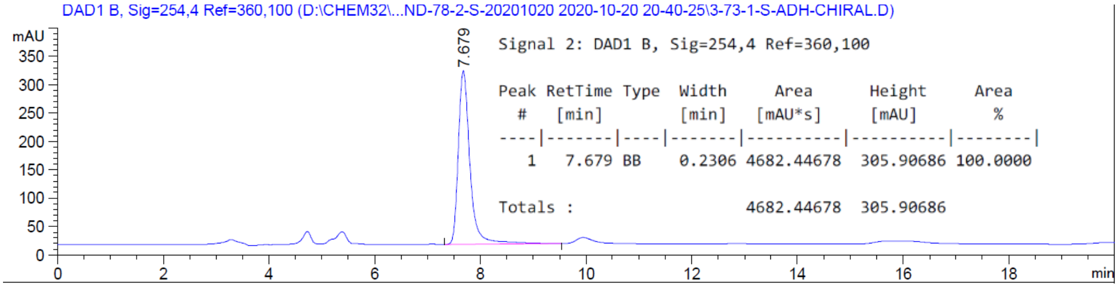

(*S*)-1-(2-((3-(4-bromophenyl)propanoyl)oxy)-7-methylnaphthalen-1-yl)isoquinoline 2-oxide (5b)

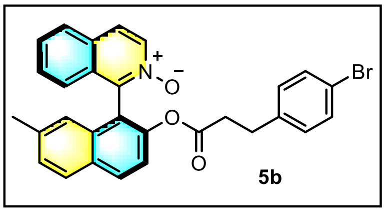

5b was obtained in **55% yield** and **74% ee** as a yellow solid. **^{1}H NMR (400 MHz, DMSO-d_6)** δ 8.38 (d, *J* = 7.2 Hz, 1H), 8.14 (d, *J* = 8.9 Hz, 1H), 8.10 (d, *J* = 7.2 Hz, 1H), 8.05 (d, *J* = 8.1 Hz, 1H), 8.00 (d, *J* = 8.4 Hz, 1H), 7.61-7.57 (m, 1H), 7.47-7.39 (m, 3H), 7.33 (d, *J* = 8.3 Hz, 2H), 6.91 (d, *J* = 8.6, 1H), 6.88 (d, *J* = 8.4 Hz, 2H), 6.83 (s, 1H), 2.48-2.34 (m, 4H), 2.26 (s, 3H). **^{13}C NMR (100 MHz, DMSO-d_6)** δ 170.0, 147.1, 139.6, 139.2, 137.5, 137.0, 131.9, 131.1, 130.4, 130.1, 129.5, 129.4, 129.3, 128.4, 128.3, 128.0, 127.8, 127.3, 124.6, 123.9, 123.4, 121.3, 119.2, 119.1, 34.4, 29.0, 21.4. **HRMS (ESI)** calcd for [M+H] $C_{29}H_{23}BrNO_3^+$, m/z: 512.0856; found: 512.0851. **HPLC analysis:** HPLC DAICEL CHIRALCEL IE, *n*-hexane/isopropanol = 60/40, 1.0 mL/min, λ = 230 nm, t_R (minor) = 55.7 min, t_R (major) = 77.3 min, ee = 74%.

Chiral HPLC spectrum of (*rac*)-**5b**

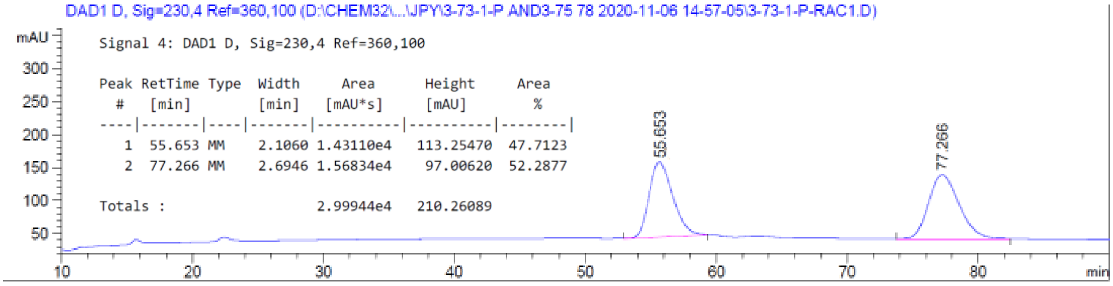

Chiral HPLC spectrum of (*S*)-**5b**

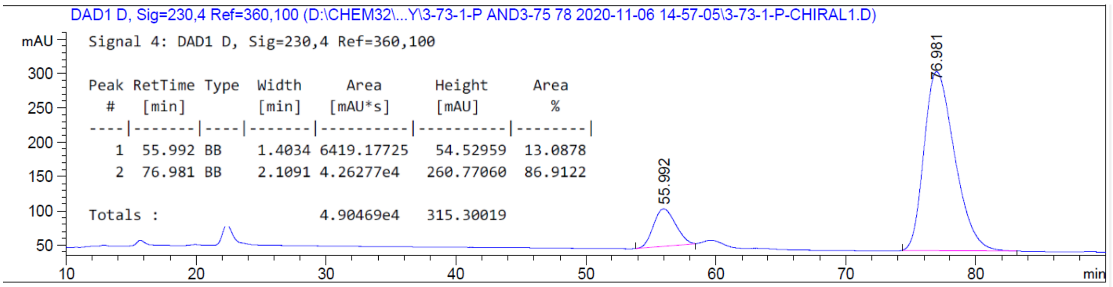

(3) Kinetic resolution of 4c with 4-bromocinnamaldehyde (4 days)

(*R*)-1-(2-hydroxy-6-methylnaphthalen-1-yl)isoquinoline 2-oxide ((*R*)-4c)

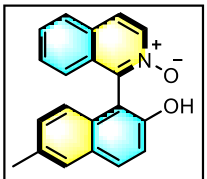

(*R*)-**4c**

(*R*)-4c was obtained in **44% yield** and **86% ee** as a white solid. **^{1}H NMR (400 MHz, DMSO-*d*$_6$)** δ 9.82 (s, 1H), 8.35 (d, *J* = 7.2 Hz, 1H), 8.04 (d, *J* = 7.2 Hz, 1H), 8.03 (d, *J* = 8.4 Hz, 1H), 7.89 (d, *J* = 8.9 Hz, 1H), 7.68 (s, 1H), 7.57 (ddd, *J* = 8.4, 6.8, 1.2 Hz, 1H), 7.45 (ddd, *J* = 8.4, 6.8, 1.2 Hz, 1H), 7.31 (d, *J* = 8.9 Hz, 1H), 7.10 (dd, *J* = 8.4, 1.8 Hz, 1H), 7.04 (d, *J* = 8.5 Hz, 1H), 6.75 (d, *J* = 8.5 Hz, 1H), 2.39 (s, 3H).

^{13}C NMR (100 MHz, DMSO-*d*$_6$) δ 153.1, 142.2, 137.5, 131.9, 130.8, 130.2, 129.8, 129.2, 128.9, 128.1, 128.1, 127.8, 127.2, 127.1, 124.7, 123.9, 123.4, 118.6, 110.7, 20.9. **HRMS (ESI)** calcd for [M+H] $C_{20}H_{16}NO_2^+$, m/z: 302.1176; found: 302.1173.

HPLC analysis: HPLC DAICEL CHIRALCEL IE, *n*-hexane/isopropanol = 75/25, 1.0 mL/min, λ = 230 nm, t_R (major) = 40.0 min, t_R (minor) = 51.3 min, ee = 86%.

Chiral HPLC spectrum of (*rac*)-**4c**

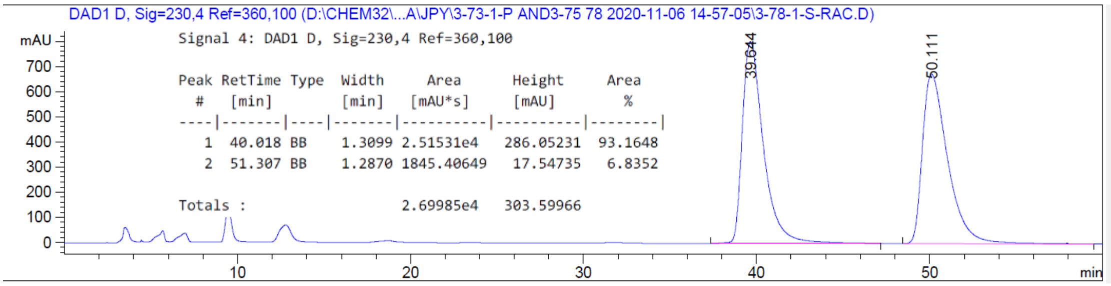

Chiral HPLC spectrum of (*R*)-**4c**

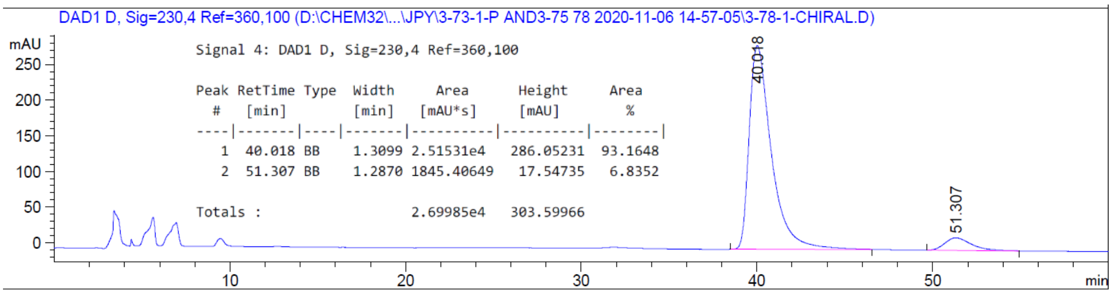

(S)-1-(2-((3-(4-bromophenyl)propanoyl)oxy)-6-methylnaphthalen-1-yl)isoquinoline 2-oxide (5c)

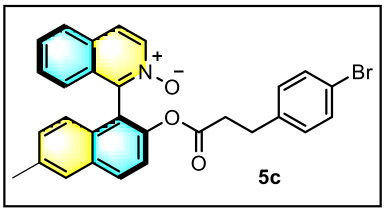

5c was obtained in **53% yield** and **76% ee** as a yellow solid. **^{1}H NMR (400 MHz, DMSO-d_6)** δ 8.38 (dd, J = 7.2, 2.1 Hz, 1H), 8.10-8.08 (m, 2H), 8.04 (d, J = 8.2 Hz, 1H), 7.86 (s, 1H), 7.58 (t, J = 7.4 Hz, 1H), 7.50 (d, J = 9.0 Hz, 1H), 7.43-7.40 (m, 1H), 7.32 (d, J = 8.0 Hz, 2H), 7.24 (d, J = 8.6 Hz, 1H), 6.96 (d, J = 8.6 Hz, 1H), 6.91-6.87 (m, 3H), 2.56-2.33 (m, 7H). **^{13}C NMR (100 MHz, DMSO-d_6)** δ 170.5, 146.9, 140.0, 139.6, 137.9, 135.9, 131.9, 131.5, 130.6, 130.4, 130.3, 130.1, 129.9, 129.8, 128.4, 128.2, 127.8, 127.7, 125.1, 125.0, 124.4, 122.7, 120.0, 119.6, 34.8, 29.5, 21.5. **HRMS (ESI)** calcd for [M+H] $C_{29}H_{23}BrNO_3^+$, m/z: 512.0856; found: 512.0850. **HPLC analysis:** HPLC DAICEL CHIRALCEL AD-H, *n*-hexane/isopropanol = 60/40, 1.0 mL/min, λ = 254 nm, t_R (major) = 20.1 min, t_R (minor) = 21.7 min, ee = 76%.

Chiral HPLC spectrum of (*rac*)-**5c**

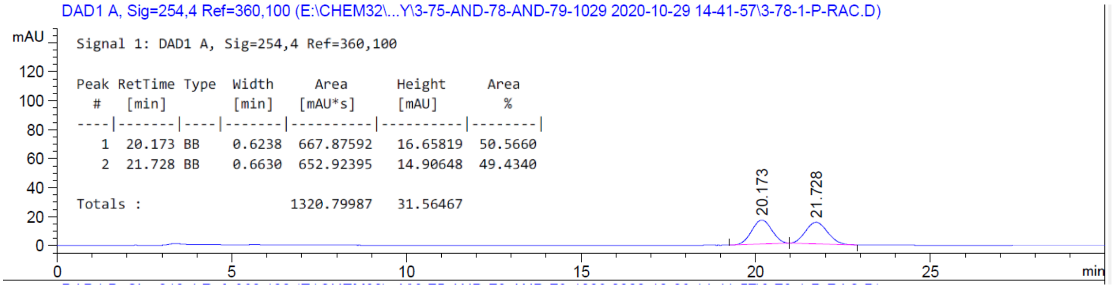

Chiral HPLC spectrum of (S)-**5c**

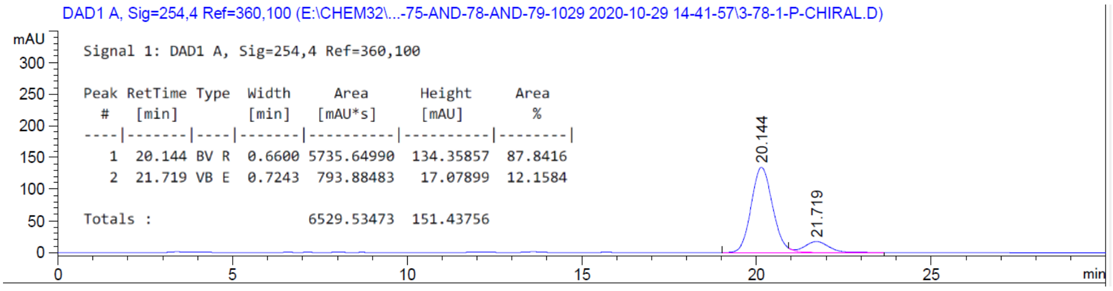

(4) Kinetic resolution of 4d with 4-bromocinnamaldehyde (3 days)

(*R*)-1-(2-hydroxy-6-phenylnaphthalen-1-yl)isoquinoline 2-oxide ((*R*)-4d)

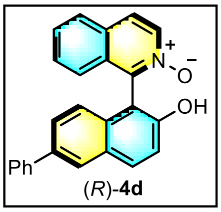

(*R*)-4d was obtained in **42% yield** and **94% ee** as a white solid. **^{1}H NMR (400 MHz, DMSO-*d*$_6$)** δ 10.03 (s, 1H), 8.37 (d, *J* = 7.2 Hz, 1H), 8.23 (d, *J* = 2.0 Hz, 1H), 8.10-8.04 (m, 3H), 7.75-7.72 (m, 2H), 7.63-7.57 (m, 2H), 7.51-7.45 (m, 3H), 7.39 (d, *J* = 8.8 Hz, 1H), 7.37-7.33 (m, 1H), 7.11 (d, *J* = 8.4 Hz, 1H), 6.93 (d, *J* = 8.8 Hz, 1H). **^{13}C NMR (100 MHz, DMSO-*d*$_6$)** δ 154.0, 141.9, 140.1, 137.6, 134.8, 132.0, 131.4, 129.8, 129.3, 129.0, 128.3, 128.1, 127.9, 127.3, 127.2, 126.7, 126.0, 125.8, 124.7, 124.2, 124.1, 119.1, 110.6. **HRMS (ESI)** calcd for [M+H] $C_{25}H_{18}NO_2^+$, m/z: 364.1332; found: 364.1328. **HPLC analysis:** HPLC DAICEL CHIRALCEL AD-H, *n*-hexane/isopropanol =75/25, 1.0 mL/min, λ = 254 nm, t_R (major) = 15.7 min, t_R (minor) = 21.8 min, ee = 94%.

Chiral HPLC spectrum of (*rac*)-**4d**

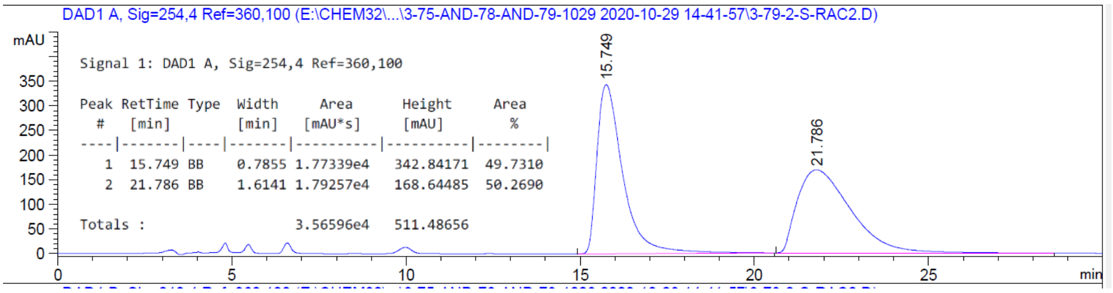

Chiral HPLC spectrum of (*R*)-**4d**

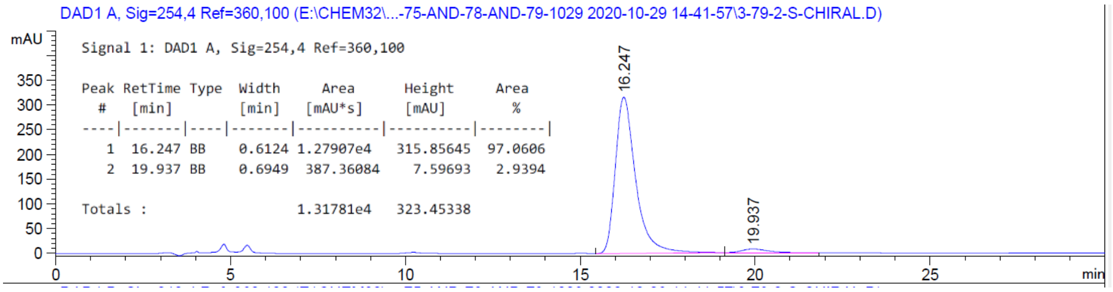

(*S*)-1-(2-((3-(4-bromophenyl)propanoyl)oxy)-6-phenylnaphthalen-1-yl)isoquinoline 2-oxide (5d)

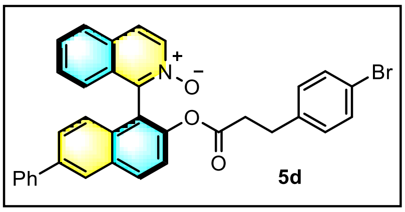

5d was obtained in **54% yield** and **72% ee** as a yellow solid. **^{1}H NMR (400 MHz, DMSO-*d*$_6$)** δ 8.42 (d, *J* = 4.5 Hz, 1H), 8.40 (s, 1H), 8.28 (d, *J* = 9.0 Hz, 1H), 8.12 (d, *J* = 7.2 Hz, 1H), 8.06 (d, *J* = 8.1 Hz, 1H), 7.79-7.77 (m, 2H), 7.74 (dd, *J* = 8.8, 2.0 Hz, 1H), 7.61-7.57 (m, 2H), 7.49 (t, *J* = 7.7 Hz, 2H), 7.46-7.37 (m, 2H), 7.33 (d, *J* = 8.4 Hz, 2H), 7.15 (d, *J* = 8.8 Hz, 1H), 6.98 (d, *J* = 8.6 Hz, 1H), 6.89 (d, *J* = 8.4 Hz, 2H), 2.51-2.34 (m, 4H). **^{13}C NMR (100 MHz, DMSO-*d*$_6$)** δ 170.5, 147.6, 140.0, 139.9, 139.6, 138.2, 138.0, 132.1, 131.6, 131.3, 130.6, 130.0, 129.8, 129.5, 129.5, 128.3, 128.2, 127.8, 127.5, 127.1, 126.4, 125.9, 125.2, 124.4, 123.2, 120.1, 119.7, 34.8, 29.5. **HRMS (ESI)** calcd for [M+H] $C_{34}H_{25}BrNO_3^+$, m/z: 574.1012; found: 574.1005. **HPLC analysis:** HPLC DAICEL CHIRALCEL OD-3, *n*-hexane/isopropanol =80/20, 0.6 mL/min, λ = 254 nm, t_R (major) = 73.3 min, t_R (minor) = 87.8 min, ee = 72%.

Chiral HPLC spectrum of (*rac*)-**5d**

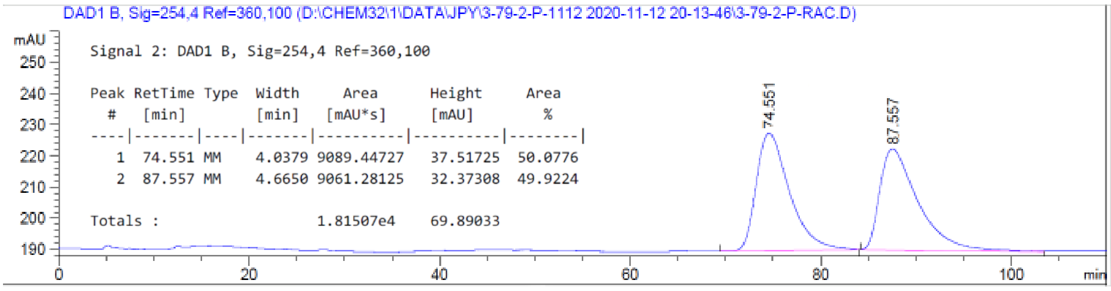

Chiral HPLC spectrum of (*S*)-**5d**

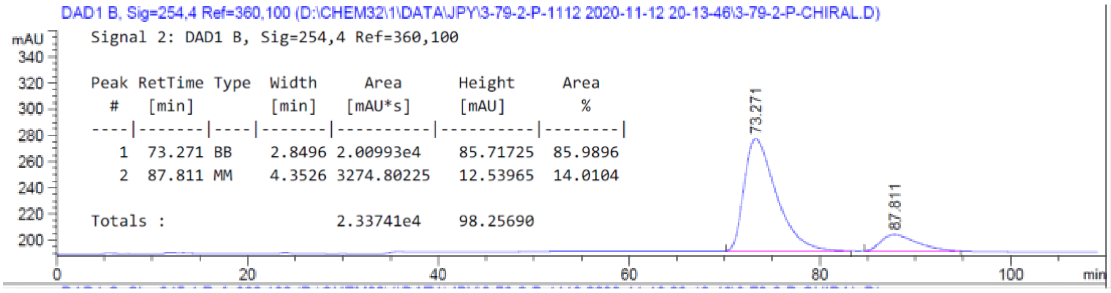

(5) Kinetic resolution of 4e with 4-bromocinnamaldehyde (4 days)

(*R*)-1-(2-hydroxy-7-methoxynaphthalen-1-yl)isoquinoline 2-oxide ((*R*)-4e)

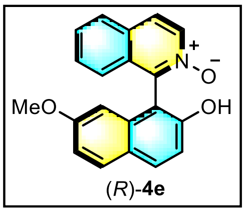

(*R*)-**4e** was obtained in **42% yield** and **91% ee** as a white solid. **^{1}H NMR (400 MHz, DMSO-*d*$_6$)** δ 9.69 (s, 1H), 8.35 (d, *J* = 7.2 Hz, 1H), 8.03 (d, *J* = 7.2, 1H), 8.02 (d, *J* = 8.4, 1H), 7.90 (d, *J* = 8.8 Hz, 1H), 7.59-7.55 (m, 1H), 7.49-7.44 (m, 1H), 7.36 (d, *J* = 2.8 Hz, 1H), 7.32 (d, *J* = 8.8 Hz, 1H), 7.05 (d, *J* = 8.4 Hz, 1H), 6.96 (dd, *J* = 9.2, 2.8 Hz, 1H), 6.76 (d, *J* = 9.2 Hz, 1H), 3.83 (s, 3H). **^{13}C NMR (100 MHz, DMSO-*d*$_6$)** δ 155.3, 152.0, 142.1, 137.5, 129.8, 129.7, 129.2, 128.9, 128.1, 127.8, 127.8, 127.2, 125.0, 124.7, 124.0, 119.2, 119.0, 111.0, 106.9, 55.2. **HRMS (ESI)** calcd for [M+H] $C_{20}H_{16}NO_3^+$, m/z: 318.1125; found: 318.1121. **HPLC analysis:** HPLC DAICEL CHIRALCEL AD-H, *n*-hexane/isopropanol =75/25, 1.0 mL/min, λ = 230 nm, t_R (major) = 13.0 min, t_R (minor) = 23.0 min, ee = 91%.

Chiral HPLC spectrum of (*rac*)-**4e**

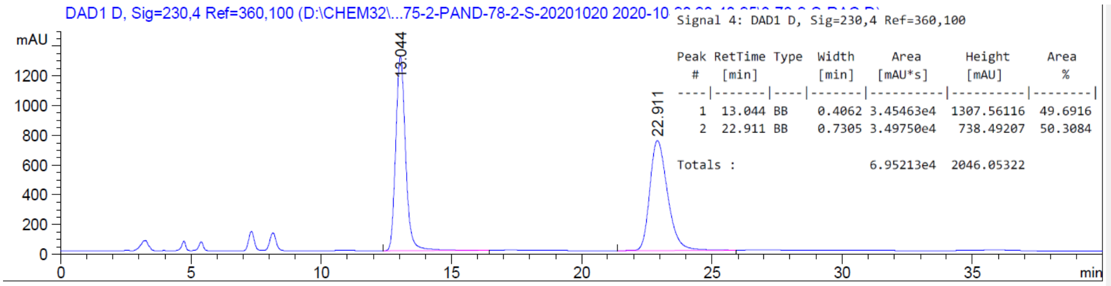

Chiral HPLC spectrum of (*R*)-**4e**

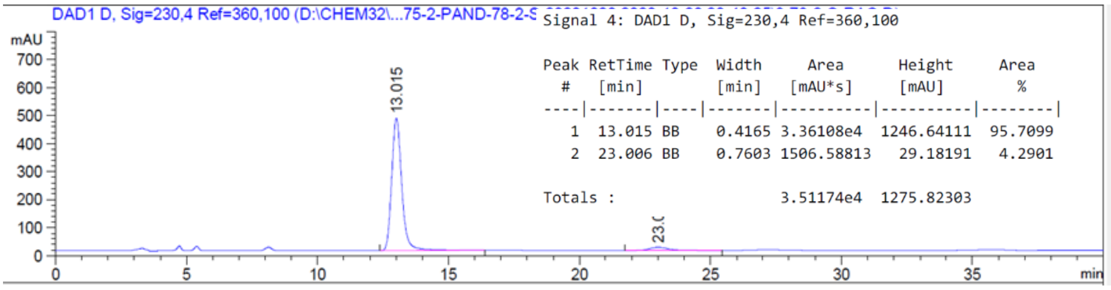

(*S*)-1-(2-((3-(4-bromophenyl)propanoyl)oxy)-7-methoxynaphthalen-1-yl)isoquinoline 2-oxide (5e)

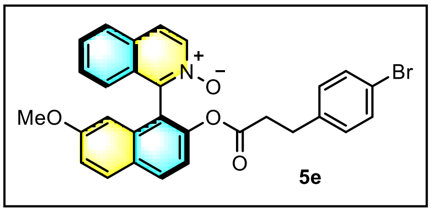

5e was obtained in **54% yield** and **73% ee** as a yellow solid. **^{1}H NMR (400 MHz, DMSO-*d*$_6$)** δ 8.38 (d, *J* = 7.2 Hz, 1H), 8.09 (d, *J* = 8.8 Hz, 1H), 8.08 (d, *J* = 7.2 Hz, 1H), 8.04 (d, *J* = 8.4 Hz, 1H), 7.60-7.56 (m, 1H), 7.53 (d, *J* = 2.6 Hz, 1H), 7.49 (d, *J* = 9.0 Hz, 1H), 7.44-7.40 (m, 1H), 7.32 (d, *J* = 8.4 Hz, 2H), 7.08 (dd, *J* = 9.0, 2.6 Hz, 1H), 6.96 (d, *J* = 9.2 Hz, 1H), 6.91 (d, *J* = 8.4 Hz, 1H), 6.89 (d, *J* = 8.4 Hz, 2H), 3.89 (s, 3H), 2.55-2.35 (m, 4H). **^{13}C NMR (100 MHz, DMSO-*d*$_6$)** δ 170.1, 157.3, 145.2, 139.5, 139.2, 137.5, 132.6, 131.1, 130.1, 129.4, 129.4, 129.3, 128.0, 127.8, 127.3, 126.8, 126.2, 124.6, 124.0, 122.6, 119.9, 119.7, 119.2, 106.9, 55.3, 34.3, 29.0. **HRMS (ESI)** calcd for [M+H] $C_{29}H_{23}BrNO_4^+$, m/z: 528.0805; found: 528.0802. **HPLC analysis:** HPLC DAICEL CHIRALCEL AD-H, *n*-hexane/isopropanol =60/40, 1.0 mL/min, λ = 254 nm, t_R (major) = 24.2 min, t_R (minor) = 28.9 min, ee = 73%.

Chiral HPLC spectrum of (*rac*)-**5e**

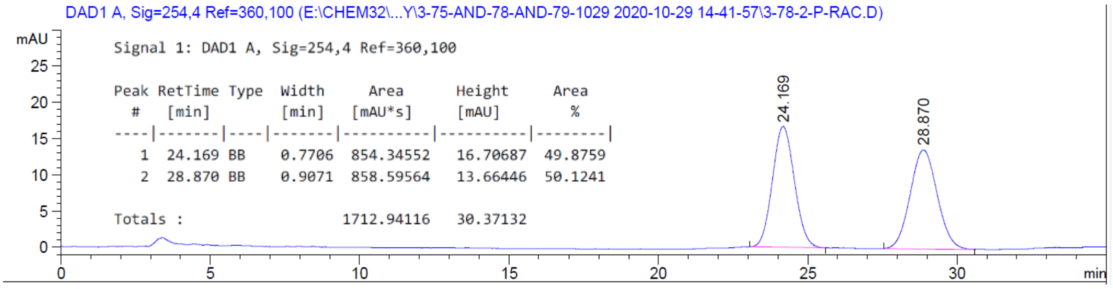

Chiral HPLC spectrum of (*S*)-**5e**

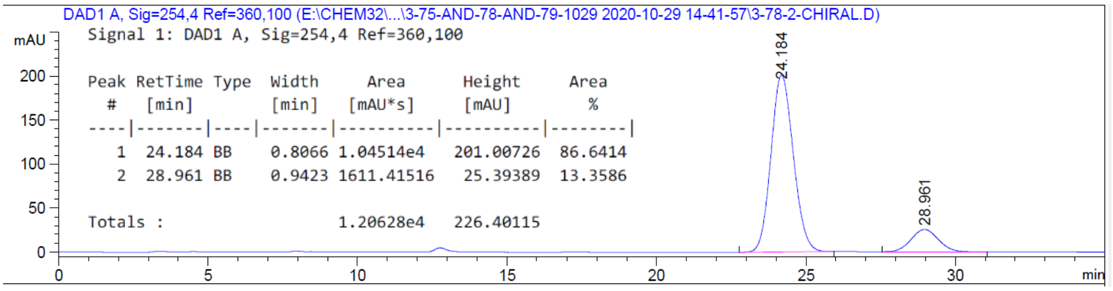

(6) Kinetic resolution of 4f with 4-bromocinnamaldehyde (7 days)

(*R*)-1-(2-hydroxynaphthalen-1-yl)-6-methylisoquinoline 2-oxide ((*R*)-4f)

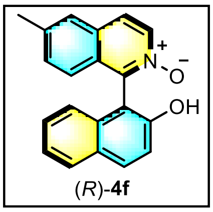

(*R*)-**4f** was obtained in **44% yield** and **85% ee** as a white solid. **^{1}H NMR (400 MHz, DMSO-*d*$_6$)** δ 9.97 (s, 1H), 8.32 (d, *J* = 6.4 Hz, 1H), 7.99-7.90 (m, 3H), 7.81 (s, 1H), 7.36-7.23 (m, 4H), 6.96 (d, *J* = 8.4 Hz, 1H), 6.82 (d, *J* = 8.0 Hz, 1H), 2.43 (s, 3H). **^{13}C NMR (100 MHz, DMSO-*d*$_6$)** δ 153.8, 141.9, 137.8, 137.5, 132.7, 131.3, 130.8, 128.4, 128.2, 128.0, 127.9, 126.8, 126.2, 124.7, 123.4, 123.4, 122.9, 118.7, 110.8, 21.1. **HRMS (ESI)** calcd for [M+H] $C_{20}H_{16}NO_2^+$, m/z: 302.1176; found: 302.1174. **HPLC analysis:** HPLC DAICEL CHIRALCEL IE, *n*-hexane/isopropanol = 75/25, 1.0 mL/min, λ = 254 nm, t_R (major) = 38.1 min, t_R (minor) = 48.1 min, ee = 85%.

Chiral HPLC spectrum of (*rac*)-**4f**

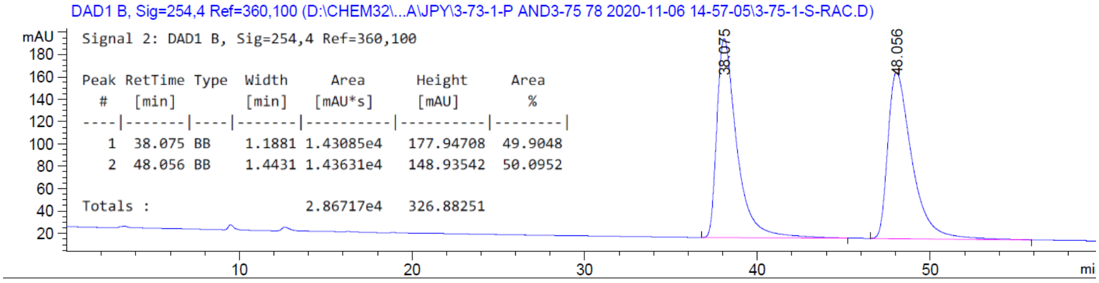

Chiral HPLC spectrum of (*R*)-**4f**

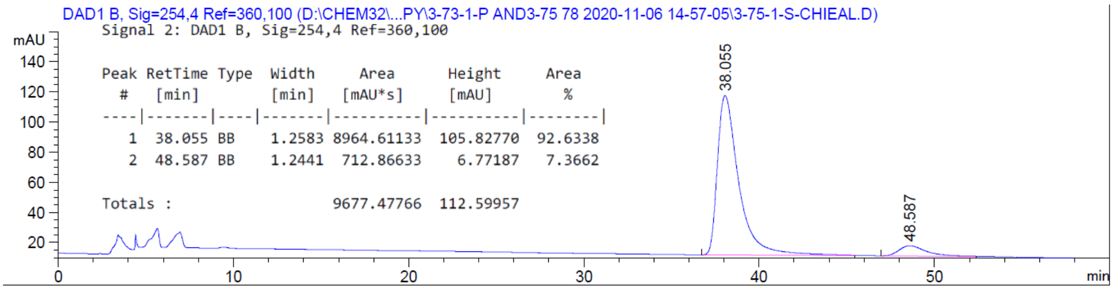

(*S*)-1-(2-((3-(4-bromophenyl)propanoyl)oxy)naphthalen-1-yl)-6-methylisoquinoline 2-oxide (5f)

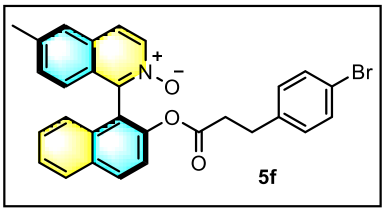

5f was obtained in **51% yield** and **77% ee** as a yellow solid.

^{1}H NMR (400 MHz, DMSO-d_6) δ 8.34 (d, *J* = 7.2 Hz, 1H), 8.20 (d, *J* = 8.8 Hz, 1H), 8.10 (d, *J* = 8.0 Hz, 1H), 7.99 (d, *J* = 7.2 Hz, 1H), 7.82 (s, 1H), 7.57-7.53 (m, 2H), 7.42-7.38 (m, 1H), 7.31 (dd, *J* = 8.4, 2.0 Hz, 2H), 7.27 (dd, *J* = 8.8, 2.0 Hz, 1H), 7.03 (d, *J* = 8.4 Hz, 1H), 6.89 (dd, *J* = 8.4, 2.0 Hz, 2H), 6.81 (d, *J* = 8.8 Hz, 1H), 2.56-2.38 (m, 7H). **^{13}C NMR (100 MHz, DMSO-d_6)** δ 170.0, 147.1, 139.3, 139.2, 138.0, 137.4, 131.7, 131.5, 131.2, 131.0, 130.6, 130.1, 128.5, 128.0, 127.6, 127.4, 126.3, 126.1, 124.7, 124.0, 123.9, 122.3, 119.8, 119.1, 34.3, 29.0, 21.1. **HRMS (ESI)** calcd for [M+H] $C_{29}H_{23}BrNO_3^+$, m/z: 512.0856; found: 512.0851.

HPLC analysis: HPLC DAICEL CHIRALCEL AD-H, *n*-hexane/isopropanol = 60/40, 1.0 mL/min, λ = 230 nm, t_R (major) = 12.4 min, t_R (minor) = 25.9 min, ee = 77%.

Chiral HPLC spectrum of (*rac*)-**5f**

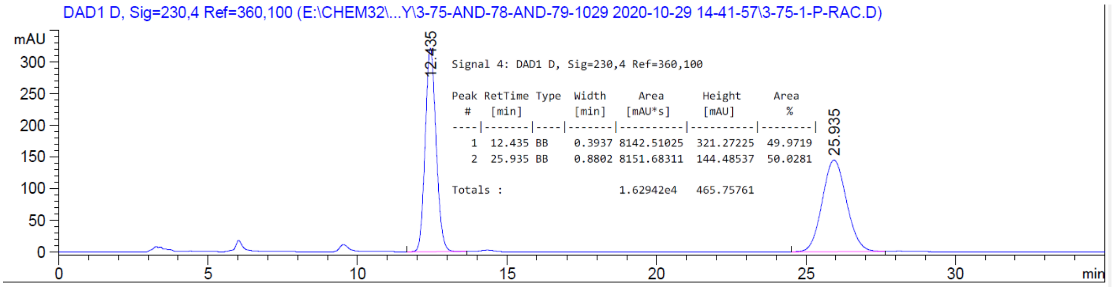

Chiral HPLC spectrum of (*S*)-**5f**

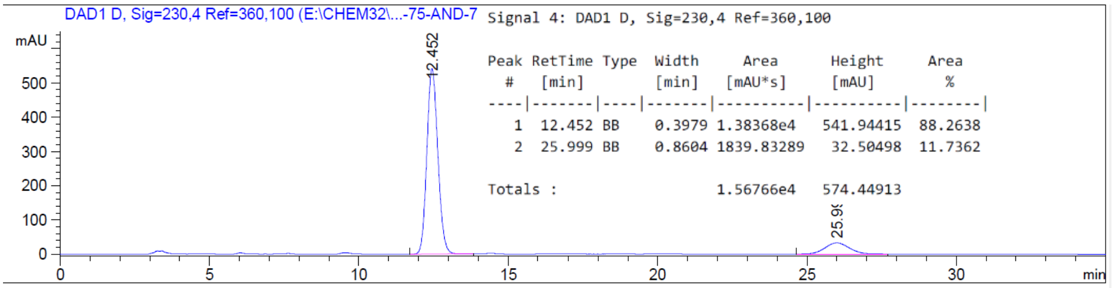

(7) Kinetic resolution of 4g with 4-bromocinnamaldehyde (3 days)

(*R*)-7-bromo-1-(2-hydroxynaphthalen-1-yl)isoquinoline 2-oxide ((*R*)-4g)

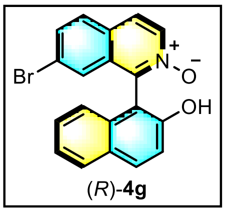

(*R*)-**4g** was obtained in **43% yield** and **89% ee** as a white solid. **^{1}H NMR (400 MHz, DMSO-*d*$_6$)** δ 10.03 (s, 1H), 8.40 (d, *J* = 7.2 Hz, 1H), 8.08 (d, *J* = 7.2 Hz, 1H), 8.02 (d, *J* = 8.4 Hz, 1H), 8.01 (d, *J* = 8.4 Hz, 1H), 7.92 (dd, *J* = 8.0, 1.6 Hz, 1H), 7.72 (dd, *J* = 8.8, 2.0 Hz, 1H), 7.36 (d, *J* = 9.2 Hz, 1H), 7.33-7.27 (m, 2H), 7.13 (d, *J* = 2.0 Hz, 1H), 6.86 (dd, *J* = 8.0, 2.0 Hz, 1H). **^{13}C NMR (100 MHz, DMSO-*d*$_6$)** δ 153.7, 141.1, 138.3, 132.4, 131.3, 131.1, 130.6, 129.7, 128.4, 127.9, 127.1, 126.7, 125.9, 124.0, 123.2, 123.1, 122.7, 118.6, 109.9. **HRMS (ESI)** calcd for [M+H] $C_{19}H_{13}BrNO_2^+$, m/z: 366.0124; found: 366.0121. **HPLC analysis:** HPLC DAICEL CHIRALCEL AD-H, *n*-hexane/isopropanol =75/25, 1.0 mL/min, λ = 254 nm, t_R (major) = 6.9 min, t_R (minor) = 11.0 min, ee = 89%.

Chiral HPLC spectrum of (*rac*)-**4g**

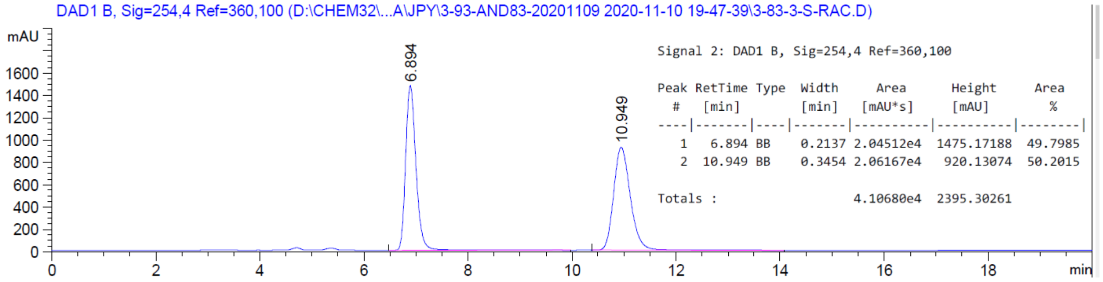

Chiral HPLC spectrum of (*R*)-**4g**

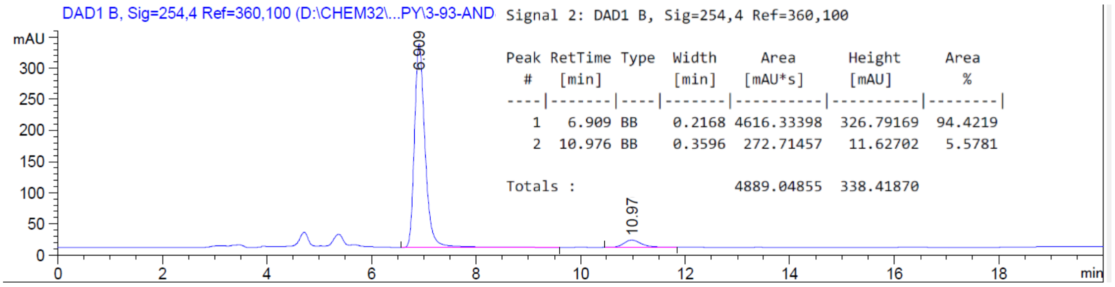

(*S*)-7-bromo-1-(2-((3-(4-bromophenyl)propanoyl)oxy)naphthalen-1-yl)isoquinoline 2-oxide (5g)

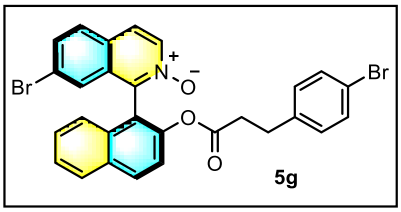

5g was obtained in **53% yield** and **75% ee** as a yellow solid. **^{1}H NMR (400 MHz, DMSO-*d*$_6$)** δ 8.43 (d, *J* = 7.2 Hz, 1H), 8.23 (d, *J* = 8.8 Hz, 1H), 8.14-8.10 (m, 2H), 8.03 (d, *J* = 8.8 Hz, 1H), 7.72 (dd, *J* = 8.4, 2.0 Hz, 1H), 7.60-7.54 (m, 2H), 7.46-7.42 (m, 1H), 7.33 (dd, *J* = 7.2, 2.0 Hz, 2H), 7.09 (d, *J* = 8.4 Hz, 1H), 7.01 (d, *J* = 1.8 Hz, 1H), 6.91 (dd, *J* = 7.2, 2.0 Hz, 2H), 2.54-2.37 (m, 4H). **^{13}C NMR (100 MHz, DMSO-*d*$_6$)** δ 170.2, 147.2, 139.1, 138.6, 138.1, 131.4, 131.2, 131.1, 131.1, 130.9, 130.6, 130.1, 129.6, 128.6, 127.7, 126.4, 126.3, 125.4, 124.7, 124.5, 123.0, 122.3, 119.2, 119.0, 34.2, 29.0. **HRMS (ESI)** calcd for [M+H] $C_{28}H_{20}Br_2NO_3^+$, m/z: 575.9804; found: 575.9799. **HPLC analysis:** HPLC DAICEL CHIRALCEL AD-H, *n*-hexane/isopropanol =60/40, 1.0 mL/min, λ = 254 nm, t_R (major) = 10.3 min, t_R (minor) = 20.9 min, ee = 75%.

Chiral HPLC spectrum of (*rac*)-**5g**

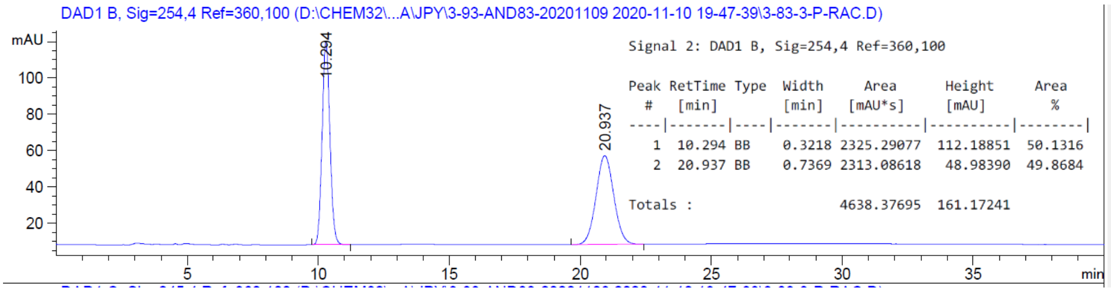

Chiral HPLC spectrum of (*S*)-**5g**

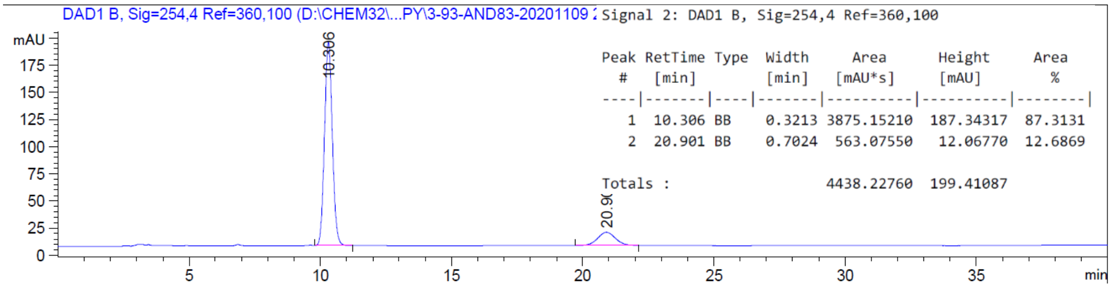

(8) Kinetic resolution of 4h with 4-bromocinnamaldehyde (4 days)

(*R*)-7-bromo-1-(2-hydroxy-6-phenylnaphthalen-1-yl)isoquinoline 2-oxide ((*R*)-4h)

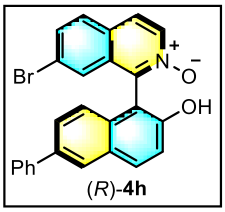

(*R*)-**4h** was obtained in **40% yield** and > **99% ee** as a white solid. **^{1}H NMR (400 MHz, DMSO-d_6)** δ 10.12 (s, 1H), 8.42 (d, *J* = 7.2 Hz, 1H), 8.23 (s, 1H), 8.11-8.09 (m, 2H), 8.03 (d, *J* = 8.4 Hz, 1H), 7.75-7.71 (m, 3H), 7.64 (d, *J* = 8.4 Hz, 1H), 7.49-7.45 (m, 2H), 7.41 (d, *J* = 8.8 Hz, 1H), 7.35 (t, *J* = 7.2 Hz, 1H), 7.20 (d, *J* = 2.0 Hz, 1H), 6.96 (d, *J* = 8.8 Hz, 1H). **^{13}C NMR (100 MHz, DMSO-d_6)** δ 154.4, 141.5, 140.5, 138.8, 135.4, 132.2, 132.2, 131.6, 131.1, 130.2, 129.2, 129.4 128.7, 127.7, 127.2, 126.7, 126.4, 124.5, 124.5, 123.3, 119.5, 110.3. **HRMS (ESI)** calcd for [M+H] $C_{25}H_{17}BrNO_2^+$, m/z: 442.0437; found: 442.0432. **HPLC analysis:** HPLC DAICEL CHIRALCEL IE, *n*-hexane/isopropanol = 75/25, 1.0 mL/min, λ = 280 nm, t_R (major) = 33.1 min, t_R (minor) = 41.9 min, ee > 99%.

Chiral HPLC spectrum of (*rac*)-**4h**

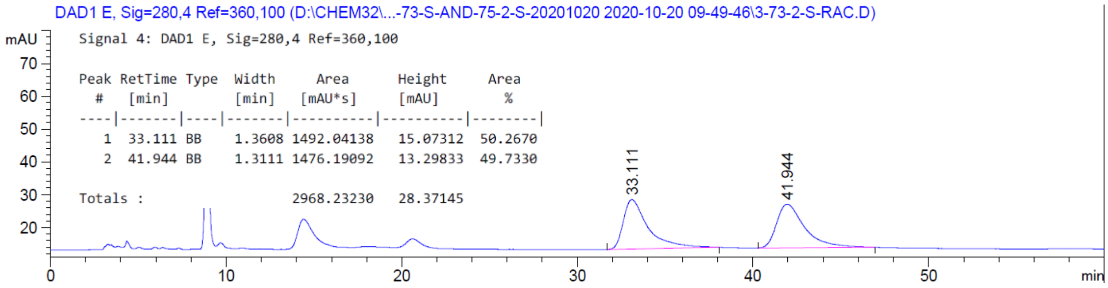

Chiral HPLC spectrum of (*R*)-**4h**

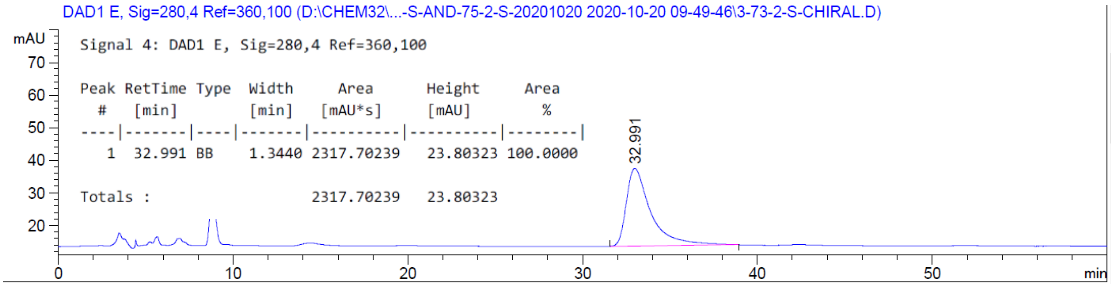

(*S*)-7-bromo-1-(2-((3-(4-bromophenyl)propanoyl)oxy)-6-phenylnaphthalen-1-yl)isoquinoline 2-oxide (5h)

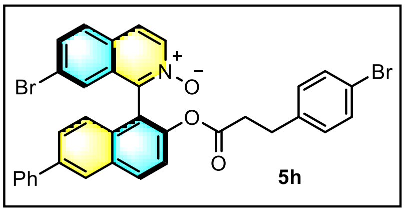

5h was obtained in **58% yield** and **66% ee** as a yellow solid. **^{1}H NMR (400 MHz, DMSO-*d*$_6$)** δ 8.46 (d, *J* = 7.2 Hz, 1H), 8.43 (d, *J* = 1.6 Hz, 1H), 8.32 (d, *J* = 8.8 Hz, 1H), 8.16 (dd, *J* = 7.2, 0.8 Hz, 1H), 8.05 (d, *J* = 8.8 Hz, 1H), 7.81-7.73 (m, 4H), 7.59 (d, *J* = 8.8 Hz, 1H), 7.51 (t, *J* = 7.6 Hz, 2H), 7.43-7.39 (m, 1H), 7.34 (dd, *J* = 6.8, 2.0 Hz, 2H), 7.18 (d, *J* = 8.8 Hz, 1H), 7.08 (d, *J* = 2.0 Hz, 1H), 6.92 (dd, *J* = 6.8, 2.0 Hz, 2H), 2.62-2.43 (m, 4H). **^{13}C NMR (100 MHz, DMSO-*d*$_6$)** δ 170.2, 147.2, 139.4, 139.1, 138.5, 138.2, 137.9, 131.6, 131.5, 131.1, 130.9, 130.6, 130.6, 130.1, 129.6, 129.1, 127.8, 127.0, 126.9, 126.4, 126.1, 125.4, 125.3, 124.7, 123.1, 122.8, 119.2, 119.0, 34.2, 29.0. **HRMS (ESI)** calcd for [M+H] $C_{34}H_{24}Br_2NO_3^+$, m/z: 652.0117; found: 652.0114. **HPLC analysis:** HPLC DAICEL CHIRALCEL AD-H, *n*-hexane/isopropanol = 60/40, 1.0 mL/min, λ = 254 nm, t_R (major) = 16.6 min, t_R (minor) = 24.3 min, ee = 66%.

Chiral HPLC spectrum of (*rac*)-**5h**

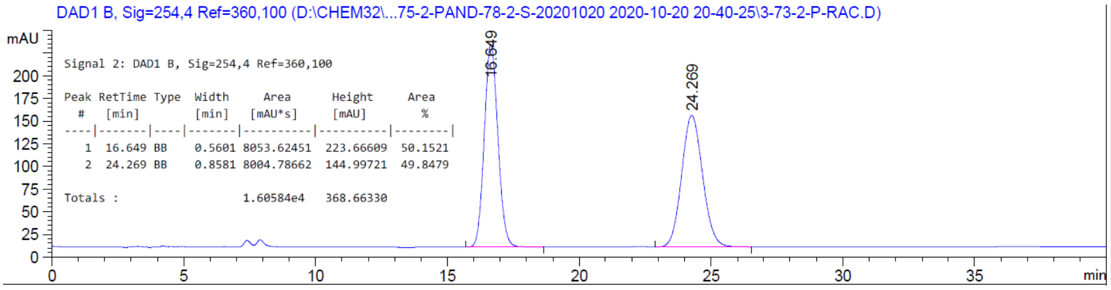

Chiral HPLC spectrum of (*S*)-**5h**

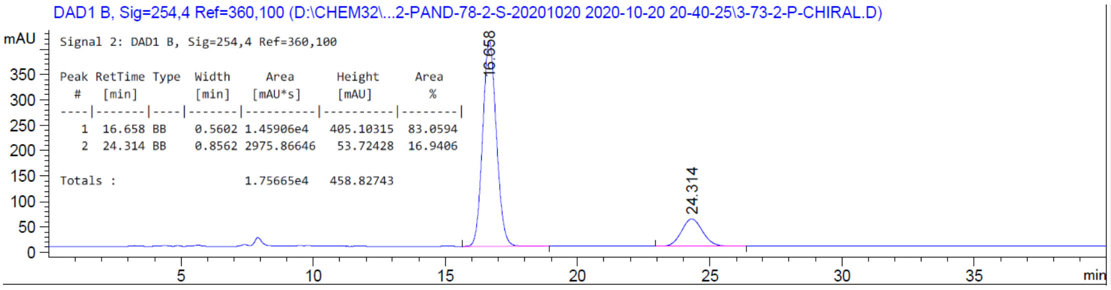

With 0.5 equiv cinnamic aldehyde to synthesis products 5 with high ee value

(9) Kinetic resolution of 4a with 0.5 eqiv 4-bromocinnamaldehyde (3 days)

(*S*)-1-(2-((3-(4-bromophenyl)propanoyl)oxy)naphthalen-1-yl)isoquinoline 2-oxide (5a)

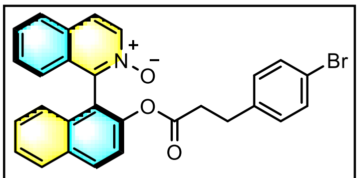

5a

5a was obtained in **45% yield** and **93% ee** as a yellow solid. **HPLC analysis:** HPLC DAICEL CHIRALCEL AD-H, *n*-hexane/isopropanol = 60/40, 1.0 mL/min, λ = 254 nm, t_R (major) = 13.4 min, t_R (minor) = 29.8 min, ee = 93%.

Chiral HPLC spectrum of (*rac*)-**5a**

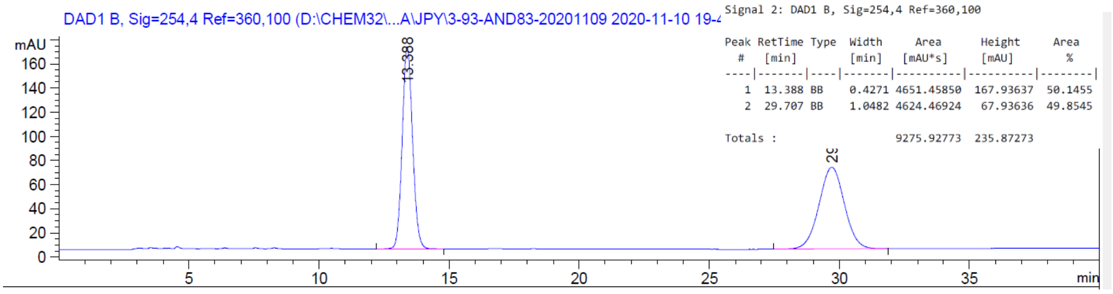

Signal 2: DAD1 B, Sig=254,4 Ref=360,100

Peak #	RetTime [min]	Type	Width [min]	Area [mAU*s]	Height [mAU]	Area %
1	13.388	BB	0.4271	4651.45850	167.93637	50.1455
2	29.707	BB	1.0482	4624.46924	67.93636	49.8545
Totals :				9275.92773	235.87273	

Chiral HPLC spectrum of (*S*)-**5a**

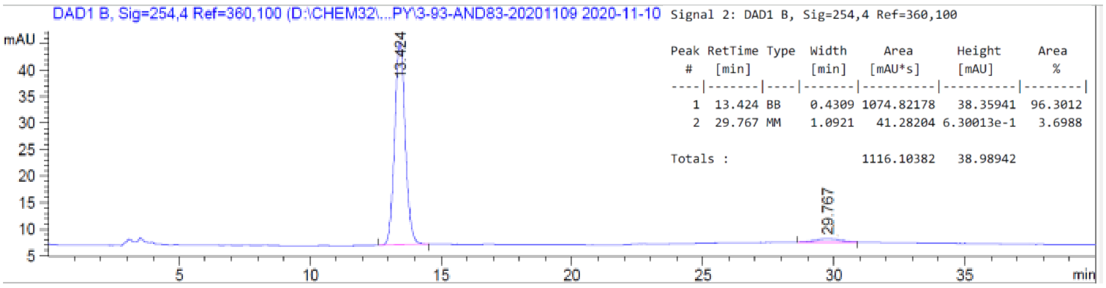

Signal 2: DAD1 B, Sig=254,4 Ref=360,100

Peak #	RetTime [min]	Type	Width [min]	Area [mAU*s]	Height [mAU]	Area %
1	13.424	BB	0.4309	1074.82178	38.35941	96.3012
2	29.767	MM	1.0921	41.28204	6.30013e-1	3.6988
Totals :				1116.10382	38.98942	

(*R*)-1-(2-hydroxynaphthalen-1-yl)isoquinoline 2-oxide ((*R*)-4a)

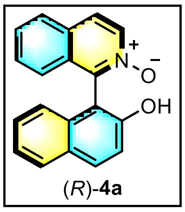

(*R*)-**4a** was obtained in **51% yield** and **79% ee** as a white solid.

HPLC analysis: HPLC DAICEL CHIRALCEL AD-H, *n*-hexane/isopropanol = 75/25, 1.0 mL/min, λ = 254 nm, t_R (major) = 9.9 min, t_R (minor) = 17.7 min, ee = 79%.

Chiral HPLC spectrum of (*rac*)-**4a**

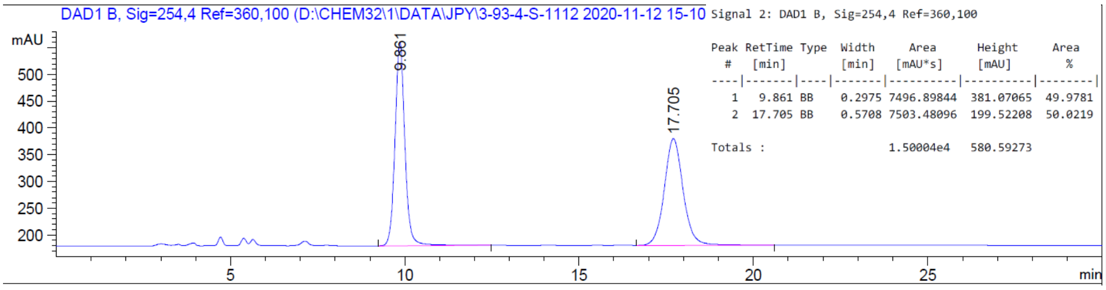

Signal 2: DAD1 B, Sig=254,4 Ref=360,100

Peak #	RetTime [min]	Type	Width [min]	Area [mAU*s]	Height [mAU]	Area %
1	9.861	BB	0.2975	7496.89844	381.07065	49.9781
2	17.705	BB	0.5708	7503.48096	199.52208	50.0219
Totals :				1.50004e4	580.59273	

Chiral HPLC spectrum of (*R*)-**4a**

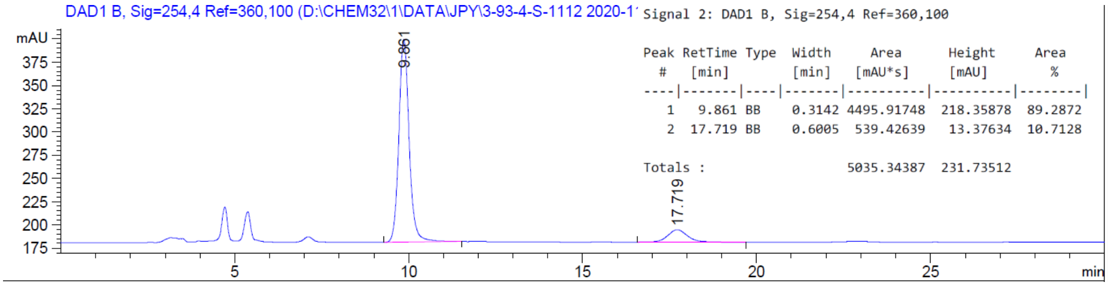

Signal 2: DAD1 B, Sig=254,4 Ref=360,100

Peak #	RetTime [min]	Type	Width [min]	Area [mAU*s]	Height [mAU]	Area %
1	9.861	BB	0.3142	4495.91748	218.35878	89.2872
2	17.719	BB	0.6005	539.42639	13.37634	10.7128
Totals :				5035.34387	231.73512	

(10) Kinetic resolution of 4a with 0.5 eqiv 4- fluorocinnamaldehyde (3 days)

(*S*)-1-(2-((3-(4-fluorophenyl)propanoyl)oxy)naphthalen-1-yl)isoquinoline 2-oxide (5i)

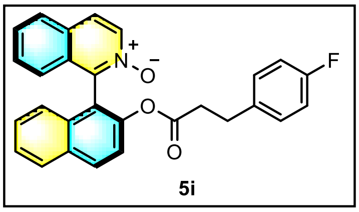

5i was obtained in **40% yield** and **89% ee** as a yellow solid. **^{1}H NMR (400 MHz, DMSO-*d*$_6$)** δ 8.40 (d, *J* = 7.2 Hz, 1H), 8.22 (d, *J* = 9.2 Hz, 1H), 8.11 (d, *J* = 7.2 Hz, 1H), 8.10 (d, *J* = 8.4 Hz, 1H), 8.06 (d, *J* = 8.4 Hz, 1H), 7.61-7.55 (m, 2H), 7.55 (d, *J* = 9.2 Hz, 1H), 7.46-7.39 (m, 2H), 7.07 (d, *J* = 8.8 Hz, 1H), 7.00-6.96 (m, 4H), 6.92 (d, *J* = 8.4 Hz, 1H), 2.56-2.37 (m, 4H). **^{13}C NMR (100 MHz, DMSO-*d*$_6$)** δ 170.1, 160.7 (d, *J* = 240.1 Hz), 147.1, 139.4, 137.5, 135.8 (d, *J* = 3.0 Hz), 131.6, 131.2, 130.7, 129.7, 129.6, 129.5, 129.3, 128.5, 128.0, 127.8, 127.5, 127.3, 126.1, 124.7, 123.9, 122.3, 119.7, 115.0, 114.8, 34.8, 28.9. ^{19}F NMR (**376 MHz, DMSO**) δ -116.95. **HRMS (ESI)** calcd for [M+H] $C_{28}H_{21}FNO_3^{+}$, m/z: 438.1500; found: 438.1496. **HPLC analysis:** HPLC DAICEL CHIRALCEL AD-H, *n*-hexane/isopropanol = 60/40, 1.0 mL/min, λ = 254 nm, t_R (major) = 12.6 min, t_R (minor) = 28.0 min, ee = 89%.

Chiral HPLC spectrum of (*rac*)-**5i**

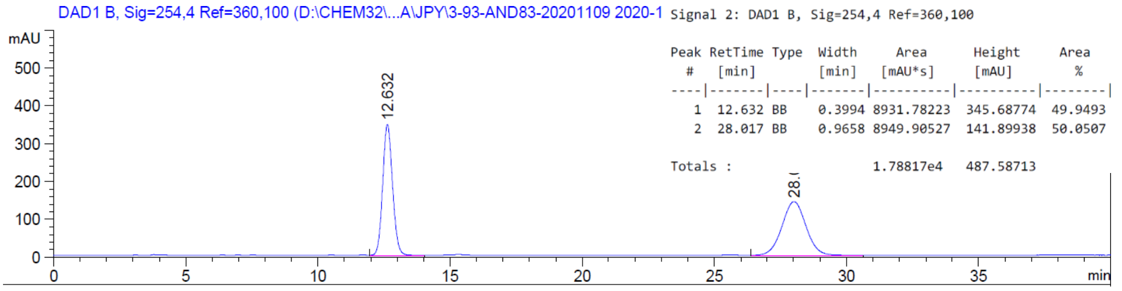

Chiral HPLC spectrum of (*S*)-**5i**

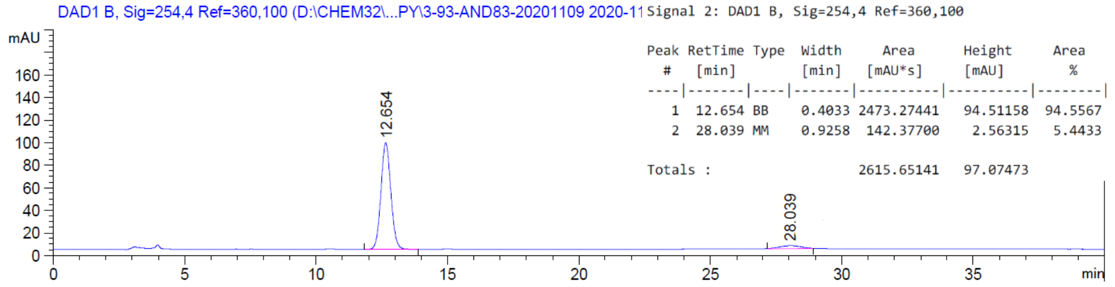

(*R*)-1-(2-hydroxynaphthalen-1-yl)isoquinoline 2-oxide ((*R*)-4a)

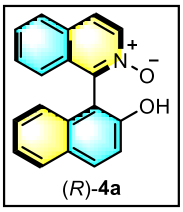

(*R*)-**4a** was obtained in **57% yield** and **59% ee** as a white solid. **HPLC analysis:** HPLC DAICEL CHIRALCEL AD-H, *n*-hexane/isopropanol = 75/25, 1.0 mL/min, λ = 254 nm, t_R (major) = 9.9 min, t_R (minor) = 17.9 min, ee = 59%.

Chiral HPLC spectrum of (*rac*)-**4a**

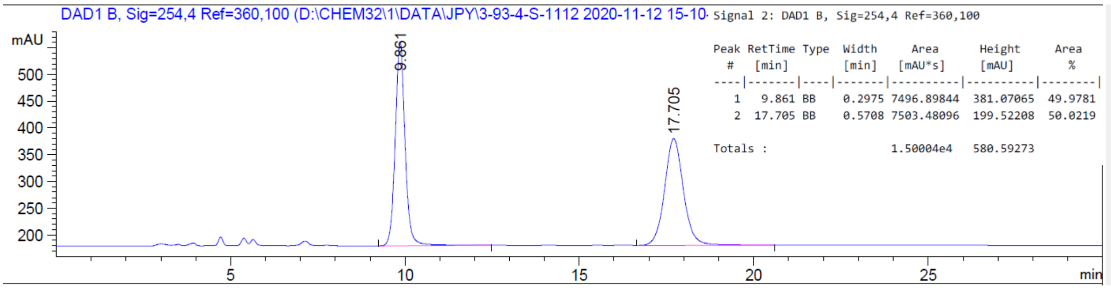

Chiral HPLC spectrum of (*R*)-**4a**

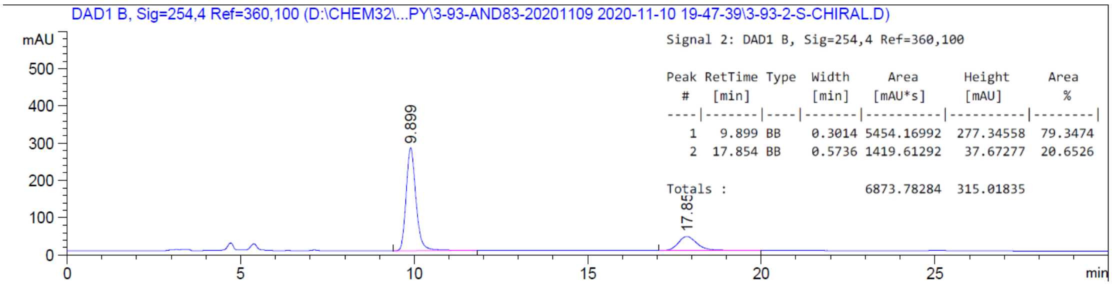

(11) Kinetic resolution of 4a with 0.5 eqiv cinnamaldehyde (3 days)

(*S*)-1-(2-((3-phenylpropanoyl)oxy)naphthalen-1-yl)isoquinoline 2-oxide (5j)

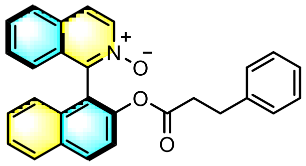

5j

5j was obtained in **40% yield** and **88% ee** as a yellow solid. **^{1}H NMR (400 MHz, DMSO-*d*$_6$)** δ 8.41 (d, *J* = 7.2, 1H), 8.22 (d, *J* = 9.0 Hz, 1H), 8.12 (d, *J* = 7.2 Hz, 1H), 8.11 (d, *J* = 8.0 Hz, 1H), 8.07 (d, *J* = 8.0 Hz, 1H), 7.62-7.54 (m, 3H), 7.47-7.40 (m, 2H), 7.20-7.13 (m, 3H), 7.09 (d, *J* = 8.4 Hz, 1H), 6.96-6.92 (m, 3H), 2.49-2.34 (m, 4H). **^{13}C NMR (100 MHz, DMSO-*d*$_6$)** δ 170.1, 147.1, 139.7, 139.5, 137.5, 131.6, 131.2, 130.7, 129.5, 129.4, 128.5, 128.3, 128.1, 127.8, 127.8, 127.8, 127.5, 127.3, 126.1, 124.7, 123.9, 122.3, 119.7, 34.8, 29.7. **HRMS (ESI)** calcd for [M+H] $C_{28}H_{22}NO_3^+$, m/z: 420.1594; found: 420.1588. **HPLC analysis:** HPLC DAICEL CHIRALCEL AD-H, *n*-hexane/isopropanol = 60/40, 1.0 mL/min, λ = 254 nm, t_R (major) = 11.7 min, t_R (minor) = 27.9 min, ee = 88%.

Chiral HPLC spectrum of (*rac*)-**5j**

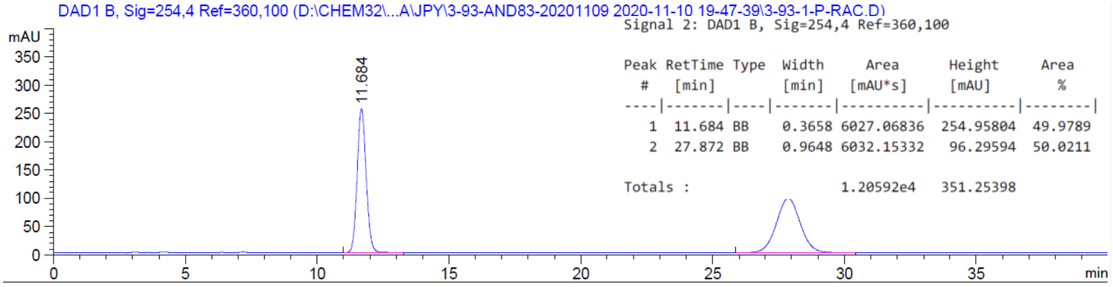

Signal 2: DAD1 B, Sig=254,4 Ref=360,100

Peak #	RetTime [min]	Type	Width [min]	Area [mAU*s]	Height [mAU]	Area %
1	11.684	BB	0.3658	6027.06836	254.95804	49.9789
2	27.872	BB	0.9648	6032.15332	96.29594	50.0211
Totals :				1.20592e4	351.25398	

Chiral HPLC spectrum of (*S*)-**5j**

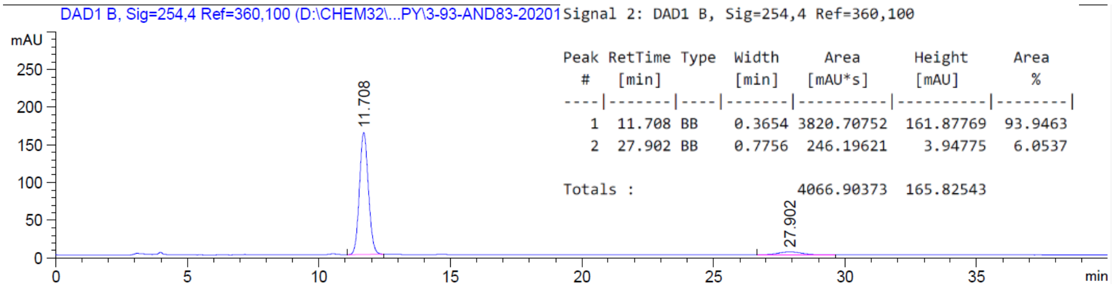

Signal 2: DAD1 B, Sig=254,4 Ref=360,100

Peak #	RetTime [min]	Type	Width [min]	Area [mAU*s]	Height [mAU]	Area %
1	11.708	BB	0.3654	3820.70752	161.87769	93.9463
2	27.902	BB	0.7756	246.19621	3.94775	6.0537
Totals :				4066.90373	165.82543	

(*R*)-1-(2-hydroxynaphthalen-1-yl)isoquinoline 2-oxide ((*R*)-4a)

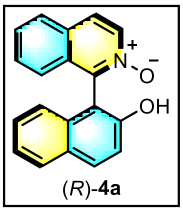

(*R*)-**4a** was obtained in **56% yield** and **60% ee** as a white solid. **HPLC analysis:** HPLC DAICEL CHIRALCEL AD-H, *n*-hexane/isopropanol = 75/25, 1.0 mL/min, λ = 254 nm, t_R (major) = 9.9 min, t_R (minor) = 17.7 min, ee = 60%.

Chiral HPLC spectrum of (*rac*)-**4a**

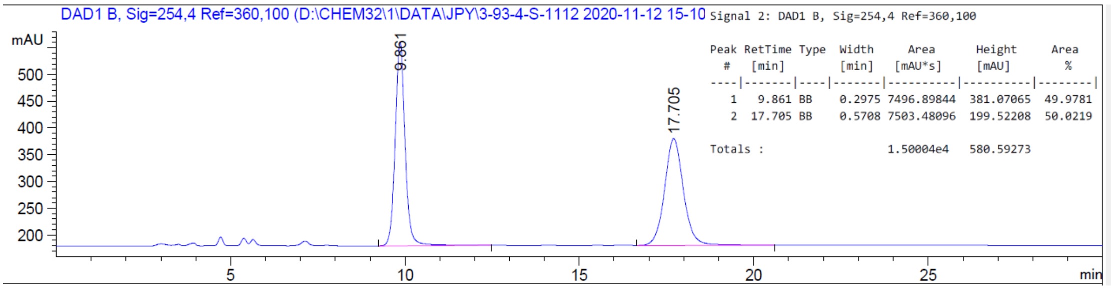

Signal 2: DAD1 B, Sig=254,4 Ref=360,100

Peak #	RetTime [min]	Type	Width [min]	Area [mAU*s]	Height [mAU]	Area %
1	9.861	BB	0.2975	7496.89844	381.07065	49.9781
2	17.705	BB	0.5708	7503.48096	199.52208	50.0219
Totals :				1.50004e4	580.59273	

Chiral HPLC spectrum of (*R*)-**4a**

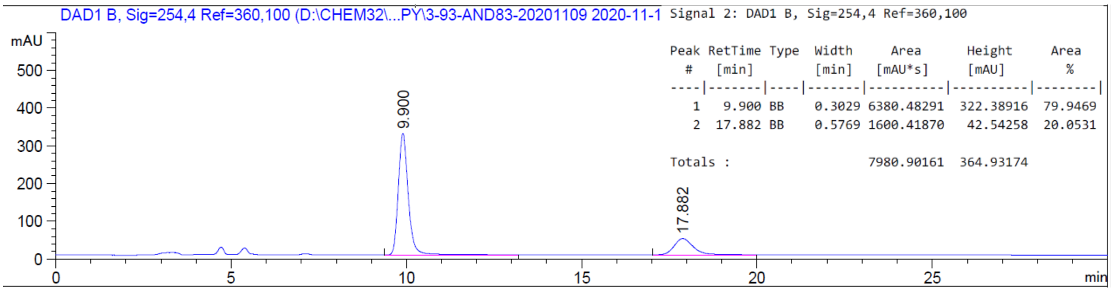

Signal 2: DAD1 B, Sig=254,4 Ref=360,100

Peak #	RetTime [min]	Type	Width [min]	Area [mAU*s]	Height [mAU]	Area %
1	9.900	BB	0.3029	6380.48291	322.38916	79.9469
2	17.882	BB	0.5769	1600.41870	42.54258	20.0531
Totals :				7980.90161	364.93174	

6. Gram-scale preparation of QUINOL and (*rac*)-QUINAP

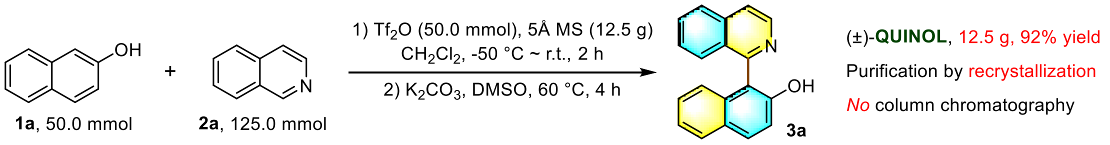

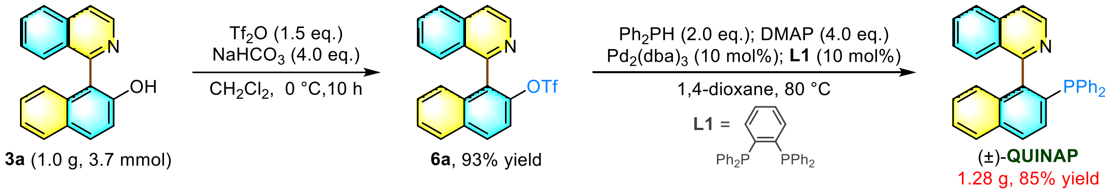

General procedure for gram-scale synthesis of QUINOL:

To a dried three-necked flask was added 5Å molecular sieve (12.5 g), isoquinoline **2a** (125.0 mmol) and CH_2Cl_2 (300 mL) under argon atmosphere. After the solution was cooled to -50 ℃, the solution of Tf_2O (50.0 mmol) in 50 mL of CH_2Cl_2 was added through a constant pressure funnel and mechanically stirred for half an hour until white solid salts appeared. After adding dropwise the solution of 2-naphthol **1a** (50.0 mmol) in CH_2Cl_2 (300 mL) within 10 minutes, the reaction was slowly raised to room temperature, and then continued to stir for 2 hours. The molecular sieve was removed by filtration, and the resulting filtrate was washed successively with 3M HCl (100 mL × 2), saturated sodium bicarbonate (100 mL × 2) and 100 mL of brine, and then dried over anhydrous Na_2SO_4. After evaporating the solvent under reduced pressure, the yielding intermediate **B** was dissolved in 60 mL of DMSO, and K_2CO_3 (13.8 g, 100 mmol) was then added. The resulting mixture was stirred at 60 ℃ for about 4 hours until the intermediate was consumed completely. The reaction system was adjusted to weakly acidic with 3M HCl, and then to weakly basic with saturated sodium bicarbonate. The mixture was diluted with 200 mL of water, and extracted with EA (200 mL × 3). The combined organic phase was washed with brine (200 mL × 2), dried over anhydrous Na_2SO_4 and concentrated to afford the crude product, which was recrystallized with EA and hexane to obtain QUINOL **3a** as a white solid (12.5 g, 92% yield).

General procedure for gram-scale synthesis of (*rac*)-QUINAP:

Tf_2O (1.5 eq.) was added dropwise under argon to a solution of QUINOL **3a** (1.0 g, 3.7 mmol) and $NaHCO_3$ (4.0 eq.) in dry CH_2Cl_2. After stirring at room temperature for 10 hours (monitored by TLC), the reaction was quenched with saturated aqueous $NaHCO_3$, then extracted with CH_2Cl_2. The combined organic layer was dried over anhydrous Na_2SO_4, filtered and concentrated. The residue was purified by flash chromatography eluted with PE/EA (10/1) to afford product **6a** as a yellow solid (1.39 g, 93% yield).

A Schlenk flask was charged with **6a** (1.39 g, 3.4 mmol), diphenylphosphine (2.0 eq.), $Pd_2(dab)_3$ (0.3 mmol, 10 mol%), **L1** (0.3 mmol, 10 mol%), DMAP (12 mmol, 4.0 eq.) and flushed with argon. 1,4-dioxane (8.0 mL) were added and the mixture was stirred at 80 ℃ for 24 hours. When TLC indicated the reaction was complete, the reaction mixture was concentrated under reduced pressure. The residue was purified by flash chromatography eluted with PE/CH_2Cl_2/EA (20/1/1) to afford (*rac*)-QUINAP. (1.28 g, 85% yield).

1-(isoquinolin-1-yl)naphthalen-2-yl trifluoromethanesulfonate (6a)

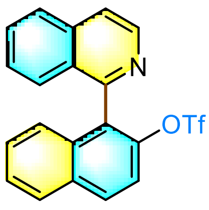

6a

^{1}H NMR (400 MHz, $CDCl_3$) δ 8.76 (d, J = 6.0 Hz, 1H), 8.08 (d, J = 8.8 Hz, 1H), 7.98-7.93 (m, 2H), 7.82 (d, J = 6.0 Hz, 1H), 7.71-7.67 (m, 1H), 7.59 (d, J = 9.2 Hz, 1H), 7.56-7.52 (m, 1H), 7.46-7.42 (m, 2H), 7.41-7.36 (m, 1H), 7.28-7.24 (m, 1H). **^{13}C NMR (100 MHz, $CDCl_3$)** δ 154.0, 145.0, 142.6, 136.3, 133.2, 132.5, 131.3, 130.6, 129.4, 128.4, 128.3, 127. 9, 127.8, 127.2, 127.1, 126.8, 126.5, 121.3, 119.5, 118.2 (q, J_{C-F} = 318.3 Hz). **^{19}F NMR (376 MHz, $CDCl_3$)** δ -74.51. **HRMS (ESI)** calcd for [M+H] $C_{20}H_{13}F_3NO_3S^+$, m/z: 404.0563; found: 404.0562.

1-(2-(diphenylphosphanyl)naphthalen-1-yl)isoquinoline

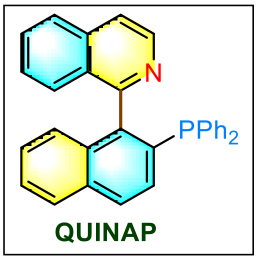

^{1}H NMR (400 MHz, CDCl$_3$) δ 8.61 (d, *J* = 5.6 Hz, 1H), 7.89-7.85 (m, 3H), 7.72 (d, *J* = 5.6 Hz, 1H), 7.57 (t, *J* = 7.6 Hz, 1H), 7.49-7.41 (m, 2H), 7.29-7.13 (m, 13H), 7.09 (d, *J* = 8.8 Hz, 1H). **^{13}C NMR (100 MHz, CDCl$_3$)** δ 160.5 (d, $J_{C\text{-}P}$ = 6.7 Hz), 144.3 (d, $J_{C\text{-}P}$ = 33.2 Hz), 142.3, 137.5 (d, $J_{C\text{-}P}$ = 10.0 Hz), 137.4 (d, $J_{C\text{-}P}$ = 9.4 Hz), 135.9, 134.9 (d, $J_{C\text{-}P}$ = 13.4 Hz), 133.8, 133.6, 133.3, 133.2, 132.7 (d, $J_{C\text{-}P}$ = 7.9 Hz), 130.1, 130.1 (d, $J_{C\text{-}P}$ = 1.8 Hz), 129.1 (d, $J_{C\text{-}P}$ = 3.0 Hz), 128.7, 128.5, 128.3, 128.3, 128.1, 128.0, 127.5, 127.1, 126.9 (d, $J_{C\text{-}P}$ = 2.0 Hz), 126.7, 126.7, 120.4. **^{31}P NMR (162 MHz, CDCl$_3$)** δ -14.16. **HRMS (ESI)** calcd for [M+H] $C_{31}H_{23}NP^{+}$, m/z: 440.1563; found: 440.1563.

7. Synthesis of (*R*)-QUINAP from (*R*)-QUINOL N-oxide

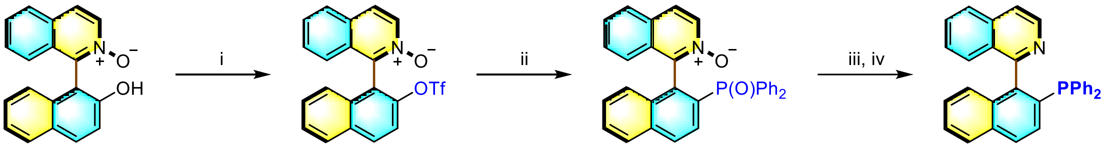

(*R*)-**4a**, 99% ee (0.75 mmol) | **6b**, 91% yield | **6c**, 75% yield, >99% ee | (*R*)-**QUINAP** 84% yield, 95% ee

Reaction conditions: i) Tf_2O (1.05 eq.), DMAP (2.0 eq.), CH_2Cl_2, r.t., 10 h; ii) diphenylphosphine oxide (2.0 eq.), $Pd(OAc)_2$ (12 mol%), **L1** (24 mol%), DIPEA (5.5 eq.), DMSO, 110 ℃, 24 h; iii) bis(pinacolato)diboron (1.0 eq.), diglyme, 70 ℃, 12 h; iv) $HSiCl_3$ (10.0 eq.), DIPEA (40.0 eq.), toluene, 70 ℃, 4 h.

Synthesis of (*R*)-6b: Tf_2O (1.05 eq.) was added dropwise under argon to a solution of (*R*)-**4a** (287 mg, 0.75 mmol) and DMAP (2.0 eq.) in dry CH_2Cl_2. After stirring at room temperature for 10 hours (monitored by TLC), the reaction was quenched with saturated $NaHCO_3$, then extracted with CH_2Cl_2. The combined organic layer was dried over anhydrous Na_2SO_4, filtered and concentrated. The residue was purified by flash chromatography eluted with PE/EA (1/2) to afford **6b** as a yellow solid (286 mg, 91% yield).

Synthesis of 6c: A Schlenk flask was charged with **6b** (264 mg, 0.63 mmol), diphenylphosphine oxide (242 mg, 1.2 mmol), $Pd(OAc)_2$ (16 mg, 12 mol%), **L1** (64 mg, 24 mol%) and flushed with argon. Diisopropylethylamine (575 μL, 3.3 mmol) and DMSO (8.0 mL) were added and the mixture was stirred at 100 ℃ for 24 hours. When TLC indicated the reaction was complete, the mixture was diluted with water (30 mL) and extracted with EA (3 x 30 mL). The combined organic layer was dried over anhydrous Na_2SO_4, filtered and concentrated to yield the crude product, which was purified by flash chromatography eluted with CH_2Cl_2/MeOH (100/1 to 20/1) to afford **6c** as a white solid (222 mg, 75% yield, >99% ee).

Synthesis of 6d[2]: $(PinB)_2$ (1.0 eq.) was added to the solution of **6c** (222 mg, 0.47 mmol) in diglyme (8.0 mL) under argon atmosphere, and the resulting mixture was stirred at 70 ℃ for 12 hours. After the completion of the reaction indicated by TLC, ethylenediamine (20 eq.) was added and the stirring was continued for 1 hour at room

temperature. The mixture was diluted with water (30 mL) and extracted with EA (3 × 15 mL). The combined organic layer was dried over Na_2SO_4 and concentrated under reduced pressure. The residue was purified by flash chromatography eluted with EA to afford **6d** as a white solid (200 mg, 93% yield, >99% ee).

Synthesis of (*R*)-QUINAP[3]: An argon filled Schlenk tube was charged with **6d** (200 mg, 0.44 mmol, 1.0 eq.) and toluene (8.0 ml). $HSiCl_3$ (475 μL, 10 eq.) and DIPEA (2.38 g, 40 eq.) were successively added and the mixture was heated to 70 ℃ for 4 hours. After the completion of the reaction indicated by TLC, the reaction was cooled to room temperature and diluted with CH_2Cl_2. 10 mL of 2M NaOH solution was then added carefully and the aqueous layer was extracted with CH_2Cl_2 (3 × 15 mL). The organic phase was dried over Na_2SO_4 and concentrated under reduced pressure. The residue was purified by flash chromatography eluted with PE/EA (3/1) to afford (*R*)-**QUINAP** as a white solid (174 mg, 90% yield, 95% ee), and its absolute configuration was further confirmed by comparison with the chiral HPLC spectrum of commercial (*R*)-**QUINAP.**

(*R*)-1-(2-(((trifluoromethyl)sulfonyl)oxy)naphthalen-1-yl)isoquinoline 2-oxide (6b)

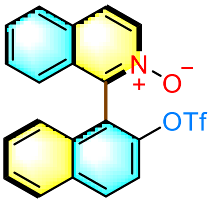

6b

^{1}H NMR (400 MHz, DMSO-d_6) δ 8.47 (d, *J* = 7.6 Hz, 1H), 8.42 (d, *J* = 9.2 Hz, 1H), 8.23-8.19 (m, 2H), 8.11 (d, *J* = 8.0 Hz, 1H), 7.78 (d, *J* = 9.2 Hz, 1H), 7.71-7.61 (m, 2H), 7.55-7.49 (m, 2H), 7.21 (d, *J* = 8.4 Hz, 1H), 6.95 (d, *J* = 8.4 Hz, 1H). **^{13}C NMR (100 MHz, DMSO-d_6)** δ 145.2, 137.5, 137.5, 132.9, 132.4, 131.6, 129.9, 129.3, 128.8, 128.7, 128.3, 127.9, 127.7, 127.5, 125.5, 125.4, 123.6, 121.5, 119.5, 117.6 (q, $J_{C\text{-}F}$ = 320.0 Hz). **^{19}F NMR (376 MHz, DMSO-d_6)** δ -74.73. **HRMS (ESI)** calcd for [M+H] $C_{20}H_{13}F_3NO_4S^+$, m/z: 420.0512; found: 420.0510.

(*R*)-1-(2-(diphenylphosphoryl)naphthalen-1-yl)isoquinoline 2-oxide (6c)

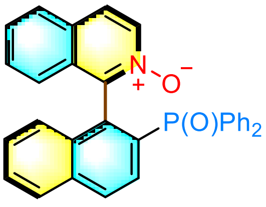

6c

^{1}H NMR (400 MHz, CDCl$_3$) δ 8.05-8.01 (m, 2H), 7.94 (d, *J* = 8.4 Hz, 1H), 7.79 (dd, *J* = 11.4, 8.6 Hz, 1H), 7.74-7.69 (m, 2H), 7.67 (d, *J* = 8.0 Hz, 1H), 7.56 (t, *J* = 7.2 Hz, 1H), 7.49-7.41 (m, 5H), 7.38-7.33 (m, 3H), 7.21 (t, *J* = 7.6 Hz, 2H), 7.10-7.05 (m, 3H), 6.90 (d, *J* = 8.4 Hz, 1H). **^{13}C NMR (100 MHz, CDCl$_3$)** δ 143.1 (d, $J_{C\text{-}P}$ = 3.8 Hz), 136.9, 134.9 (d, $J_{C\text{-}P}$ = 2.1 Hz), 134.2 (d, $J_{C\text{-}P}$ = 6.9 Hz), 133.4, 132.3 (d, $J_{C\text{-}P}$ = 3.7 Hz), 132.1 (d, $J_{C\text{-}P}$ = 9.6 Hz), 131.7 (d, $J_{C\text{-}P}$ = 2.8 Hz), 131.7, 131.5, 131.5, 131.4, 131.4, 131.3, 131.4, 130.7, 129.6 (d, $J_{C\text{-}P}$ = 11.7 Hz), 129.0, 128.8, 128.7, 128.6, 128.5, 128.4, 128.3, 128.3, 128.2, 128.1, 127.8 (d, $J_{C\text{-}P}$ = 12.4 Hz), 126.6, 125.4 (d, $J_{C\text{-}P}$ = 13.8 Hz), 124.4. **^{31}P NMR (162 MHz, CDCl$_3$)** δ 28.26. **HRMS (ESI)** calcd for [M+H] $C_{31}H_{23}NO_2P^+$, m/z: 472.1461; found: 472.1460. **HPLC analysis:** DAICEL

CHIRALCEL AD-H, *n*-hexane/isopropanol = 75/25, 1.0 mL/min, λ = 230 nm, t_R (major) = 31.2 min, t_R (minor) = 57.6 min, ee > 99%.

Chiral HPLC spectrum of (*rac*)-**6c**

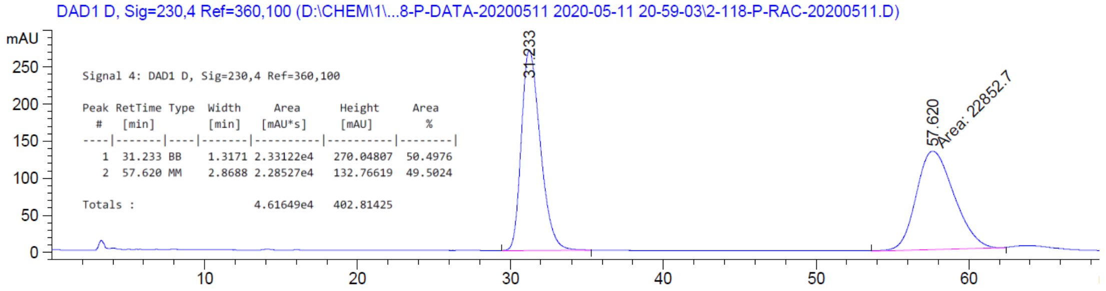

Chiral HPLC spectrum of **6c**

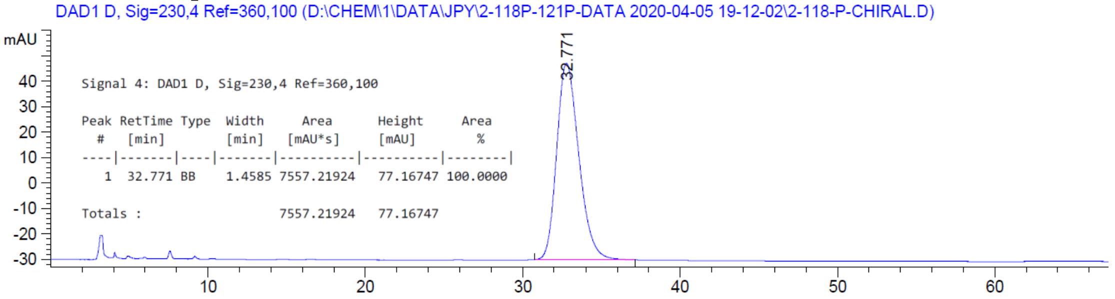

(*R*)-(1-(isoquinolin-1-yl)naphthalen-2-yl)diphenylphosphine oxide (6d)

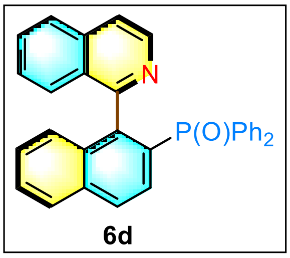

^{1}H NMR (400 MHz, CDCl$_3$) δ 8.37 (d, *J* = 5.6 Hz, 1H), 8.16-8.06 (m, 2H), 7.94 (d, *J* = 8.8 Hz, 1H), 7.88-7.82 (m, 2H), 7.62 (d, *J* = 8.4 Hz, 1H), 7.54-7.36 (m, 6H), 7.26-7.17 (m, 3H), 7.11-7.06 (m, 2H), 6.98-6.94 (m, 1H), 6.87 (d, *J* = 8.8 Hz, 1H), 6.84-6.79 (m, 2H). **^{13}C NMR (100 MHz, CDCl$_3$)** δ 158.4 (d, $J_{C\text{-}P}$ = 5.0 Hz), 141.6 (d, $J_{C\text{-}P}$ = 9.2 Hz), 141.6, 135.5, 134.8 (d, $J_{C\text{-}P}$ = 2.3 Hz), 132.7 (d, $J_{C\text{-}P}$ = 103.9 Hz), 132.6 (d, $J_{C\text{-}P}$ = 18.9 Hz), 132.6, 131.6 (d, $J_{C\text{-}P}$ = 2.8 Hz), 132.6 (d, $J_{C\text{-}P}$ = 10.1 Hz), 131.51, 131.1 (d, $J_{C\text{-}P}$ = 10.2 Hz), 130.5 (d, $J_{C\text{-}P}$ = 2.9 Hz), 130.0, 129.9 (d, $J_{C\text{-}P}$ = 100.5 Hz), 129.2, 128.8 (d, $J_{C\text{-}P}$ = 11.4 Hz), 128.5 (d, $J_{C\text{-}P}$ = 9.9 Hz), 128.2 (d, $J_{C\text{-}P}$ = 24.0 Hz), 128.2, 127.6, 127.3, 127.2, 127.1, 127.0, 126.6, 121.2. **^{31}P NMR (162 MHz, CDCl$_3$)** δ 30.07. **HRMS (ESI)** calcd for [M+H] $C_{31}H_{23}NOP^+$, m/z: 456.1512; found: 456.1512. **HPLC analysis:** DAICEL CHIRALCEL IA, *n*-hexane/isopropanol = 85/15,

1.0 mL/min, λ = 230 nm, t_R (minor) = 21.4 min, t_R (major) = 25.9 min, ee > 99%.

Chiral HPLC spectrum of (*rac*)-**6d**

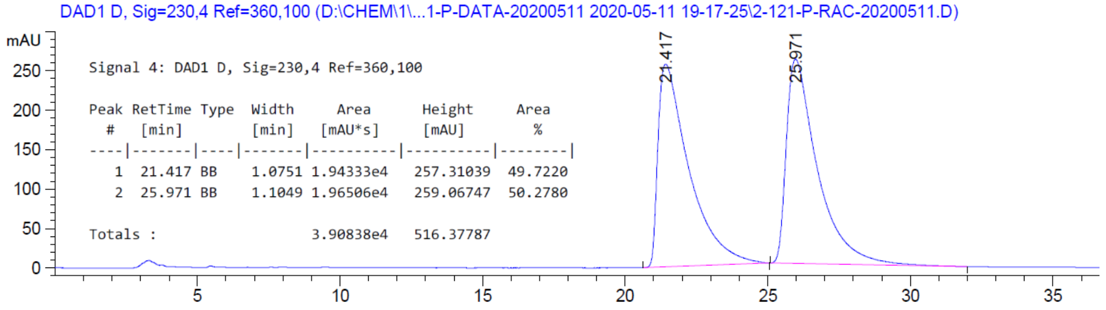

Chiral HPLC spectrum of **6d**

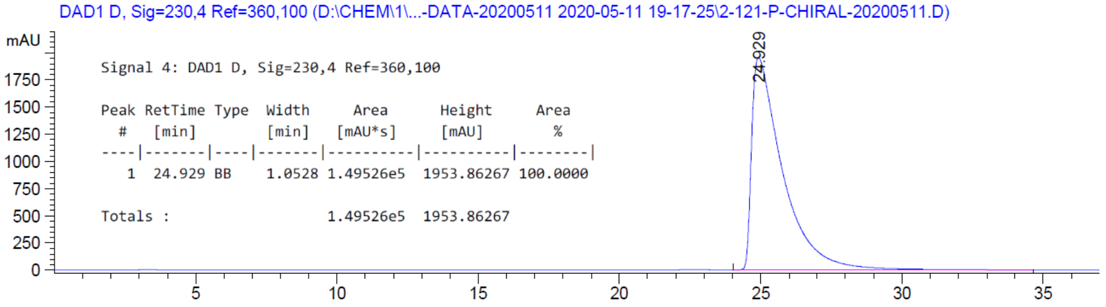

(*R*)-1-(2-(diphenylphosphanyl)naphthalen-1-yl)isoquinoline

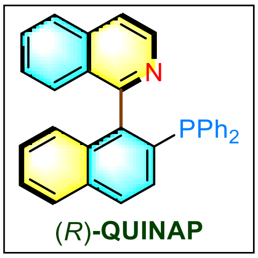

^{1}H NMR (400 MHz, $CDCl_3$) δ 8.61 (d, *J* = 5.6 Hz, 1H), 7.89-7.85 (m, 3H), 7.72 (d, *J* = 5.6 Hz, 1H), 7.57 (t, *J* = 7.6 Hz, 1H), 7.49-7.41 (m, 2H), 7.29-7.13 (m, 13H), 7.09 (d, *J* = 8.8 Hz, 1H). **^{13}C NMR (100 MHz, $CDCl_3$)** δ 160.5 (d, $J_{C\text{-}P}$ = 6.7 Hz), 144.3 (d, $J_{C\text{-}P}$ = 33.2 Hz), 142.3, 137.5 (d, $J_{C\text{-}P}$ = 10.0 Hz), 137.4 (d, $J_{C\text{-}P}$ = 9.4 Hz), 135.9, 134.9 (d, $J_{C\text{-}P}$ = 13.4 Hz), 133.8, 133.6, 133.3, 133.2, 132.7 (d, $J_{C\text{-}P}$ = 7.9 Hz), 130.1, 130.1 (d, $J_{C\text{-}P}$ = 1.8 Hz), 129.1 (d, $J_{C\text{-}P}$ = 3.0 Hz), 128.7, 128.5, 128.3, 128.3, 128.1, 128.0, 127.5, 127.1, 126.9 (d, $J_{C\text{-}P}$ = 2.0 Hz), 126.7, 126.7, 120.4. **^{31}P NMR (162 MHz, $CDCl_3$)** δ -14.16. **HRMS (ESI)** calcd for [M+H] $C_{31}H_{23}NP^+$, m/z: 440.1563; found: 440.1563. **HPLC analysis:** DAICEL CHIRALCEL ID, *n*-hexane/isopropanol = 85/15, 1.0 mL/min, λ = 230 nm, t_R (minor) = 11.6 min, t_R (major) = 16.3 min, ee = 95%.

Chiral HPLC spectrum of (*rac*)-**QUINAP**

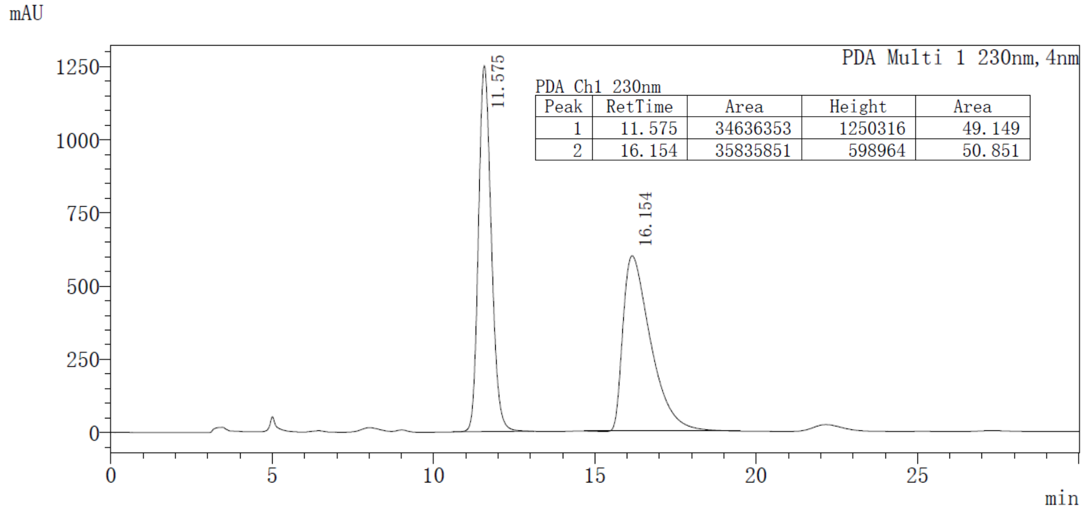

Chiral HPLC spectrum of commercial (*R*)-**QUINAP**

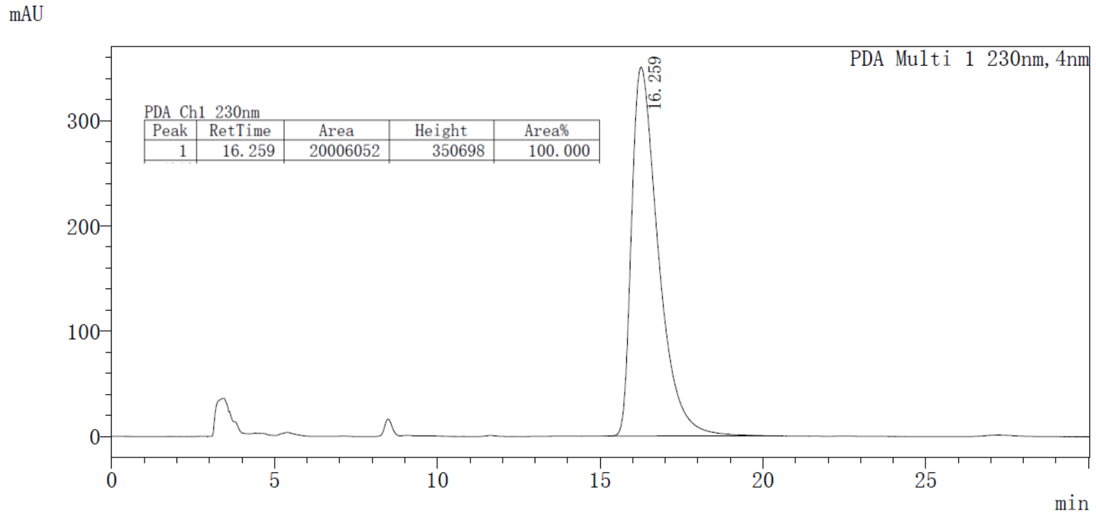

Chiral HPLC spectrum of (*R*)-**QUINAP**

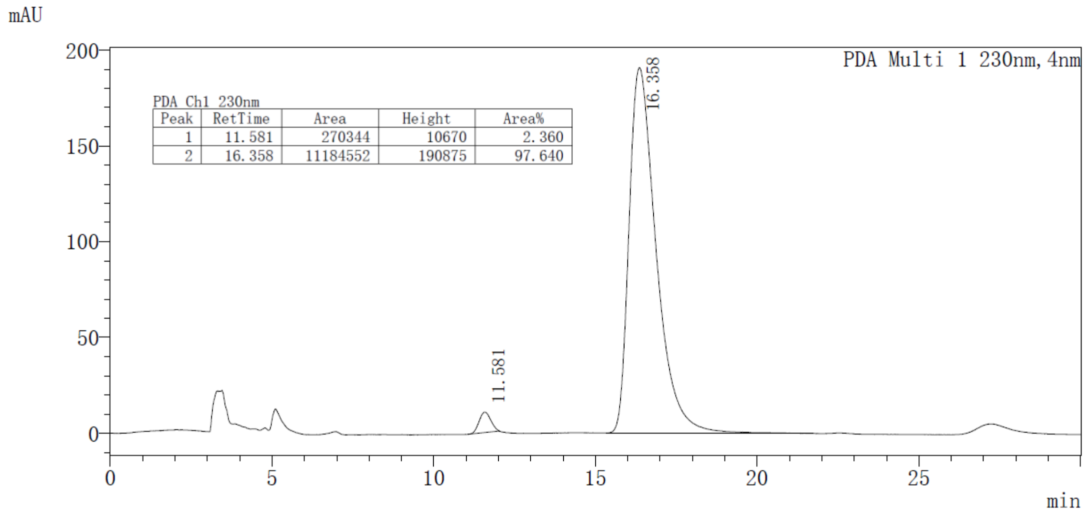

8. Control experiment for mechanism investigation

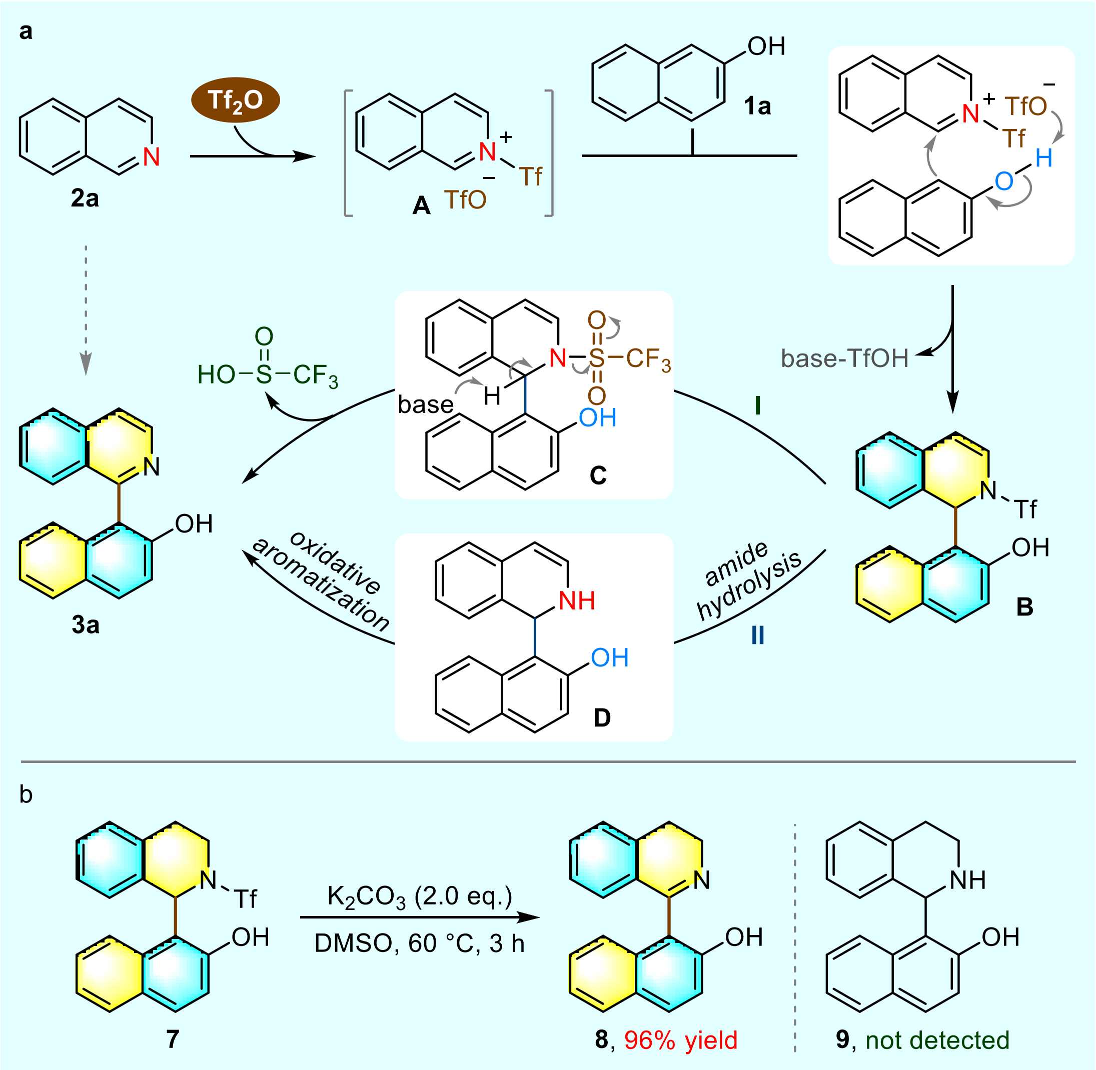

Proposed reaction mechanism and control experiment. a Proposed reaction pathway. **b** Control experiment.

Procedures for the synthesis of compound 7:

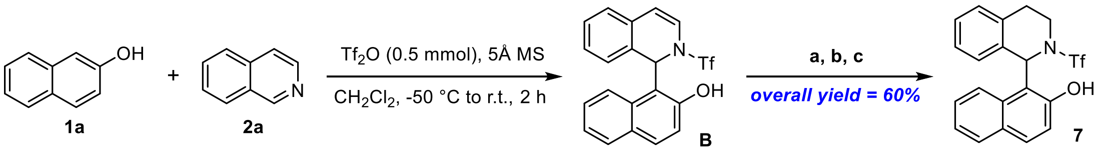

(a) 4-Methoxybenzyl bromide (3 eq.), Bu_4NBr (10 mol%), Cs_2CO_3 (4.0 eq.), toluene, r.t., 10 h; (b) Pd/C, H_2 (1 atm), THF, r.t., 10 h; (c) HCl, EtOH, 60 °C, 10 h

Synthetic procedure of intermediate B: To a dried three-necked flask was added 5Å molecular sieve (500 mg), isoquinoline **2a** (2.5 mmol) and CH_2Cl_2 (8 mL) under argon. After the solution was cooled to -50 ℃, Tf_2O (1.0 mmol) was added and stirred for half an hour until white solid salts appeared. After adding dropwise the solution of

2-naphthol **1a** (1.0 mmol) in CH_2Cl_2 (8 mL) within 10 minutes, the reaction was slowly raised to room temperature, and then continued to stir for 2 hours. The molecular sieve was removed by filtration, and the resulting filtrate was washed successively with 3M HCl (20 mL × 2), saturated sodium bicarbonate (20 mL × 2) and 20 mL of brine, dried over anhydrous Na_2SO_4 and concentrated to afford the crude product, which was purified by flash chromatography eluted with PE/EA (10/1 to 3/1) to afford the intermediate **B** as a white solid (393 mg, 97% yield).

Synthetic procedure of intermediate 7: 4-Methoxybenzyl bromide (3.0 eq.) was added to a solution of intermediate **B** (1.0 mmol, 1.0 eq.), Bu_4NBr (10 mol%) and Cs_2CO_3 (4.0 eq.) in toluene. The reaction mixture was stirred at room temperature for 10 hours until the intermediate **B** was completely consumed (monitored by TLC). The solid was removed by filtration, and the filtrate was washed successively with brine, dried over anhydrous Na_2SO_4 and concentrated to afford the crude product, which was used directly in the next step.

The intermediate obtained above was dissolved in 15 mL of THF, and then Pd/C (10 mol%) was added. The resulting mixture was stirred at room temperature under H_2 atmosphere for about 10 hours. When TLC indicated the complete consumption of starting material, the reaction mixture was filtered through celite and the filtrate was concentrated under reduced pressure.

The obtained solid was dissolved with ethanol, and then HCl (0.5 ml, 12 mol/L) was added. The resulting mixture was heated to 60 ℃ for 10 hours. After the reaction was completed, saturated $NaHCO_3$ solution was added to quench the reaction, and then extracted with EA (3 x 30 mL). The combined organic portions was dried over Na_2SO_4 and concentrated under reduced pressure to yield the crude product, which was purified by flash chromatography eluted with PE/EA (5/1) to afford the compound **7** as oily liquid (237 mg, 60% overall yield for three steps).

Synthetic procedure of intermediate 8: To a tube was added compound **7** (203 mg, 0.5 mmol), K_2CO_3 (138 mg, 1.0 mmol) and DMSO (4 mL), then the mixture was

stirred at 60 ℃ for about 3 hours until the intermediate was consumed completely. The reaction system was adjusted to weakly acidic with 3M HCl, and then to weakly basic with saturated sodium bicarbonate. The mixture was diluted with 20 mL of water, and extracted with EA (20 mL × 3). The combined organic phase was washed with brine (20 mL × 2), dried over anhydrous Na_2SO_4 and concentrated to afford the crude product, which was purified by column chromatography eluted with PE/EA (10/1 to 3/1) to afford the compound **8** as a yellow solid (131 mg, 96% yield).

1-(2-((trifluoromethyl)sulfonyl)-1,2-dihydroisoquinolin-1-yl)naphthalen-2-ol (intermediate B)

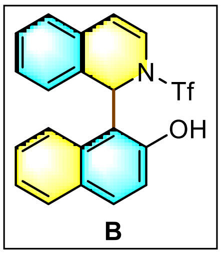

^{1}H NMR (400 MHz, DMSO-*d*$_6$, 80 ℃) δ 10.12 (s, 1H), 8.11 (d, *J* = 8.6 Hz, 1H), 7.80-7.75 (m, 2H), 7.61 (s, 1H), 7.44-7.40 (m, 1H), 7.29-7.22 (m, 2H), 7.18-7.11 (m, 2H), 7.06-7.02 (m, 1H), 6.91 (d, *J* = 7.8 Hz, 1H), 6.74 (d, *J* = 7.9 Hz, 1H), 6.33 (d, *J* = 8.0 Hz, 1H). **^{13}C NMR (100 MHz, DMSO-*d*$_6$, 80 ℃)** δ 153.2, 131.4, 130.1, 128.6, 128.3, 128.0, 127.4, 127.1, 126.6, 125.7, 125.4, 122.8, 122.3, 122.1, 121.8, 119.3 (q, *J* = 325.3 Hz), 118.1, 110.6, 53.9. **^{19}F NMR (376 MHz, DMSO-*d*$_6$)** δ -73.76. **HRMS (ESI)** calcd for [M-H] $C_{20}H_{13}F_3NO_3S^-$, m/z: 404.0574; found: 404.0574.

1-(2-((trifluoromethyl)sulfonyl)-1,2,3,4-tetrahydroisoquinolin-1-yl)naphthalen-2-ol (7)

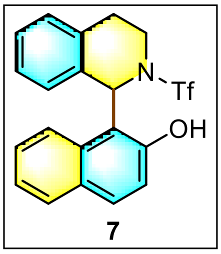

^{1}H NMR (400 MHz, DMSO-*d*$_6$, 80 ℃) δ 9.79 (s, 1H), 8.09 (d, *J* = 8.8 Hz, 1H), 7.84-7.79 (m, 2H), 7.48 (t, *J* = 7.7 Hz, 1H), 7.34-7.26 (m, 2H), 7.20-7.14 (m, 2H), 7.02-6.99 (m, 2H), 6.56 (d, *J* = 8.0 Hz, 1H), 4.39-4.08 (m, 2H), 3.17-3.12 (m, 2H).

^{13}C NMR (100 MHz, DMSO-d_6, 80 ℃) δ 153.6, 135.0, 133.0, 132.9, 129.9, 128.1, 128.0, 127.8, 126.4, 126.0, 126.0, 125.3, 122.0, 121.8, 119.2 (q, J_{C-F} = 323.6 Hz), 118.3, 52.9, 43.5, 29.3. **^{19}F NMR (376 MHz, DMSO-d_6, 80 ℃)** δ -75.41. **HRMS (ESI)** calcd for [M-H] $C_{20}H_{15}F_3NO_3S^-$, m/z: 406.0730; found: 406.0730.

1-(3,4-dihydroisoquinolin-1-yl)naphthalen-2-ol (8)

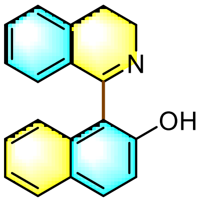

8

^{1}H NMR (400 MHz, DMSO-d_6) δ 9.88 (s, 1H), 7.86-7.82 (m, 2H), 7.44 (d, *J* = 8.0 Hz, 1H), 7.37-7.24 (m, 5H), 7.12-7.08 (m, 1H), 6.70 (d, *J* = 8.0 Hz, 1H), 3.94-3.85 (m, 2H), 2.96-2.82 (m, 2H). **^{13}C NMR (100 MHz, DMSO-d_6)** δ 164.3, 152.4, 136.9, 133.0, 130.6, 129.6, 129.5, 128.0, 127.7, 127.4, 126.9, 126.4, 126.2, 123.8, 122.7, 118.4, 118.4, 47.0, 25.3. **HRMS (ESI)** calcd for [M+H] $C_{19}H_{16}NO^+$, m/z: 274.1226; found: 274.1223.

9. Crystal data and structure of compounds 3a and (*R*)-4a

Compound 3a: The crystal structure of compound **3a** has been deposited at the Cambridge Crystallographic Data Centre (**CCDC 2005905**). The data is available free of charge at www.ccdc.cam.ac.uk/conts/retrieving.html.

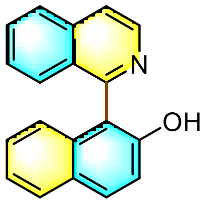

3a
CCDC 2005905

≡

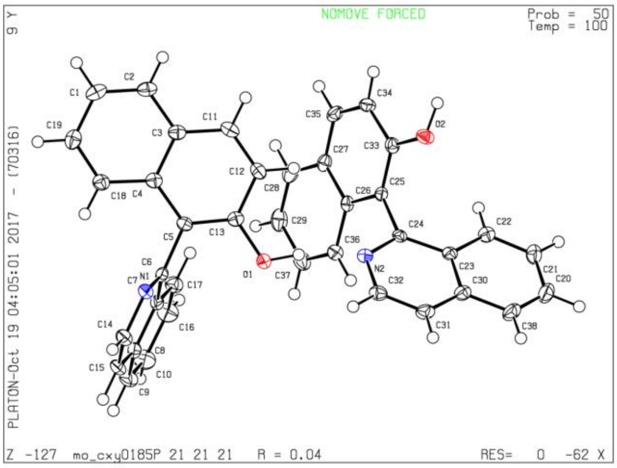

(1) Crystal data and structure refinement for 3a.

Identification code	mo_cxy0185_0m
Empirical formula	$C_{38}H_{26}N_2O_2$
Formula weight	542.61
Temperature/K	100
Crystal system	orthorhombic
Space group	$P2_12_12_1$
a/Å	9.719(6)
b/Å	12.619(6)
c/Å	22.643(12)
α/°	90
β/°	90
γ/°	90
Volume/Å^3	2777(3)
Z	4
ρ_{calc}g/cm^3	1.298
μ/mm^{-1}	0.080
F(000)	1136.0
Crystal size/mm^3	0.4 × 0.26 × 0.23
Radiation	MoKα (λ = 0.71073)
2Θ range for data collection/°	4.56 to 55.178
Index ranges	$-12 \leq h \leq 12$, $-15 \leq k \leq 16$, $-29 \leq l \leq 29$
Reflections collected	17088

Independent reflections	6406 [R_{int} = 0.0438, R_{sigma} = 0.0574]
Data/restraints/parameters	6406/0/386
Goodness-of-fit on F^2	1.053
Final R indexes [I>=2σ (I)]	R_1 = 0.0409, wR_2 = 0.0792
Final R indexes [all data]	R_1 = 0.0549, wR_2 = 0.0843
Largest diff. peak/hole / e Å^{-3}	0.21/-0.19
Flack parameter	0.2(7)

(2) Fractional Atomic Coordinates (×10^4) and Eq.alent Isotropic Displacement Parameters (Å^2×10^3) for 3a. U_{eq} is defined as 1/3 of of the trace of the orthogonalised U_{IJ} tensor.

**Atom**	***x***	***y***	***z***	**U(eq)**
O1	3537.8(16)	4579.2(12)	6472.0(7)	18.9(3)
O2	3603.5(17)	2613.2(13)	8611.6(6)	20.5(4)
N1	5037.4(19)	6467.7(15)	5534.9(8)	17.5(4)
N2	2065.8(19)	4200.3(14)	7482.7(8)	17.0(4)
C1	9977(2)	4381.8(19)	5991.9(10)	23.7(5)
C2	9203(2)	4004(2)	6444.7(10)	22.0(5)
C3	7767(2)	4143.9(18)	6457.2(10)	18.2(5)
C4	7112(2)	4703.9(18)	5990.6(9)	16.6(5)
C5	5658(2)	4834.3(17)	6005.7(9)	15.1(5)
C6	4942(2)	5421.7(18)	5528.9(9)	15.6(5)
C7	4215(2)	4860.7(18)	5083.2(9)	16.5(5)
C8	3567(2)	5456.5(19)	4633.0(9)	18.9(5)
C9	2859(3)	4914(2)	4180.5(10)	26.1(6)
C10	2802(3)	3834(2)	4179.0(11)	29.6(6)
C11	6952(2)	3733.9(18)	6920.8(10)	20.3(5)
C12	5575(2)	3861.1(18)	6927.1(9)	19.2(5)
C13	4914(2)	4417.5(17)	6465.1(9)	15.9(4)
C14	4404(2)	7026.0(19)	5098.3(9)	20.5(5)
C15	3678(2)	6571(2)	4655.4(9)	21.4(5)
C16	3430(3)	3242(2)	4629.7(10)	26.4(6)
C17	4116(2)	3745.1(19)	5073.5(10)	21.3(5)
C18	7950(2)	5105.2(18)	5532.1(9)	19.1(5)
C19	9339(2)	4941.4(19)	5530.9(10)	22.8(5)
C20	-1843(2)	2850(2)	8709.5(10)	26.5(6)
C21	-949(2)	1990(2)	8632.5(10)	23.2(5)
C22	254(2)	2110.3(18)	8324.4(9)	18.6(5)
C23	590(2)	3101.7(18)	8076.0(9)	15.1(4)
C24	1796(2)	3272.5(17)	7734.4(9)	14.0(4)

C25	2858(2)	2441.2(17)	7628.0(9)	14.5(4)
C26	3031(2)	1998.9(17)	7053.7(9)	15.7(5)
C27	4173(2)	1334.7(18)	6939.2(9)	17.1(5)
C28	4341(2)	905.9(19)	6369.7(10)	23.0(5)
C29	3441(3)	1128(2)	5930.7(10)	25.8(6)
C30	-321(2)	3969.6(18)	8155.2(9)	18.4(5)
C31	25(2)	4946.8(19)	7896.8(10)	21.5(5)
C32	1189(2)	5021.9(18)	7569.0(9)	19.7(5)
C33	3787(2)	2199.2(17)	8065.3(9)	16.4(5)
C34	4958(2)	1575.5(17)	7938.4(9)	18.4(5)
C35	5132(2)	1152.8(18)	7394.4(10)	20.0(5)
C36	2101(2)	2197.2(19)	6588.7(9)	18.9(5)
C37	2300(3)	1775(2)	6041.2(10)	24.6(5)
C38	-1540(2)	3820(2)	8479.4(10)	24.4(5)

(3) Anisotropic Displacement Parameters (Å^2×10^3) for 3a. The Anisotropic displacement factor exponent takes the form: $-2\pi^2[h^2a^{*2}U_{11}+2hka^*b^*U_{12}+\ldots]$.

**Atom**	**U_{11}**	**U_{22}**	**U_{33}**	**U_{23}**	**U_{13}**	**U_{12}**
O1	15.7(8)	23.4(9)	17.6(7)	5.2(7)	0.0(7)	-0.6(7)
O2	23.8(8)	23.9(9)	13.9(7)	-1.0(6)	-2.2(7)	7.0(7)
N1	17.8(9)	19.6(10)	15.0(8)	2.8(7)	-0.7(8)	-0.2(8)
N2	16.1(9)	17.6(10)	17.4(9)	1.9(8)	-0.9(8)	2.2(8)
C1	15.5(11)	24.8(13)	30.9(12)	-6.5(10)	-3.1(10)	3(1)
C2	18.9(11)	24.2(12)	23.0(11)	-2.1(10)	-6.3(10)	3.3(10)
C3	18.7(11)	17.3(11)	18.5(10)	-3.2(9)	-3.3(9)	1.7(9)
C4	18.3(11)	16.7(11)	14.7(10)	-4.2(9)	-2.8(9)	-0.4(9)
C5	17.2(11)	15.8(11)	12.4(10)	-1.7(9)	-3.3(9)	-0.6(9)
C6	11.9(10)	21.7(12)	13.1(10)	-0.2(9)	1.8(9)	-1.0(9)
C7	14.2(10)	22.6(12)	12.6(10)	0.8(9)	0.5(9)	-1.0(9)
C8	15.2(11)	26.4(13)	15.1(10)	2.2(9)	1.6(9)	-1.9(10)
C9	24.3(12)	35.0(15)	18.9(11)	3.9(10)	-7(1)	-3.8(12)
C10	30.2(14)	36.2(16)	22.4(12)	-5.9(11)	-9.3(11)	-5.1(12)
C11	25.8(12)	18.2(12)	16.9(11)	1.8(9)	-5.8(10)	1.4(10)
C12	22.7(12)	19.1(12)	15.9(10)	3.3(9)	-1.3(9)	-1.9(10)
C13	16(1)	15.6(11)	16(1)	-3.5(9)	-2.7(9)	-0.9(9)
C14	23.9(12)	19.4(12)	18.2(11)	3.3(9)	-0.6(10)	1.3(10)
C15	22.3(12)	26.5(13)	15.3(10)	7.1(9)	-2.7(10)	-0.7(11)
C16	29.8(14)	23.5(13)	25.8(12)	-4.8(10)	-5.7(11)	-3.5(11)
C17	22.2(12)	22.7(12)	19.0(11)	-0.3(9)	-2.5(10)	1.4(10)

C18	20.2(11)	21.3(12)	15.7(10)	-2.6(9)	-2.7(9)	0.2(10)
C19	21.3(12)	25.4(13)	21.5(11)	-4.9(10)	2.6(10)	-5.1(11)
C20	18.9(12)	36.8(15)	23.8(12)	-2.2(11)	6(1)	-1.2(11)
C21	23.2(12)	25.2(13)	21.2(11)	0.8(10)	3.1(10)	-5.6(10)
C22	18.1(11)	18.9(12)	18.7(11)	-2.5(9)	-1.3(9)	0.6(10)
C23	14.8(10)	19.0(11)	11.5(9)	-1.8(8)	-2.9(8)	0.3(9)
C24	14.9(11)	16.7(11)	10.5(9)	-0.2(8)	-2.6(8)	1.5(9)
C25	13.3(10)	14.7(11)	15.5(10)	2.2(8)	2.5(9)	0.1(9)
C26	16.2(10)	15.2(11)	15.8(10)	1.7(9)	2.6(9)	-2.4(9)
C27	17.1(11)	14.3(11)	19.9(11)	-1.4(9)	4.3(9)	-1.6(9)
C28	22.1(12)	22.3(13)	24.8(12)	-5.1(10)	7.2(10)	-0.9(10)
C29	30.8(13)	28.4(14)	18.3(11)	-7.5(10)	6(1)	-5.7(12)
C30	16.1(11)	22.5(12)	16.6(10)	-3.8(9)	-1.8(9)	1.9(10)
C31	21.1(11)	18.8(12)	24.6(11)	-1.4(9)	-0.6(10)	8.6(10)
C32	21.4(11)	14.7(11)	22.9(11)	2.5(9)	-2.8(10)	3(1)
C33	18.7(11)	13.7(11)	16.7(10)	1.1(8)	2.0(9)	-0.3(9)
C34	16.7(11)	18.0(12)	20.5(11)	4.6(9)	-2.6(9)	1.8(9)
C35	14.5(11)	18.2(11)	27.3(12)	1.1(9)	4.0(9)	3.7(9)
C36	18.5(11)	19.5(12)	18.8(11)	2.4(9)	-0.5(9)	0.2(10)
C37	27.2(13)	29.7(14)	16.9(11)	0.5(10)	-2.5(10)	-5.6(11)
C38	17.2(11)	29.5(14)	26.5(12)	-4.3(11)	3.7(10)	6.2(11)

(4) Bond Lengths for 3a.

**Atom**	**Atom**	**Length/Å**	**Atom**	**Atom**	**Length/Å**
O1	C13	1.353(3)	C14	C15	1.354(3)
O2	C33	1.355(3)	C16	C17	1.363(3)
N1	C6	1.323(3)	C18	C19	1.366(3)
N1	C14	1.361(3)	C20	C21	1.401(3)
N2	C24	1.328(3)	C20	C38	1.362(4)
N2	C32	1.357(3)	C21	C22	1.370(3)
C1	C2	1.359(3)	C22	C23	1.410(3)
C1	C19	1.405(3)	C23	C24	1.421(3)
C2	C3	1.407(3)	C23	C30	1.420(3)
C3	C4	1.422(3)	C24	C25	1.491(3)
C3	C11	1.413(3)	C25	C26	1.425(3)
C4	C5	1.423(3)	C25	C33	1.375(3)
C4	C18	1.413(3)	C26	C27	1.415(3)
C5	C6	1.483(3)	C26	C36	1.410(3)
C5	C13	1.372(3)	C27	C28	1.408(3)

C6	C7	1.421(3)	C27	C35	1.409(3)
C7	C8	1.415(3)	C28	C29	1.354(3)
C7	C17	1.411(3)	C29	C37	1.399(4)
C8	C9	1.411(3)	C30	C31	1.406(3)
C8	C15	1.412(3)	C30	C38	1.407(3)
C9	C10	1.364(4)	C31	C32	1.356(3)
C10	C16	1.405(4)	C33	C34	1.413(3)
C11	C12	1.348(3)	C34	C35	1.353(3)
C12	C13	1.414(3)	C36	C37	1.363(3)

(5) Bond Angles for 3a.

Atom	Atom	Atom	Angle/°	Atom	Atom	Atom	Angle/°
C6	N1	C14	118.50(19)	C18	C19	C1	120.7(2)
C24	N2	C32	119.21(19)	C38	C20	C21	121.0(2)
C2	C1	C19	119.5(2)	C22	C21	C20	120.5(2)
C1	C2	C3	121.4(2)	C21	C22	C23	119.9(2)
C2	C3	C4	119.4(2)	C22	C23	C24	122.9(2)
C2	C3	C11	121.6(2)	C22	C23	C30	119.3(2)
C11	C3	C4	118.9(2)	C30	C23	C24	117.8(2)
C3	C4	C5	119.0(2)	N2	C24	C23	122.00(19)
C18	C4	C3	117.8(2)	N2	C24	C25	114.49(18)
C18	C4	C5	123.3(2)	C23	C24	C25	123.5(2)
C4	C5	C6	120.4(2)	C26	C25	C24	120.31(18)
C13	C5	C4	119.8(2)	C33	C25	C24	119.64(18)
C13	C5	C6	119.8(2)	C33	C25	C26	119.5(2)
N1	C6	C5	117.28(19)	C27	C26	C25	119.45(19)
N1	C6	C7	122.6(2)	C36	C26	C25	122.4(2)
C7	C6	C5	120.08(19)	C36	C26	C27	118.1(2)
C8	C7	C6	117.9(2)	C28	C27	C26	119.1(2)
C17	C7	C6	122.8(2)	C28	C27	C35	122.0(2)
C17	C7	C8	119.3(2)	C35	C27	C26	118.80(19)
C9	C8	C7	118.9(2)	C29	C28	C27	121.2(2)
C9	C8	C15	123.1(2)	C28	C29	C37	120.1(2)
C15	C8	C7	118.0(2)	C31	C30	C23	118.3(2)
C10	C9	C8	120.4(2)	C31	C30	C38	122.5(2)
C9	C10	C16	120.8(2)	C38	C30	C23	119.2(2)
C12	C11	C3	121.4(2)	C32	C31	C30	119.3(2)
C11	C12	C13	120.2(2)	C31	C32	N2	123.3(2)
O1	C13	C5	118.16(19)	O2	C33	C25	119.0(2)

O1	C13	C12	121.03(19)	O2	C33	C34	120.44(19)
C5	C13	C12	120.8(2)	C25	C33	C34	120.43(19)
C15	C14	N1	123.7(2)	C35	C34	C33	120.4(2)
C14	C15	C8	119.3(2)	C34	C35	C27	121.3(2)
C17	C16	C10	120.0(2)	C37	C36	C26	121.2(2)
C16	C17	C7	120.6(2)	C36	C37	C29	120.2(2)
C19	C18	C4	121.1(2)	C20	C38	C30	120.2(2)

(6) Torsion Angles for 3a.

A	B	C	D	Angle/°	A	B	C	D	Angle/°
O2	C33	C34	C35	179.3(2)	C18	C4	C5	C6	-0.5(3)
N1	C6	C7	C8	-0.3(3)	C18	C4	C5	C13	179.8(2)
N1	C6	C7	C17	179.7(2)	C19	C1	C2	C3	1.2(3)
N1	C14	C15	C8	-0.6(4)	C20	C21	C22	C23	-0.8(3)
N2	C24	C25	C26	-69.2(3)	C21	C20	C38	C30	0.5(4)
N2	C24	C25	C33	102.4(2)	C21	C22	C23	C24	-177.8(2)
C1	C2	C3	C4	-0.8(4)	C21	C22	C23	C30	0.7(3)
C1	C2	C3	C11	178.7(2)	C22	C23	C24	N2	177.0(2)
C2	C1	C19	C18	-0.3(3)	C22	C23	C24	C25	-3.0(3)
C2	C3	C4	C5	179.6(2)	C22	C23	C30	C31	-179.1(2)
C2	C3	C4	C18	-0.5(3)	C22	C23	C30	C38	-0.1(3)
C2	C3	C11	C12	-179.3(2)	C23	C24	C25	C26	110.8(2)
C3	C4	C5	C6	179.48(19)	C23	C24	C25	C33	-77.5(3)
C3	C4	C5	C13	-0.3(3)	C23	C30	C31	C32	1.8(3)
C3	C4	C18	C19	1.4(3)	C23	C30	C38	C20	-0.5(3)
C3	C11	C12	C13	-0.2(4)	C24	N2	C32	C31	-0.8(3)
C4	C3	C11	C12	0.2(3)	C24	C23	C30	C31	-0.5(3)
C4	C5	C6	N1	-76.0(3)	C24	C23	C30	C38	178.51(19)
C4	C5	C6	C7	102.8(2)	C24	C25	C26	C27	170.4(2)
C4	C5	C13	O1	178.4(2)	C24	C25	C26	C36	-9.6(3)
C4	C5	C13	C12	0.2(3)	C24	C25	C33	O2	9.1(3)
C4	C18	C19	C1	-1.0(4)	C24	C25	C33	C34	-167.44(19)
C5	C4	C18	C19	-178.7(2)	C25	C26	C27	C28	-179.6(2)
C5	C6	C7	C8	-179.05(19)	C25	C26	C27	C35	-1.8(3)
C5	C6	C7	C17	1.0(3)	C25	C26	C36	C37	179.1(2)
C6	N1	C14	C15	0.0(3)	C25	C33	C34	C35	-4.2(3)
C6	C5	C13	O1	-1.3(3)	C26	C25	C33	O2	-179.16(19)
C6	C5	C13	C12	-179.5(2)	C26	C25	C33	C34	4.3(3)
C6	C7	C8	C9	178.9(2)	C26	C27	C28	C29	0.8(3)

C6	C7	C8	C15	-0.2(3)	C26	C27	C35	C34	1.9(3)
C6	C7	C17	C16	-178.7(2)	C26	C36	C37	C29	0.2(4)
C7	C8	C9	C10	0.0(4)	C27	C26	C36	C37	-0.9(3)
C7	C8	C15	C14	0.7(4)	C27	C28	C29	C37	-1.5(4)
C8	C7	C17	C16	1.4(3)	C28	C27	C35	C34	179.7(2)
C8	C9	C10	C16	0.8(4)	C28	C29	C37	C36	1.0(4)
C9	C8	C15	C14	-178.5(2)	C30	C23	C24	N2	-1.5(3)
C9	C10	C16	C17	-0.5(4)	C30	C23	C24	C25	178.44(19)
C10	C16	C17	C7	-0.6(4)	C30	C31	C32	N2	-1.2(3)
C11	C3	C4	C5	0.1(3)	C31	C30	C38	C20	178.5(2)
C11	C3	C4	C18	-180.0(2)	C32	N2	C24	C23	2.2(3)
C11	C12	C13	O1	-178.1(2)	C32	N2	C24	C25	-177.75(19)
C11	C12	C13	C5	0.0(3)	C33	C25	C26	C27	-1.3(3)
C13	C5	C6	N1	103.8(2)	C33	C25	C26	C36	178.7(2)
C13	C5	C6	C7	-77.4(3)	C33	C34	C35	C27	1.1(3)
C14	N1	C6	C5	179.22(19)	C35	C27	C28	C29	-177.0(2)
C14	N1	C6	C7	0.5(3)	C36	C26	C27	C28	0.4(3)
C15	C8	C9	C10	179.2(2)	C36	C26	C27	C35	178.2(2)
C17	C7	C8	C9	-1.1(3)	C38	C20	C21	C22	0.2(4)
C17	C7	C8	C15	179.7(2)	C38	C30	C31	C32	-177.2(2)

(7) Hydrogen Atom Coordinates (Å×10^4) and Isotropic Displacement Parameters (Å^2×10^3) for 3a.

**Atom**	***x***	***y***	***z***	**U(eq)**
H1	3110(30)	4330(20)	6822(12)	28
H2A	4100(30)	2210(20)	8893(11)	31
H1A	10944	4268	5987	28
H2	9640	3637	6760	26
H9	2419	5304	3875	31
H10	2331	3478	3869	36
H11	7384	3361	7235	24
H12	5047	3576	7243	23
H14	4475	7777	5105	25
H15	3248	6997	4363	26
H16	3376	2490	4625	32
H17	4532	3341	5379	26
H18	7540	5496	5220	23
H19	9879	5209	5215	27
H20	-2672	2756	8925	32

H21	-1179	1319	8795	28
H22	862	1526	8278	22
H28	5100	452	6292	28
H29	3583	845	5547	31
H31	-550	5548	7951	26
H32	1400	5684	7391	24
H34	5626	1452	8237	22
H35	5917	725	7318	24
H36	1320	2633	6658	23
H37	1663	1920	5734	30
H38	-2154	4396	8538	29

Compound (*R*)-4a: The crystal structure of compound (*R*)-**4a** has been deposited at the Cambridge Crystallographic Data Centre (**CCDC 2046559**). The data is available free of charge at www.ccdc.cam.ac.uk/conts/retrieving.html.

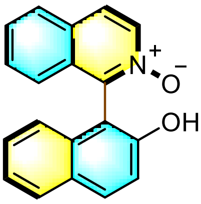

(*R*)-**4a**
CCDC 2046559

≡

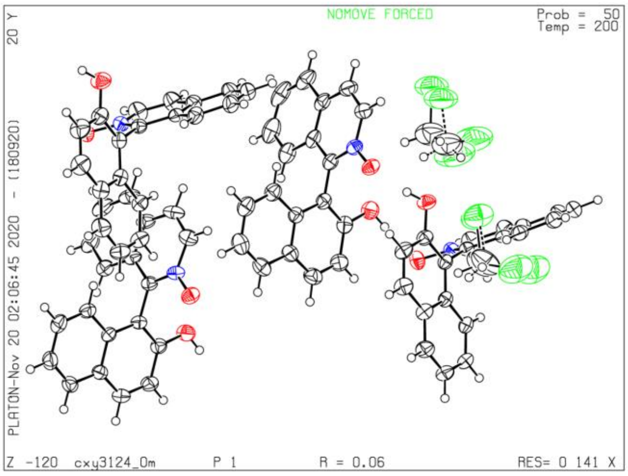

Table 1 Crystal data and structure refinement for (*R*)-4a.

Identification code	(*R*)-**4a**
Empirical formula	$C_{19.5}H_{14}ClNO_2$
Formula weight	329.77
Temperature/K	200
Crystal system	triclinic
Space group	P1
a/Å	11.4046(2)
b/Å	11.9273(2)
c/Å	12.4044(2)
α/°	88.3110(10)
β/°	84.8890(10)
γ/°	72.5780(10)
Volume/Å^3	1603.51(5)
Z	4
ρ_{calc}g/cm^3	1.366
μ/mm^{-1}	2.191
F(000)	684.0
Crystal size/mm^3	0.32 × 0.28 × 0.19
Radiation	CuKα (λ = 1.54178)
2Θ range for data collection/°	7.154 to 137.462
Index ranges	$-13 \leq h \leq 13$, $-14 \leq k \leq 14$, $-13 \leq l \leq 14$
Reflections collected	41596
Independent reflections	11400 [R_{int} = 0.0356, R_{sigma} = 0.0296]
Data/restraints/parameters	11400/37/871

Goodness-of-fit on F^2	1.067
Final R indexes [I>=2σ (I)]	R_1 = 0.0578, wR_2 = 0.1664
Final R indexes [all data]	R_1 = 0.0614, wR_2 = 0.1704
Largest diff. peak/hole / e Å^{-3}	0.82/-0.53
Flack parameter	0.016(12)

Table 2 Fractional Atomic Coordinates (×10^4) and Equivalent Isotropic Displacement Parameters (Å^2×10^3) for (*R*)-4a. U_{eq} is defined as 1/3 of of the trace of the orthogonalised U_{IJ} tensor.

Atom	*x*	*y*	*z*	U(eq)
O1	7321(3)	2230(3)	6625(2)	38.3(6)
O2	4733(3)	3078(3)	8655(3)	45.2(7)
N1	7547(3)	2778(3)	7456(3)	33.0(7)
C14	8592(5)	-2017(4)	8973(4)	50.9(11)
C13	9231(4)	-1182(4)	8809(4)	47.7(11)
C12	8609(4)	-14(4)	8694(4)	41.4(9)
C11	7299(4)	379(3)	8744(3)	33.1(8)
C6	6617(4)	1586(4)	8650(3)	33.2(8)
C5	7254(3)	2499(3)	8481(3)	33.5(8)
C16	7554(4)	3097(4)	9341(3)	36.3(9)
C23	7281(4)	2810(4)	10432(4)	46.1(10)
C22	7560(5)	3411(5)	11249(4)	60.3(14)
C21	8087(5)	4324(5)	11022(5)	61.3(15)
C15	7333(5)	-1670(4)	9023(4)	46.2(10)
C10	6647(4)	-474(4)	8908(3)	38.2(9)
C19	8109(4)	3641(4)	7216(4)	41.0(9)
C18	8379(4)	4253(4)	8015(4)	44.7(10)
C17	8100(4)	3998(4)	9117(4)	40.2(10)
C20	8361(4)	4612(4)	9980(5)	51.5(12)
C7	5346(4)	1916(4)	8713(3)	36.6(9)
C8	4708(4)	1068(4)	8862(4)	44.0(10)
C9	5346(4)	-92(4)	8944(4)	46.4(10)
O4	4837(3)	-1801(2)	13592(3)	44.7(7)
O3	7206(3)	-2669(3)	11518(2)	40.7(7)
N2	7502(3)	-2073(3)	12275(3)	32.9(7)
C40	8405(4)	-416(4)	15659(4)	48.6(12)
C39	8577(4)	-161(4)	14591(4)	46.0(11)
C36	8237(4)	-788(4)	13779(4)	37.4(9)
C35	7724(3)	-1711(3)	14115(3)	31.0(8)

Table 2 Fractional Atomic Coordinates (×10^4) and Equivalent Isotropic Displacement Parameters (Å^2×10^3) for (*R*)-4a. U_{eq} is defined as 1/3 of of the trace of the orthogonalised U_{IJ} tensor.

**Atom**	***x***	***y***	***z***	**U(eq)**
C24	7345(3)	-2329(3)	13325(3)	31.2(8)
C25	6743(3)	-3258(3)	13621(3)	30.2(8)
C30	7460(3)	-4437(3)	13800(3)	29.9(8)
C29	6839(4)	-5296(3)	14077(3)	32.3(8)
C34	7556(4)	-6466(4)	14251(3)	38.2(9)
C33	8821(4)	-6784(4)	14154(4)	43.0(10)
C41	7926(4)	-1333(4)	15977(4)	44.8(10)
C42	7581(4)	-1963(4)	15227(4)	37.6(9)
C31	8767(4)	-4791(4)	13709(3)	37.1(9)
C32	9424(4)	-5936(4)	13889(4)	42.6(10)
C28	5544(4)	-4942(4)	14156(4)	39.1(9)
C27	4870(4)	-3807(4)	13996(4)	39.7(9)
C26	5480(4)	-2944(3)	13722(3)	34.1(8)
C38	8000(4)	-1183(4)	11936(4)	42.9(10)
C37	8358(4)	-557(4)	12665(4)	45.2(10)
O6	5004(3)	3132(3)	6181(3)	41.5(7)
O5	2417(3)	3405(3)	8253(2)	40.2(7)
N3	2081(3)	4138(3)	7447(3)	31.8(7)
C52	1428(4)	-25(4)	5840(4)	43.5(10)
C51	727(4)	1138(4)	6041(4)	40.1(9)
C50	1279(3)	1998(4)	6154(3)	33.7(8)
C49	2580(3)	1725(3)	6081(3)	30.4(8)
C44	3186(3)	2590(3)	6198(3)	30.5(8)
C43	2452(3)	3821(3)	6417(3)	29.9(8)
C54	2089(3)	4662(3)	5577(3)	32.0(8)
C61	2508(4)	4383(4)	4485(3)	39.6(9)
C60	2181(5)	5214(5)	3691(4)	50.0(11)
C59	1422(5)	6364(5)	3958(4)	53.4(13)
C48	3297(4)	538(4)	5877(3)	34.7(8)
C47	4596(4)	250(4)	5812(4)	41.5(9)
C46	5162(4)	1090(4)	5922(4)	39.9(9)
C45	4462(4)	2279(4)	6110(3)	34.3(8)
C57	1300(4)	5250(4)	7708(4)	42.7(10)
C56	919(4)	6071(4)	6933(4)	45.3(10)
C55	1324(4)	5805(4)	5834(4)	38.1(9)
C58	996(4)	6652(4)	4998(4)	48.3(11)

Table 2 Fractional Atomic Coordinates (×10^4) and Equivalent Isotropic Displacement Parameters (Å^2×10^3) for (*R*)-4a. U_{eq} is defined as 1/3 of of the trace of the orthogonalised U_{IJ} tensor.

**Atom**	***x***	***y***	***z***	**U(eq)**
C53	2688(4)	-325(4)	5753(4)	41.9(10)
O7	12542(3)	-1562(3)	13248(2)	39.4(6)
O8	14862(3)	-1803(3)	11130(3)	49.6(8)
N4	12127(3)	-802(3)	12478(3)	32.1(7)
C71	11262(5)	-4932(4)	10794(4)	47.9(11)
C72	12515(5)	-5220(4)	10664(4)	49.0(11)
C67	13135(4)	-4368(4)	10775(3)	38.4(9)
C68	12434(4)	-3189(3)	11025(3)	33.1(8)
C63	13050(4)	-2350(3)	11158(3)	31.4(8)
C62	12346(3)	-1108(3)	11434(3)	31.2(8)
C73	11912(3)	-250(3)	10624(3)	32.1(8)
C74	11214(4)	910(4)	10936(4)	38.2(9)
C77	10809(4)	1768(4)	10132(4)	47.1(11)
C78	11089(4)	1481(4)	9065(4)	49.8(11)
C70	10562(4)	-3770(4)	11023(4)	44.5(10)
C69	11130(4)	-2921(4)	11133(3)	35.6(8)
C66	14432(4)	-4660(4)	10677(4)	48.7(11)
C65	15017(4)	-3829(4)	10803(5)	51.1(12)
C64	14322(4)	-2654(4)	11032(4)	40.8(9)
C76	11451(5)	325(4)	12791(4)	46.8(11)
C75	10997(5)	1157(4)	12054(4)	49.4(11)
C79	11762(4)	340(4)	8754(4)	46.8(10)
C80	12172(4)	-520(4)	9514(3)	39.8(9)
Cl1	5517(5)	2034(5)	3224(4)	92.5(9)
Cl2	4546(5)	975(5)	1587(4)	139.3(15)
C1	4690(16)	1051(13)	2965(10)	92(4)
Cl3	5898(4)	6766(4)	7405(5)	132.0(18)
Cl4	3496(5)	6488(7)	7731(8)	184(3)
C2	5009(12)	5785(11)	7458(13)	106(4)
Cl5	5776(11)	1845(11)	2861(7)	92.5(9)
Cl6	3572(10)	1908(9)	1970(8)	139.3(15)
C3	4870(30)	930(20)	2610(20)	92(4)
Cl7	5810(12)	6378(12)	6864(16)	132.0(18)
Cl8	3389(15)	7161(19)	7420(20)	184(3)
C4	4530(30)	5850(30)	7080(40)	106(4)

Table 3 Anisotropic Displacement Parameters ($Å^2 \times 10^3$) for (*R*)-4a. The Anisotropic displacement factor exponent takes the form: $-2\pi^2[h^2a^{*2}U_{11}+2hka^*b^*U_{12}+\ldots]$.

Atom	U_{11}	U_{22}	U_{33}	U_{23}	U_{13}	U_{12}
O1	39.6(15)	46.6(16)	32.9(15)	-8.6(12)	-4.7(11)	-18.0(13)
O2	33.6(15)	40.8(16)	56.7(19)	0.5(13)	-2.9(13)	-4.8(12)
N1	31.1(16)	34.6(17)	33.7(18)	-5.5(13)	-2.6(13)	-10.1(13)
C14	56(3)	42(2)	47(3)	2.4(19)	-2(2)	-4(2)
C13	36(2)	50(3)	52(3)	-5(2)	-6.9(18)	-3.9(19)
C12	36(2)	41(2)	45(2)	-4.0(17)	-4.9(17)	-7.0(17)
C11	34(2)	37(2)	28.6(19)	-0.4(15)	-1.6(14)	-10.2(16)
C6	33.2(19)	40(2)	26.7(18)	-3.0(15)	-2.8(14)	-10.9(16)
C5	28.8(18)	37(2)	33(2)	-5.0(15)	-3.3(14)	-6.1(15)
C16	29.2(19)	39(2)	36(2)	-8.2(16)	-8.4(15)	-1.1(15)
C23	45(2)	54(3)	36(2)	-8.3(18)	-6.2(18)	-6.8(19)
C22	55(3)	79(4)	39(3)	-14(2)	-11(2)	-4(3)
C21	46(3)	70(3)	59(3)	-34(3)	-22(2)	5(2)
C15	58(3)	43(2)	37(2)	4.0(17)	2.5(19)	-17(2)
C10	40(2)	40(2)	33(2)	1.4(16)	1.5(16)	-12.0(18)
C19	38(2)	41(2)	47(3)	-2.3(18)	-4.2(17)	-15.4(17)
C18	41(2)	44(2)	55(3)	-2.8(19)	-9.6(19)	-18.9(18)
C17	29.4(19)	35(2)	53(3)	-8.9(17)	-13.4(17)	-1.2(15)
C20	43(2)	44(2)	66(3)	-24(2)	-20(2)	-4.1(18)
C7	32(2)	41(2)	33(2)	-0.7(16)	0.7(15)	-6.2(16)
C8	34(2)	50(2)	46(2)	4.0(19)	1.6(18)	-12.9(19)
C9	44(2)	49(2)	49(3)	7.5(19)	2.0(19)	-19(2)
O4	33.8(15)	33.3(15)	65(2)	1.3(13)	-0.7(14)	-7.5(12)
O3	40.2(16)	48.6(17)	35.8(16)	-6.9(12)	-2.2(12)	-16.6(13)
N2	30.9(16)	34.4(16)	35.9(18)	-6.8(13)	3.7(13)	-14.7(13)
C40	36(2)	46(2)	63(3)	-27(2)	-7(2)	-7.7(18)
C39	36(2)	35(2)	70(3)	-18(2)	-2(2)	-13.6(17)
C36	26.4(18)	33.0(19)	53(3)	-9.1(17)	3.1(16)	-9.5(15)
C35	23.2(16)	28.2(18)	42(2)	-10.6(15)	-1.1(14)	-7.8(14)
C24	27.8(18)	30.2(18)	36(2)	-3.7(15)	0.5(15)	-9.6(15)
C25	31.3(18)	30.3(18)	31.6(19)	-2.8(14)	-0.8(14)	-13.6(15)
C30	30.7(18)	32.4(18)	28.0(18)	-3.7(14)	-5.1(14)	-10.7(15)
C29	39(2)	34.5(19)	26.7(18)	-1.3(14)	-2.6(14)	-16.0(16)
C34	50(2)	37(2)	32(2)	3.2(15)	-7.4(17)	-18.1(18)
C33	49(2)	34(2)	44(2)	2.3(17)	-14.0(19)	-7.5(18)
C41	38(2)	48(2)	47(3)	-14.7(19)	-8.9(18)	-7.1(18)
C42	35(2)	34.7(19)	43(2)	-6.5(16)	-3.9(16)	-8.7(16)

Table 3 Anisotropic Displacement Parameters (Å^2×10^3) for (*R*)-4a. The Anisotropic displacement factor exponent takes the form: -2π^2[h^{2}a*^{2}U$_{11}$+2hka*b*U$_{12}$+…].

Atom	U_{11}	U_{22}	U_{33}	U_{23}	U_{13}	U_{12}
C31	38(2)	36(2)	41(2)	-2.8(16)	-8.2(16)	-15.7(17)
C32	35(2)	42(2)	48(2)	-4.6(18)	-10.7(17)	-3.8(17)
C28	37(2)	41(2)	45(2)	1.5(17)	-0.1(17)	-20.5(17)
C27	27.9(19)	45(2)	48(2)	1.9(18)	0.9(17)	-13.9(17)
C26	28.7(19)	34.3(19)	38(2)	-2.2(15)	-0.9(15)	-8.0(15)
C38	45(2)	41(2)	43(2)	-0.3(17)	5.5(18)	-16.8(18)
C37	42(2)	35(2)	63(3)	-5.0(19)	8(2)	-21.8(18)
O6	29.5(14)	42.9(16)	53.9(18)	-3.1(13)	0.1(13)	-14.0(12)
O5	42.8(16)	41.8(15)	32.2(15)	4.5(12)	-1.8(12)	-7.8(12)
N3	28.5(15)	31.1(15)	34.7(17)	1.2(12)	-0.8(12)	-7.9(12)
C52	44(2)	47(2)	47(3)	-3.4(19)	-11.2(19)	-22.8(19)
C51	33(2)	46(2)	44(2)	1.0(18)	-12.3(17)	-15.4(18)
C50	26.3(18)	37.0(19)	35(2)	-1.0(15)	-5.1(15)	-4.8(15)
C49	30.1(19)	36.0(19)	24.5(17)	-2.7(14)	-2.0(14)	-8.7(15)
C44	25.6(18)	33.7(19)	30.8(19)	-1.6(14)	-1.5(14)	-6.9(15)
C43	25.5(17)	32.0(19)	33.0(19)	-1.9(14)	-2.3(14)	-9.4(15)
C54	23.8(17)	37.1(19)	39(2)	3.7(15)	-5.6(14)	-15.0(15)
C61	37(2)	51(2)	37(2)	0.6(17)	-4.1(16)	-20.9(19)
C60	50(3)	72(3)	38(2)	13(2)	-9.9(19)	-33(2)
C59	55(3)	55(3)	61(3)	26(2)	-23(2)	-29(2)
C48	31(2)	38(2)	34(2)	-8.2(16)	0.6(15)	-7.9(16)
C47	35(2)	36(2)	50(3)	-10.5(17)	1.8(17)	-5.5(17)
C46	22.9(18)	44(2)	48(2)	-7.6(18)	1.4(16)	-4.3(16)
C45	27.1(19)	40(2)	35(2)	-2.1(16)	0.8(15)	-10.4(16)
C57	44(2)	37(2)	43(2)	-7.3(17)	1.9(18)	-6.0(18)
C56	46(2)	36(2)	48(3)	-1.2(18)	2.9(19)	-5.5(18)
C55	34(2)	32.3(19)	50(2)	3.5(17)	-7.9(17)	-12.1(16)
C58	44(2)	38(2)	67(3)	14(2)	-17(2)	-16.7(19)
C53	47(2)	37(2)	44(2)	-10.8(17)	-4.0(18)	-14.0(18)
O7	41.3(16)	39.2(15)	35.3(15)	4.8(12)	-4.8(12)	-8.7(12)
O8	31.7(15)	49.2(18)	67(2)	-2.5(15)	1.7(14)	-12.5(13)
N4	33.0(16)	30.0(15)	33.2(17)	0.0(12)	-1.1(13)	-9.9(13)
C71	54(3)	44(2)	52(3)	-10(2)	-7(2)	-24(2)
C72	62(3)	35(2)	49(3)	-10.5(18)	-5(2)	-12(2)
C67	40(2)	35(2)	36(2)	-8.1(16)	-0.7(16)	-4.7(17)
C68	31(2)	37(2)	28.3(19)	-2.6(15)	-1.5(14)	-5.8(16)
C63	31.0(19)	29.7(18)	29.9(19)	-2.7(14)	-1.1(14)	-3.6(15)

Table 3 Anisotropic Displacement Parameters ($Å^2\times10^3$) for (*R*)-4a. The Anisotropic displacement factor exponent takes the form: $-2\pi^2[h^2a^{*2}U_{11}+2hka^*b^*U_{12}+…]$.

Atom	U_{11}	U_{22}	U_{33}	U_{23}	U_{13}	U_{12}
C62	26.8(18)	32.8(19)	33.4(19)	-1.6(15)	0.7(14)	-8.9(15)
C73	25.9(18)	34.0(19)	39(2)	2.6(15)	-4.5(14)	-11.8(15)
C74	37(2)	31.8(19)	46(2)	0.7(17)	-6.0(17)	-10.4(16)
C77	44(2)	33(2)	64(3)	9.1(19)	-11(2)	-10.5(18)
C78	44(2)	54(3)	54(3)	22(2)	-14(2)	-19(2)
C70	41(2)	49(2)	48(3)	-4.6(19)	-7.0(19)	-18.0(19)
C69	32(2)	35(2)	38(2)	-5.4(15)	-2.9(16)	-6.6(16)
C66	40(2)	42(2)	56(3)	-13(2)	3(2)	0.1(19)
C65	29(2)	51(3)	66(3)	-8(2)	4(2)	-1.5(19)
C64	30(2)	45(2)	43(2)	-2.7(18)	-0.8(17)	-6.9(17)
C76	58(3)	37(2)	41(2)	-10.2(18)	1(2)	-8(2)
C75	60(3)	32(2)	49(3)	-6.6(19)	2(2)	-5.1(19)
C79	45(2)	61(3)	37(2)	10.8(19)	-8.0(18)	-20(2)
C80	34(2)	47(2)	37(2)	0.2(17)	-3.5(16)	-9.9(17)
Cl1	101(3)	120(3)	70(3)	-27(2)	7.5(19)	-55(2)
Cl2	157(4)	162(4)	125(3)	-33(2)	-25(2)	-80(3)
C1	141(8)	62(5)	72(9)	-11(6)	20(7)	-36(5)
Cl3	90.4(17)	109(3)	214(6)	14(3)	-8(3)	-58.6(18)
Cl4	91(2)	174(6)	306(8)	-3(6)	-8(3)	-68(4)
C2	115(8)	83(5)	135(9)	-17(6)	-13(6)	-50(6)
Cl5	101(3)	120(3)	70(3)	-27(2)	7.5(19)	-55(2)
Cl6	157(4)	162(4)	125(3)	-33(2)	-25(2)	-80(3)
C3	141(8)	62(5)	72(9)	-11(6)	20(7)	-36(5)
Cl7	90.4(17)	109(3)	214(6)	14(3)	-8(3)	-58.6(18)
Cl8	91(2)	174(6)	306(8)	-3(6)	-8(3)	-68(4)
C4	115(8)	83(5)	135(9)	-17(6)	-13(6)	-50(6)

Table 4 Bond Lengths for (*R*)-4a.

Atom	Atom	Length/Å	Atom	Atom	Length/Å
O1	N1	1.321(4)	C52	C51	1.395(7)
O2	C7	1.355(5)	C52	C53	1.368(7)
N1	C5	1.343(5)	C51	C50	1.371(6)
N1	C19	1.379(5)	C50	C49	1.416(5)
C14	C13	1.401(7)	C49	C44	1.421(5)
C14	C15	1.366(8)	C49	C48	1.424(5)
C13	C12	1.371(6)	C44	C43	1.476(5)

Table 4 Bond Lengths for (*R*)-4a.

Atom	Atom	Length/Å	Atom	Atom	Length/Å
C12	C11	1.421(6)	C44	C45	1.384(5)
C11	C6	1.423(6)	C43	C54	1.425(5)
C11	C10	1.430(6)	C54	C61	1.409(6)
C6	C5	1.480(6)	C54	C55	1.409(6)
C6	C7	1.380(6)	C61	C60	1.371(6)
C5	C16	1.421(5)	C60	C59	1.416(8)
C16	C23	1.416(7)	C59	C58	1.353(8)
C16	C17	1.404(6)	C48	C47	1.413(6)
C23	C22	1.371(7)	C48	C53	1.422(6)
C22	C21	1.404(9)	C47	C46	1.360(6)
C21	C20	1.361(9)	C46	C45	1.418(6)
C15	C10	1.416(6)	C57	C56	1.354(7)
C10	C9	1.414(6)	C56	C55	1.414(7)
C19	C18	1.357(6)	C55	C58	1.420(6)
C18	C17	1.420(7)	O7	N4	1.316(4)
C17	C20	1.415(6)	O8	C64	1.348(6)
C7	C8	1.411(6)	N4	C62	1.337(5)
C8	C9	1.363(7)	N4	C76	1.381(5)
O4	C26	1.354(5)	C71	C72	1.362(7)
O3	N2	1.319(4)	C71	C70	1.400(7)
N2	C24	1.338(5)	C72	C67	1.417(7)
N2	C38	1.384(5)	C67	C68	1.422(5)
C40	C39	1.361(8)	C67	C66	1.409(6)
C40	C41	1.394(7)	C68	C63	1.405(6)
C39	C36	1.418(6)	C68	C69	1.418(6)
C36	C35	1.430(5)	C63	C62	1.491(5)
C36	C37	1.404(7)	C63	C64	1.381(6)
C35	C24	1.414(5)	C62	C73	1.423(5)
C35	C42	1.408(6)	C73	C74	1.420(6)
C24	C25	1.490(5)	C73	C80	1.409(6)
C25	C30	1.422(5)	C74	C77	1.413(6)
C25	C26	1.370(5)	C74	C75	1.411(7)
C30	C29	1.427(5)	C77	C78	1.363(8)
C30	C31	1.417(6)	C78	C79	1.394(7)
C29	C34	1.412(6)	C70	C69	1.372(6)
C29	C28	1.405(6)	C66	C65	1.369(7)
C34	C33	1.373(7)	C65	C64	1.412(6)
C33	C32	1.400(7)	C76	C75	1.345(7)

Table 4 Bond Lengths for (*R*)-4a.

Atom	Atom	Length/Å	Atom	Atom	Length/Å
C41	C42	1.368(6)	C79	C80	1.376(6)
C31	C32	1.370(6)	Cl1	C1	1.762(15)
C28	C27	1.360(6)	Cl2	C1	1.740(13)
C27	C26	1.423(6)	Cl3	C2	1.759(11)
C38	C37	1.353(7)	Cl4	C2	1.686(14)
O6	C45	1.348(5)	Cl5	C3	1.76(2)
O5	N3	1.315(4)	Cl6	C3	1.82(3)
N3	C43	1.338(5)	Cl7	C4	1.75(2)
N3	C57	1.387(5)	Cl8	C4	1.75(3)

Table 5 Bond Angles for (*R*)-4a.

Atom	Atom	Atom	Angle/°	Atom	Atom	Atom	Angle/°
O1	N1	C5	121.6(3)	C43	N3	C57	121.3(3)
O1	N1	C19	116.5(3)	C53	C52	C51	120.1(4)
C5	N1	C19	121.9(3)	C50	C51	C52	121.1(4)
C15	C14	C13	120.0(4)	C51	C50	C49	120.7(4)
C12	C13	C14	120.8(4)	C50	C49	C44	122.4(3)
C13	C12	C11	120.8(4)	C50	C49	C48	118.3(3)
C12	C11	C6	122.6(4)	C44	C49	C48	119.3(3)
C12	C11	C10	118.4(4)	C49	C44	C43	119.8(3)
C6	C11	C10	119.0(4)	C45	C44	C49	120.1(3)
C11	C6	C5	120.9(3)	C45	C44	C43	120.1(3)
C7	C6	C11	120.0(4)	N3	C43	C44	118.1(3)
C7	C6	C5	119.1(4)	N3	C43	C54	119.3(3)
N1	C5	C6	117.6(3)	C54	C43	C44	122.6(3)
N1	C5	C16	118.9(4)	C61	C54	C43	121.2(4)
C16	C5	C6	123.5(4)	C55	C54	C43	119.9(4)
C23	C16	C5	120.5(4)	C55	C54	C61	118.9(4)
C17	C16	C5	120.2(4)	C60	C61	C54	120.4(4)
C17	C16	C23	119.3(4)	C61	C60	C59	120.3(4)
C22	C23	C16	119.5(5)	C58	C59	C60	120.5(4)
C23	C22	C21	121.0(5)	C47	C48	C49	119.0(4)
C20	C21	C22	120.5(4)	C47	C48	C53	121.8(4)
C14	C15	C10	121.4(4)	C53	C48	C49	119.2(4)
C15	C10	C11	118.6(4)	C46	C47	C48	120.9(4)
C9	C10	C11	118.7(4)	C47	C46	C45	120.8(4)
C9	C10	C15	122.7(4)	O6	C45	C44	118.4(3)

Table 5 Bond Angles for (*R*)-4a.

Atom	Atom	Atom	Angle/°	Atom	Atom	Atom	Angle/°
C18	C19	N1	120.9(4)	O6	C45	C46	121.8(3)
C19	C18	C17	120.1(4)	C44	C45	C46	119.8(4)
C16	C17	C18	117.9(4)	C56	C57	N3	121.3(4)
C16	C17	C20	119.7(4)	C57	C56	C55	120.0(4)
C20	C17	C18	122.3(5)	C54	C55	C56	118.1(4)
C21	C20	C17	120.0(5)	C54	C55	C58	119.7(4)
O2	C7	C6	118.0(4)	C56	C55	C58	122.2(4)
O2	C7	C8	121.3(4)	C59	C58	C55	120.1(4)
C6	C7	C8	120.7(4)	C52	C53	C48	120.6(4)
C9	C8	C7	120.1(4)	O7	N4	C62	121.4(3)
C8	C9	C10	121.4(4)	O7	N4	C76	117.3(3)
O3	N2	C24	121.4(3)	C62	N4	C76	121.3(3)
O3	N2	C38	117.2(3)	C72	C71	C70	119.9(4)
C24	N2	C38	121.4(3)	C71	C72	C67	121.3(4)
C39	C40	C41	120.4(4)	C72	C67	C68	119.3(4)
C40	C39	C36	121.2(4)	C66	C67	C72	122.0(4)
C39	C36	C35	118.0(4)	C66	C67	C68	118.7(4)
C37	C36	C39	124.4(4)	C63	C68	C67	119.3(4)
C37	C36	C35	117.6(4)	C63	C68	C69	123.1(3)
C24	C35	C36	119.1(4)	C69	C68	C67	117.6(4)
C42	C35	C36	119.1(3)	C68	C63	C62	120.7(3)
C42	C35	C24	121.7(3)	C64	C63	C68	120.8(4)
N2	C24	C35	120.1(3)	C64	C63	C62	118.4(4)
N2	C24	C25	117.8(3)	N4	C62	C63	118.3(3)
C35	C24	C25	122.0(3)	N4	C62	C73	119.6(3)
C30	C25	C24	120.8(3)	C73	C62	C63	122.1(3)
C26	C25	C24	118.3(3)	C74	C73	C62	119.5(4)
C26	C25	C30	120.9(3)	C80	C73	C62	121.5(4)
C25	C30	C29	118.7(3)	C80	C73	C74	118.9(4)
C31	C30	C25	122.4(3)	C77	C74	C73	119.5(4)
C31	C30	C29	118.9(3)	C75	C74	C73	117.4(4)
C34	C29	C30	118.5(4)	C75	C74	C77	123.0(4)
C28	C29	C30	118.6(3)	C78	C77	C74	120.1(4)
C28	C29	C34	122.9(4)	C77	C78	C79	120.6(4)
C33	C34	C29	121.4(4)	C69	C70	C71	120.4(4)
C34	C33	C32	119.9(4)	C70	C69	C68	121.5(4)
C42	C41	C40	120.7(5)	C65	C66	C67	121.3(4)
C41	C42	C35	120.6(4)	C66	C65	C64	120.1(4)

Table 5 Bond Angles for (*R*)-4a.

Atom	Atom	Atom	Angle/°	Atom	Atom	Atom	Angle/°
C32	C31	C30	120.6(4)	O8	C64	C63	118.3(4)
C31	C32	C33	120.8(4)	O8	C64	C65	122.0(4)
C27	C28	C29	122.0(4)	C63	C64	C65	119.8(4)
C28	C27	C26	119.8(4)	C75	C76	N4	121.1(4)
O4	C26	C25	118.7(3)	C76	C75	C74	120.9(4)
O4	C26	C27	121.3(3)	C80	C79	C78	121.0(4)
C25	C26	C27	120.0(4)	C79	C80	C73	119.9(4)
C37	C38	N2	120.5(4)	Cl2	C1	Cl1	111.1(8)
C38	C37	C36	121.2(4)	Cl4	C2	Cl3	111.5(7)
O5	N3	C43	121.6(3)	Cl5	C3	Cl6	103.9(15)
O5	N3	C57	117.1(3)	Cl8	C4	Cl7	99.3(15)

Table 6 Torsion Angles for (*R*)-4a.

A	B	C	D	Angle/°	A	B	C	D	Angle/°
O1	N1	C5	C6	-2.5(5)	O5	N3	C43	C44	4.2(5)
O1	N1	C5	C16	178.2(3)	O5	N3	C43	C54	-178.2(3)
O1	N1	C19	C18	-180.0(4)	O5	N3	C57	C56	179.0(4)
O2	C7	C8	C9	-178.4(4)	N3	C43	C54	C61	176.9(3)
N1	C5	C16	C23	-178.8(4)	N3	C43	C54	C55	-1.5(5)
N1	C5	C16	C17	2.7(5)	N3	C57	C56	C55	-0.5(7)
N1	C19	C18	C17	0.9(6)	C52	C51	C50	C49	-0.7(6)
C14	C13	C12	C11	0.4(7)	C51	C52	C53	C48	0.7(7)
C14	C15	C10	C11	-0.4(7)	C51	C50	C49	C44	-179.7(4)
C14	C15	C10	C9	179.2(4)	C51	C50	C49	C48	0.7(6)
C13	C14	C15	C10	0.2(7)	C50	C49	C44	C43	0.8(5)
C13	C12	C11	C6	178.7(4)	C50	C49	C44	C45	-179.4(4)
C13	C12	C11	C10	-0.5(6)	C50	C49	C48	C47	-179.5(4)
C12	C11	C6	C5	0.7(6)	C50	C49	C48	C53	0.1(6)
C12	C11	C6	C7	-179.5(4)	C49	C44	C43	N3	86.0(4)
C12	C11	C10	C15	0.5(6)	C49	C44	C43	C54	-91.5(4)
C12	C11	C10	C9	-179.1(4)	C49	C44	C45	O6	177.3(3)
C11	C6	C5	N1	90.4(4)	C49	C44	C45	C46	-1.1(6)
C11	C6	C5	C16	-90.4(5)	C49	C48	C47	C46	-1.1(7)
C11	C6	C7	O2	177.9(3)	C49	C48	C53	C52	-0.8(6)
C11	C6	C7	C8	-0.6(6)	C44	C49	C48	C47	0.9(6)
C11	C10	C9	C8	-2.3(7)	C44	C49	C48	C53	-179.5(4)
C6	C11	C10	C15	-178.7(4)	C44	C43	C54	C61	-5.6(5)

Table 6 Torsion Angles for (*R*)-4a.

A	B	C	D	Angle/°	A	B	C	D	Angle/°
C6	C11	C10	C9	1.6(6)	C44	C43	C54	C55	176.0(3)
C6	C5	C16	C23	2.0(6)	C43	N3	C57	C56	-2.3(6)
C6	C5	C16	C17	-176.6(3)	C43	C44	C45	O6	-2.9(5)
C6	C7	C8	C9	0.0(7)	C43	C44	C45	C46	178.7(4)
C5	N1	C19	C18	-0.6(6)	C43	C54	C61	C60	-177.9(4)
C5	C6	C7	O2	-2.3(6)	C43	C54	C55	C56	-1.2(5)
C5	C6	C7	C8	179.3(4)	C43	C54	C55	C58	178.0(4)
C5	C16	C23	C22	-178.9(4)	C54	C61	C60	C59	0.1(6)
C5	C16	C17	C18	-2.3(5)	C54	C55	C58	C59	-0.3(6)
C5	C16	C17	C20	178.1(4)	C61	C54	C55	C56	-179.6(4)
C16	C23	C22	C21	1.4(7)	C61	C54	C55	C58	-0.5(5)
C16	C17	C20	C21	0.3(6)	C61	C60	C59	C58	-0.9(7)
C23	C16	C17	C18	179.1(4)	C60	C59	C58	C55	0.9(7)
C23	C16	C17	C20	-0.5(6)	C48	C49	C44	C43	-179.6(3)
C23	C22	C21	C20	-1.6(7)	C48	C49	C44	C45	0.2(5)
C22	C21	C20	C17	0.8(7)	C48	C47	C46	C45	0.2(7)
C15	C14	C13	C12	-0.3(7)	C47	C48	C53	C52	178.8(4)
C15	C10	C9	C8	178.1(4)	C47	C46	C45	O6	-177.5(4)
C10	C11	C6	C5	179.9(4)	C47	C46	C45	C44	0.9(6)
C10	C11	C6	C7	-0.2(6)	C45	C44	C43	N3	-93.8(4)
C19	N1	C5	C6	178.1(3)	C45	C44	C43	C54	88.7(5)
C19	N1	C5	C16	-1.2(5)	C57	N3	C43	C44	-174.3(3)
C19	C18	C17	C16	0.5(6)	C57	N3	C43	C54	3.3(5)
C19	C18	C17	C20	-179.9(4)	C57	C56	C55	C54	2.2(6)
C18	C17	C20	C21	-179.3(4)	C57	C56	C55	C58	-177.0(4)
C17	C16	C23	C22	-0.4(6)	C56	C55	C58	C59	178.9(4)
C7	C6	C5	N1	-89.5(5)	C55	C54	C61	C60	0.5(6)
C7	C6	C5	C16	89.8(5)	C53	C52	C51	C50	0.0(7)
C7	C8	C9	C10	1.5(7)	C53	C48	C47	C46	179.3(4)
O3	N2	C24	C35	177.1(3)	O7	N4	C62	C63	1.5(5)
O3	N2	C24	C25	-4.2(5)	O7	N4	C62	C73	-178.1(3)
O3	N2	C38	C37	-178.1(4)	O7	N4	C76	C75	179.7(4)
N2	C24	C25	C30	96.1(4)	N4	C62	C73	C74	-2.2(5)
N2	C24	C25	C26	-84.2(4)	N4	C62	C73	C80	176.9(4)
N2	C38	C37	C36	0.0(7)	N4	C76	C75	C74	-1.0(8)
C40	C39	C36	C35	-0.9(6)	C71	C72	C67	C68	-0.1(7)
C40	C39	C36	C37	178.4(4)	C71	C72	C67	C66	178.0(5)
C40	C41	C42	C35	1.1(6)	C71	C70	C69	C68	-0.2(7)

Table 6 Torsion Angles for (*R*)-4a.

A	B	C	D	Angle/°	A	B	C	D	Angle/°
C39	C40	C41	C42	-2.2(6)	C72	C71	C70	C69	-1.0(7)
C39	C36	C35	C24	178.3(4)	C72	C67	C68	C63	178.7(4)
C39	C36	C35	C42	-0.3(5)	C72	C67	C68	C69	-1.1(6)
C39	C36	C37	C38	-179.2(4)	C72	C67	C66	C65	-179.0(5)
C36	C35	C24	N2	2.0(5)	C67	C68	C63	C62	-179.3(4)
C36	C35	C24	C25	-176.7(3)	C67	C68	C63	C64	1.0(6)
C36	C35	C42	C41	0.2(6)	C67	C68	C69	C70	1.3(6)
C35	C36	C37	C38	0.1(6)	C67	C66	C65	C64	-0.2(8)
C35	C24	C25	C30	-85.2(4)	C68	C67	C66	C65	-0.9(7)
C35	C24	C25	C26	94.5(5)	C68	C63	C62	N4	92.7(5)
C24	N2	C38	C37	0.9(6)	C68	C63	C62	C73	-87.7(5)
C24	C35	C42	C41	-178.3(4)	C68	C63	C64	O8	177.6(4)
C24	C25	C30	C29	-179.7(3)	C68	C63	C64	C65	-2.1(6)
C24	C25	C30	C31	0.0(5)	C63	C68	C69	C70	-178.5(4)
C24	C25	C26	O4	-2.7(6)	C63	C62	C73	C74	178.1(3)
C24	C25	C26	C27	179.6(4)	C63	C62	C73	C80	-2.7(6)
C25	C30	C29	C34	179.8(4)	C62	N4	C76	C75	-0.6(7)
C25	C30	C29	C28	0.3(5)	C62	C63	C64	O8	-2.2(6)
C25	C30	C31	C32	180.0(4)	C62	C63	C64	C65	178.1(4)
C30	C25	C26	O4	177.0(4)	C62	C73	C74	C77	178.5(4)
C30	C25	C26	C27	-0.8(6)	C62	C73	C74	C75	0.7(6)
C30	C29	C34	C33	-0.3(6)	C62	C73	C80	C79	-178.4(4)
C30	C29	C28	C27	-1.2(6)	C73	C74	C77	C78	-0.1(6)
C30	C31	C32	C33	0.8(6)	C73	C74	C75	C76	0.9(7)
C29	C30	C31	C32	-0.3(6)	C74	C73	C80	C79	0.8(6)
C29	C34	C33	C32	0.7(6)	C74	C77	C78	C79	0.8(7)
C29	C28	C27	C26	1.1(7)	C77	C74	C75	C76	-176.8(5)
C34	C29	C28	C27	179.3(4)	C77	C78	C79	C80	-0.7(7)
C34	C33	C32	C31	-0.9(7)	C78	C79	C80	C73	-0.1(7)
C41	C40	C39	C36	2.1(7)	C70	C71	C72	C67	1.2(8)
C42	C35	C24	N2	-179.5(3)	C69	C68	C63	C62	0.5(6)
C42	C35	C24	C25	1.8(6)	C69	C68	C63	C64	-179.3(4)
C31	C30	C29	C34	0.1(5)	C66	C67	C68	C63	0.5(6)
C31	C30	C29	C28	-179.4(4)	C66	C67	C68	C69	-179.2(4)
C28	C29	C34	C33	179.2(4)	C66	C65	C64	O8	-177.9(5)
C28	C27	C26	O4	-177.8(4)	C66	C65	C64	C63	1.7(8)
C28	C27	C26	C25	-0.1(6)	C64	C63	C62	N4	-87.5(5)
C26	C25	C30	C29	0.7(5)	C64	C63	C62	C73	92.1(5)

Table 6 Torsion Angles for (*R*)-4a.

A	B	C	D	Angle/°	A	B	C	D	Angle/°
C26	C25	C30	C31	-179.7(4)	C76	N4	C62	C63	-178.2(4)
C38	N2	C24	C35	-1.9(6)	C76	N4	C62	C73	2.2(6)
C38	N2	C24	C25	176.8(3)	C75	C74	C77	C78	177.6(5)
C37	C36	C35	C24	-1.1(5)	C80	C73	C74	C77	-0.7(6)
C37	C36	C35	C42	-179.6(4)	C80	C73	C74	C75	-178.5(4)

Table 7 Hydrogen Atom Coordinates (Å×10^4) and Isotropic Displacement Parameters (Å^2×10^3) for (*R*)-4a.

Atom	*x*	*y*	*z*	U(eq)
H2	3995.44	3164.9	8542.91	68
H14	9033.65	-2824.45	9048.6	61
H13	10106.52	-1427.57	8778.17	57
H12	9058.09	538.89	8578.55	50
H23	6907.23	2206.01	10595.84	55
H22	7393.66	3206.8	11980.54	72
H21	8253.94	4743.97	11599.04	74
H15	6908.57	-2243.63	9136.54	55
H19	8309.34	3810.13	6481.8	49
H18	8755.28	4852.69	7837.7	54
H20	8726.74	5224.18	9831.58	62
H8	3832.35	1307.25	8905.27	53
H9	4905.91	-655.57	9026.39	56
H4	4100.27	-1746.63	13505.47	67
H40	8613.47	32.31	16190.38	58
H39	8930.53	448.54	14385.23	55
H34	7154.33	-7045.5	14439.38	46
H33	9287.14	-7578.34	14266.13	52
H41	7838.26	-1521.14	16723.62	54
H42	7243.29	-2576.48	15457.24	45
H31	9190.79	-4228.81	13521.54	44
H32	10299.26	-6157.92	13833.25	51
H28	5125.75	-5512.19	14325.73	47
H27	3994.81	-3590.97	14067.74	48
H38	8090.41	-1011.06	11186.15	52
H37	8697.32	51.04	12418.44	54
H6	5695.41	2846.96	6426.1	62
H52	1029.88	-609.81	5764.26	52

Table 7 Hydrogen Atom Coordinates (Å×10^4) and Isotropic Displacement Parameters (Å^2×10^3) for (*R*)-4a.

Atom	*x*	*y*	*z*	U(eq)
H51	-147.68	1336.62	6100.28	48
H50	782.97	2785.85	6281.77	40
H61	3018.89	3614.16	4299.37	48
H60	2465.97	5018.2	2957.37	60
H59	1210.01	6937.87	3403.88	64
H47	5081.52	-542.14	5688.97	50
H46	6036.64	877.2	5872.35	48
H57	1027.56	5439.86	8444.62	51
H56	378.98	6825.3	7129	54
H58	477.12	7421.89	5168.89	58
H53	3160.48	-1116.61	5607.48	50
H8A	15578.58	-2103.66	11319.99	74
H71	10863.59	-5518.99	10729.5	57
H72	12983.06	-6007.97	10496.06	59
H77	10340.61	2545.4	10334.88	57
H78	10822.09	2063.65	8527.59	60
H70	9686.97	-3567.42	11101.77	53
H69	10640.53	-2136.34	11285.61	43
H66	14908.72	-5447.32	10520.24	58
H65	15892.51	-4044.71	10735.73	61
H76	11303.37	514.94	13538.59	56
H75	10523.3	1921.57	12290.73	59
H79	11941.35	153.08	8006.69	56
H80	12628.91	-1294.45	9289.98	48
H1A	5122.46	260.13	3237.28	111
H1B	3859.29	1314.44	3356.45	111
H2A	5134.96	5375.08	6755.33	127
H2B	5289.31	5185.85	8024.44	127
H3A	4589.15	607.88	3295.45	111
H3B	5335.25	278.76	2125.47	111
H4A	4371.57	5491.58	6416.35	127
H4B	4625.23	5267.25	7678.94	127

Table 8 Atomic Occupancy for cxy3124_0m.

Atom	*Occupancy*	Atom	*Occupancy*	Atom	*Occupancy*
Cl1	0.650(4)	Cl2	0.650(4)	C1	0.650(4)

Table 8 Atomic Occupancy for cxy3124_0m.

**Atom**	***Occupancy***	**Atom**	***Occupancy***	**Atom**	***Occupancy***
H1A	0.650(4)	H1B	0.650(4)	Cl3	0.740(8)
Cl4	0.740(8)	C2	0.740(8)	H2A	0.740(8)
H2B	0.740(8)	Cl5	0.350(4)	Cl6	0.350(4)
C3	0.350(4)	H3A	0.350(4)	H3B	0.350(4)
Cl7	0.260(8)	Cl8	0.260(8)	C4	0.260(8)
H4A	0.260(8)	H4B	0.260(8)		

10. Copies of ^{1}H and ^{13}C NMR spectra of related compounds

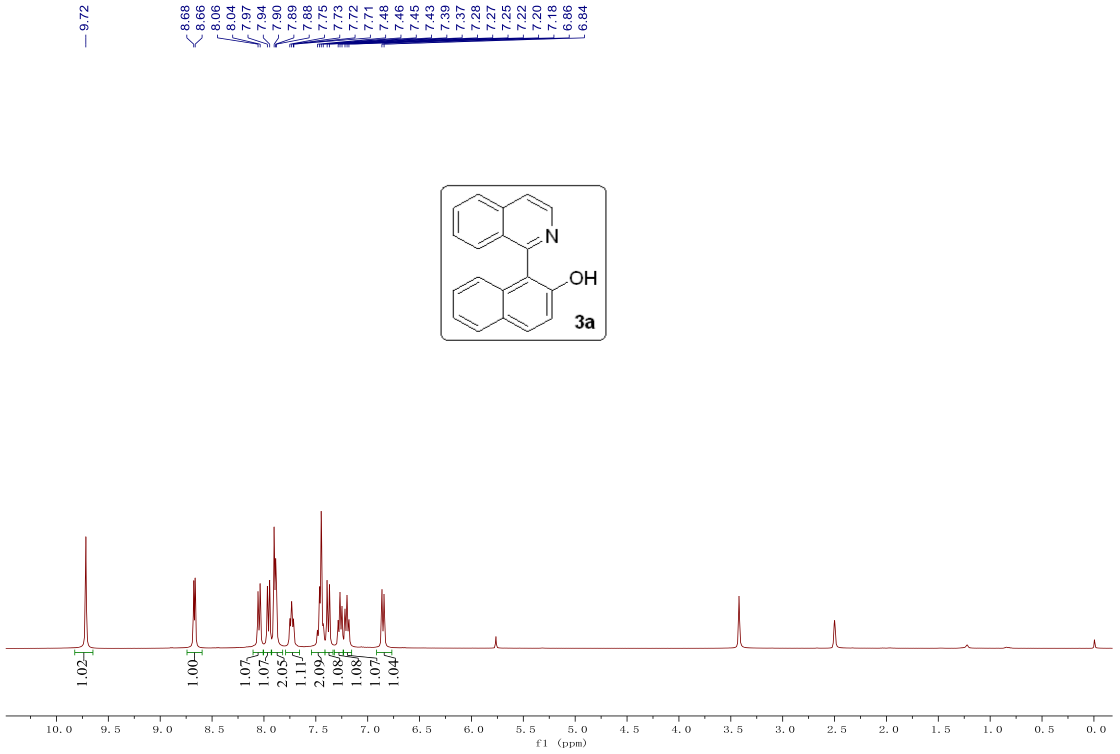

Supplementary Figure 1. ^{1}H NMR spectrum of **3a**

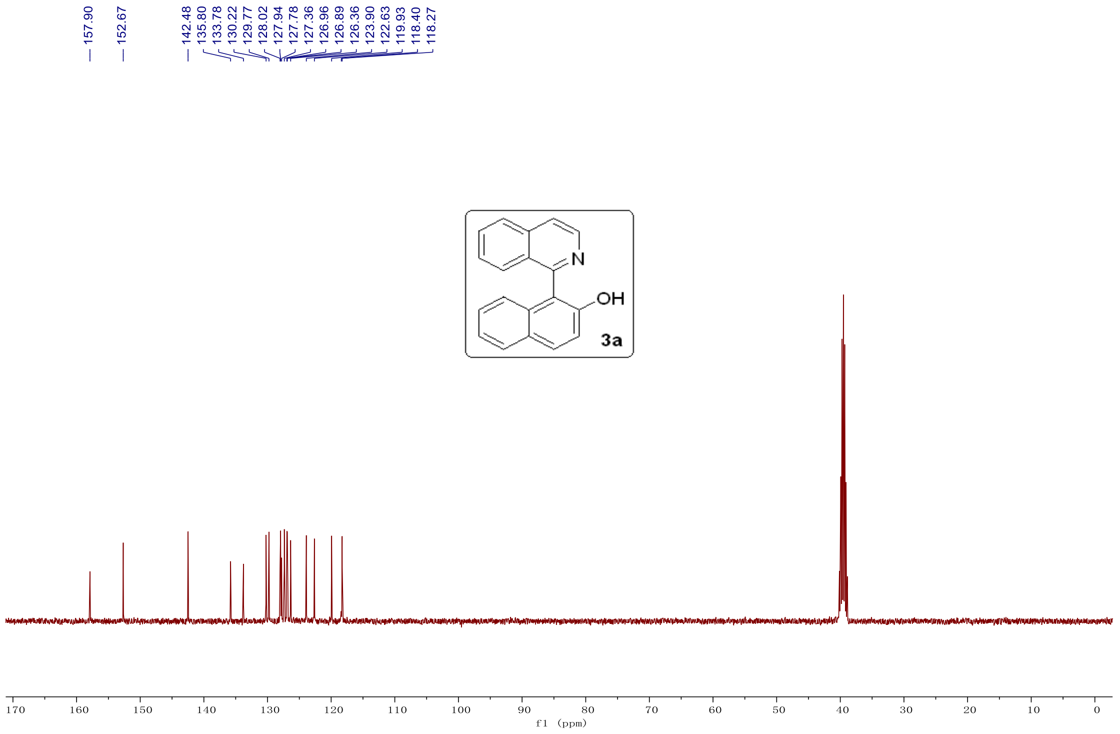

Supplementary Figure 2. ^{13}C NMR spectrum of **3a**

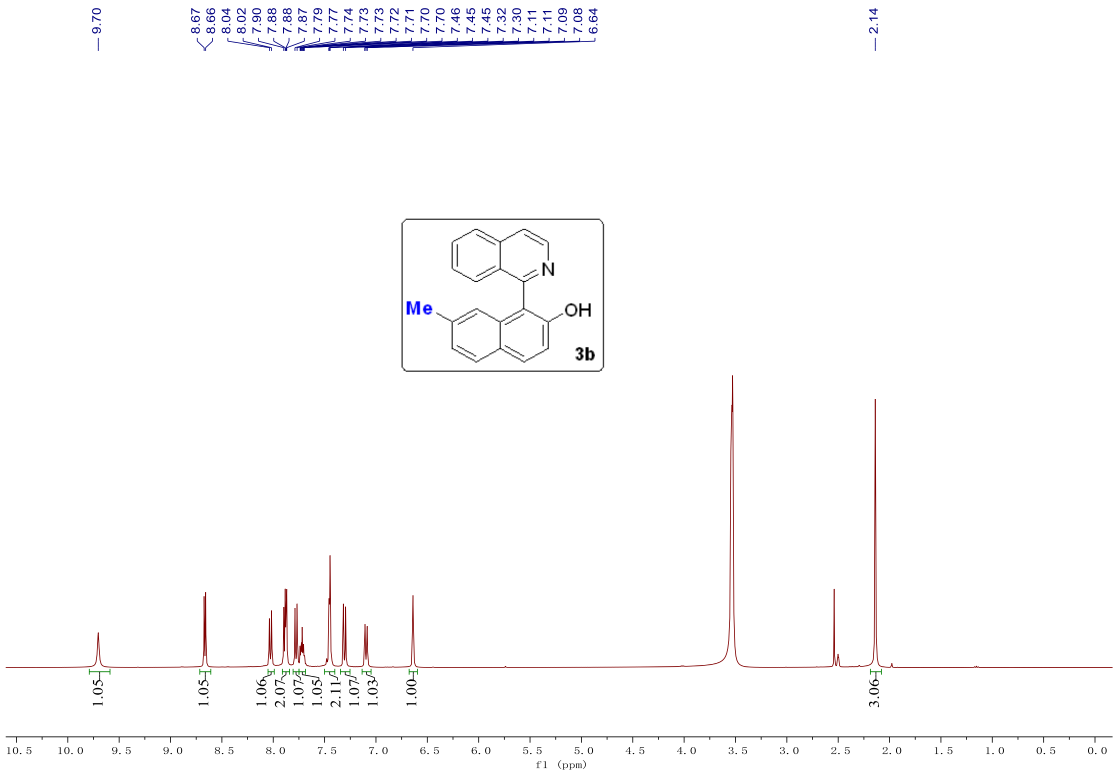

Supplementary Figure 3. ^{1}H NMR spectrum of **3b**

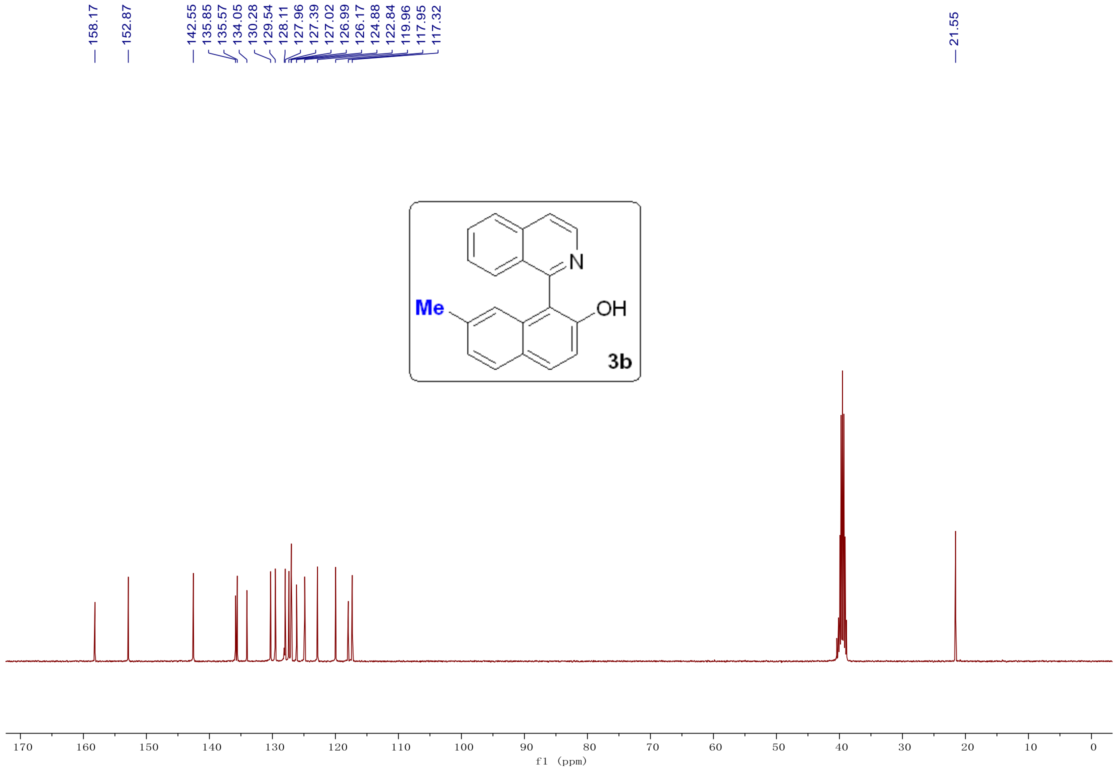

Supplementary Figure 4. ^{13}C NMR spectrum of **3b**

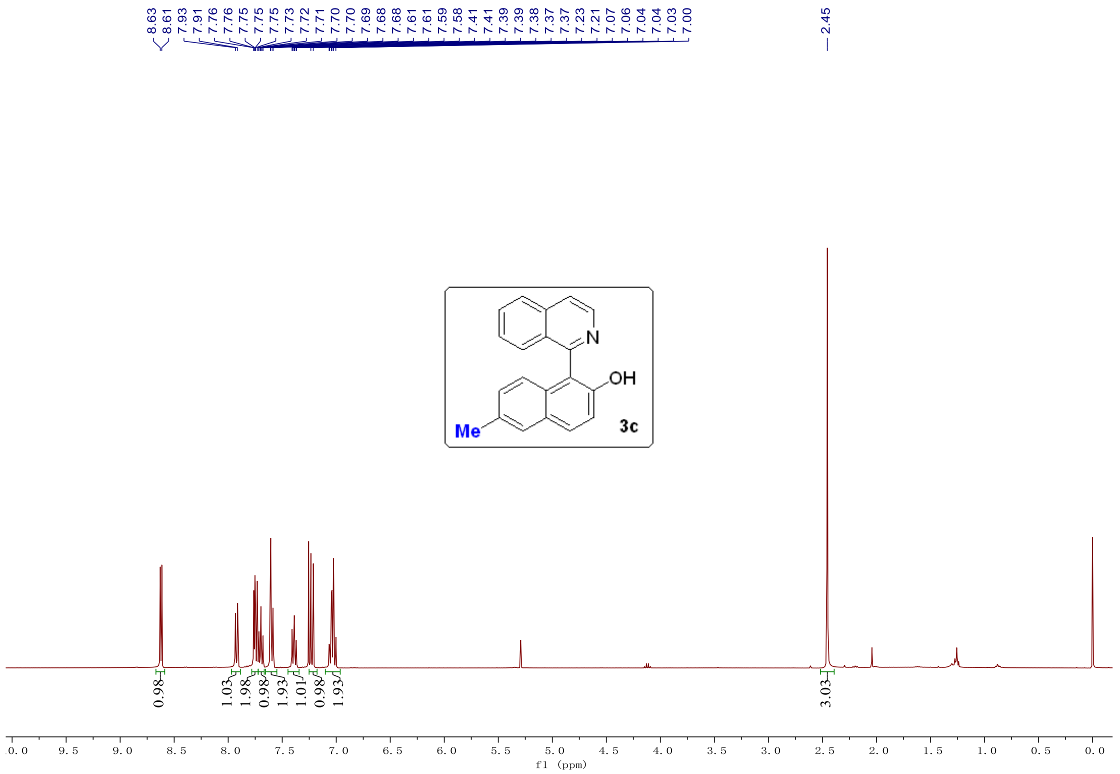

Supplementary Figure 5. ^{1}H NMR spectrum of **3c**

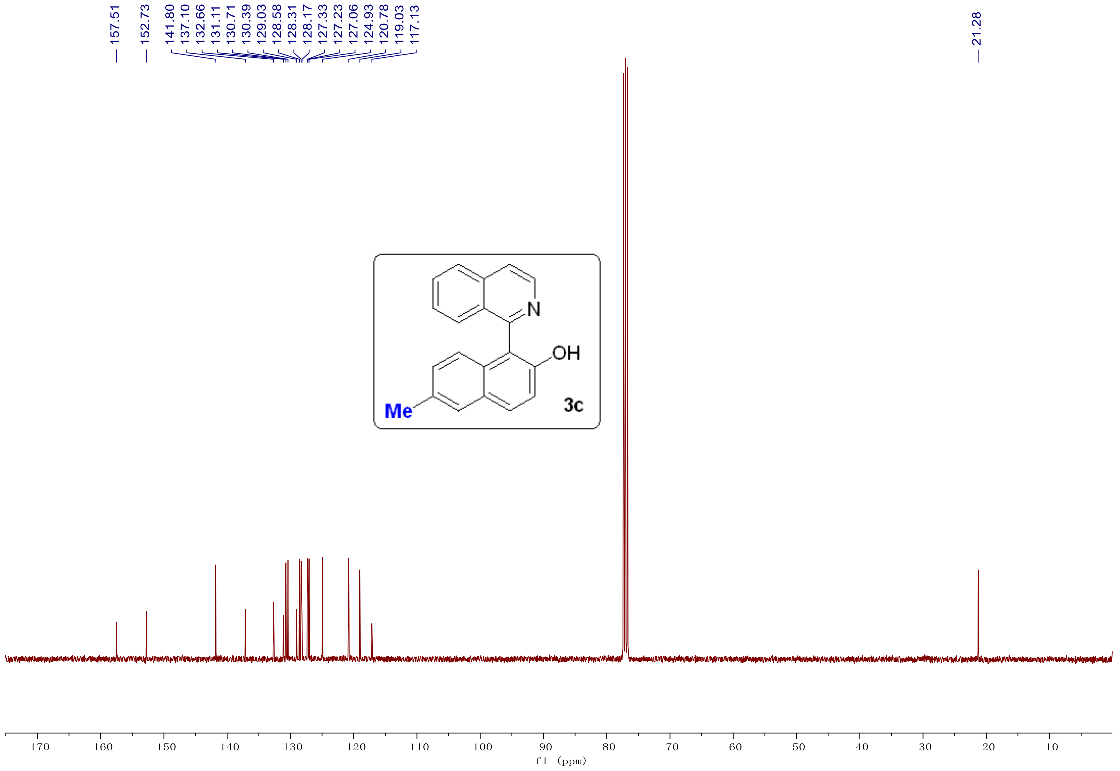

Supplementary Figure 6. ^{13}C NMR spectrum of **3c**

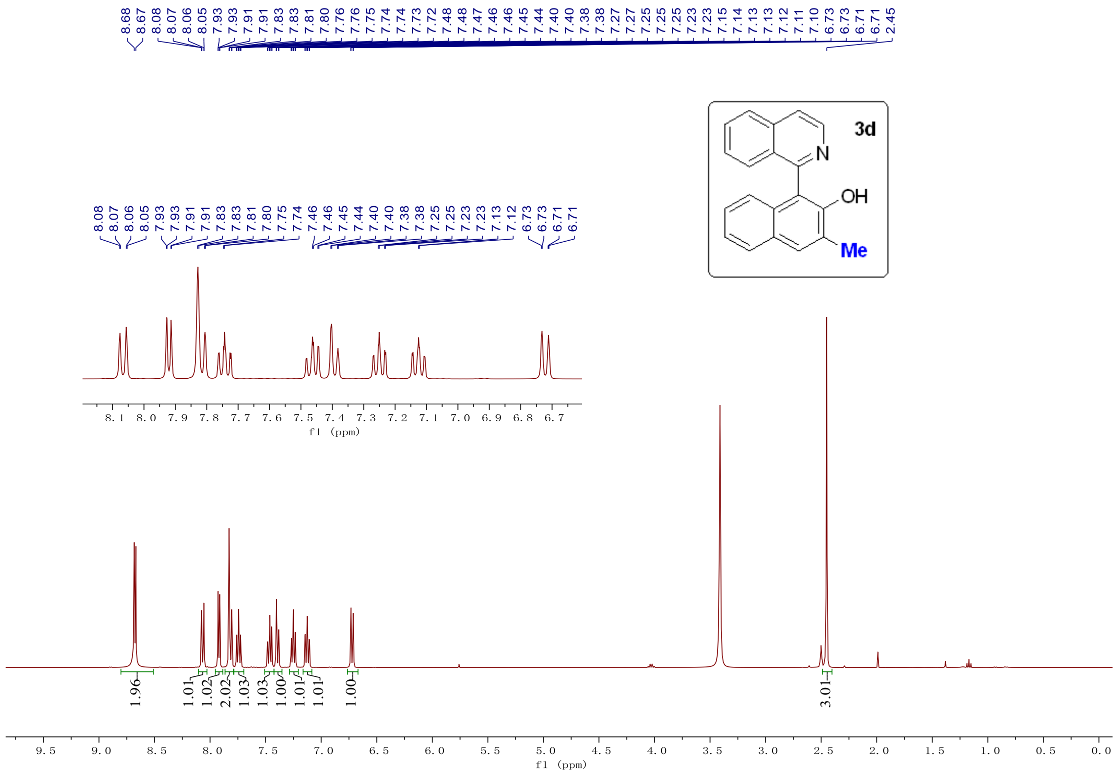

Supplementary Figure 7. ^{1}H NMR spectrum of **3d**

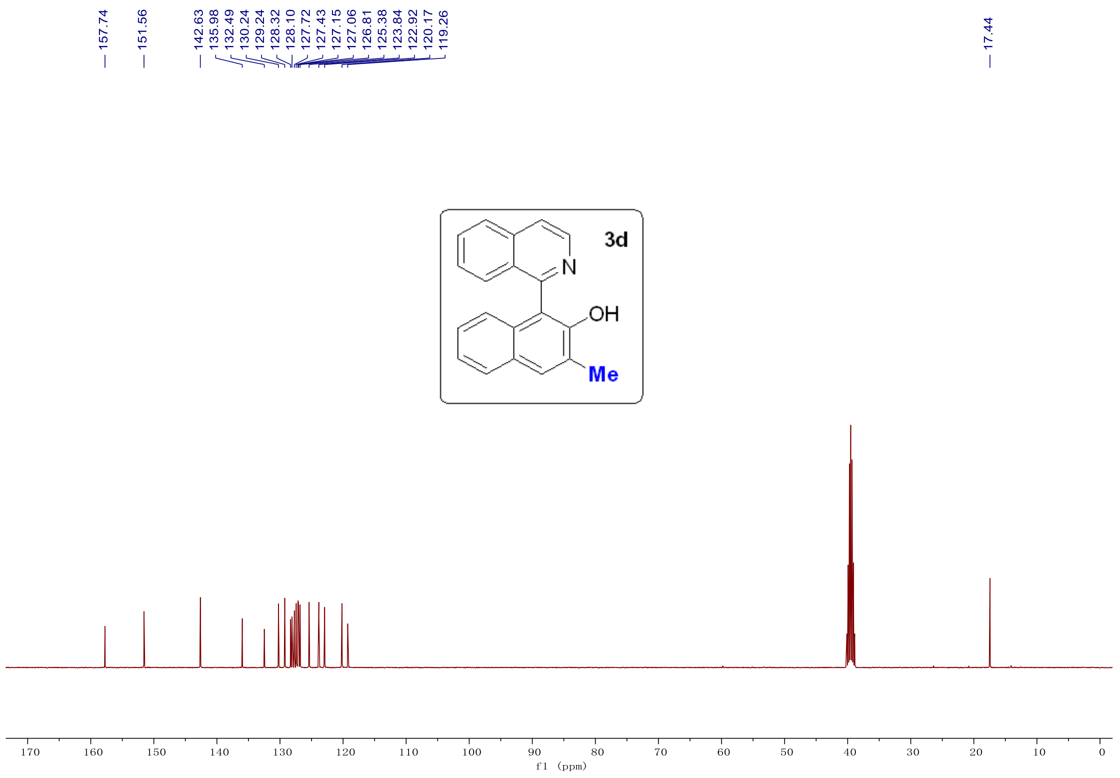

Supplementary Figure 8. ^{13}C NMR spectrum of **3d**

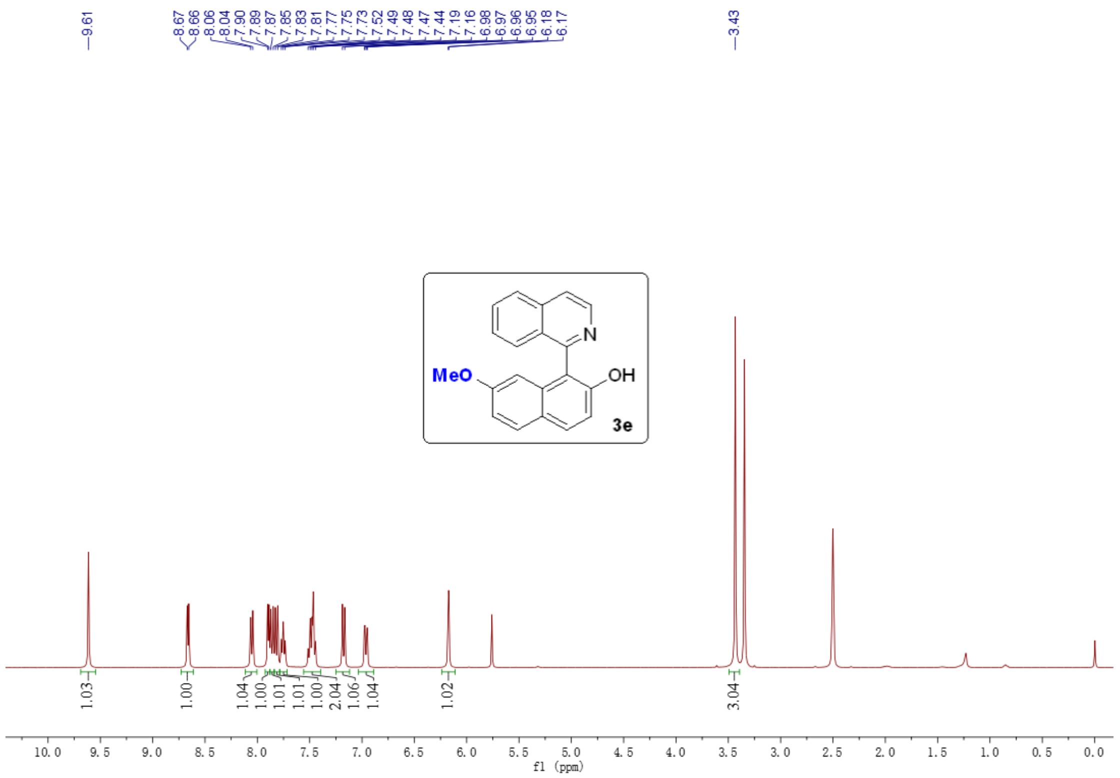

Supplementary Figure 9. ^{1}H NMR spectrum of **3e**

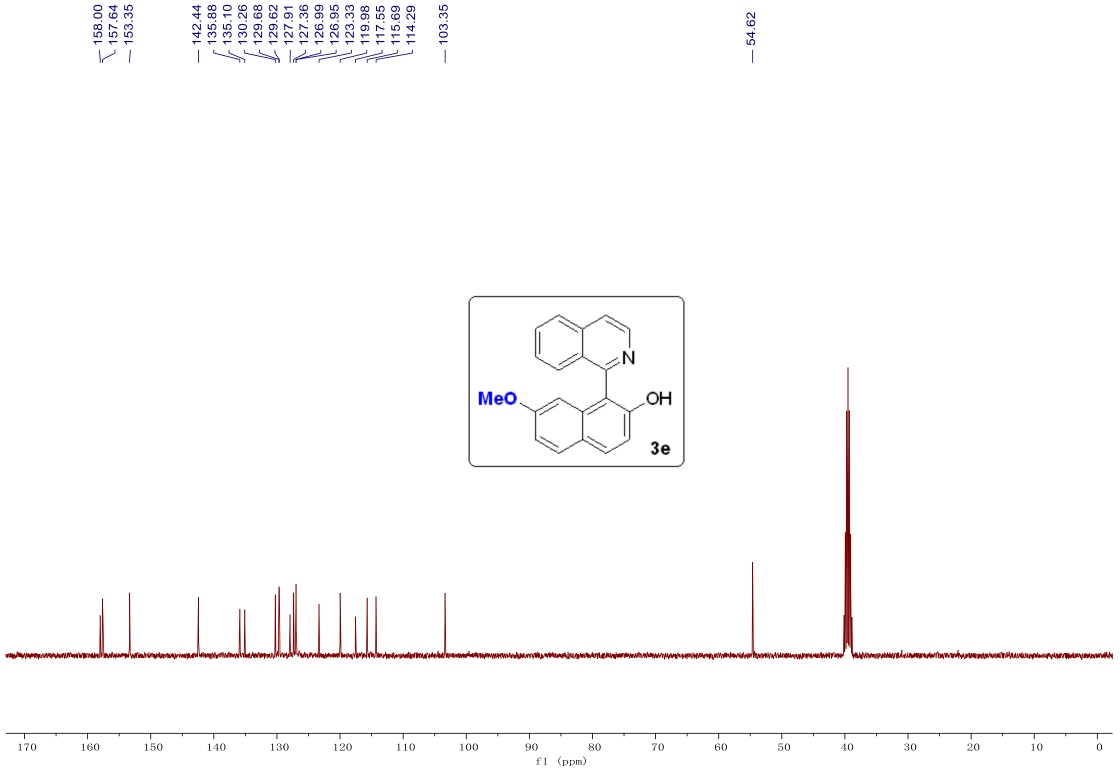

Supplementary Figure 10. ^{13}C NMR spectrum of **3e**

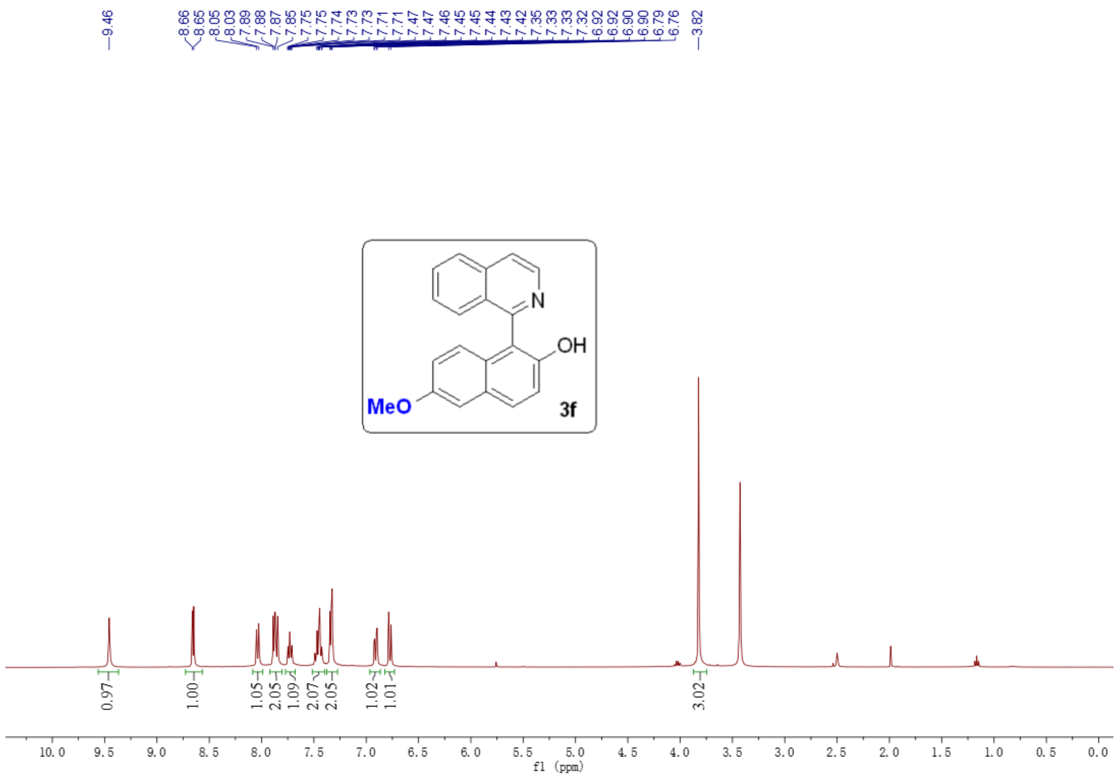

Supplementary Figure 11. ^{1}H NMR spectrum of **3f**

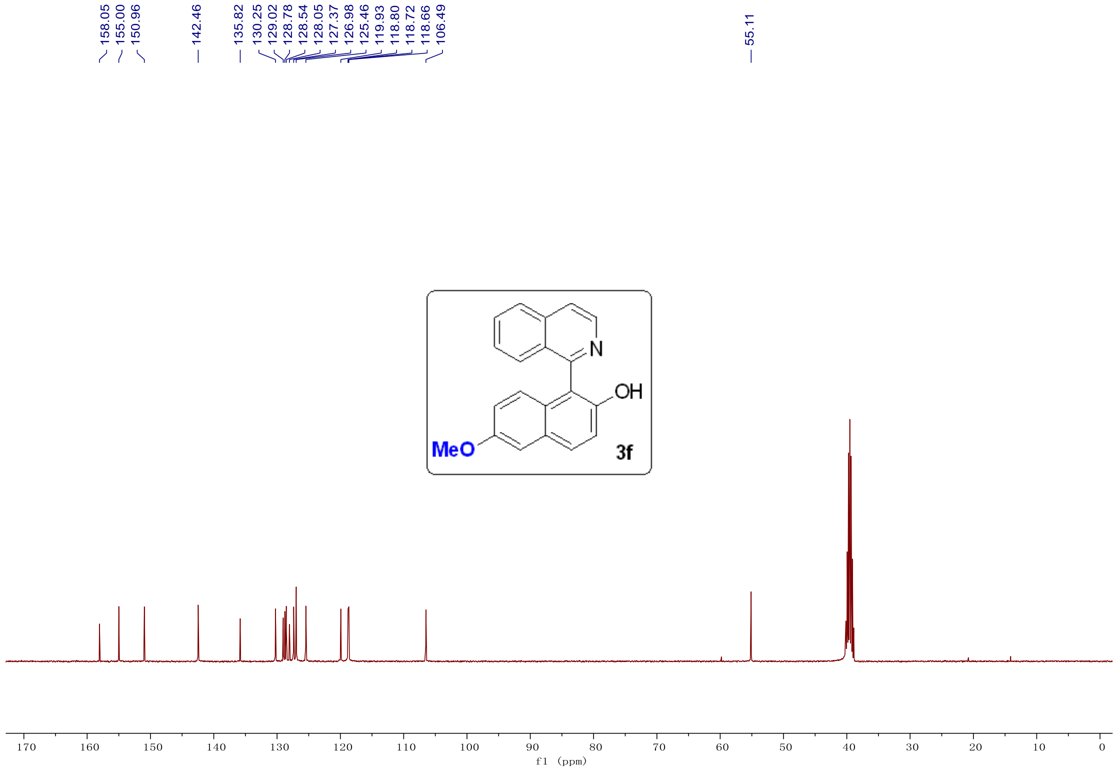

Supplementary Figure 12. ^{13}C NMR spectrum of **3f**

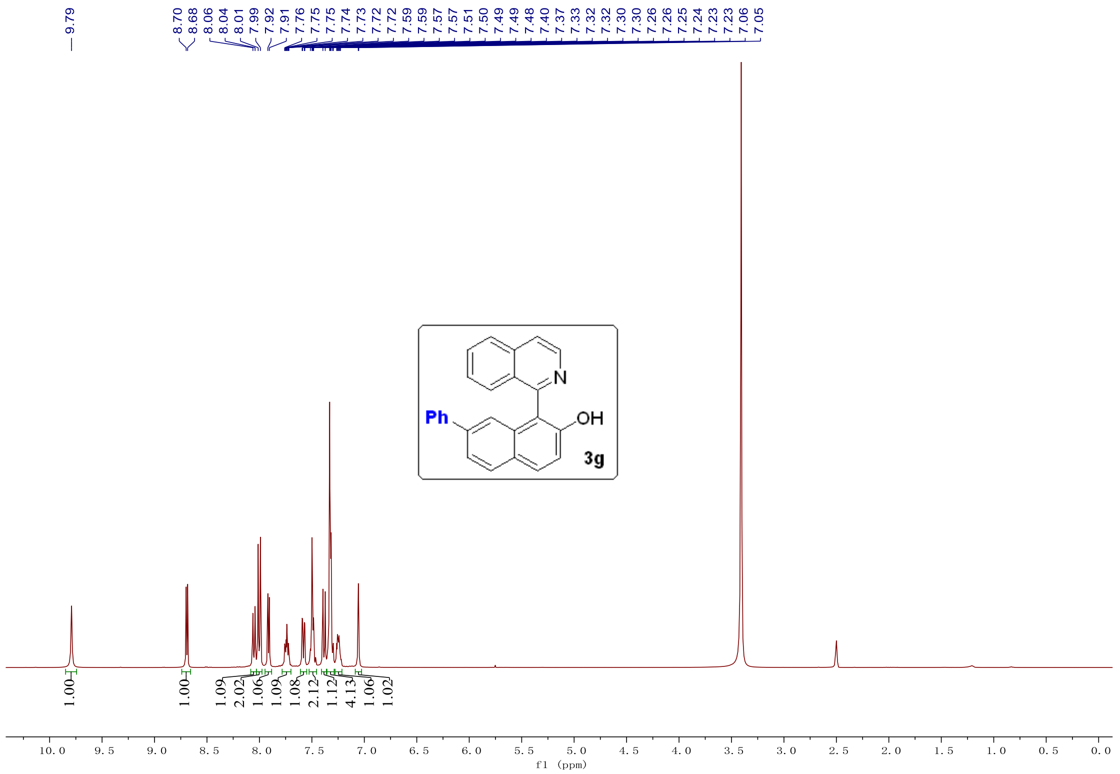

Supplementary Figure 13. ^{1}H NMR spectrum of **3g**

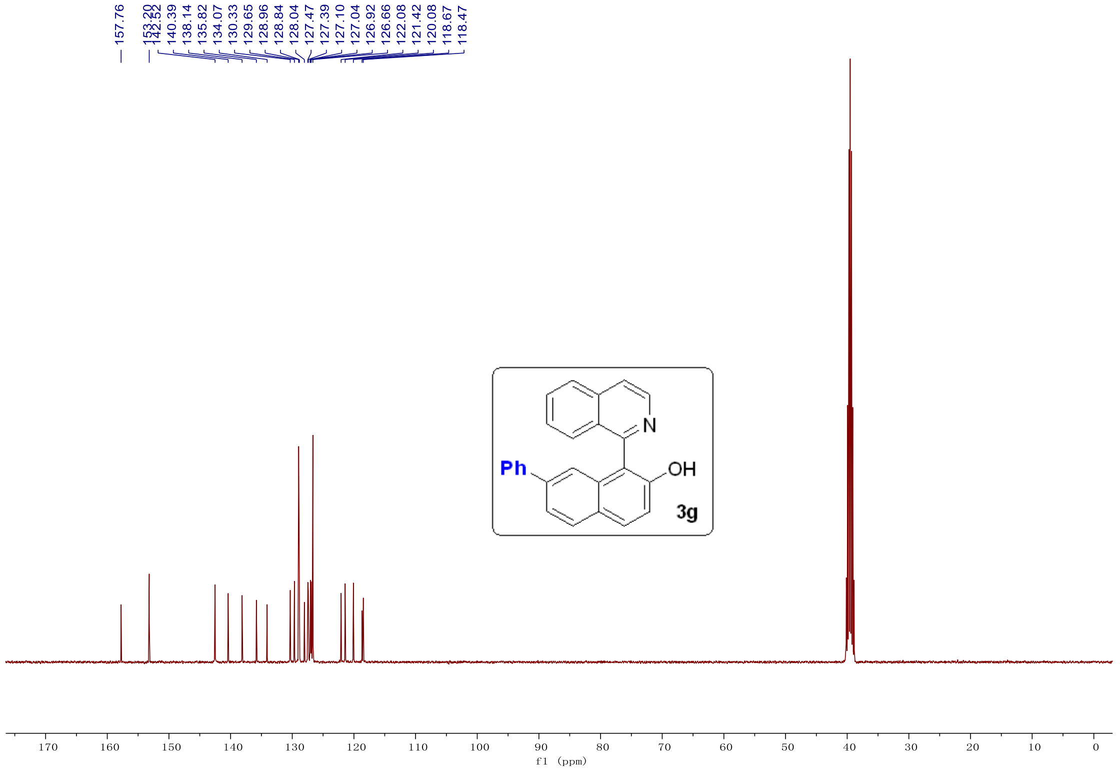

Supplementary Figure 14. ^{13}C NMR spectrum of **3g**

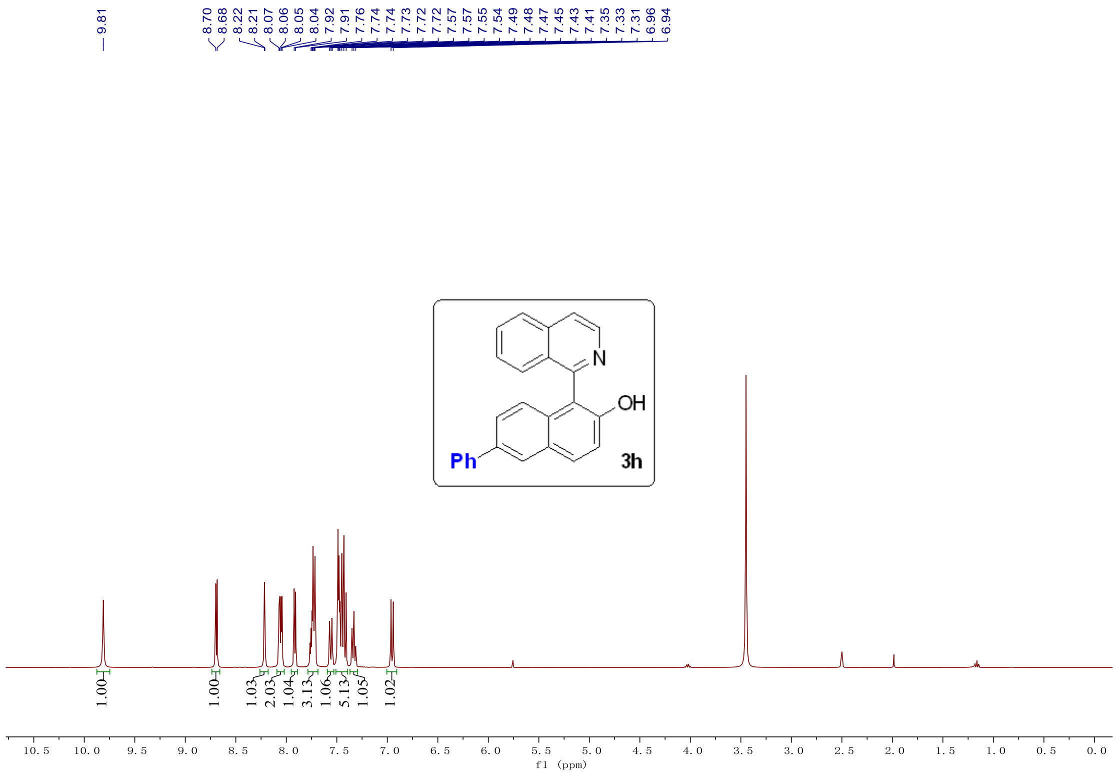

Supplementary Figure 15. ^{1}H NMR spectrum of **3h**

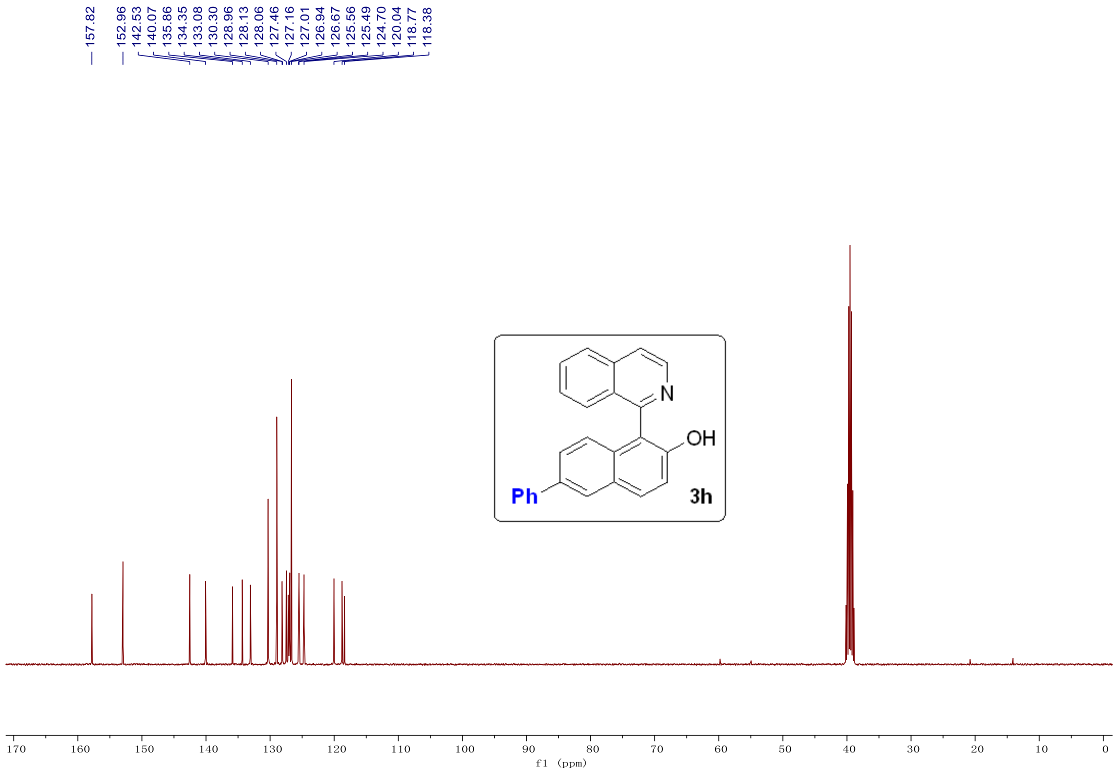

Supplementary Figure 16. ^{13}C NMR spectrum of **3h**

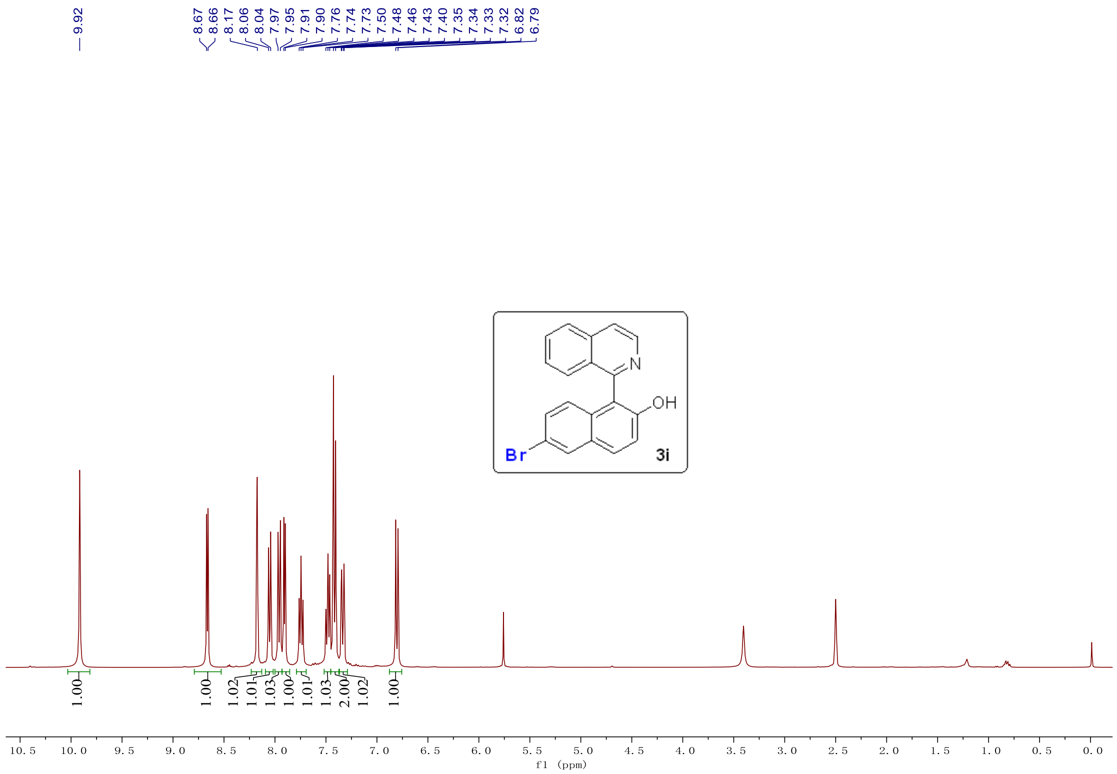

Supplementary Figure 17. ^{1}H NMR spectrum of **3i**

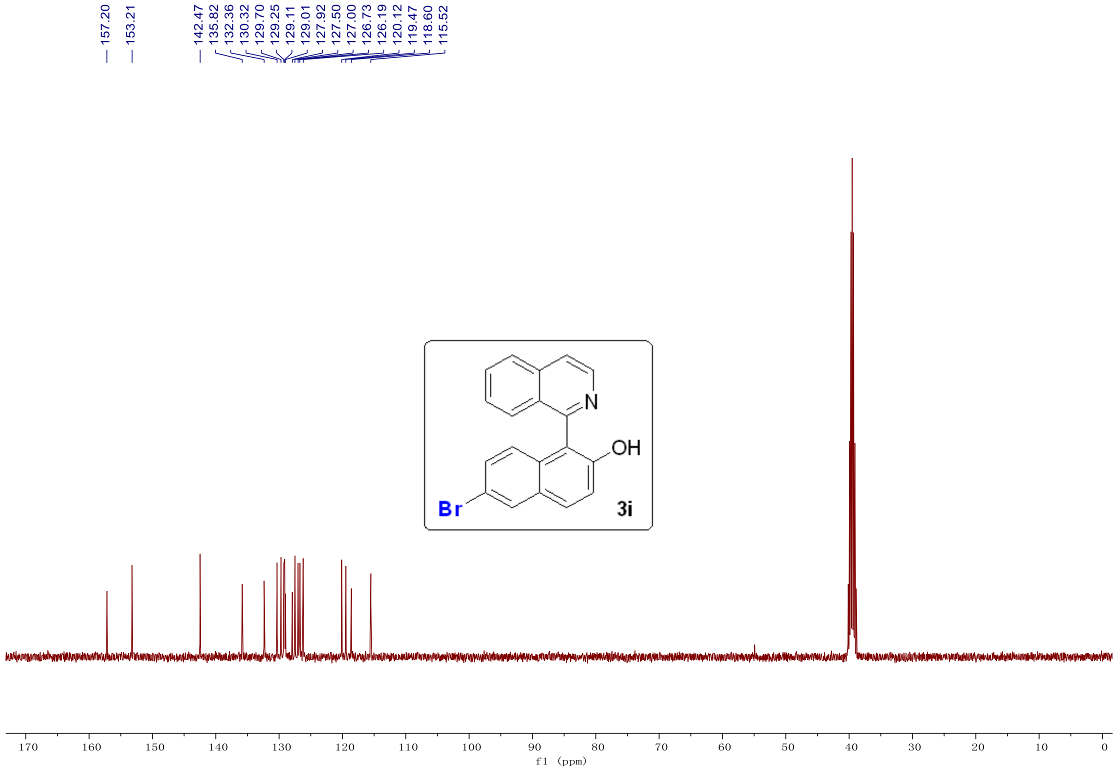

Supplementary Figure 18. ^{13}C NMR spectrum of **3i**

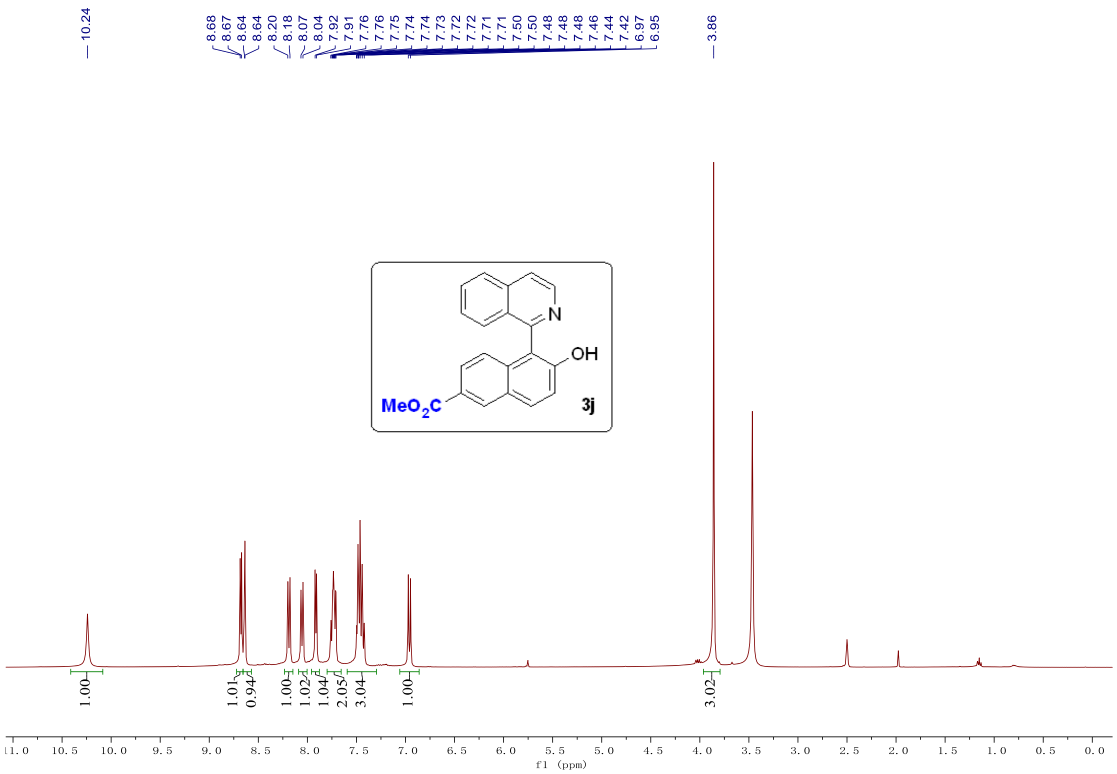

Supplementary Figure 19. ^{1}H NMR spectrum of **3j**

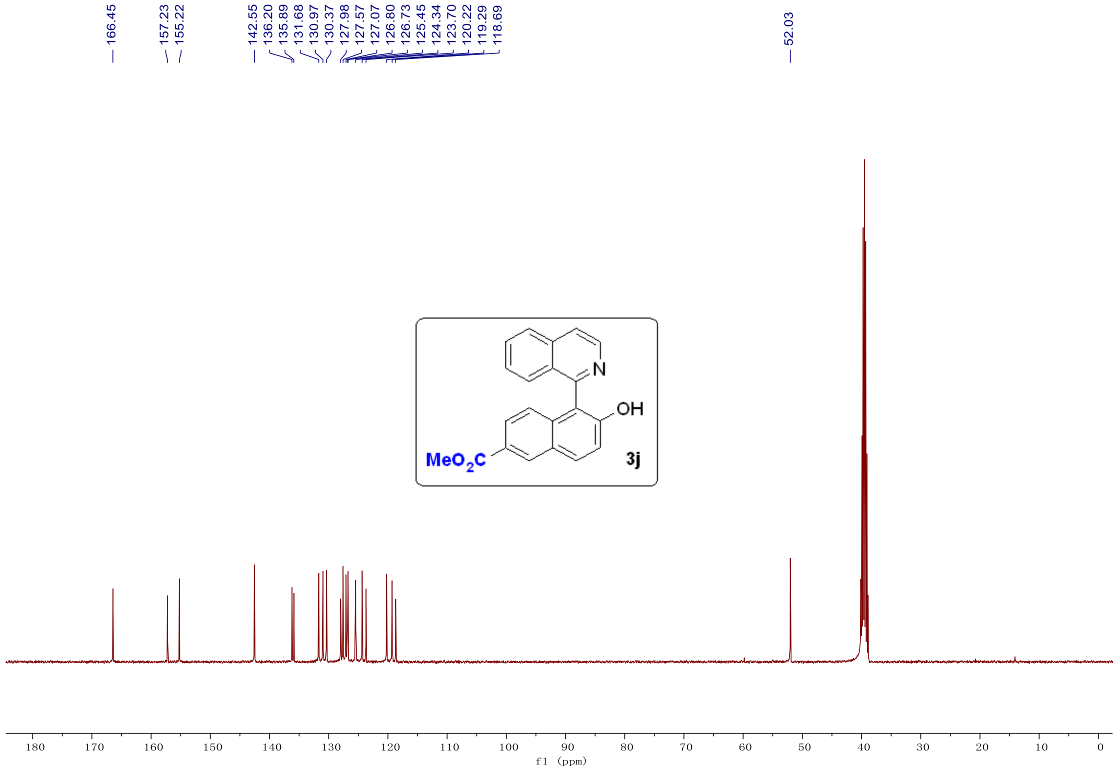

Supplementary Figure 20. ^{13}C NMR spectrum of **3j**

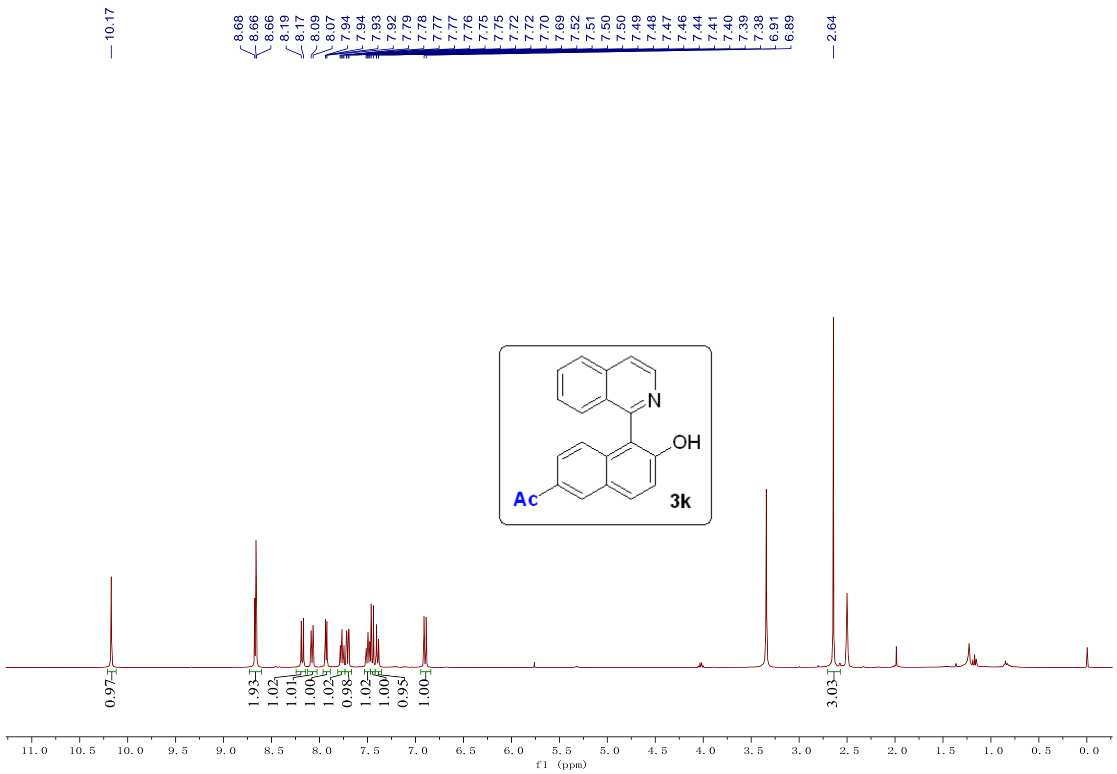

Supplementary Figure 21. ^{1}H NMR spectrum of **3k**

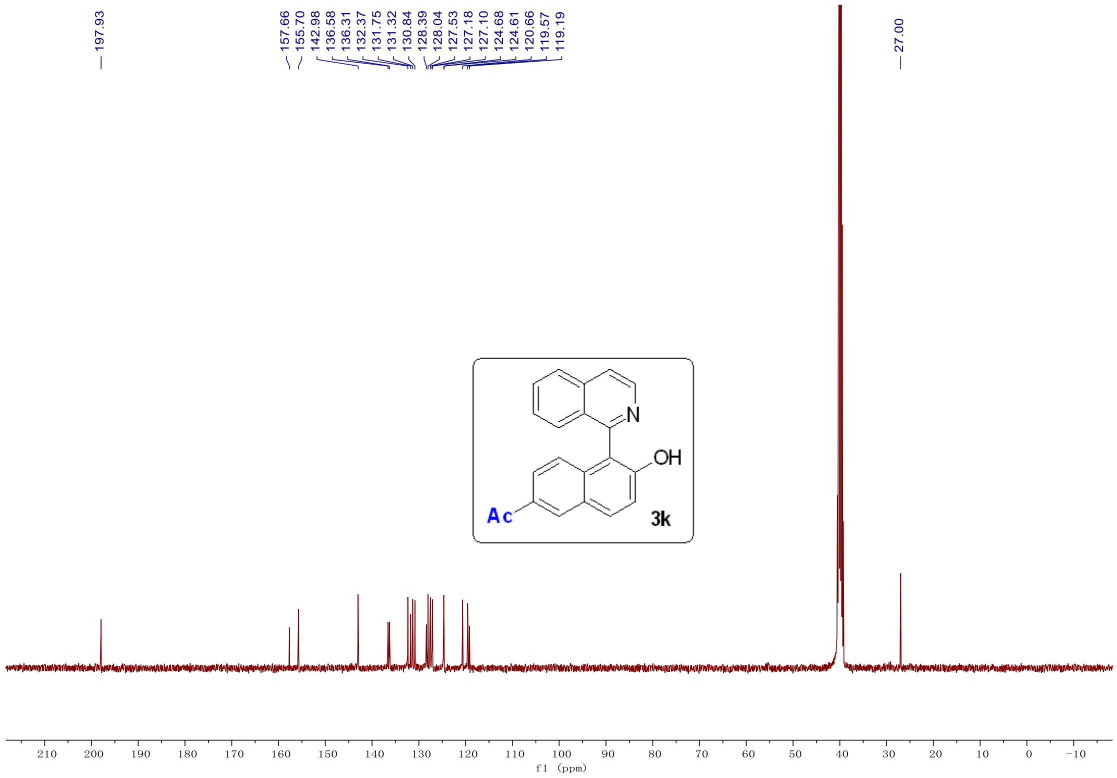

Supplementary Figure 22. ^{13}C NMR spectrum of **3k**

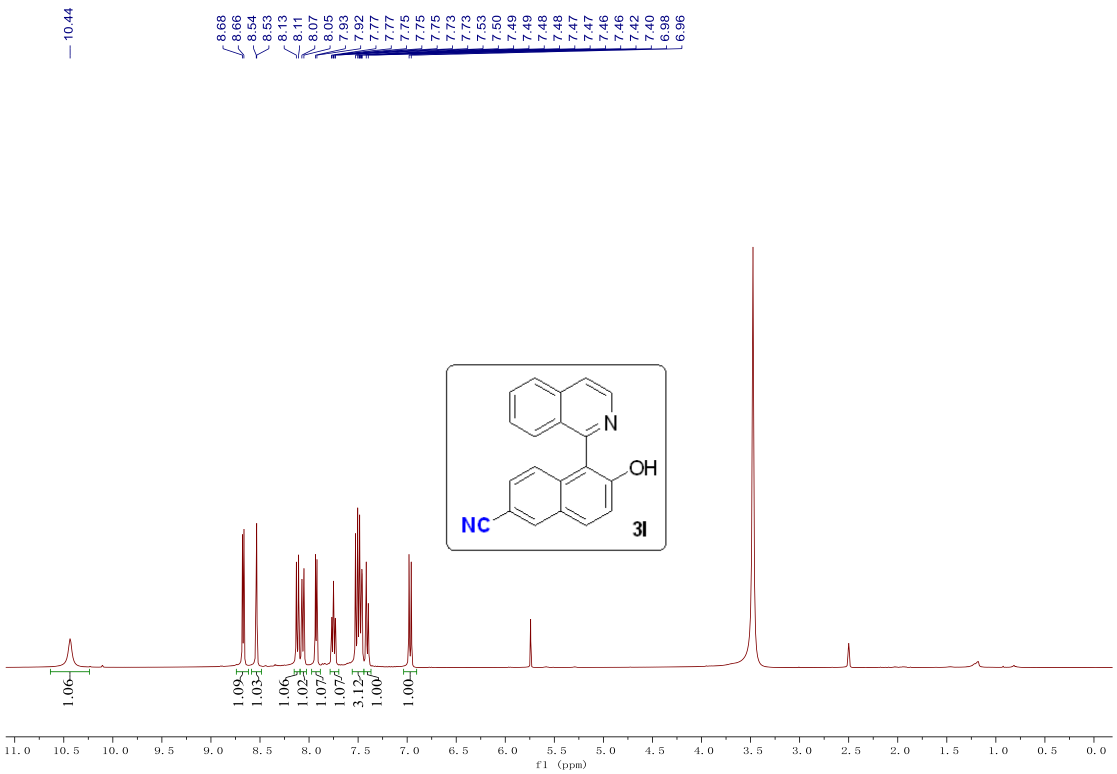

Supplementary Figure 23. ^{1}H NMR spectrum of **3l**

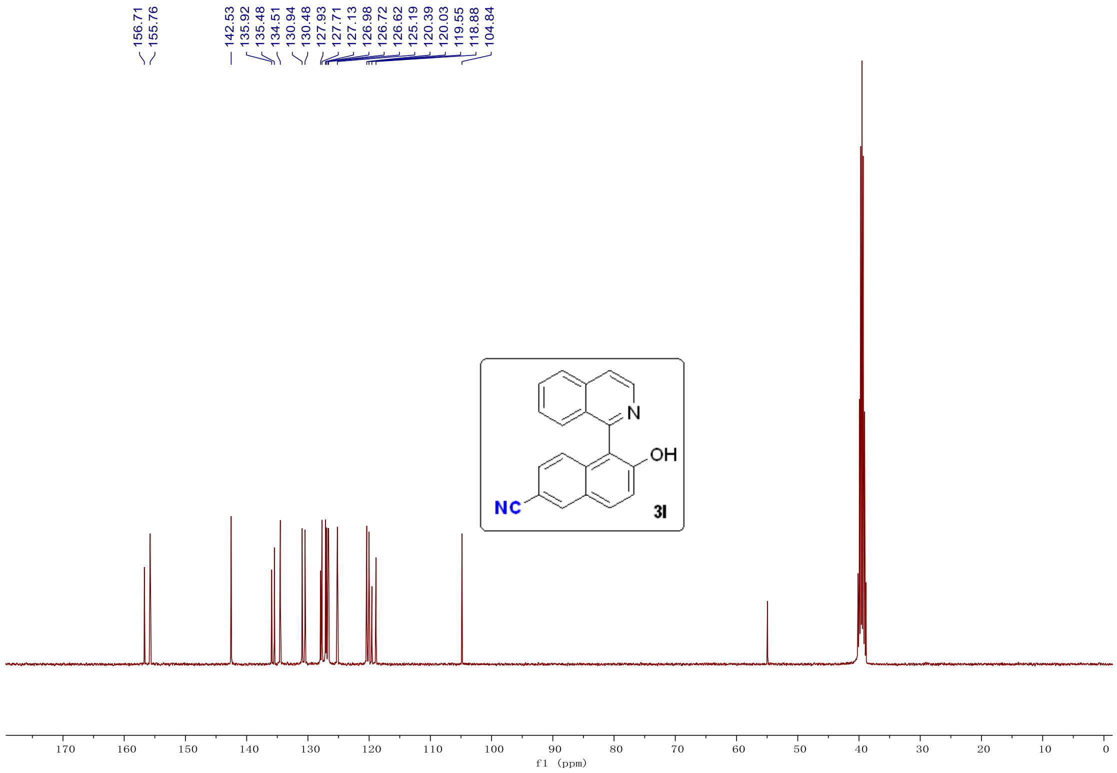

Supplementary Figure 24. ^{13}C NMR spectrum of **3l**

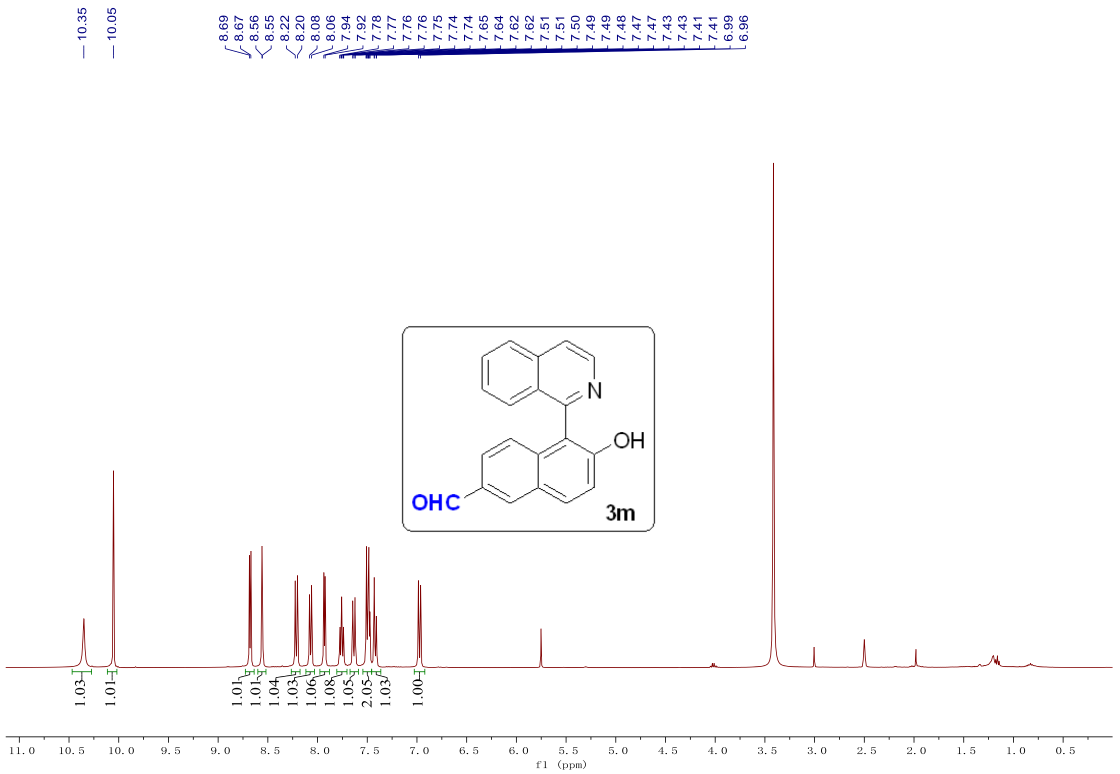

Supplementary Figure 25. ^{1}H NMR spectrum of **3m**

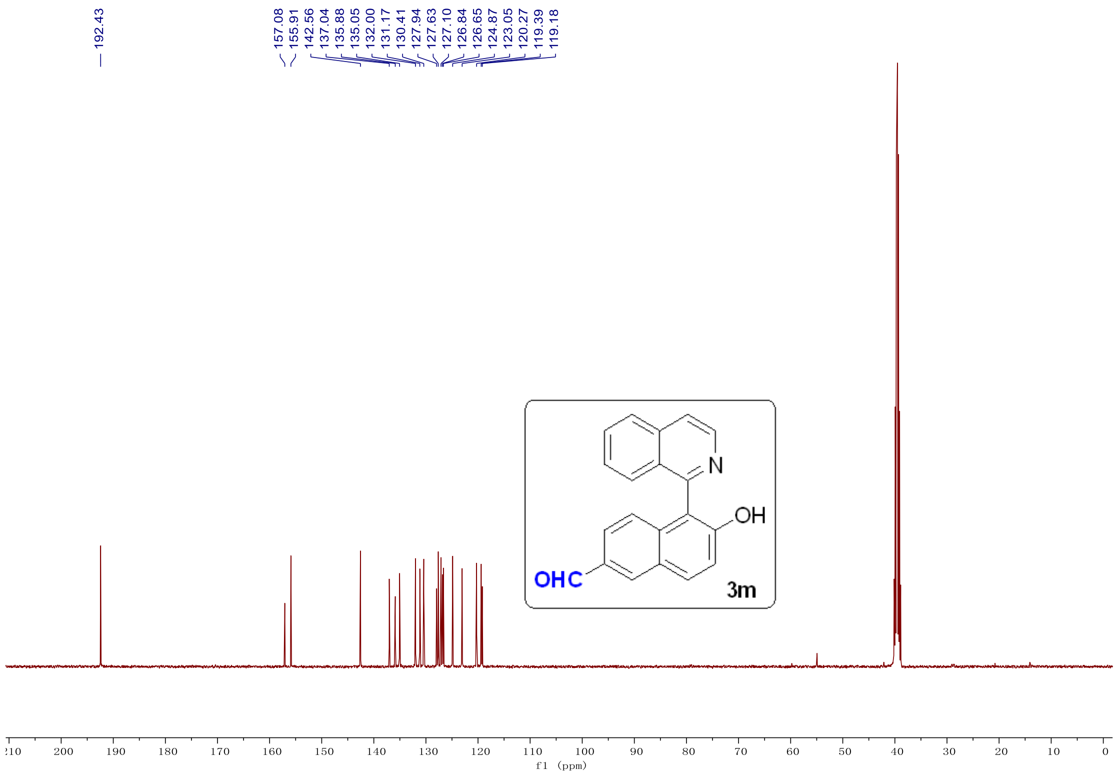

Supplementary Figure 26. ^{13}C NMR spectrum of **3m**

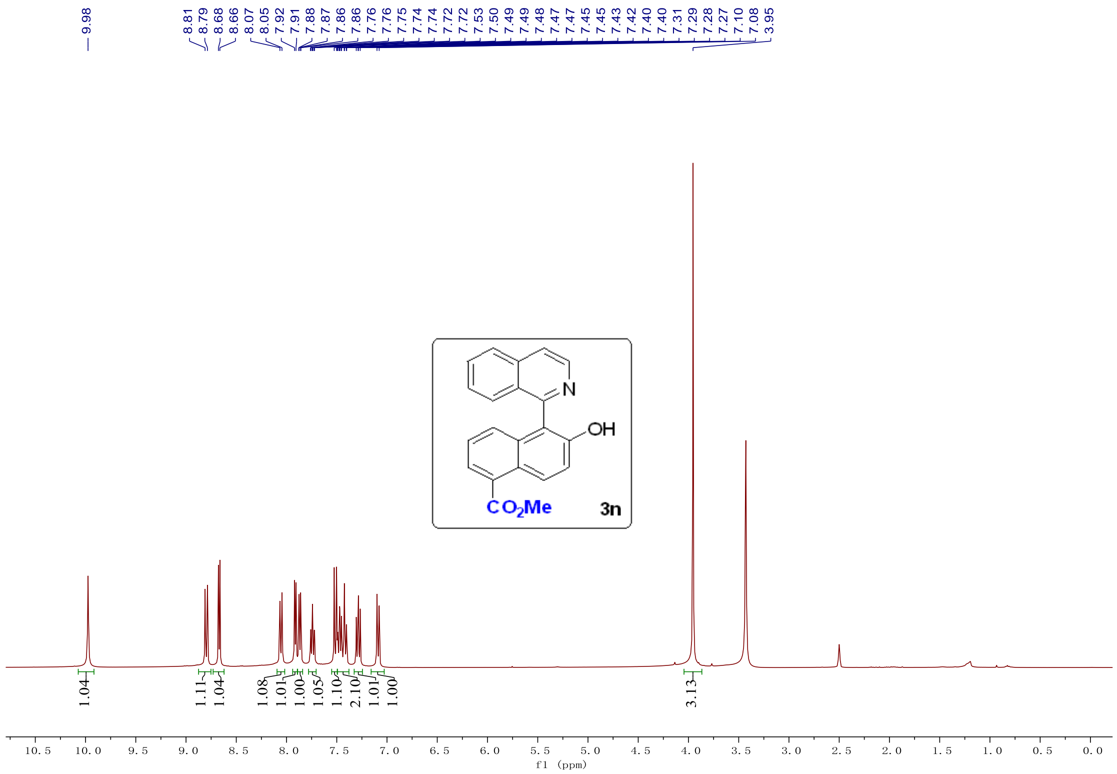

Supplementary Figure 27. ^{1}H NMR spectrum of **3n**

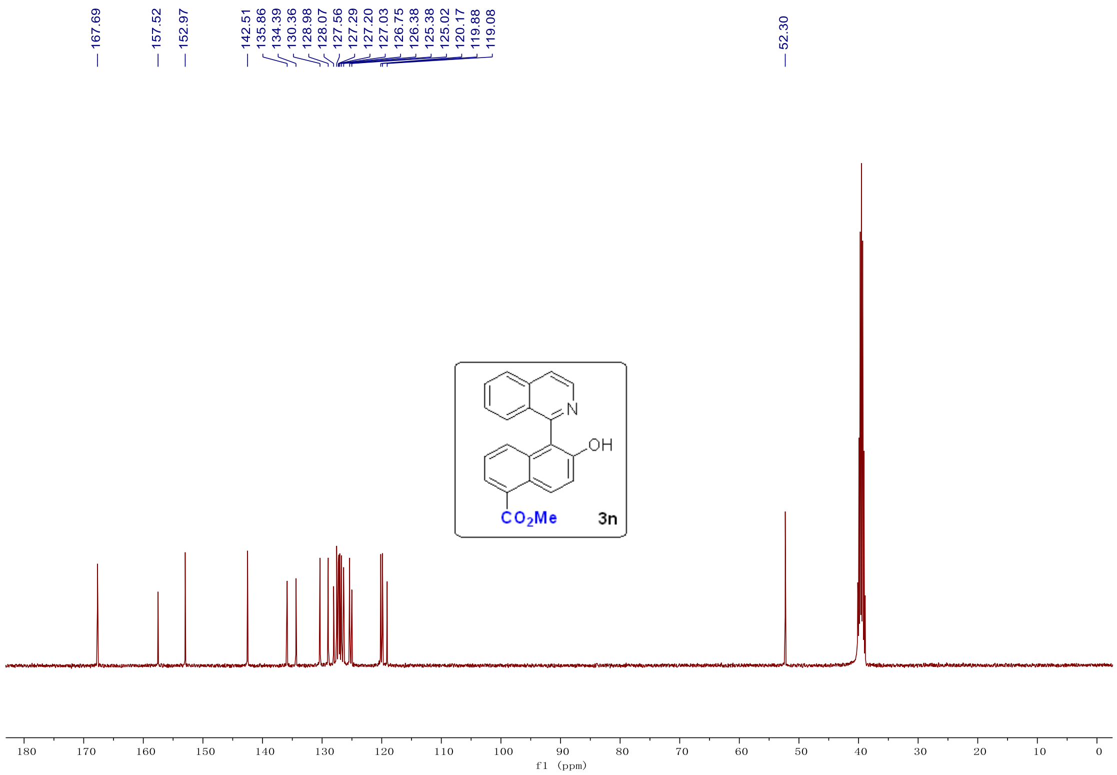

Supplementary Figure 28. ^{13}C NMR spectrum of **3n**

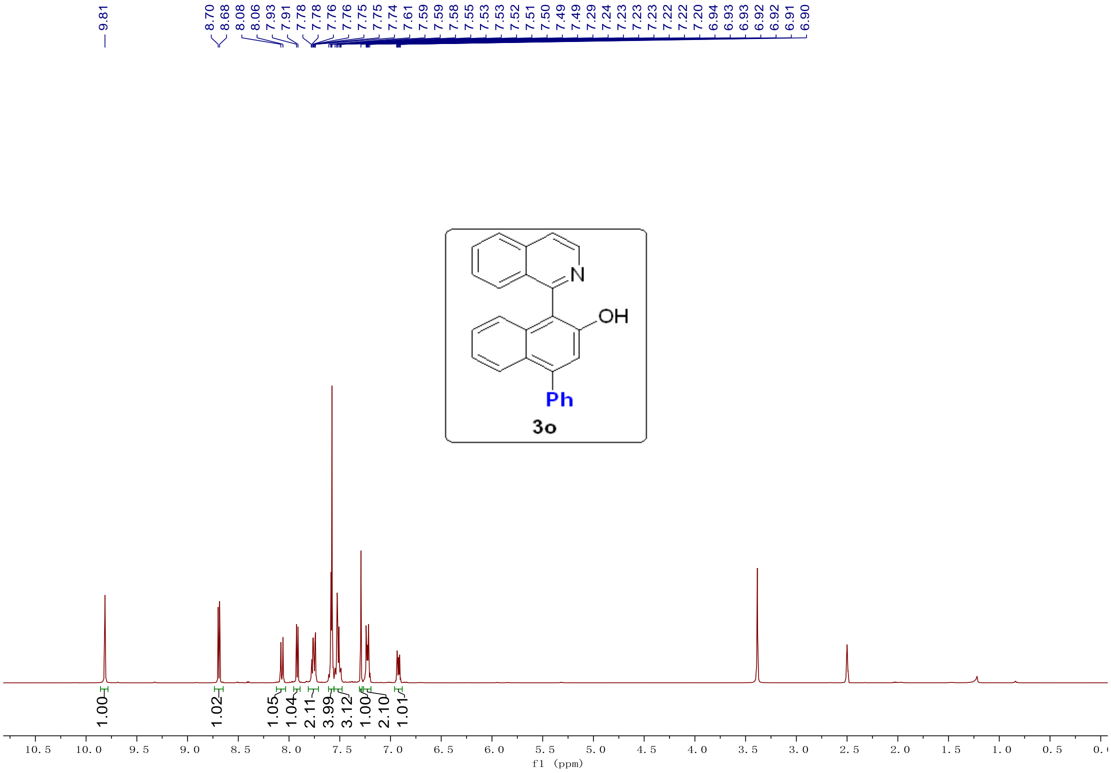

Supplementary Figure 29. ^{1}H NMR spectrum of **3o**

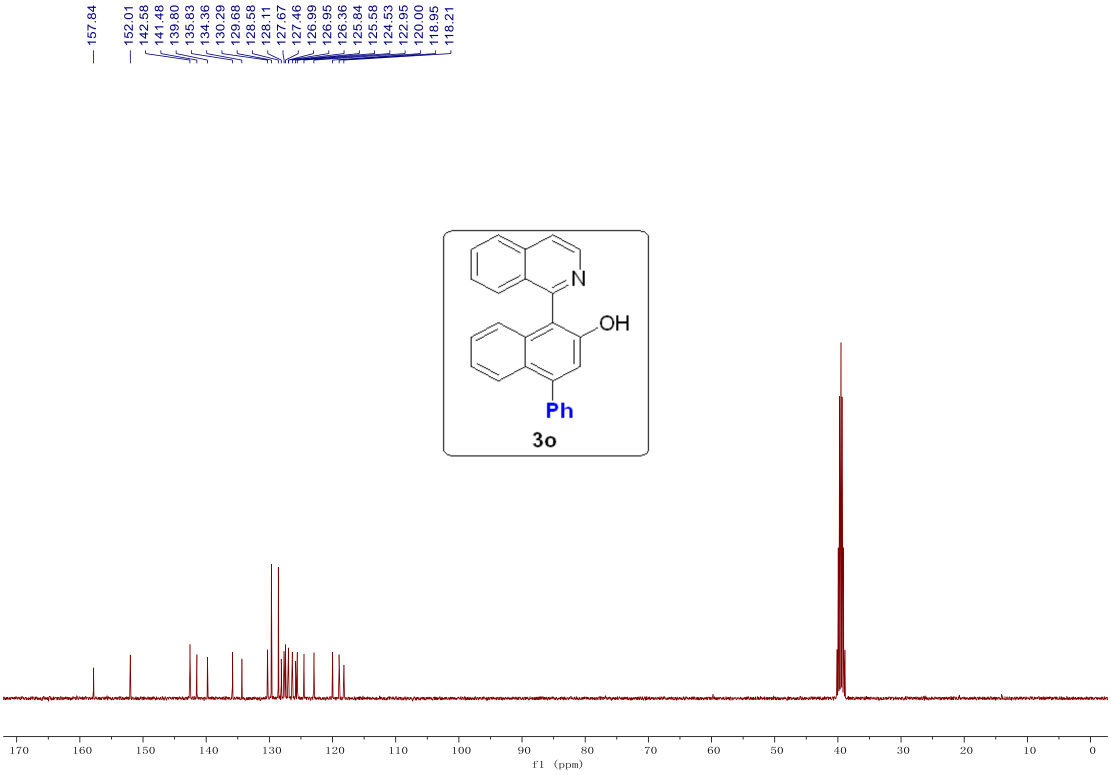

Supplementary Figure 30. ^{13}C NMR spectrum of **3o**

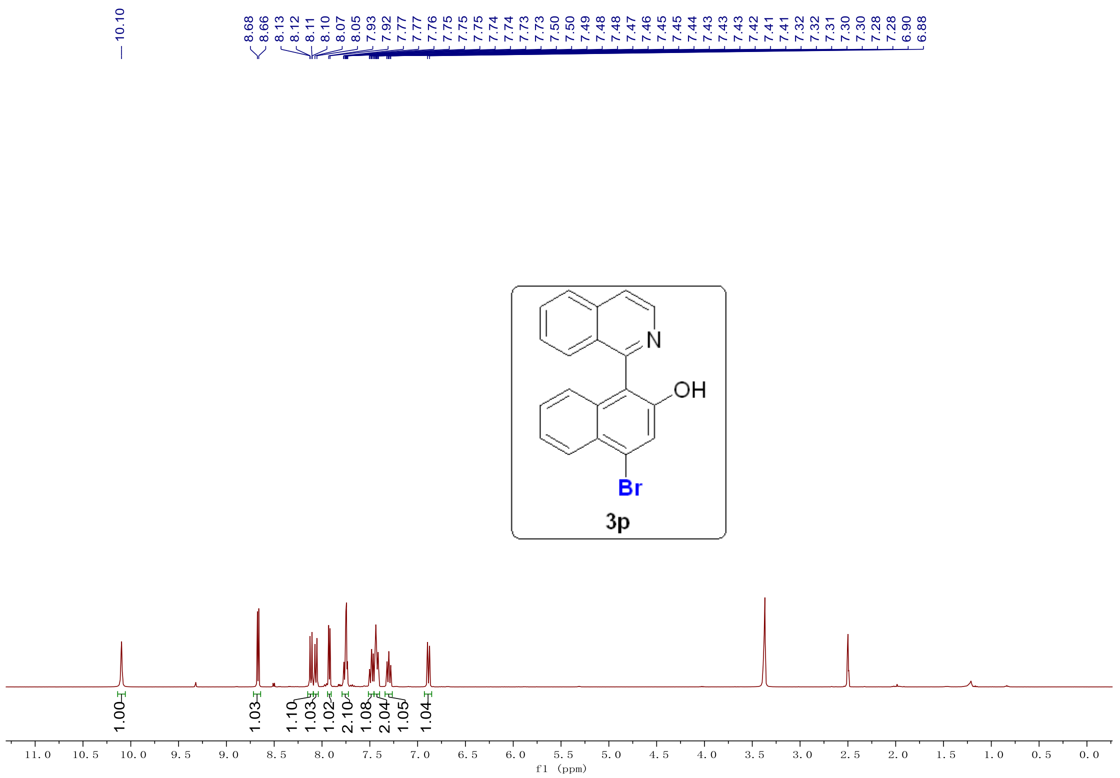

Supplementary Figure 31. ^{1}H NMR spectrum of **3p**

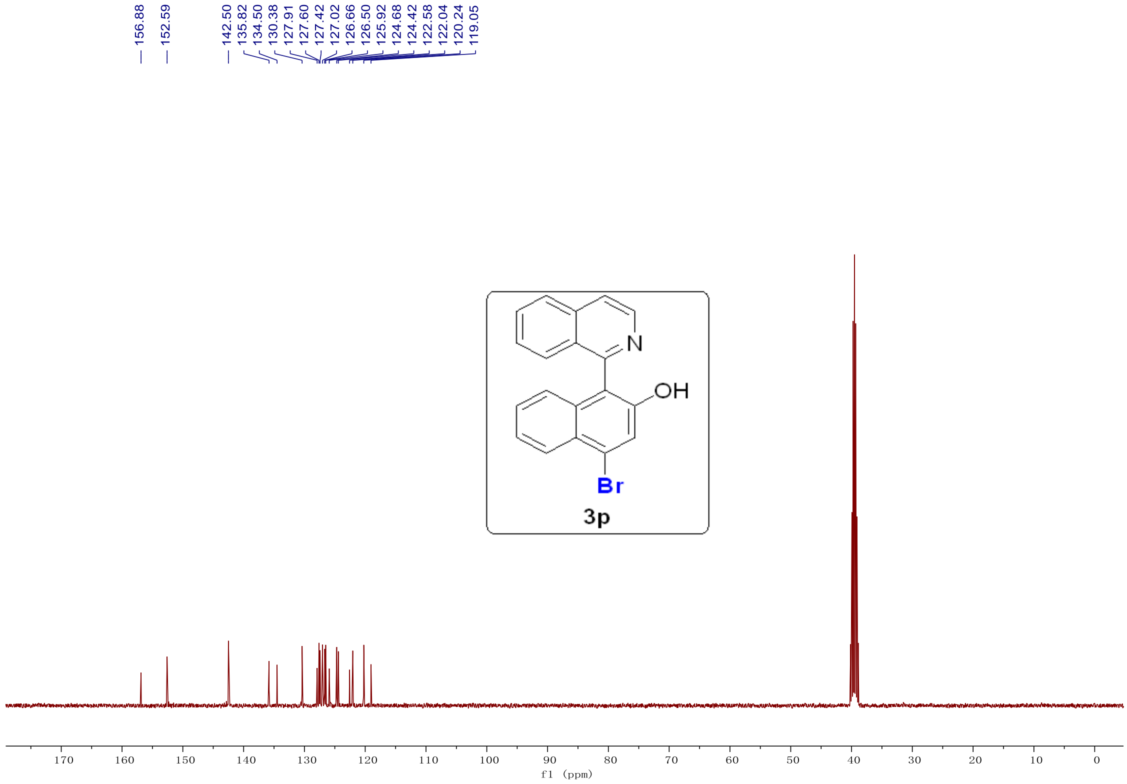

Supplementary Figure 32. ^{13}C NMR spectrum of **3p**

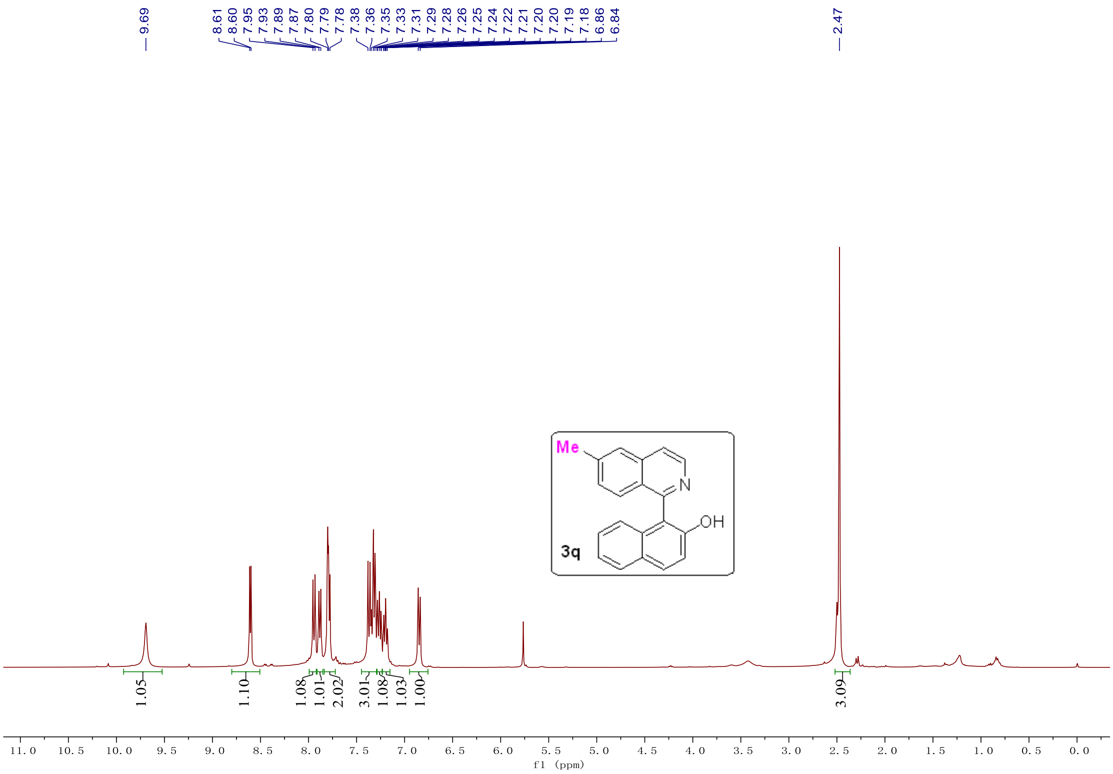

Supplementary Figure 33. ^{1}H NMR spectrum of **3q**

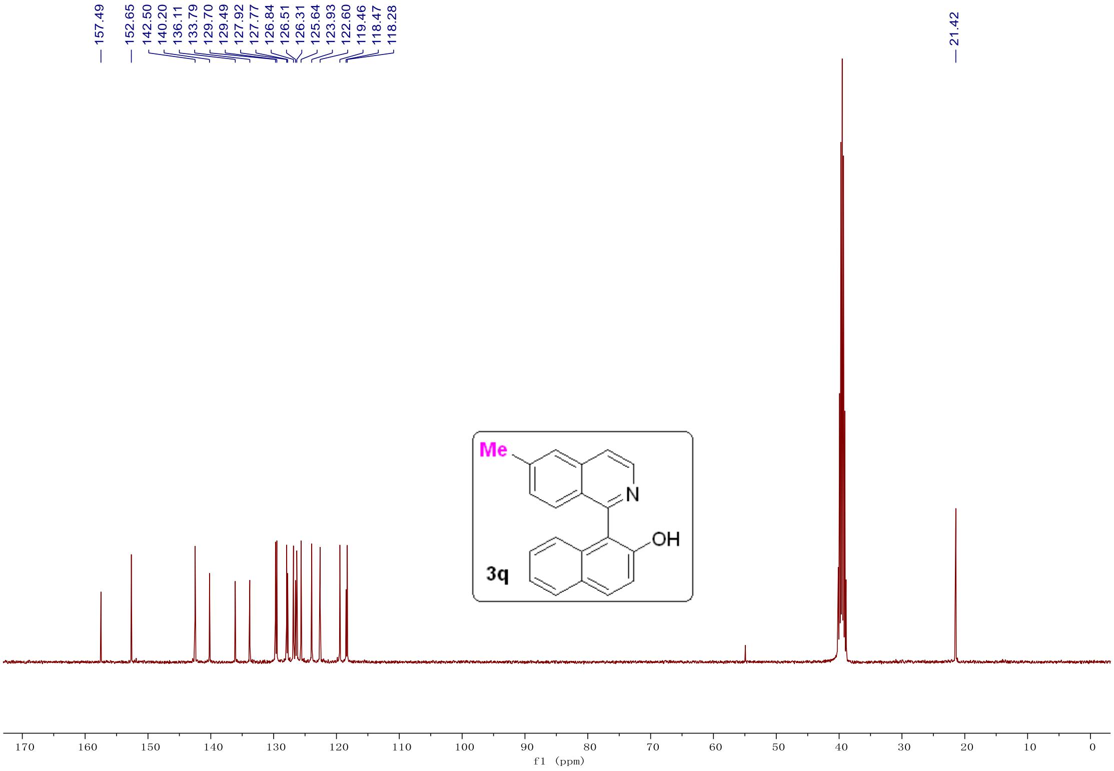

Supplementary Figure 34. ^{13}C NMR spectrum of **3q**

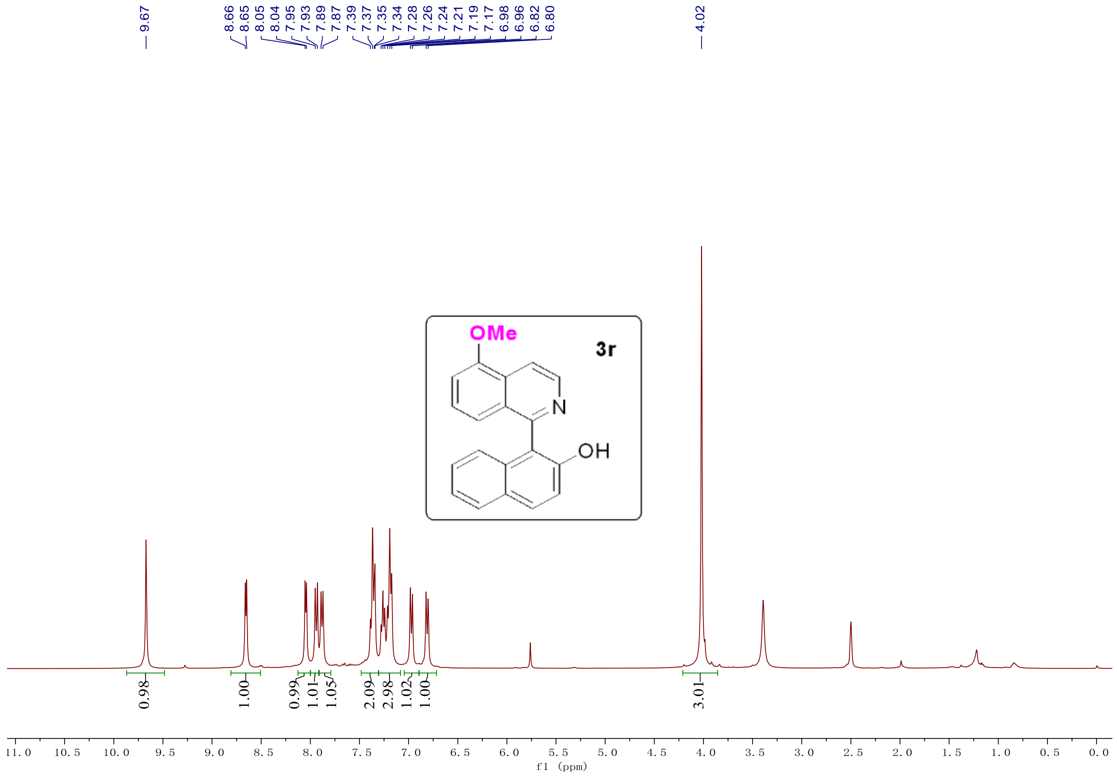

Supplementary Figure 35. ^{1}H NMR spectrum of **3r**

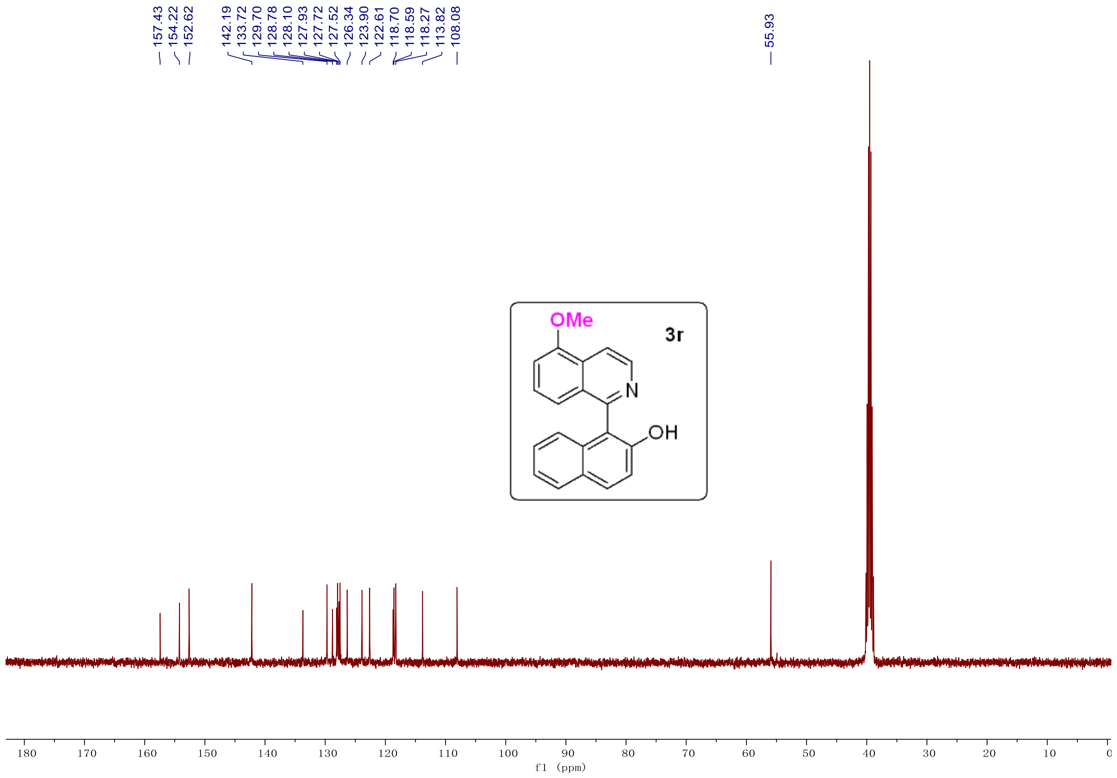

Supplementary Figure 36. ^{13}C NMR spectrum of **3r**

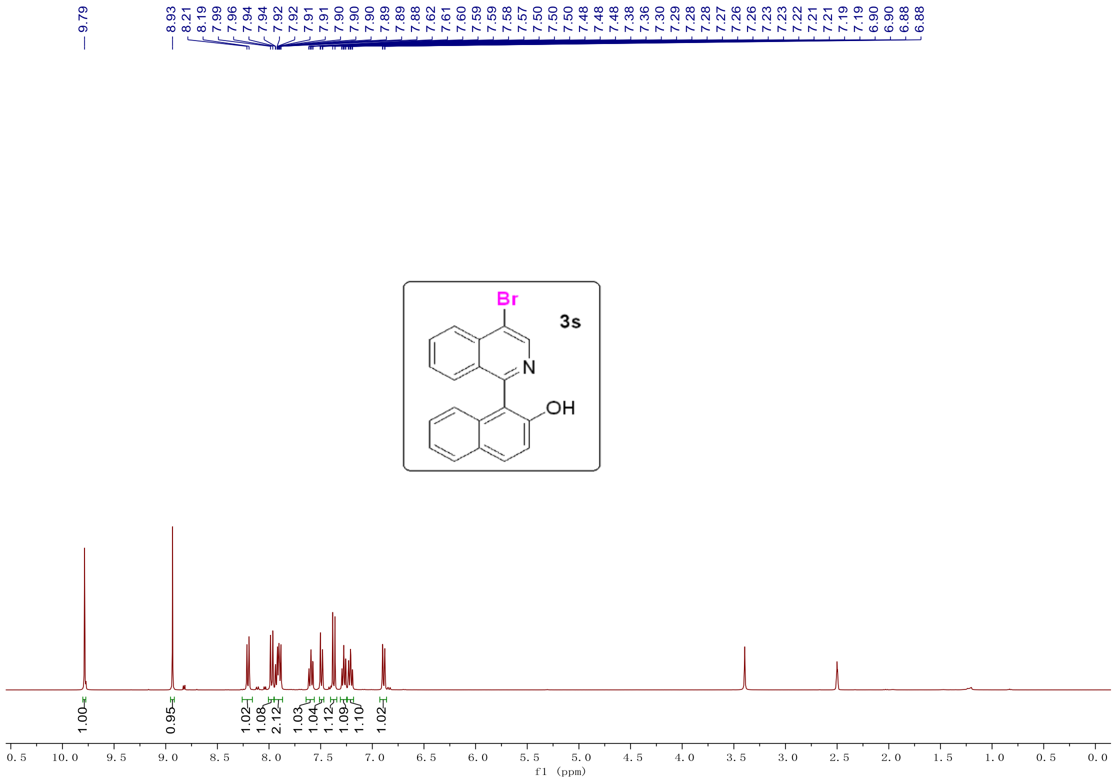

Supplementary Figure 37. ^{1}H NMR spectrum of **3s**

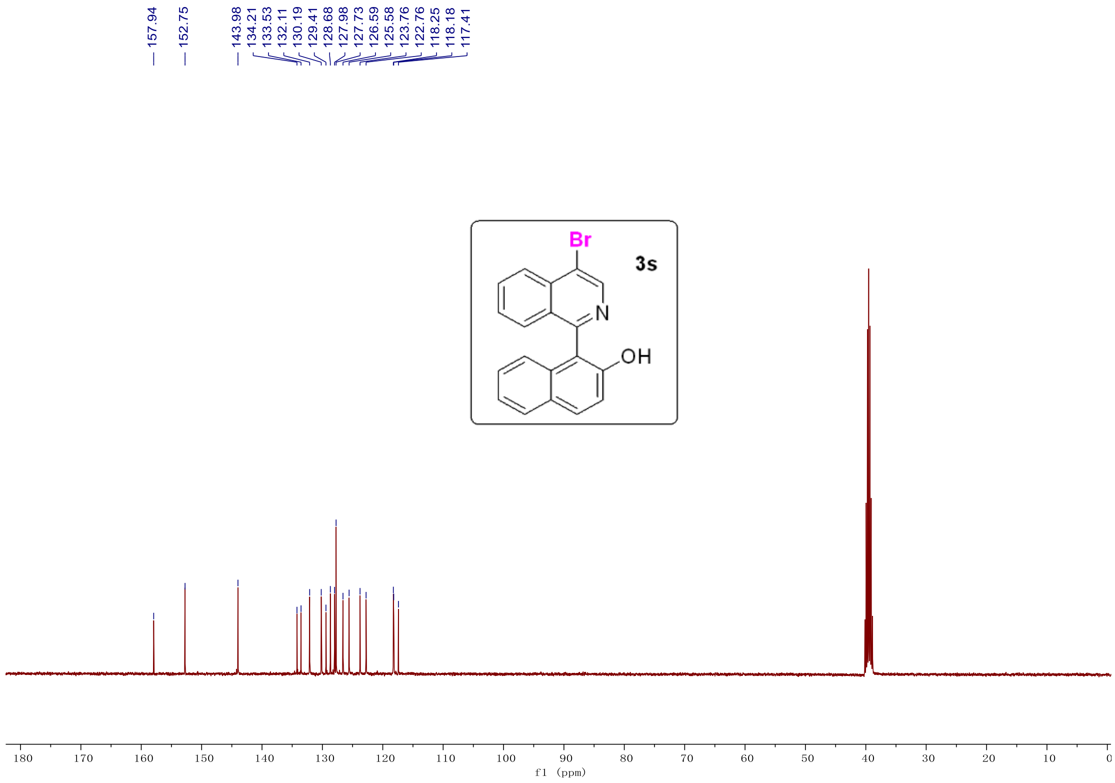

Supplementary Figure 38. ^{13}C NMR spectrum of **3s**

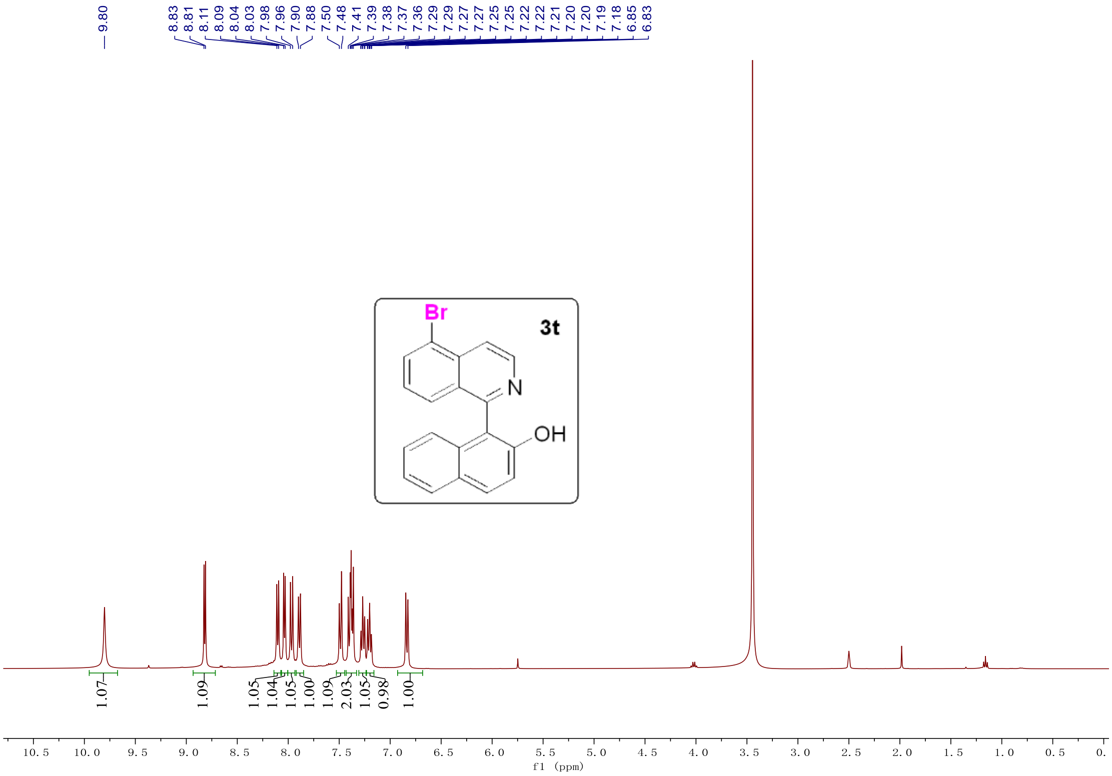

Supplementary Figure 39. ^{1}H NMR spectrum of **3t**

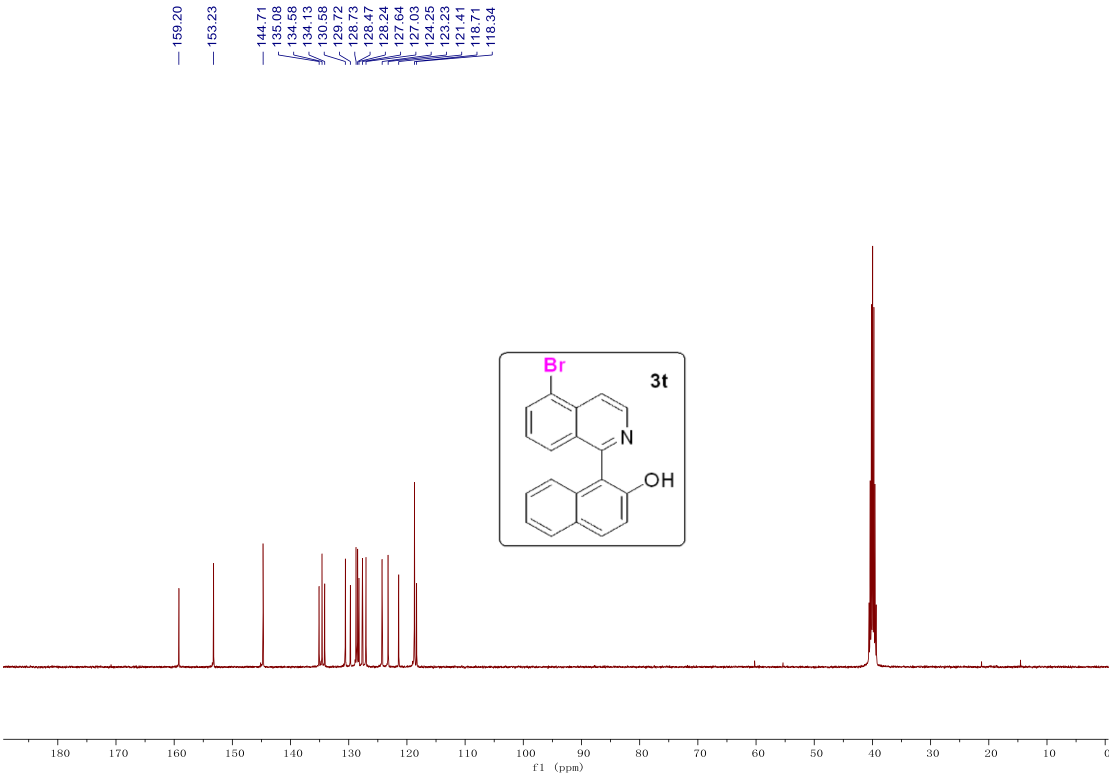

Supplementary Figure 40. ^{13}C NMR spectrum of **3t**

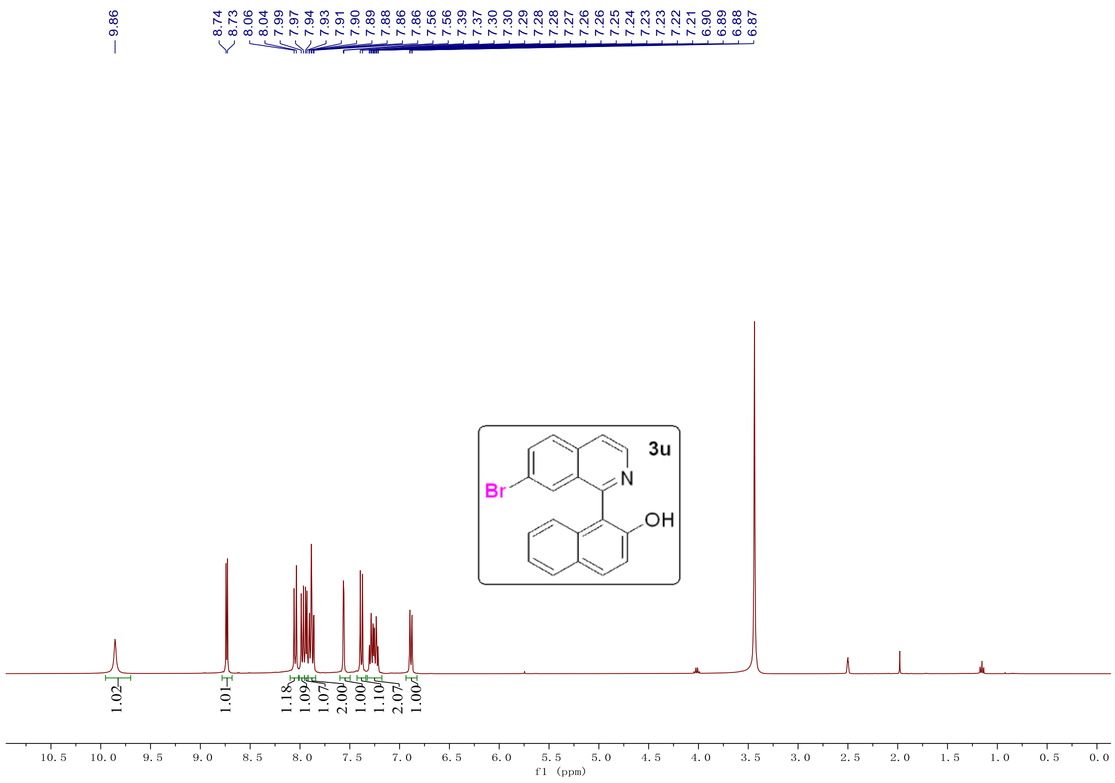

Supplementary Figure 41. ^{1}H NMR spectrum of **3u**

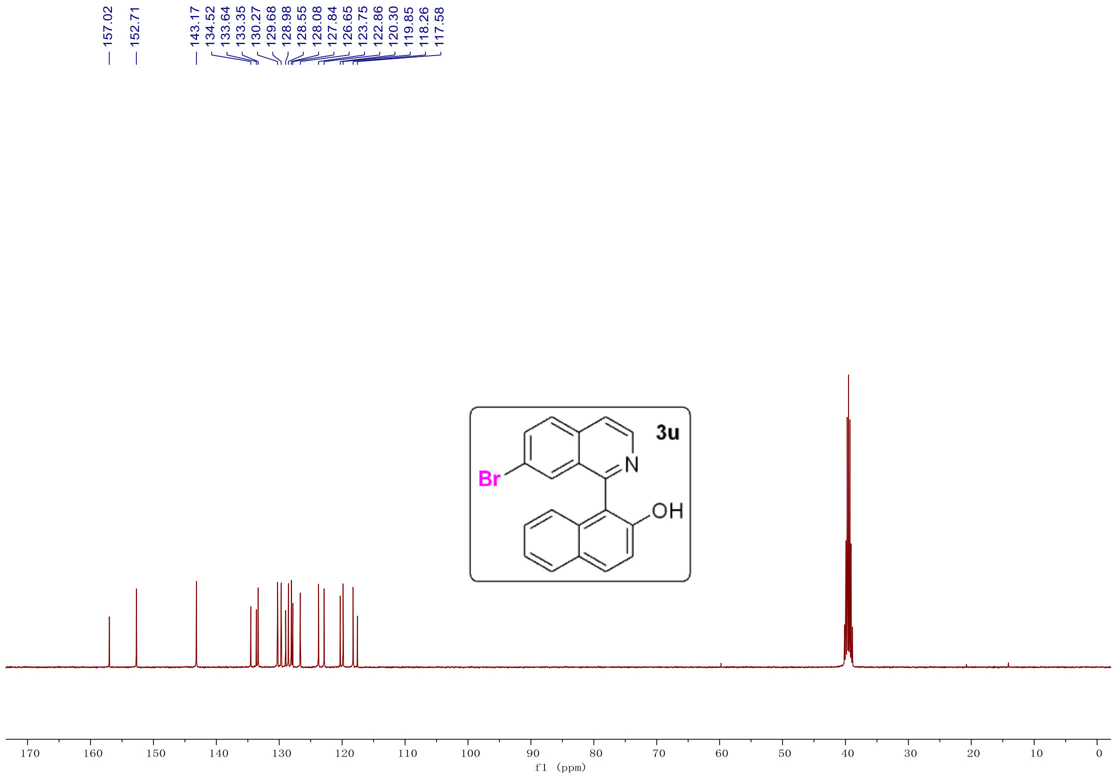

Supplementary Figure 42. ^{13}C NMR spectrum of **3u**

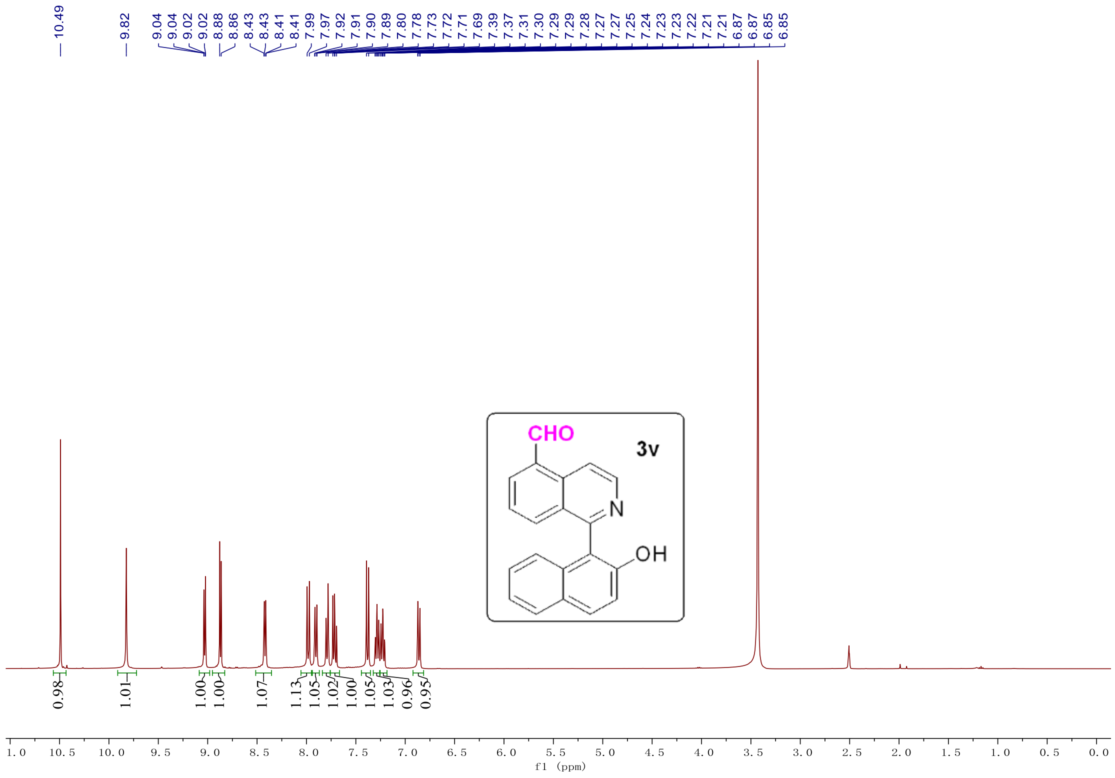

Supplementary Figure 43. ^{1}H NMR spectrum of **3v**

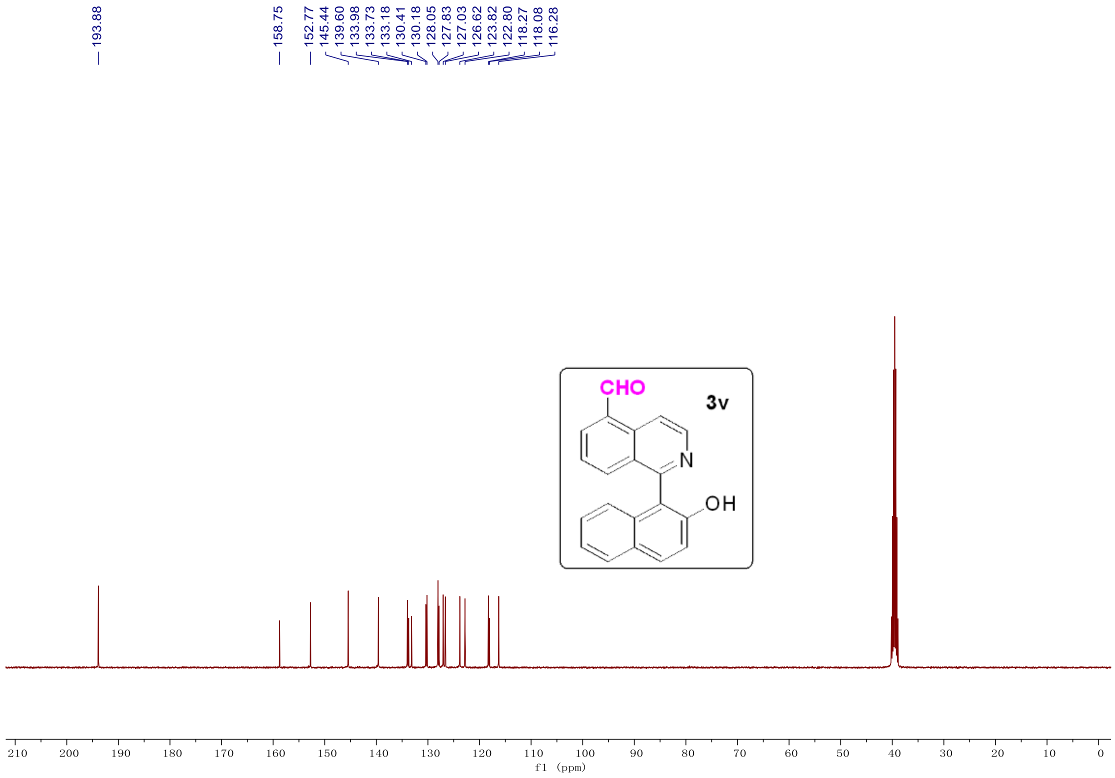

Supplementary Figure 44. ^{13}C NMR spectrum of **3v**

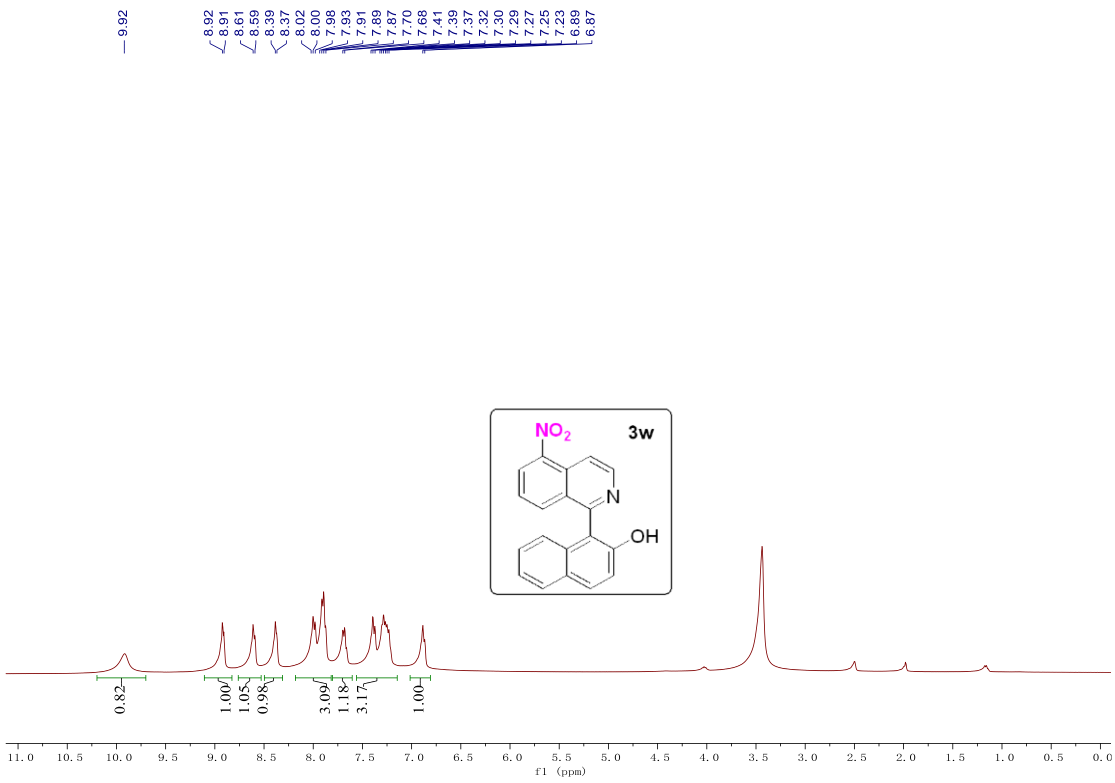

Supplementary Figure 45. ^{1}H NMR spectrum of **3w**

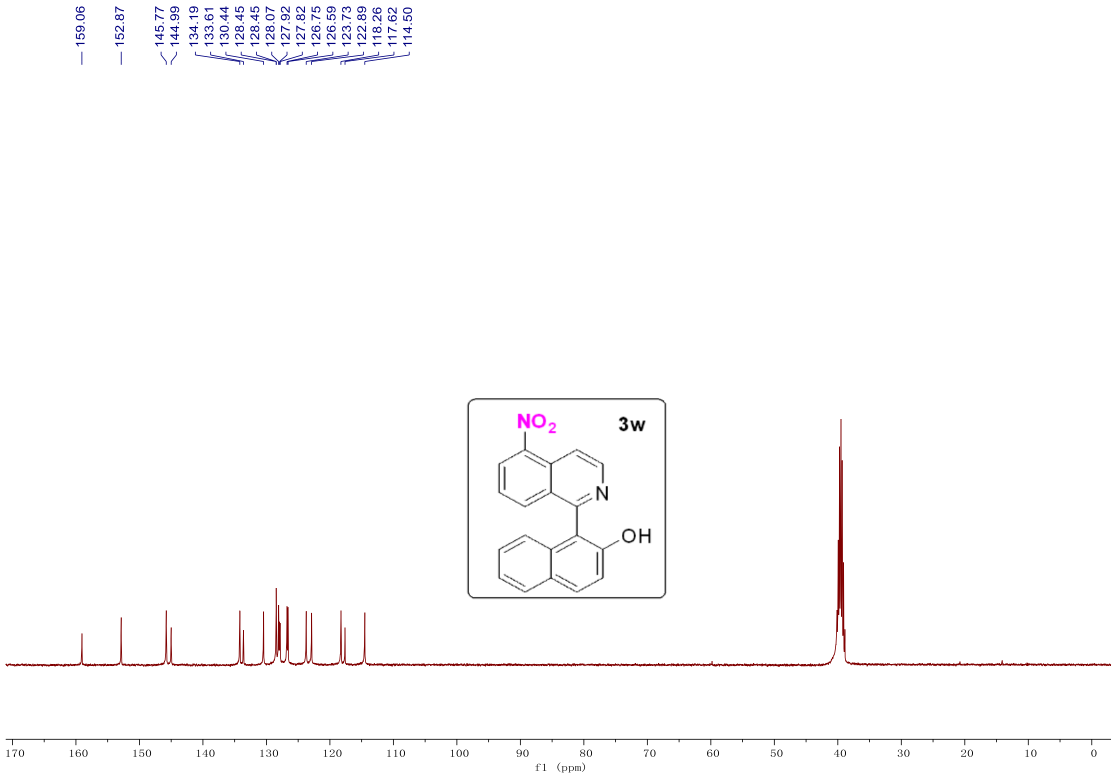

Supplementary Figure 46. ^{13}C NMR spectrum of **3w**

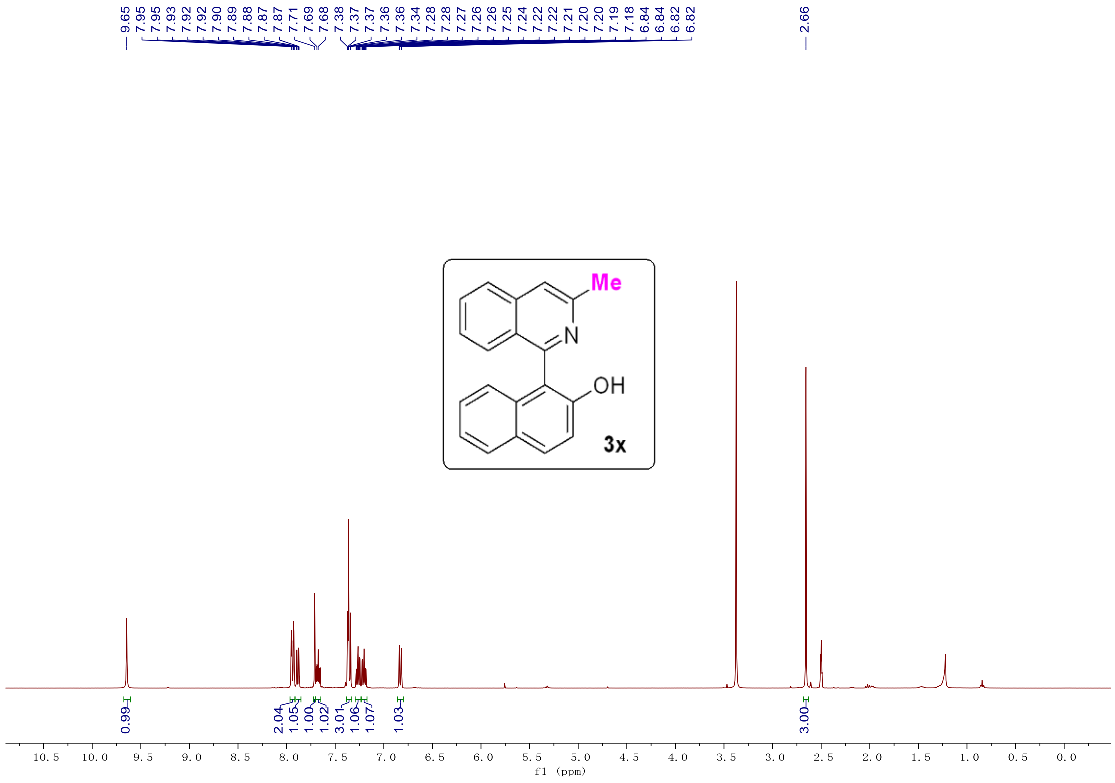

Supplementary Figure 47. ^{1}H NMR spectrum of **3x**

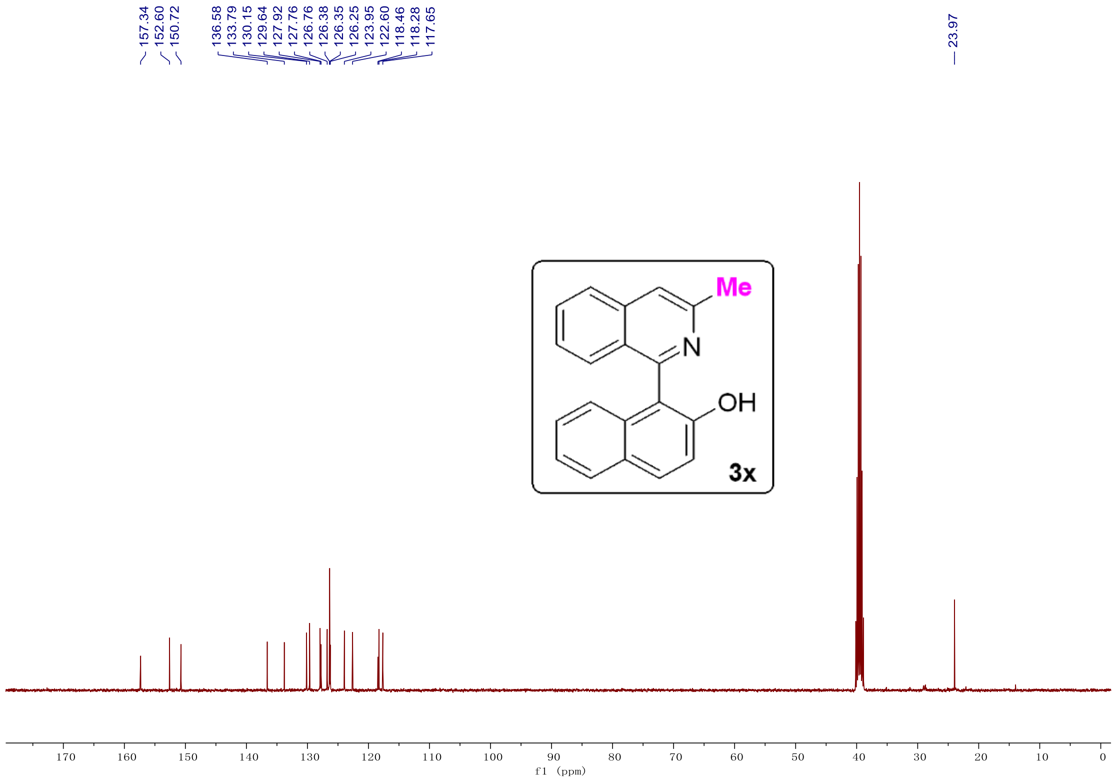

Supplementary Figure 48. ^{13}C NMR spectrum of **3x**

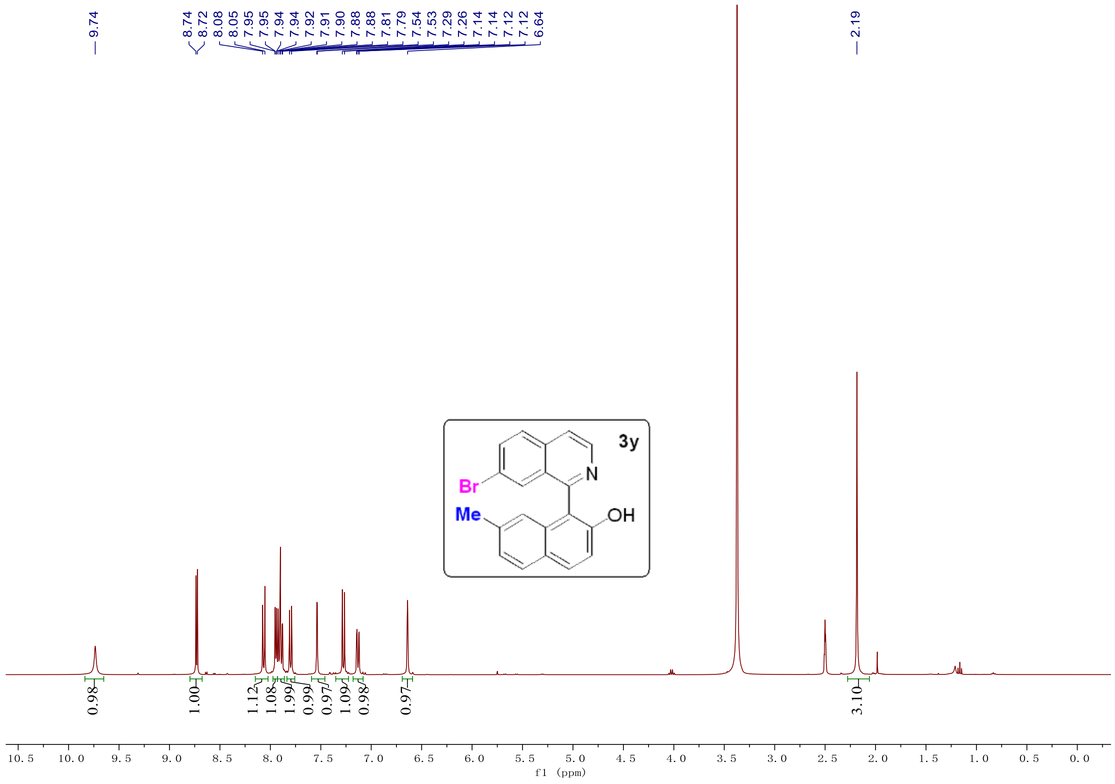

Supplementary Figure 49. ^{1}H NMR spectrum of **3y**

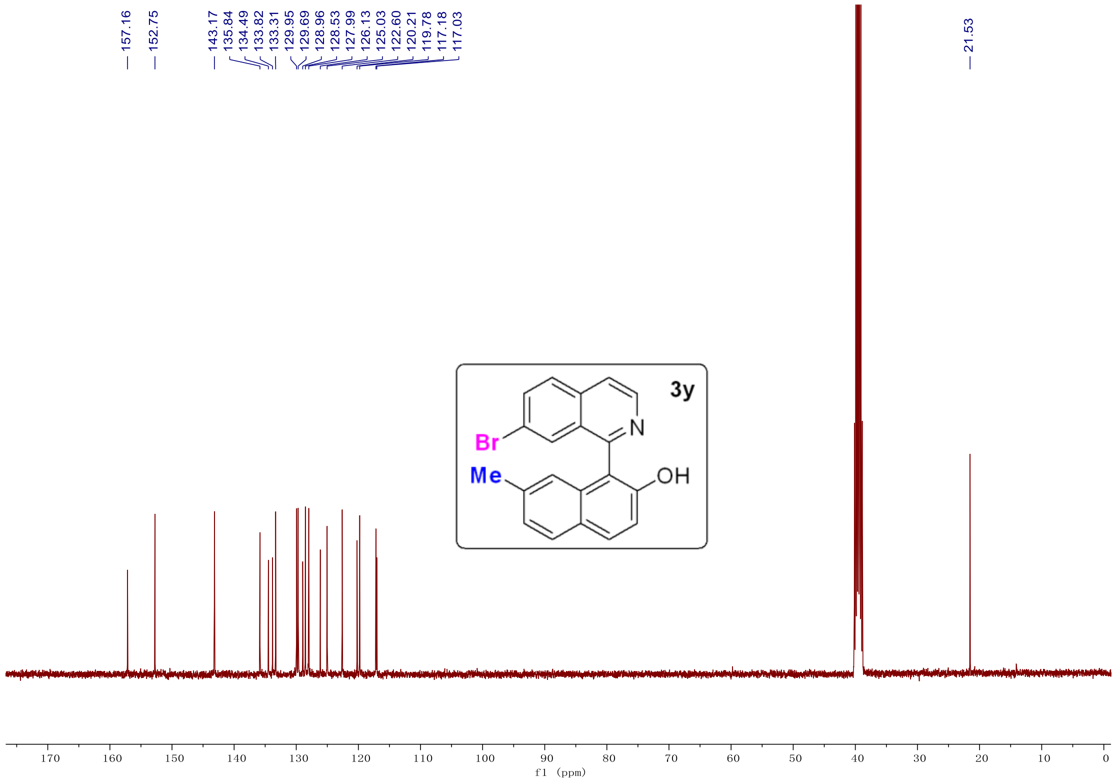

Supplementary Figure 50. ^{13}C NMR spectrum of **3y**

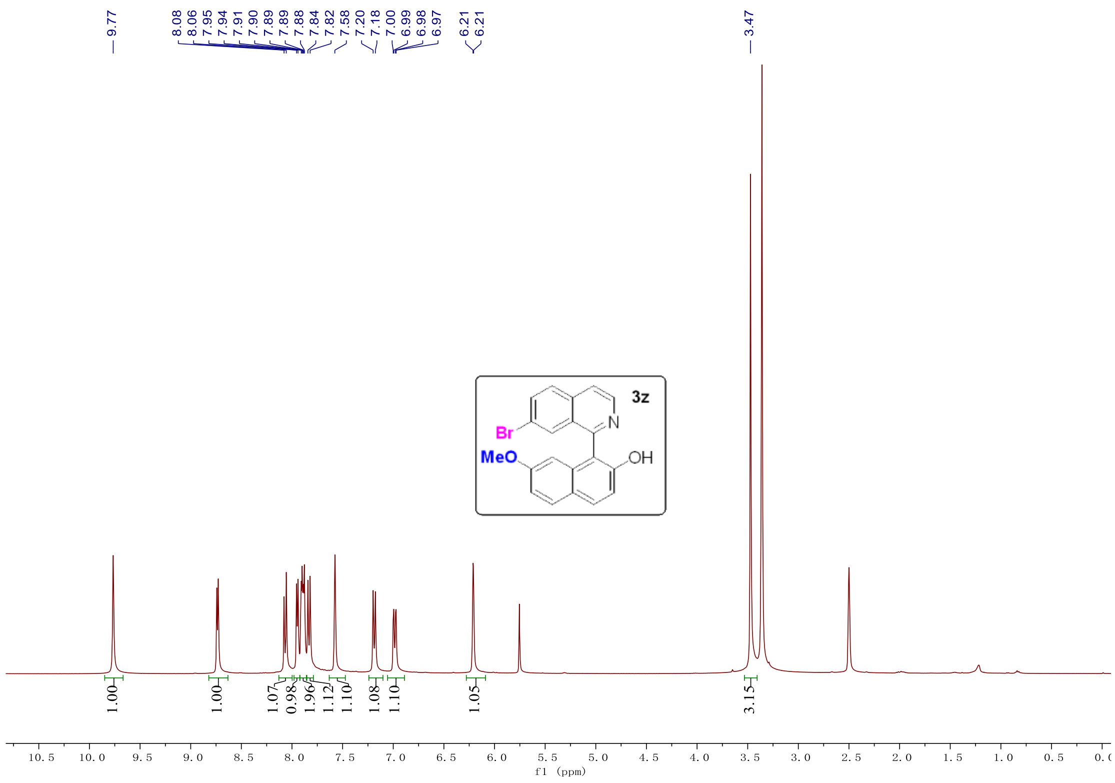

Supplementary Figure 51. ^{1}H NMR spectrum of **3z**

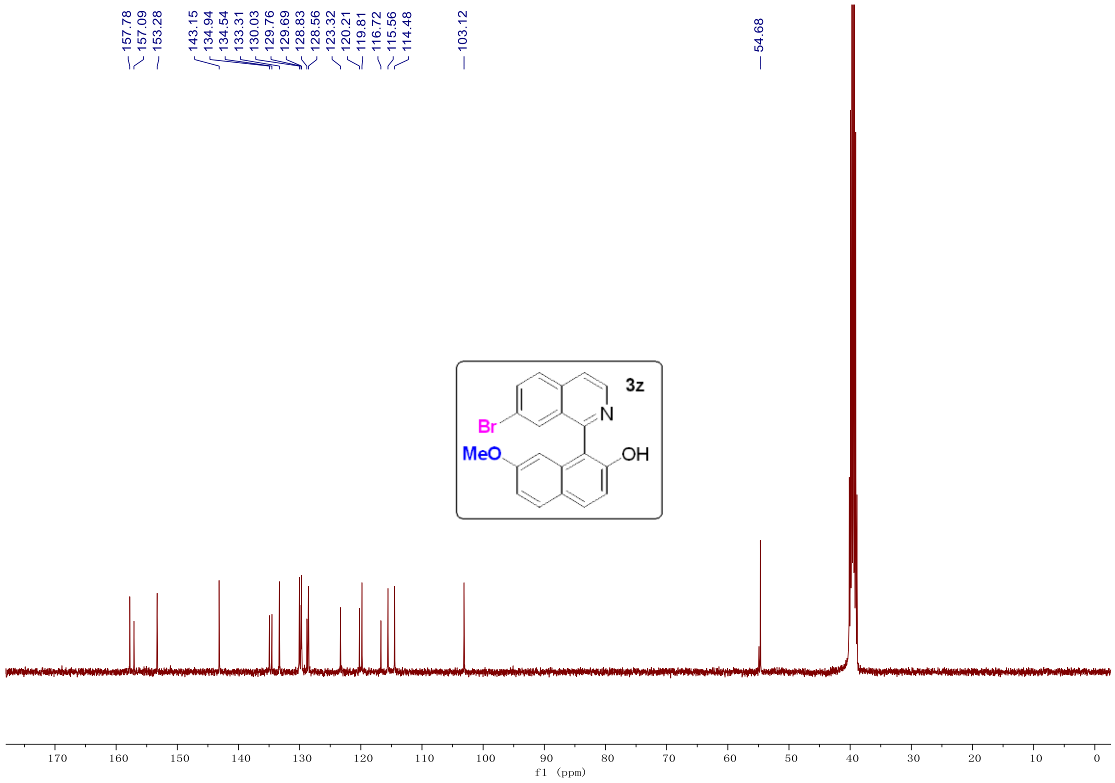

Supplementary Figure 52. ^{13}C NMR spectrum of **3z**

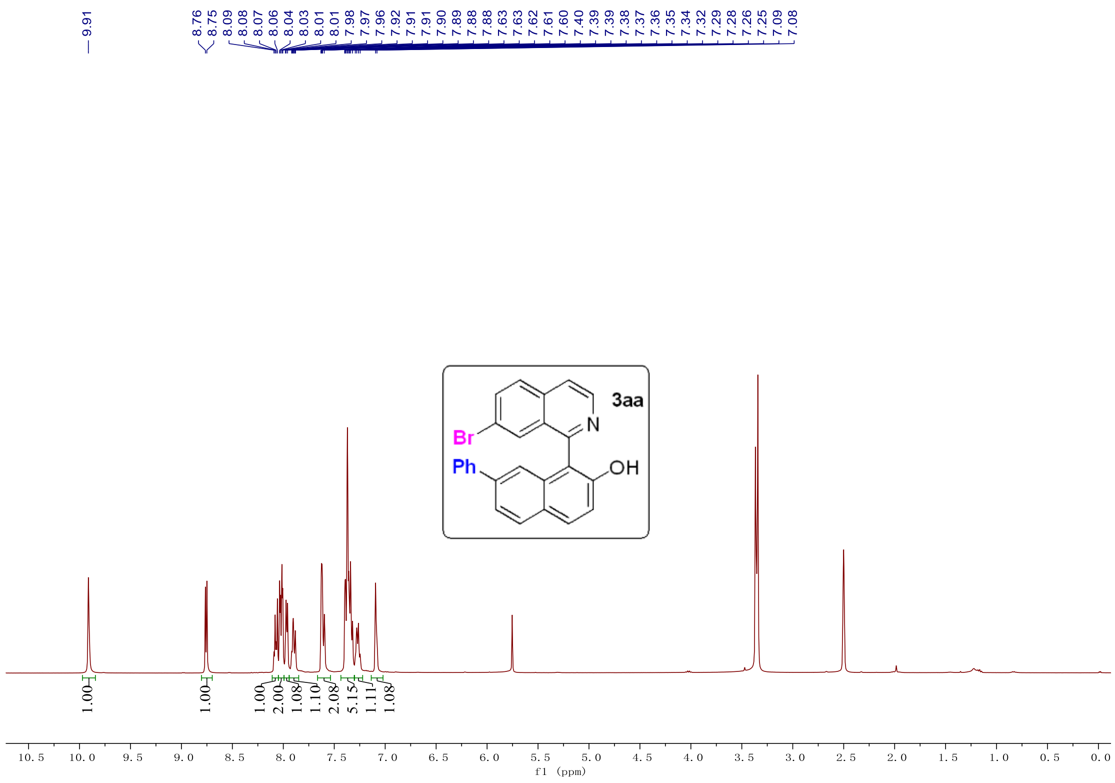

Supplementary Figure 53. ^{1}H NMR spectrum of **3aa**

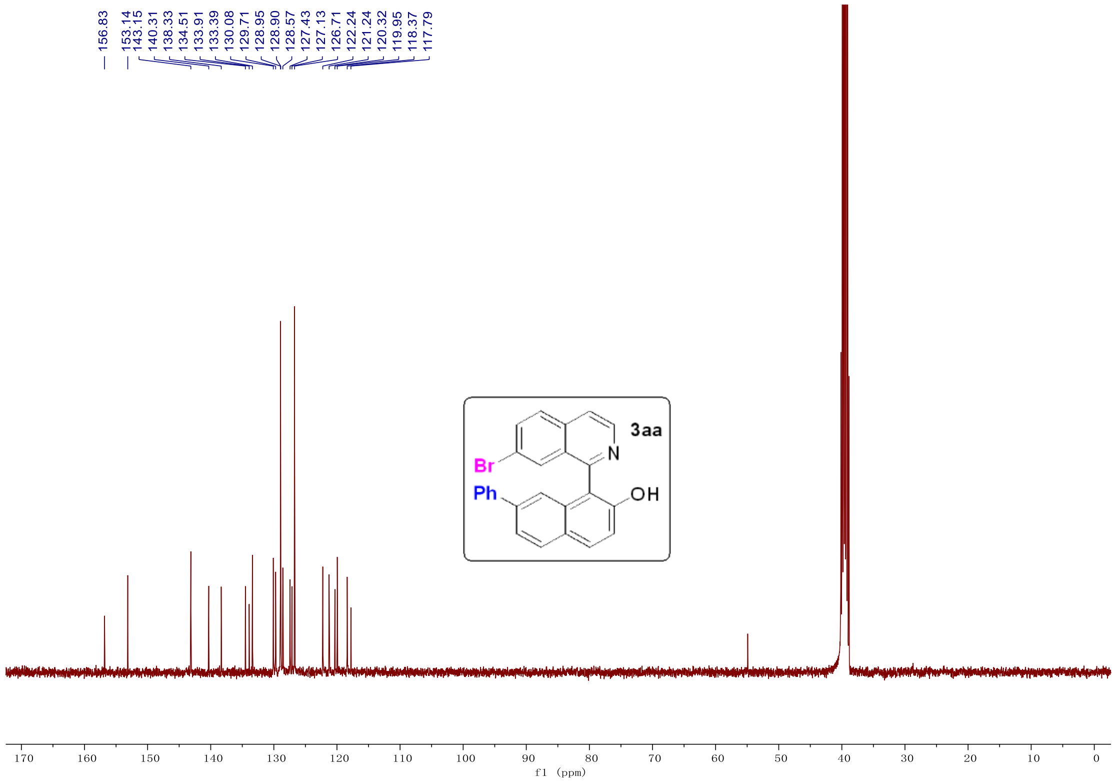

Supplementary Figure 54. ^{13}C NMR spectrum of **3aa**

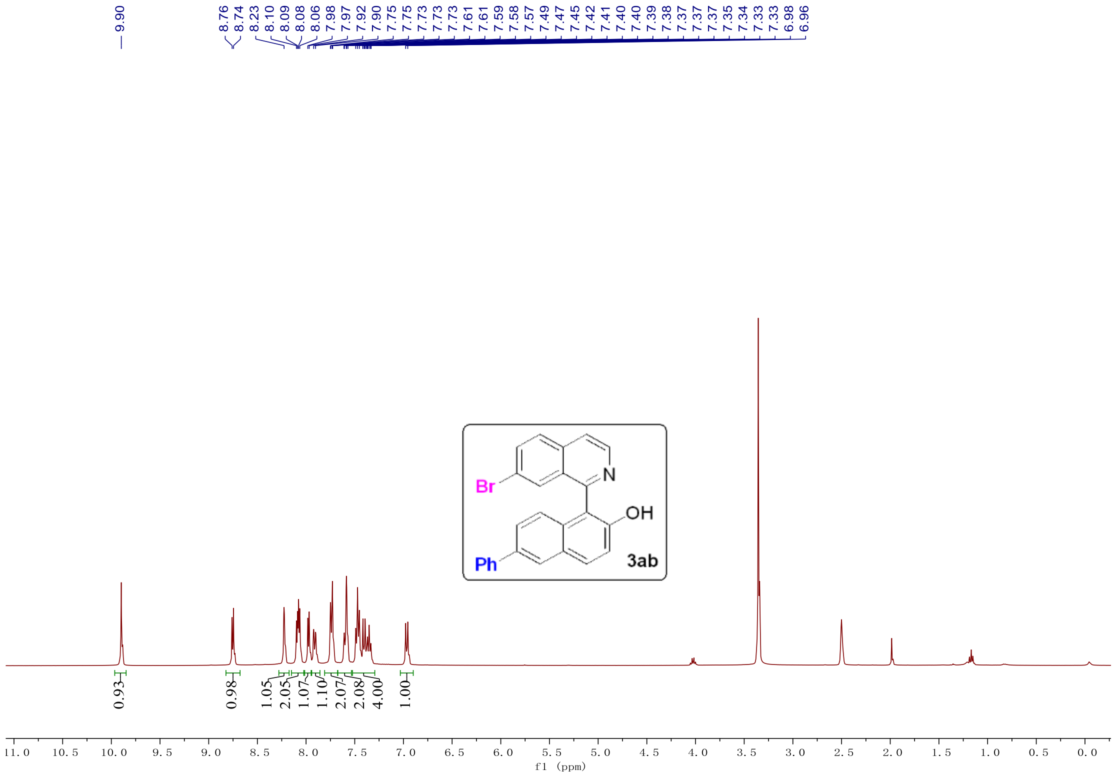

Supplementary Figure 55. ^{1}H NMR spectrum of **3ab**

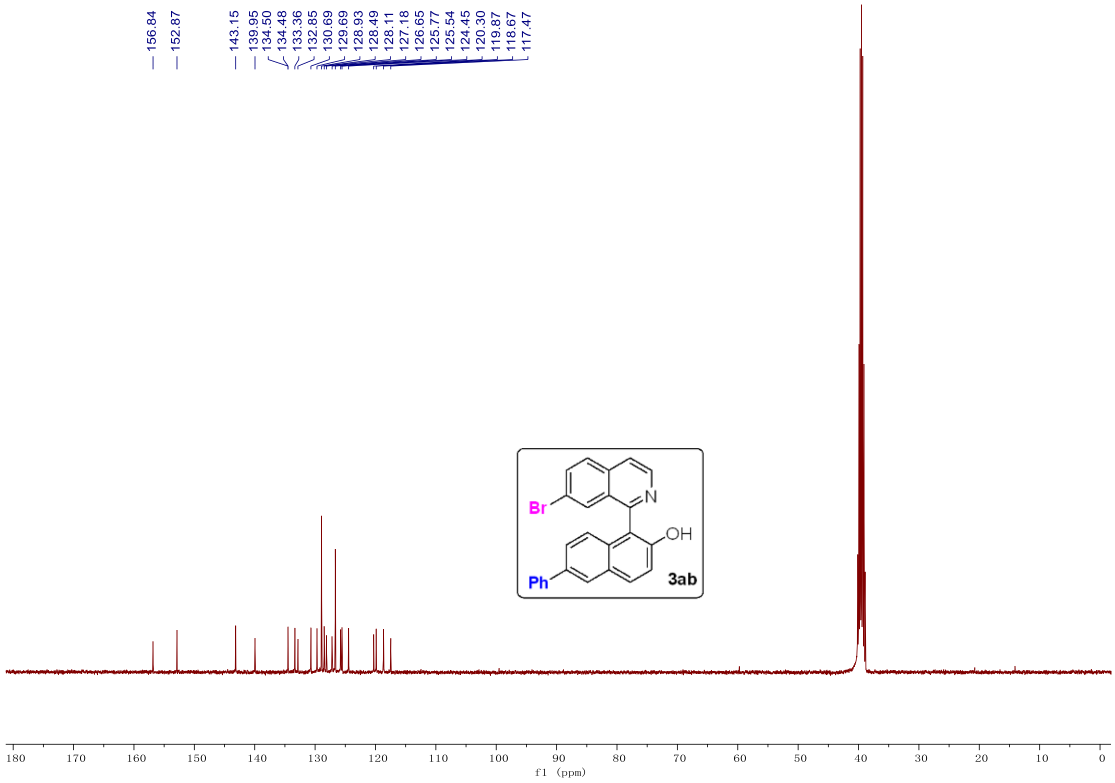

Supplementary Figure 56. ^{13}C NMR spectrum of **3ab**

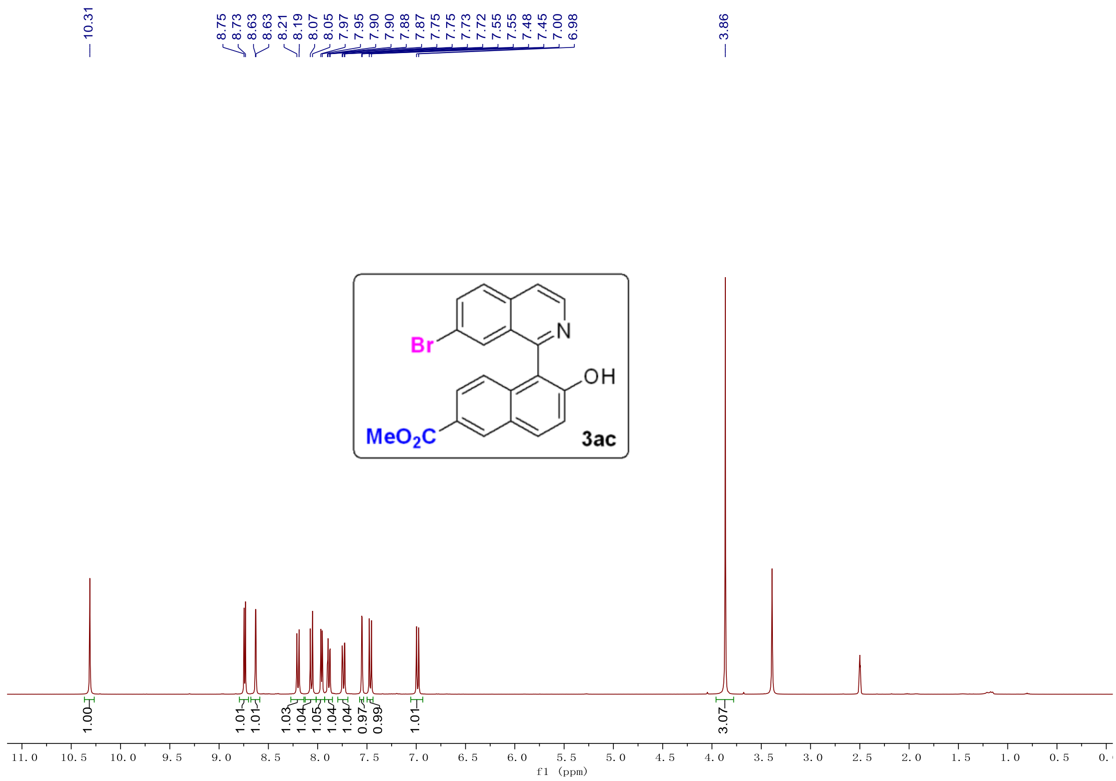

Supplementary Figure 57. ^{1}H NMR spectrum of **3ac**

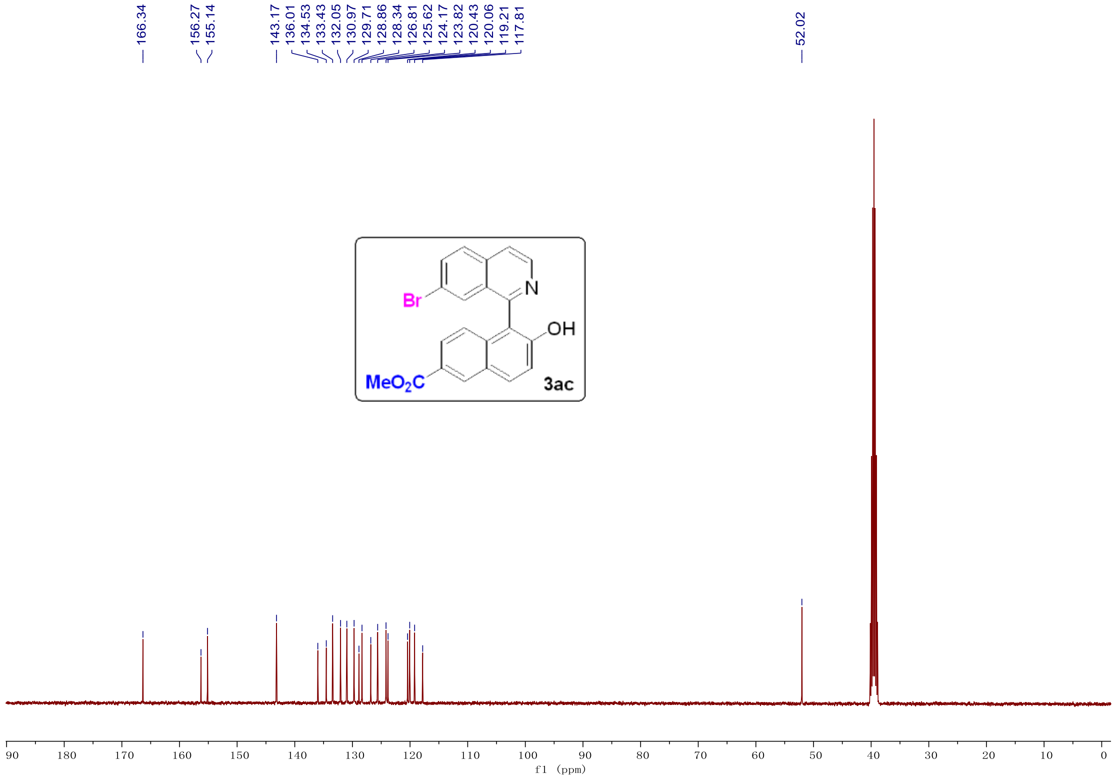

Supplementary Figure 58. ^{13}C NMR spectrum of **3ac**

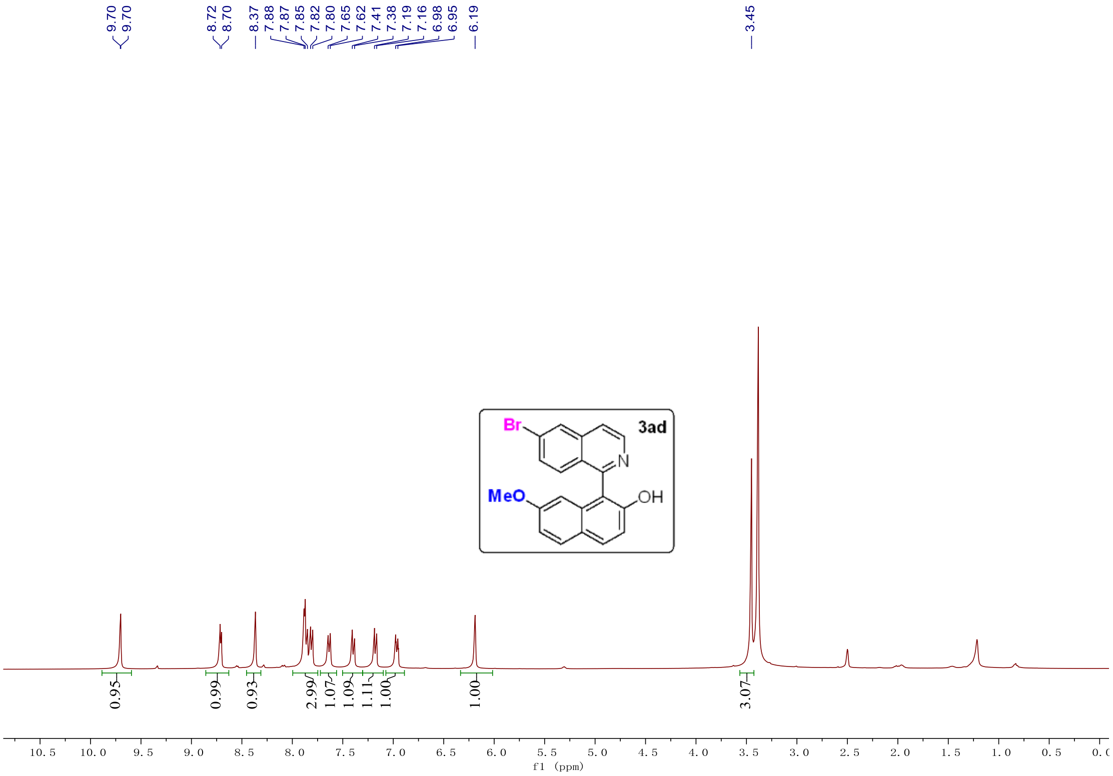

Supplementary Figure 59. ^{1}H NMR spectrum of **3ad**

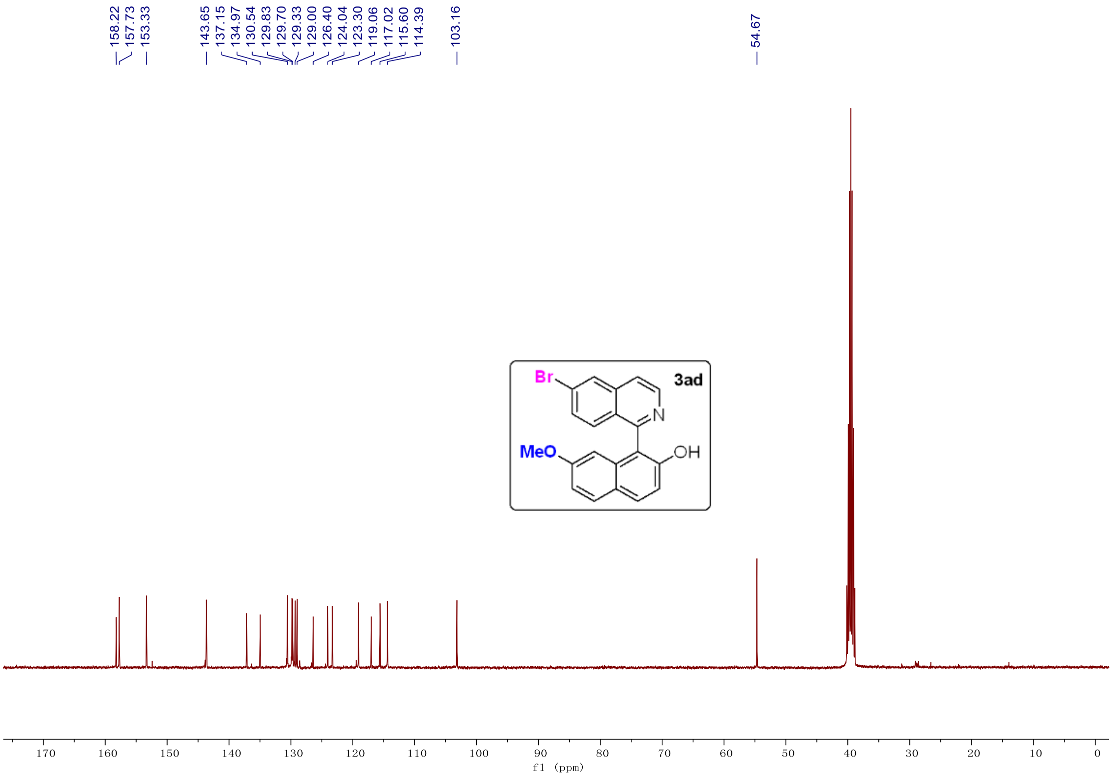

Supplementary Figure 60. ^{13}C NMR spectrum of **3ad**

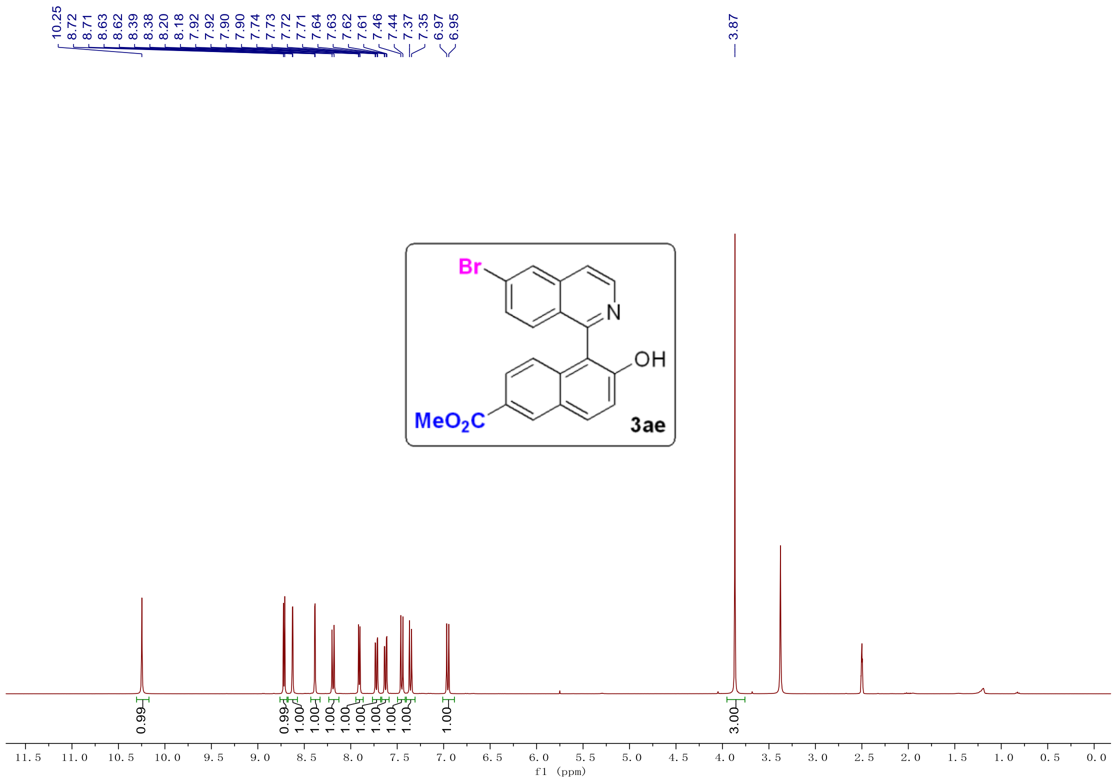

Supplementary Figure 61. ^{1}H NMR spectrum of **3ae**

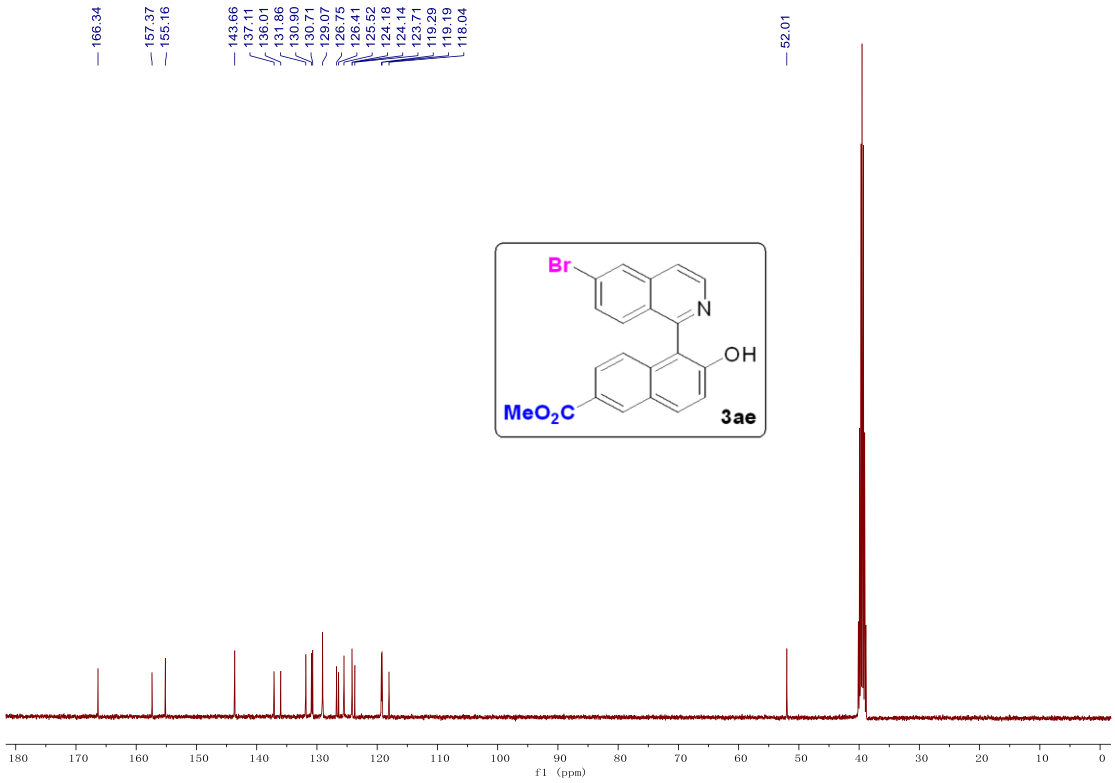

Supplementary Figure 62. ^{13}C NMR spectrum of **3ae**

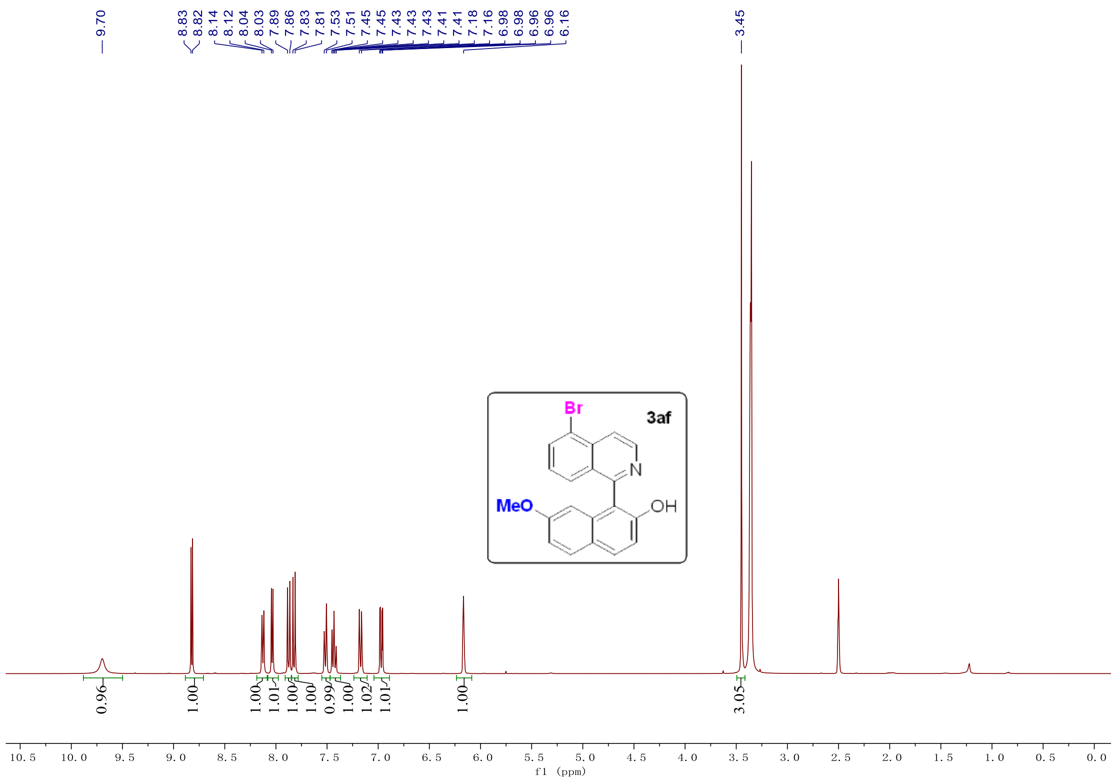

Supplementary Figure 63. ^{1}H NMR spectrum of **3af**

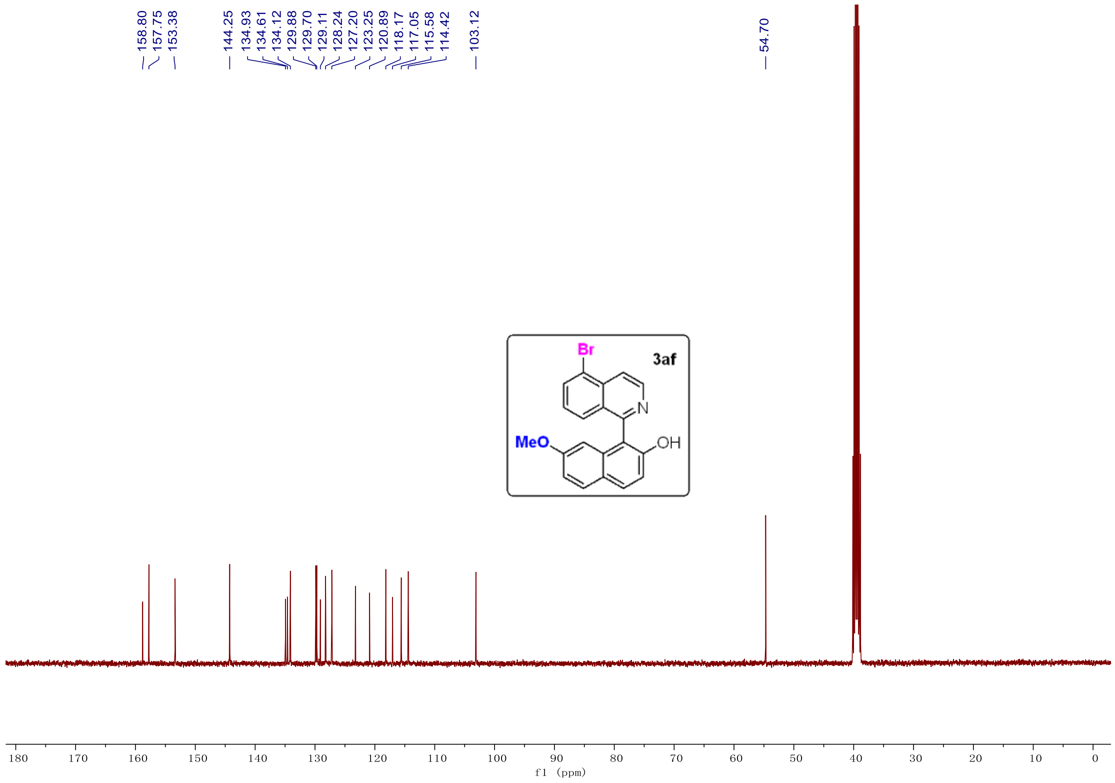

Supplementary Figure 64. ^{13}C NMR spectrum of **3af**

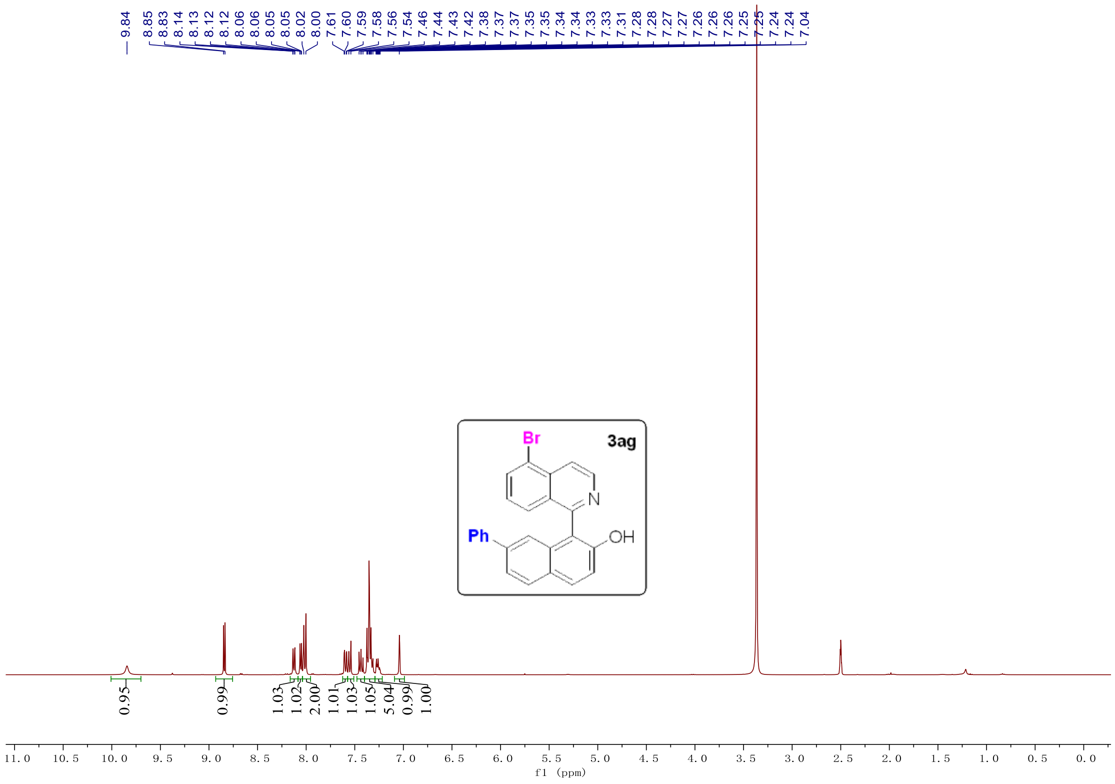

Supplementary Figure 65. ^{1}H NMR spectrum of **3ag**

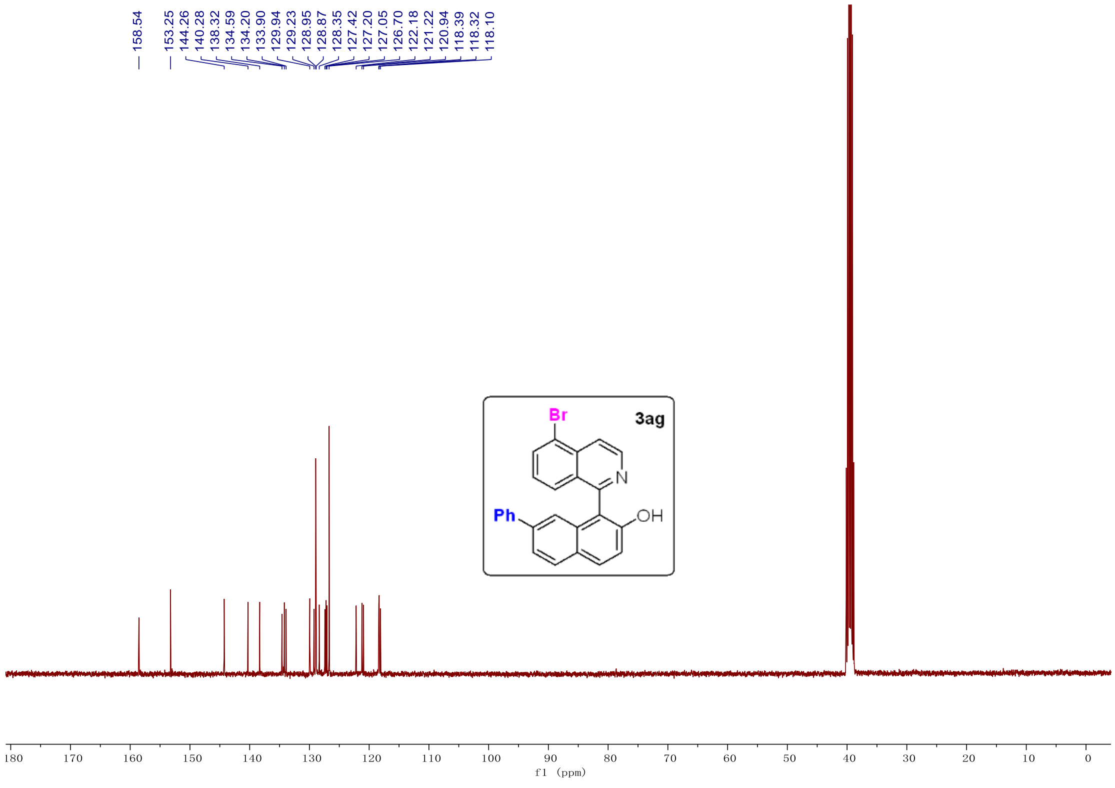

Supplementary Figure 66. ^{13}C NMR spectrum of **3ag**

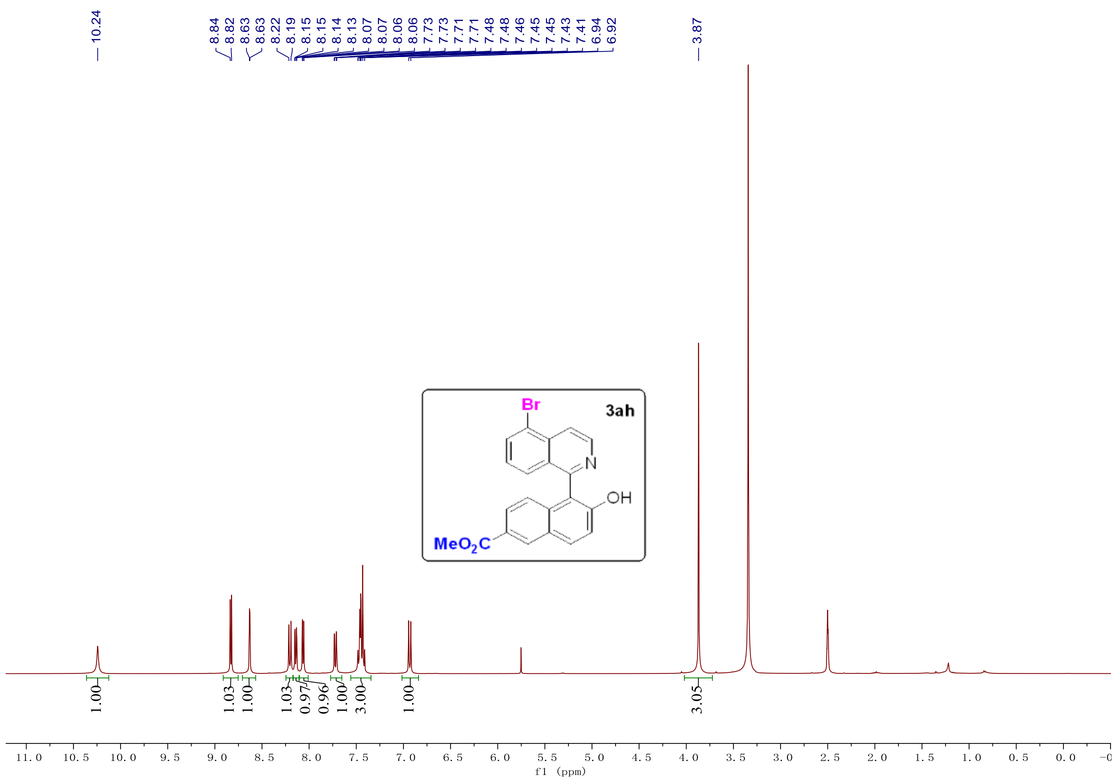

Supplementary Figure 67. ^{1}H NMR spectrum of **3ah**

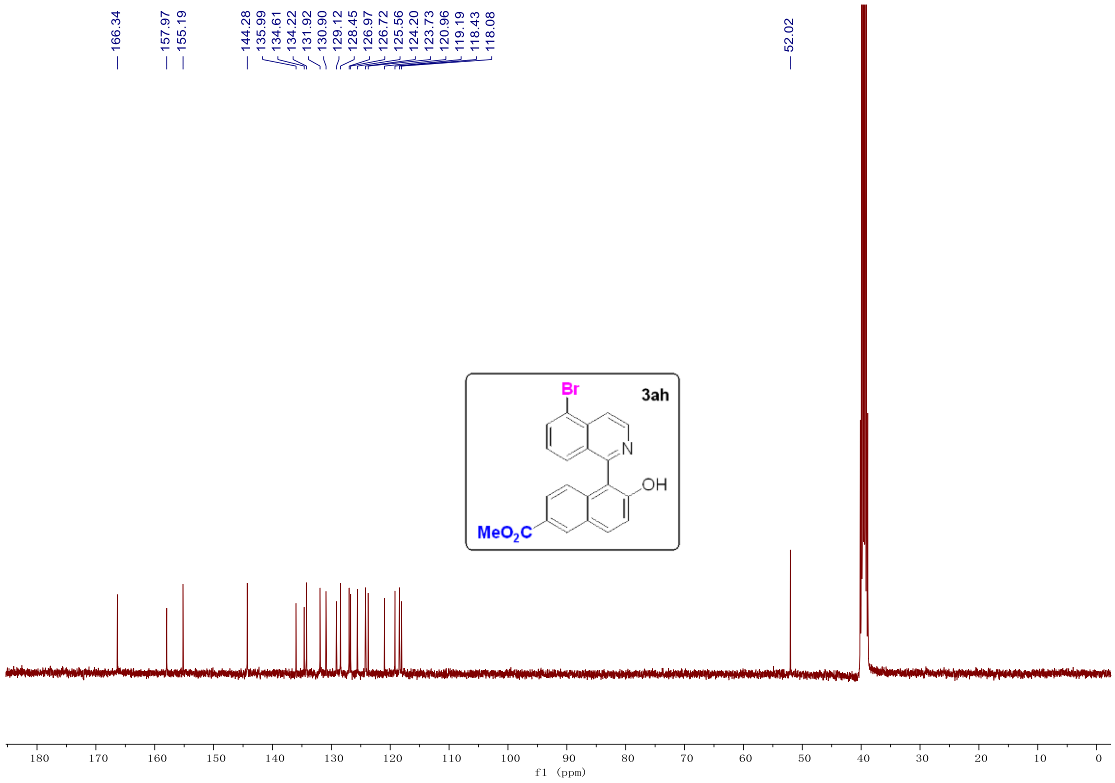

Supplementary Figure 68. ^{13}C NMR spectrum of **3ah**

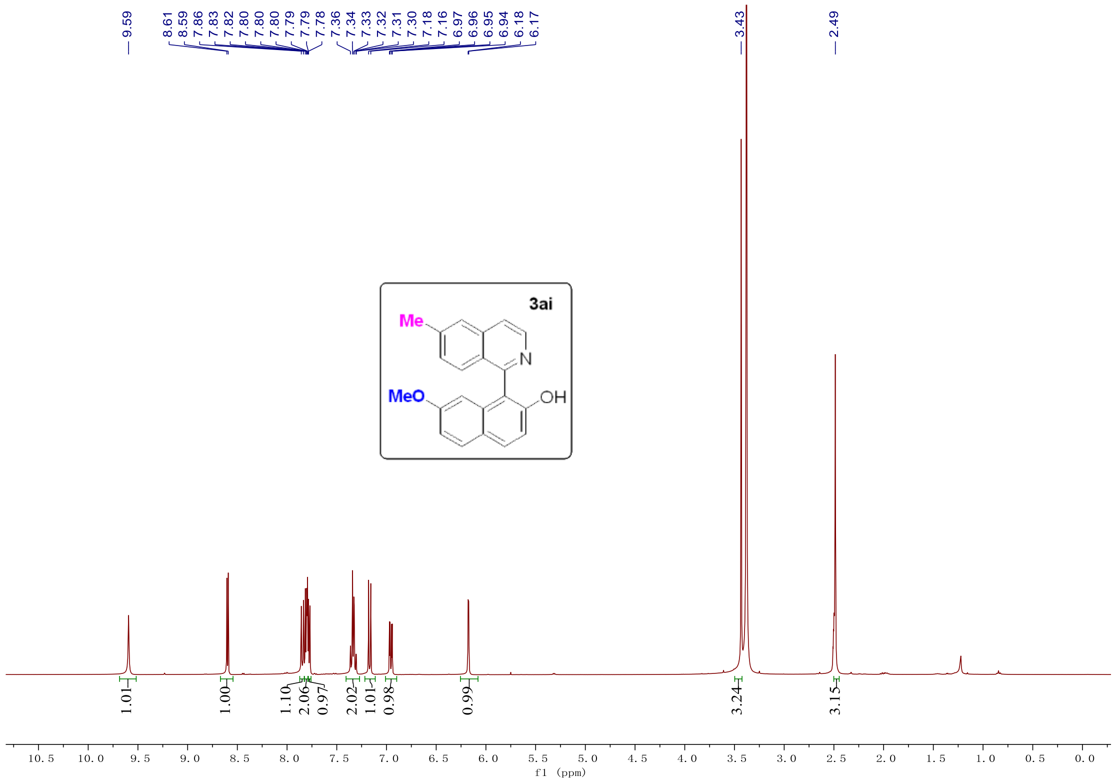

Supplementary Figure 69. ^{1}H NMR spectrum of **3ai**

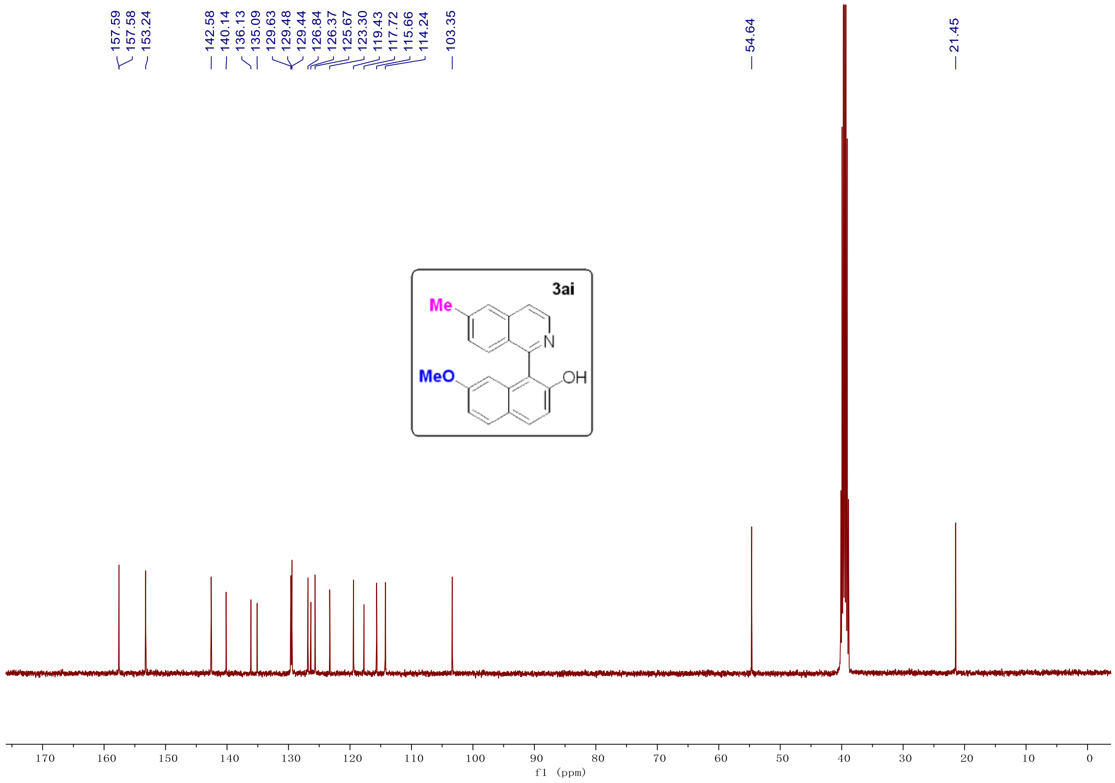

Supplementary Figure 70. ^{13}C NMR spectrum of **3ai**

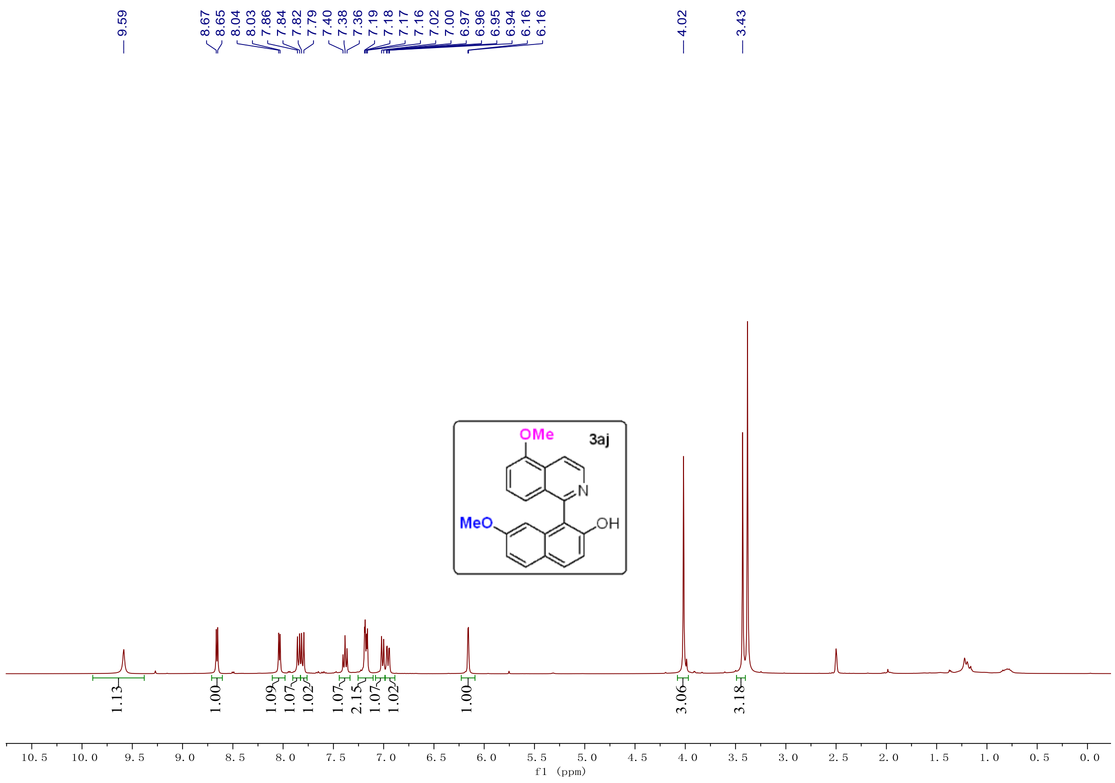

Supplementary Figure 71. ^{1}H NMR spectrum of **3aj**

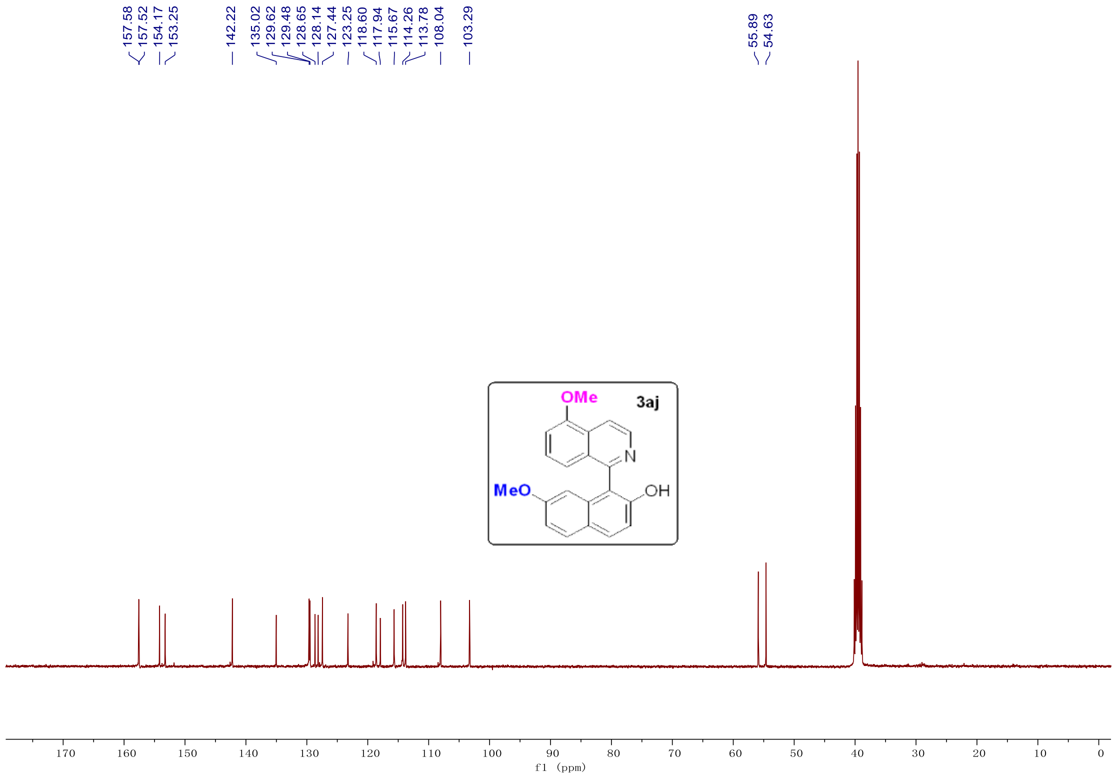

Supplementary Figure 72. ^{13}C NMR spectrum of **3aj**

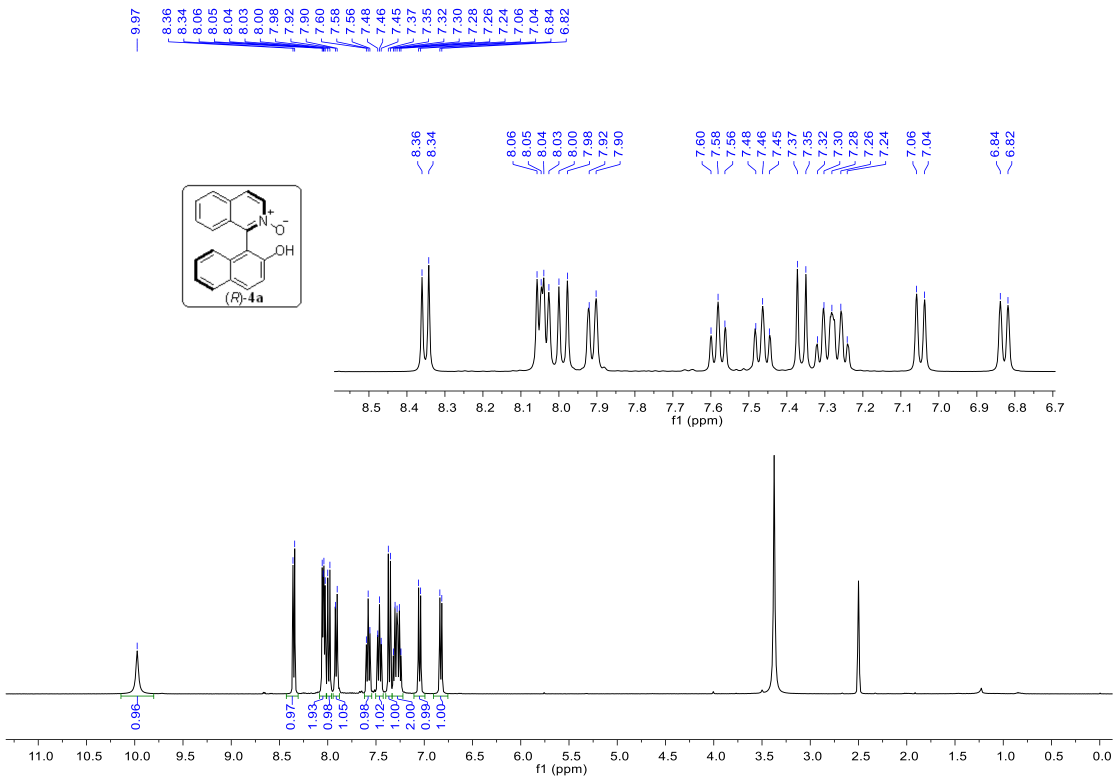

Supplementary Figure 73. ^{1}H NMR spectrum of (*R*)-**4a**

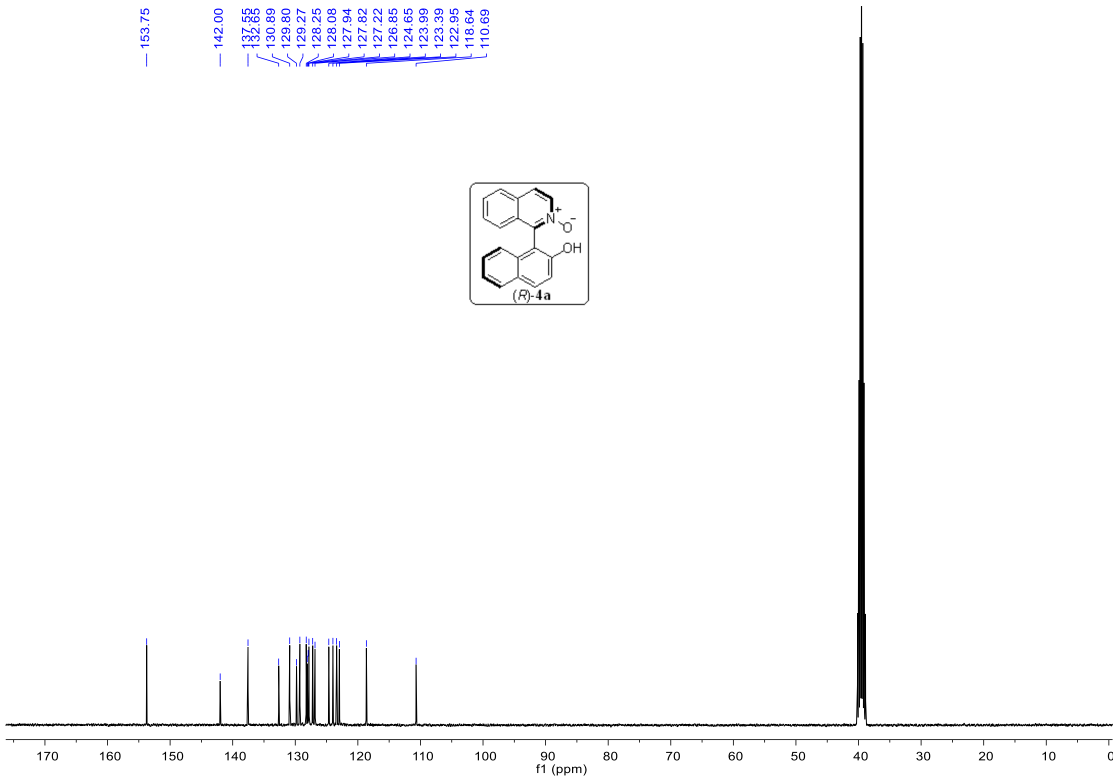

Supplementary Figure 74. ^{13}C NMR spectrum of (*R*)-**4a**

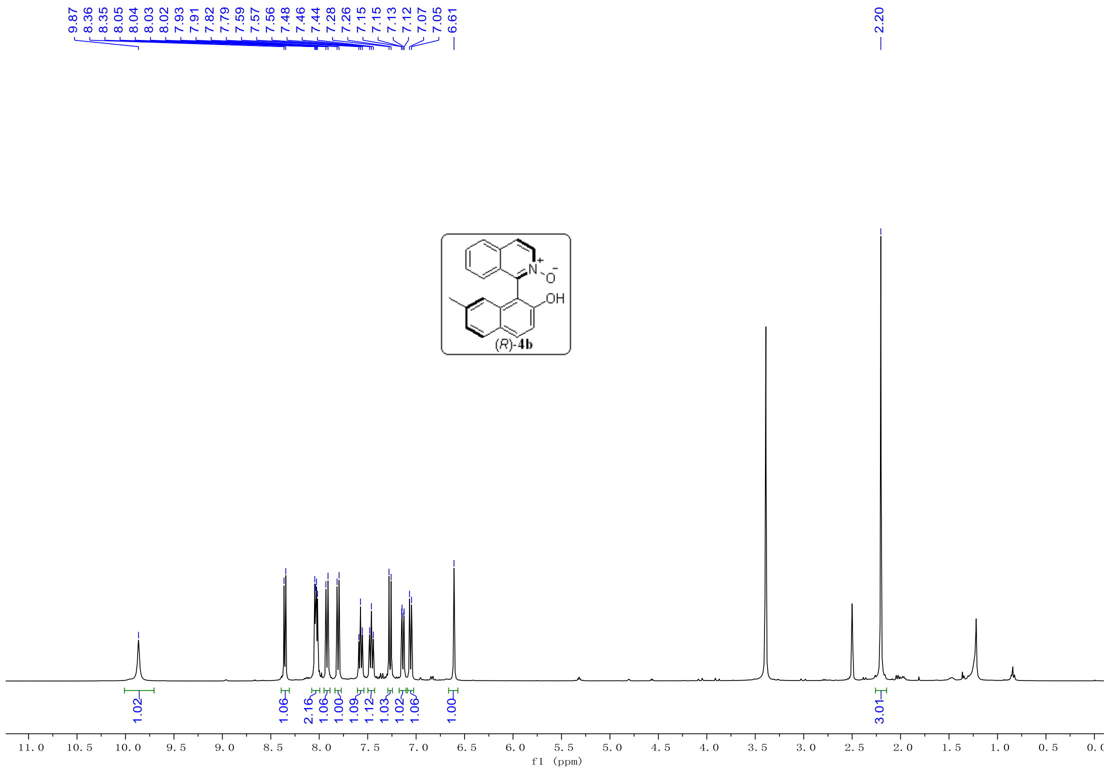

Supplementary Figure 75. ^{1}H NMR spectrum of (*R*)-**4b**

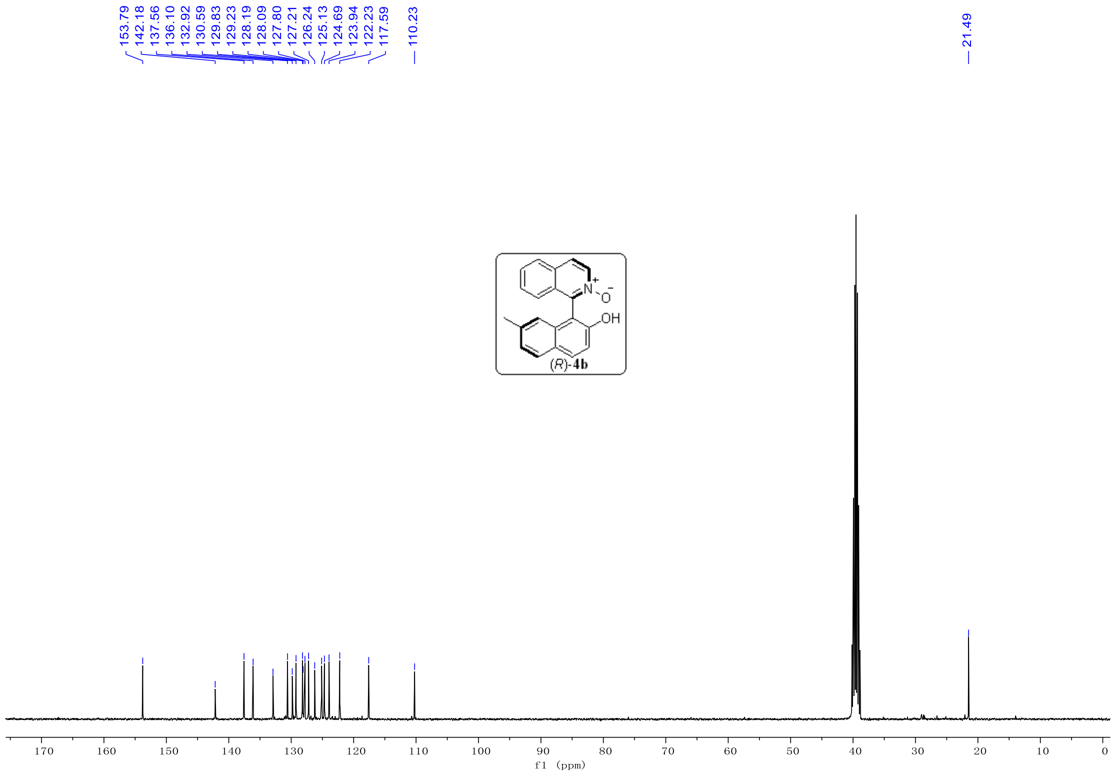

Supplementary Figure 76. ^{13}C NMR spectrum of (*R*)-**4b**

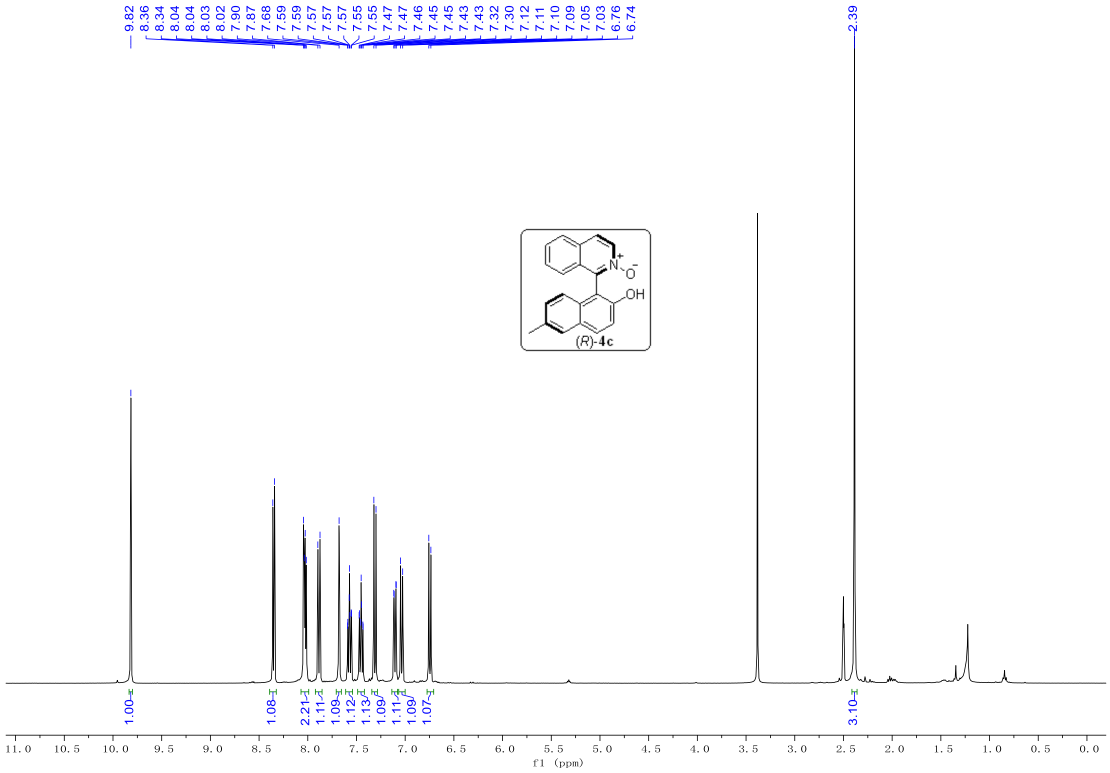

Supplementary Figure 77. ^{1}H NMR spectrum of (*R*)-**4c**

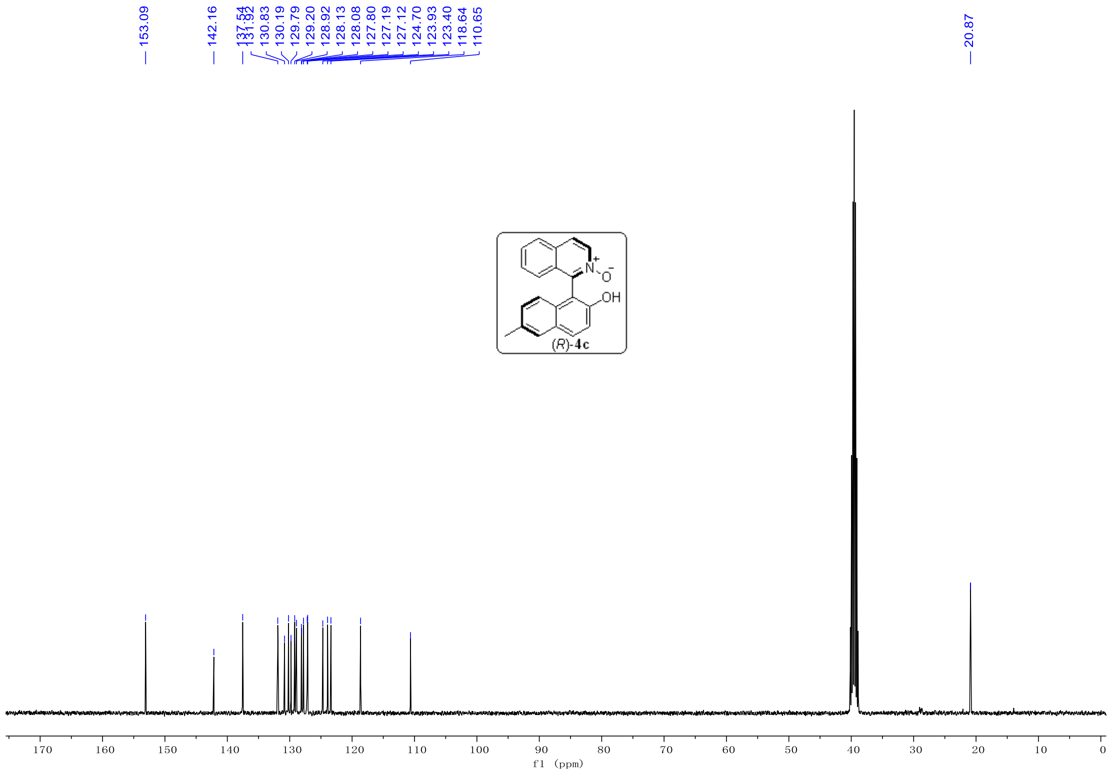

Supplementary Figure 78. ^{13}C NMR spectrum of (*R*)-**4c**

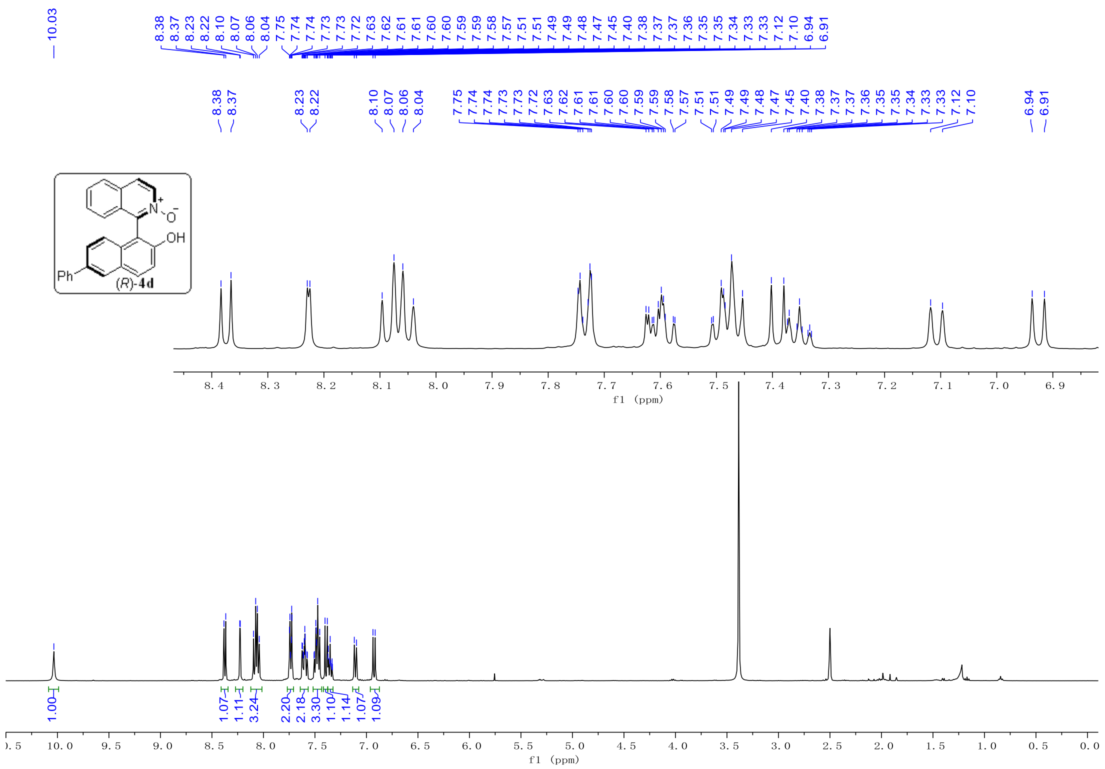

Supplementary Figure 79. ^{1}H NMR spectrum of (*R*)-**4d**

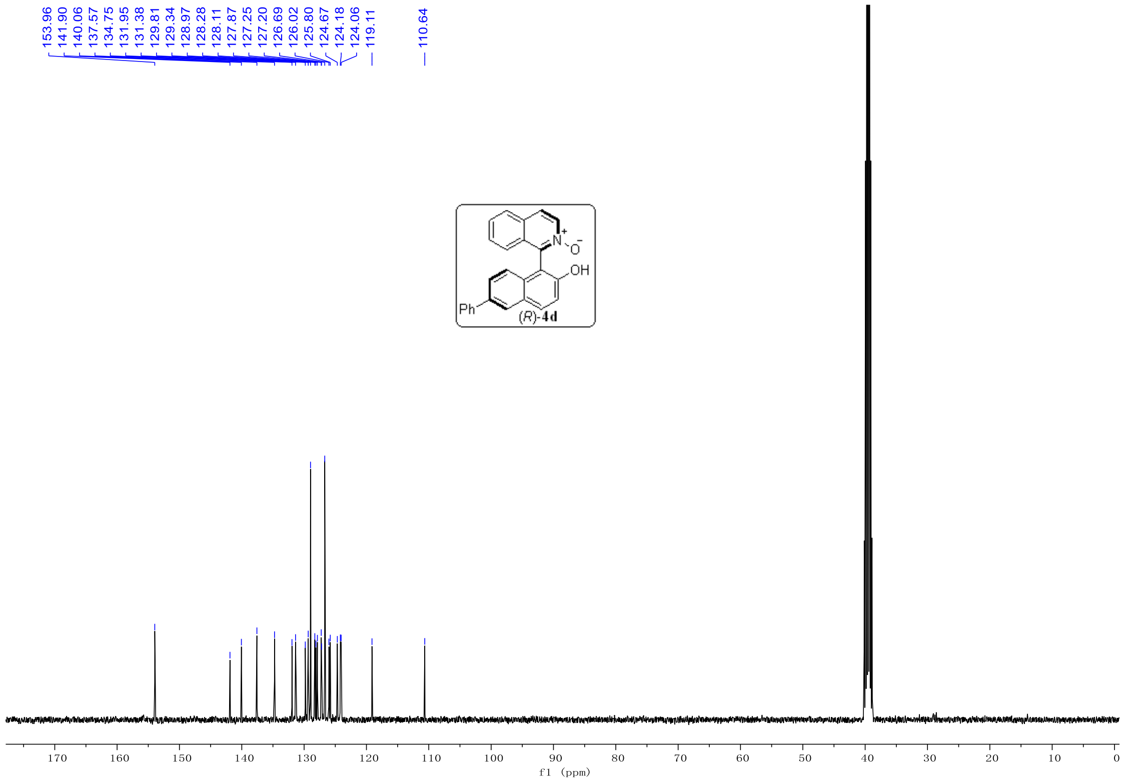

Supplementary Figure 80. ^{13}C NMR spectrum of (*R*)-**4d**

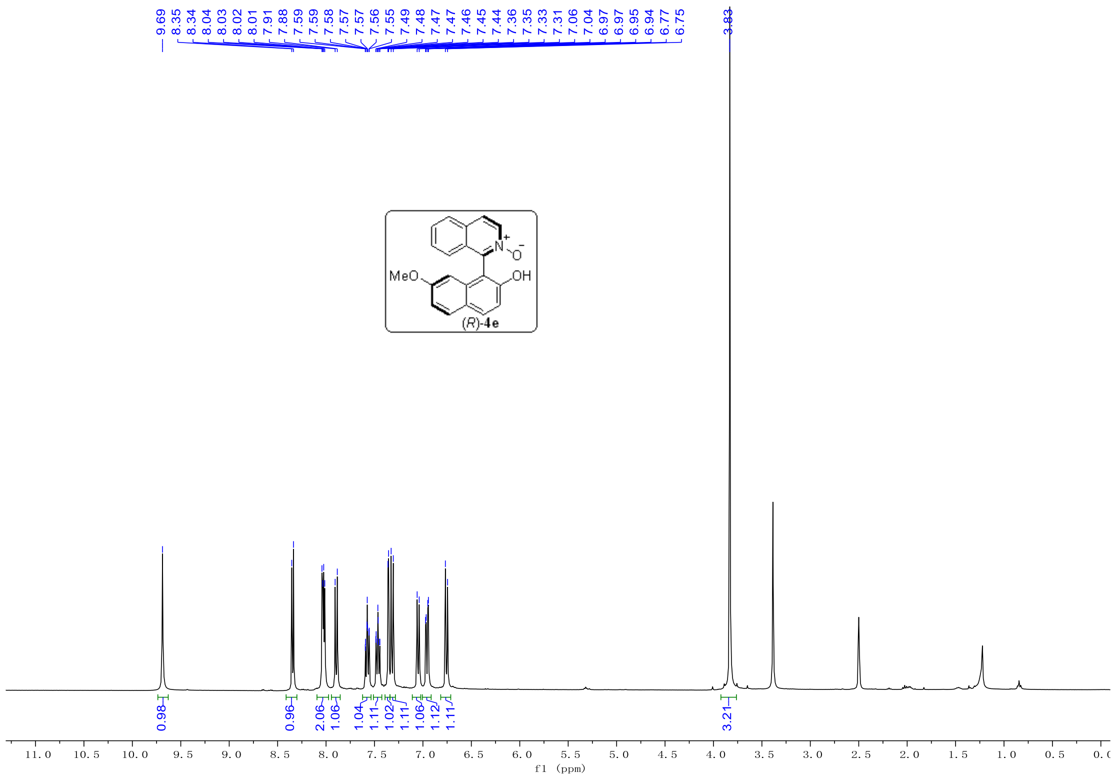

Supplementary Figure 81. ^{1}H NMR spectrum of (*R*)-**4e**

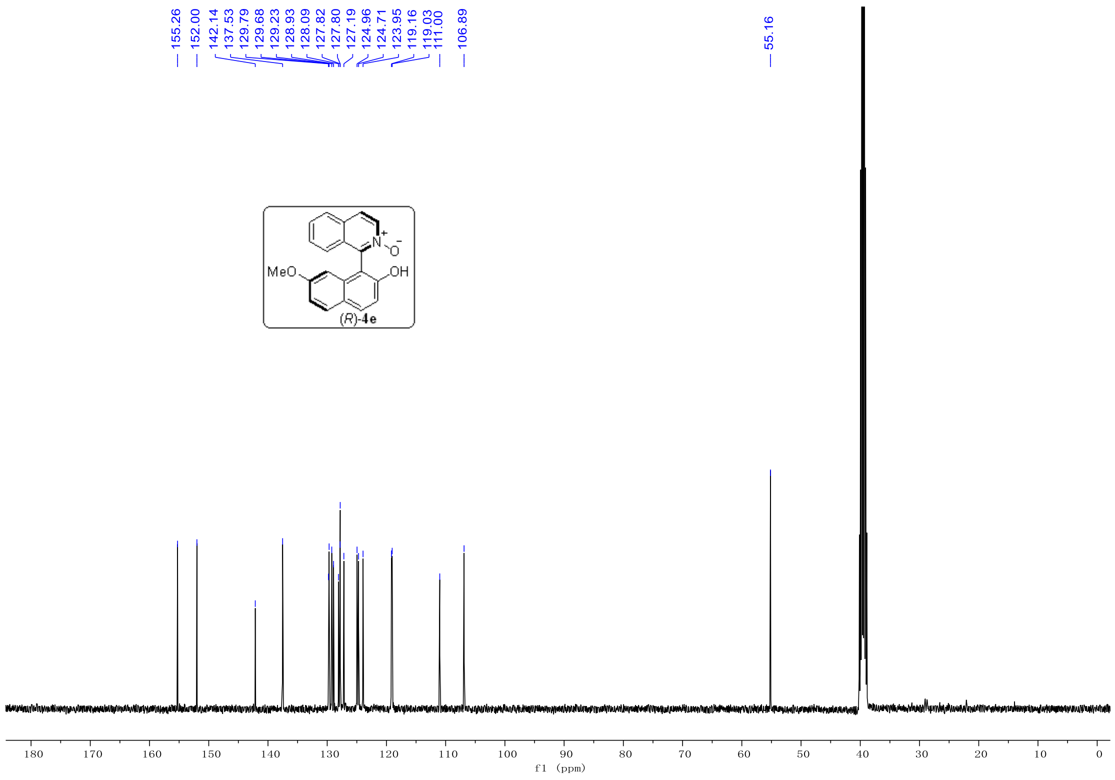

Supplementary Figure 82. ^{13}C NMR spectrum of (*R*)-**4e**

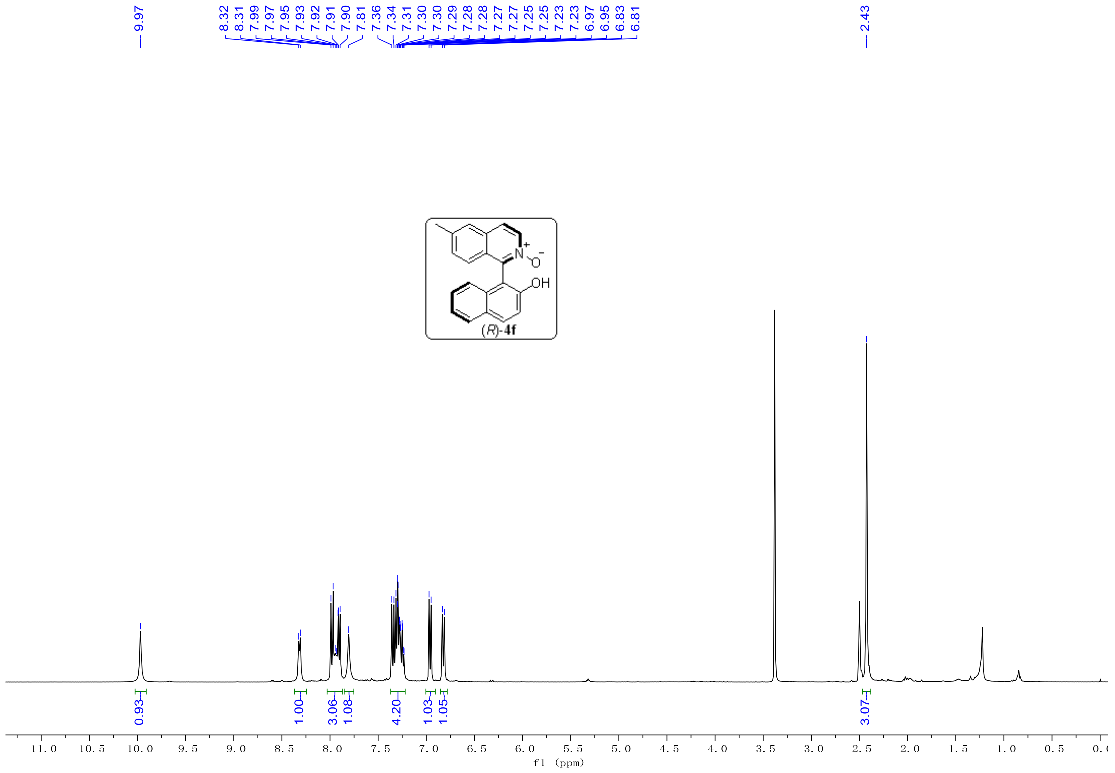

Supplementary Figure 83. ^{1}H NMR spectrum of (*R*)-**4f**

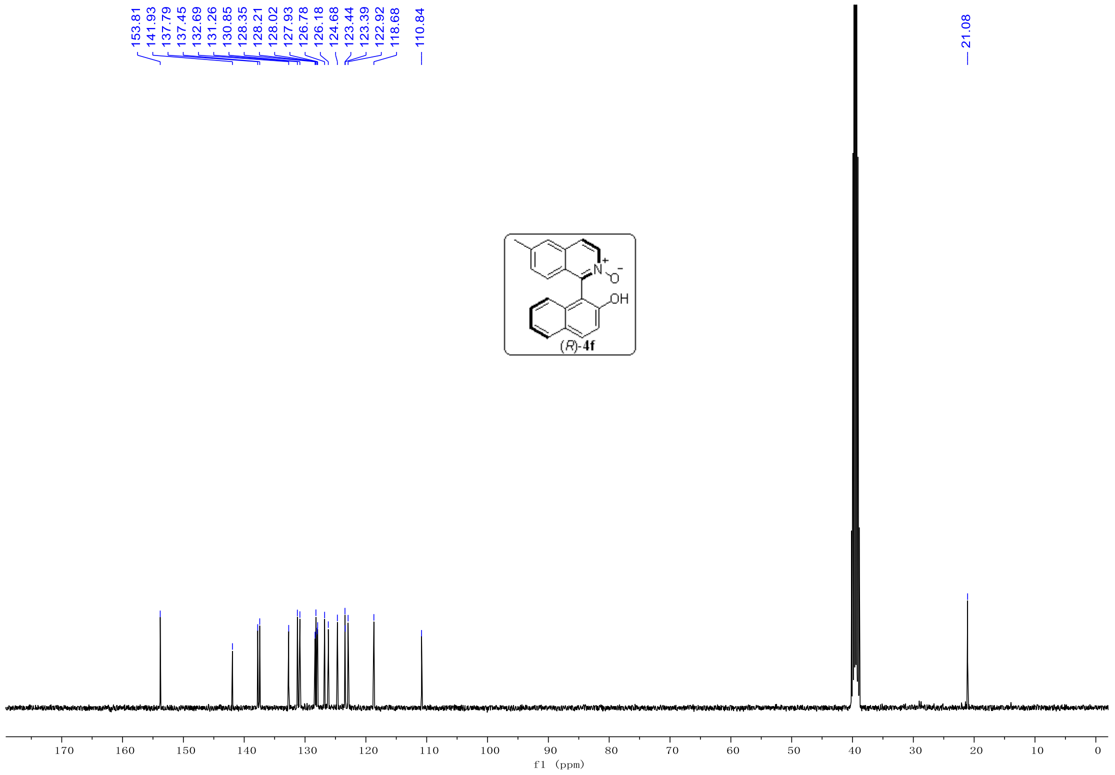

Supplementary Figure 84. ^{13}C NMR spectrum of (*R*)-**4f**

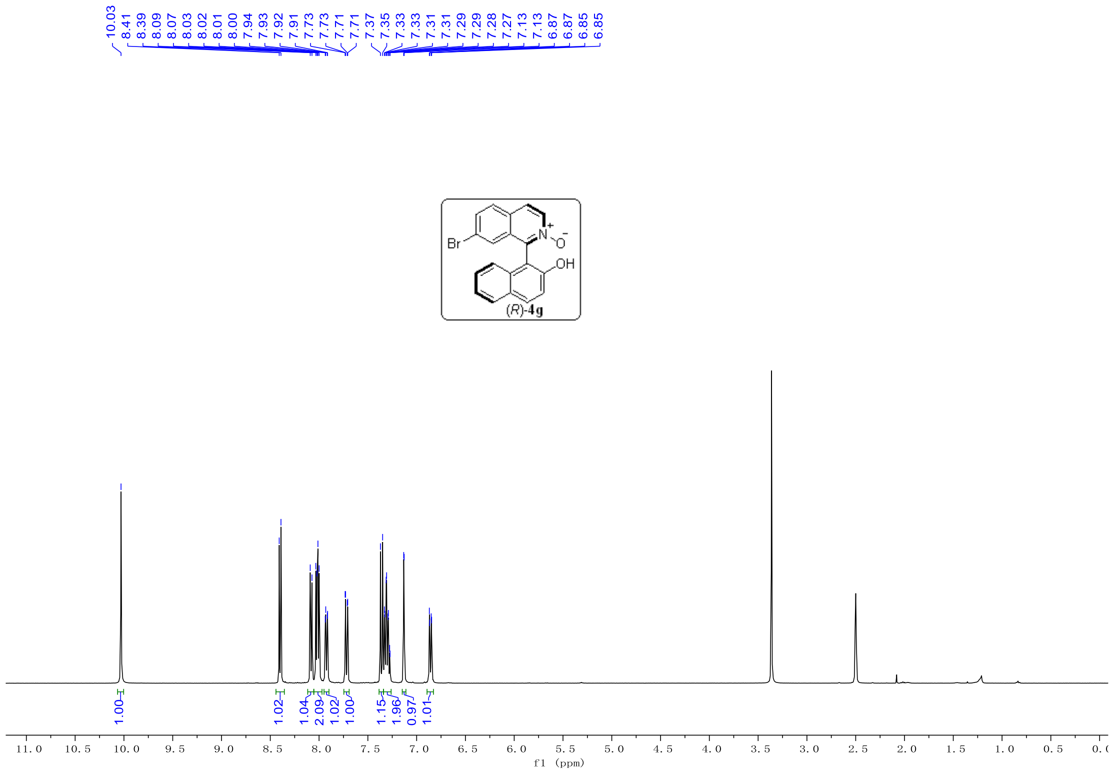

Supplementary Figure 85. ^{1}H NMR spectrum of (*R*)-**4g**

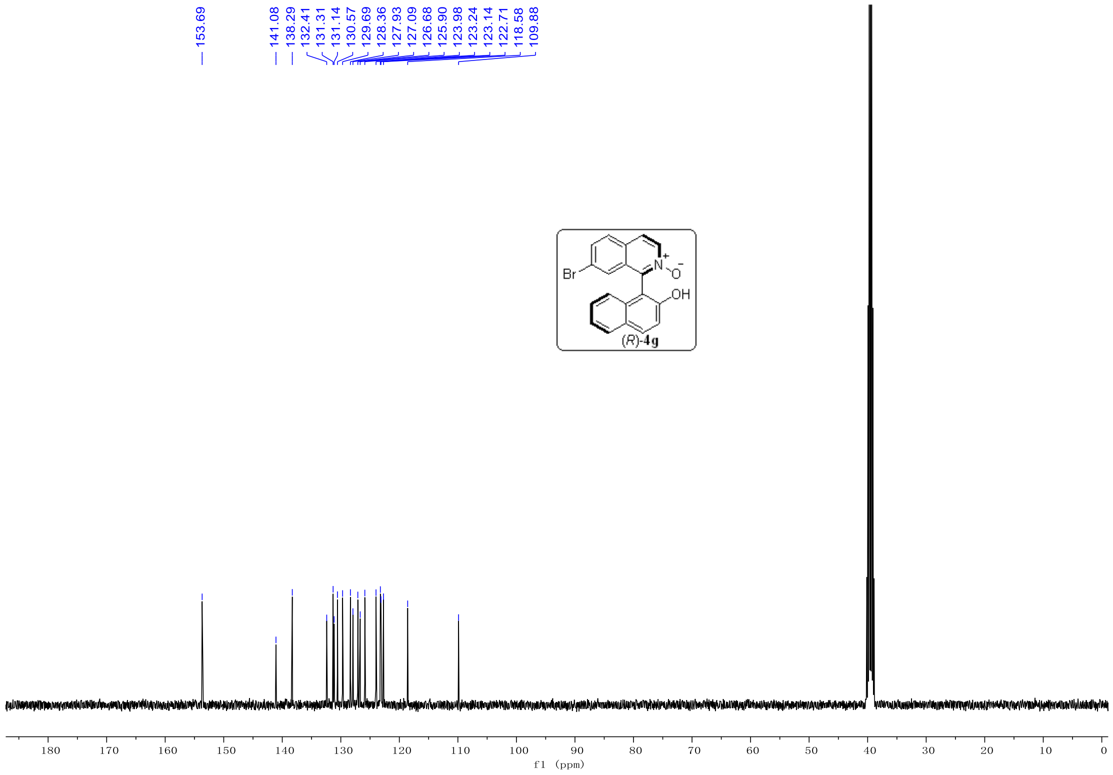

Supplementary Figure 86. ^{13}C NMR spectrum of (*R*)-**4g**

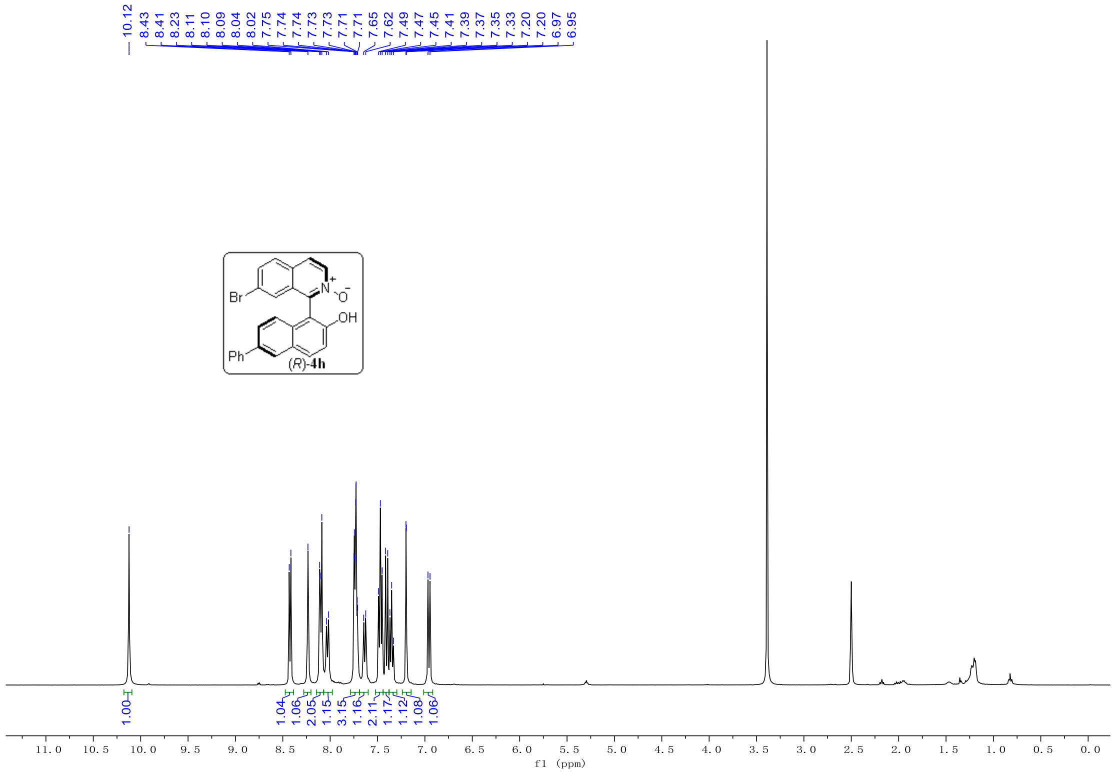

Supplementary Figure 87. ^{1}H NMR spectrum of (*R*)-**4h**

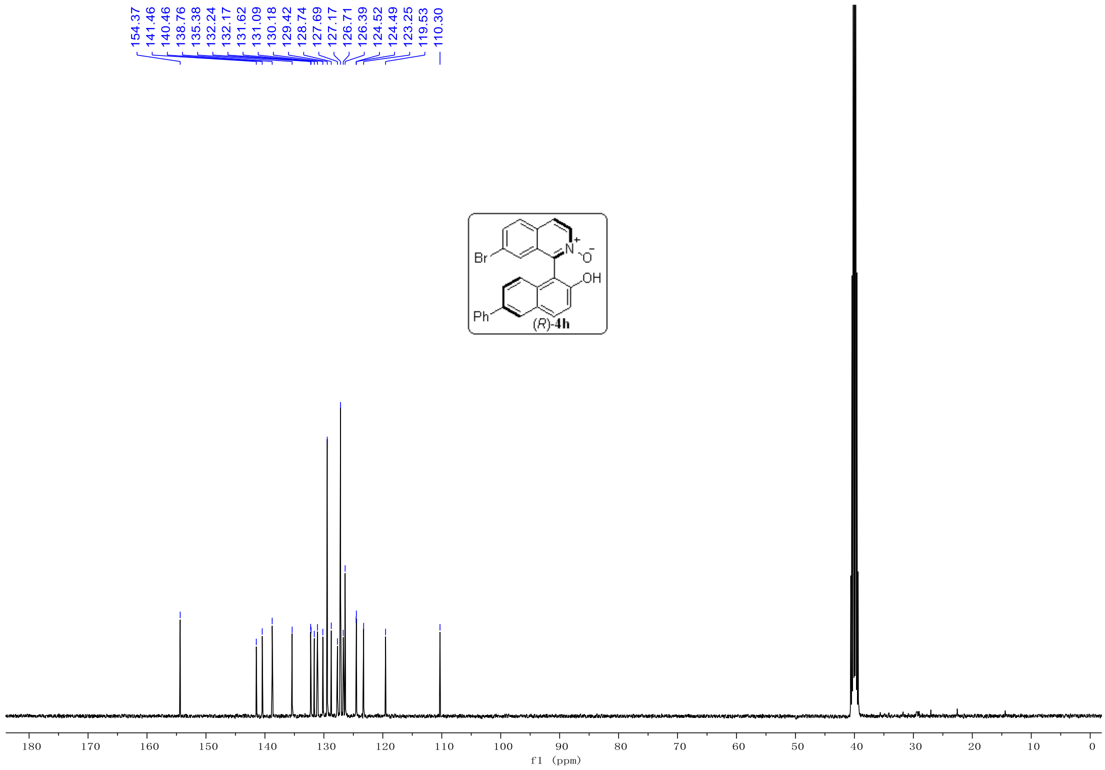

Supplementary Figure 88. ^{13}C NMR spectrum of (*R*)-**4h**

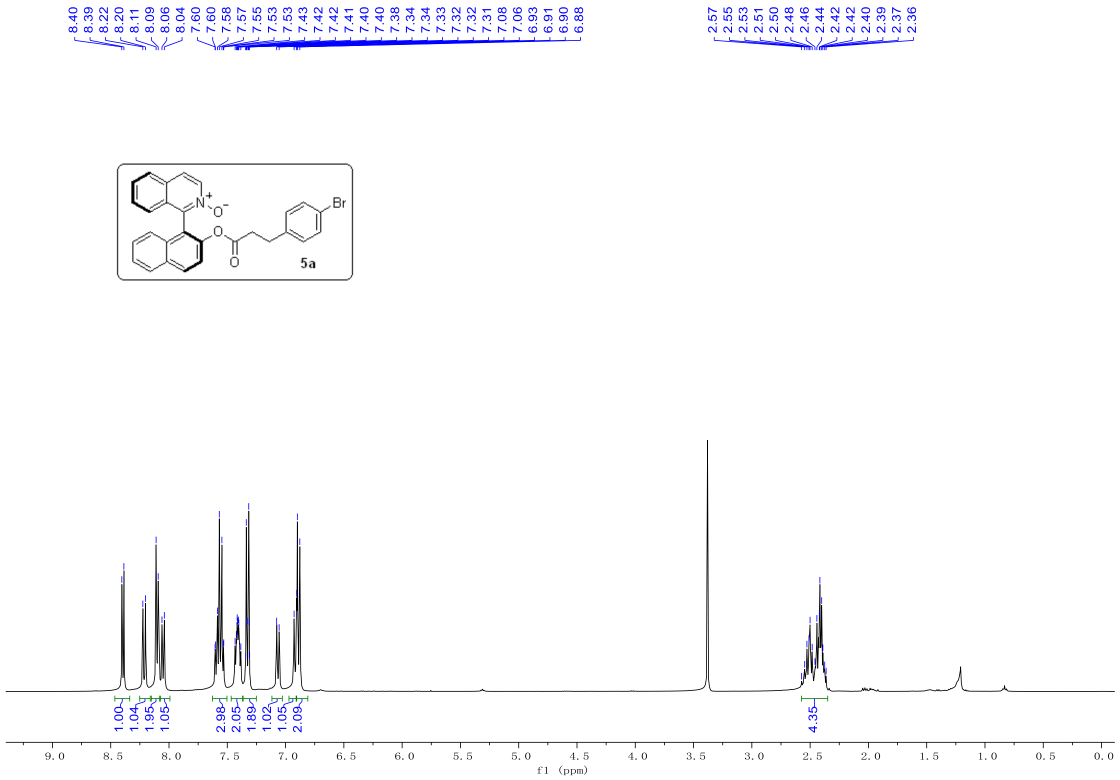

Supplementary Figure 89. ^{1}H NMR spectrum of **5a**

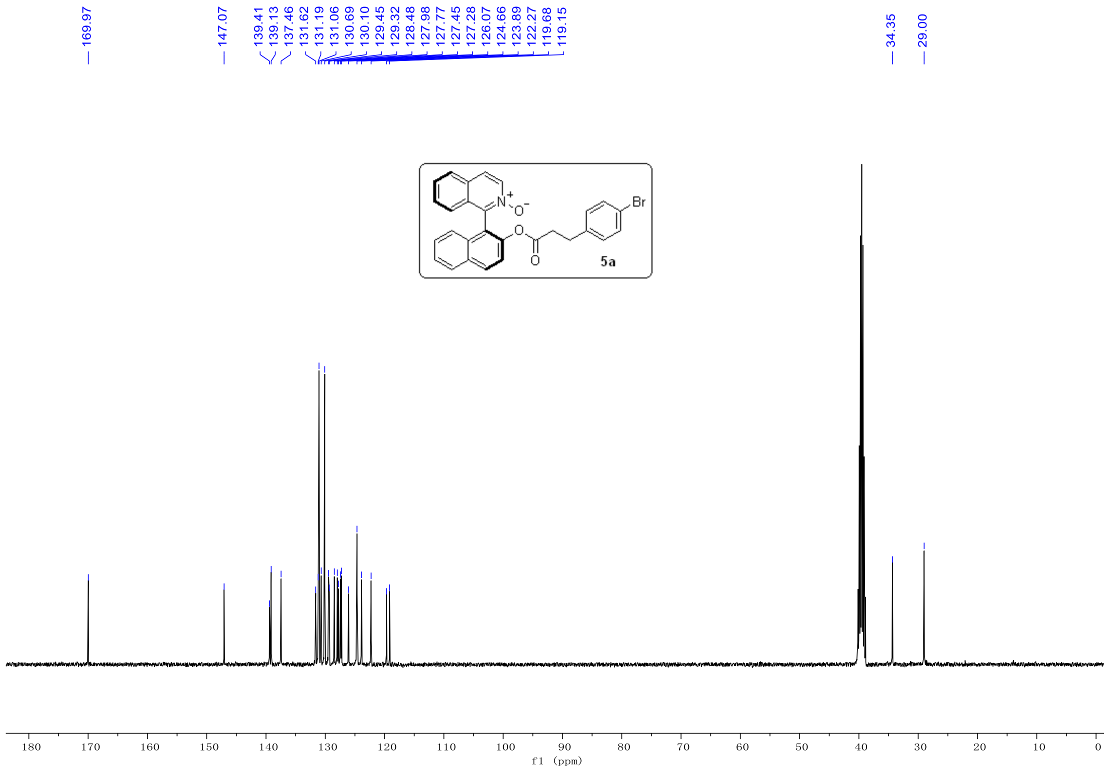

Supplementary Figure 90. ^{13}C NMR spectrum of **5a**

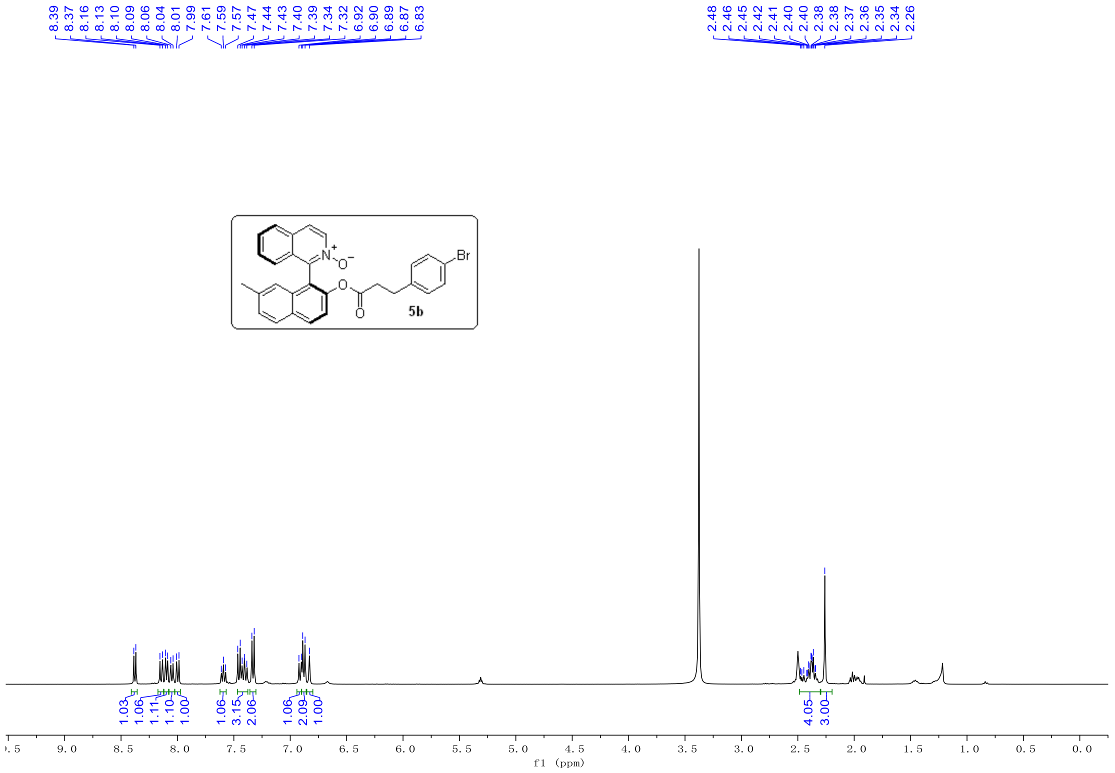

Supplementary Figure 91. ^{1}H NMR spectrum of **5b**

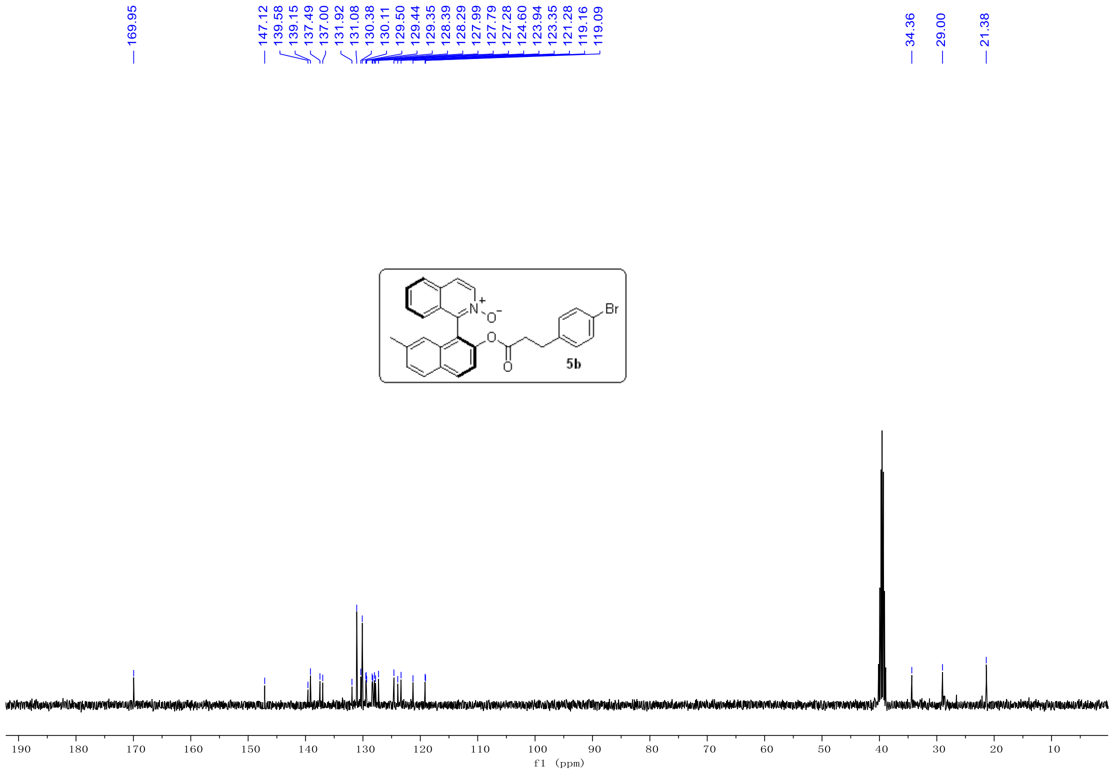

Supplementary Figure 92. ^{13}C NMR spectrum of **5b**

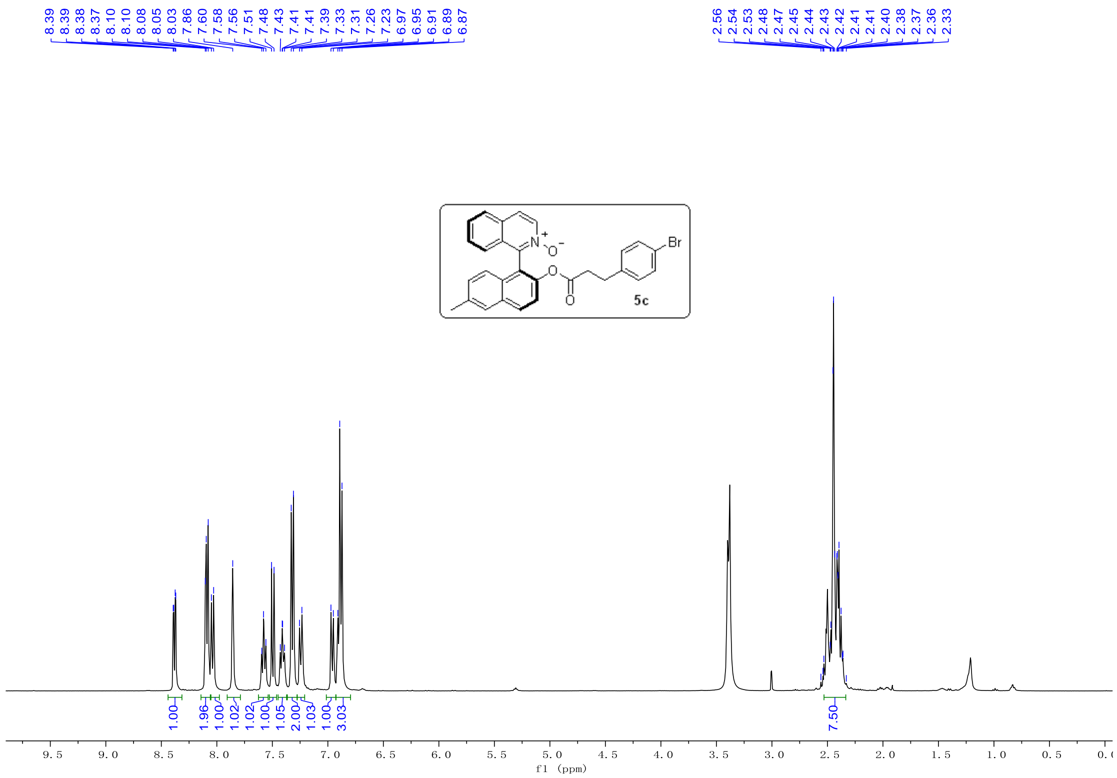

Supplementary Figure 93. ^{1}H NMR spectrum of **5c**

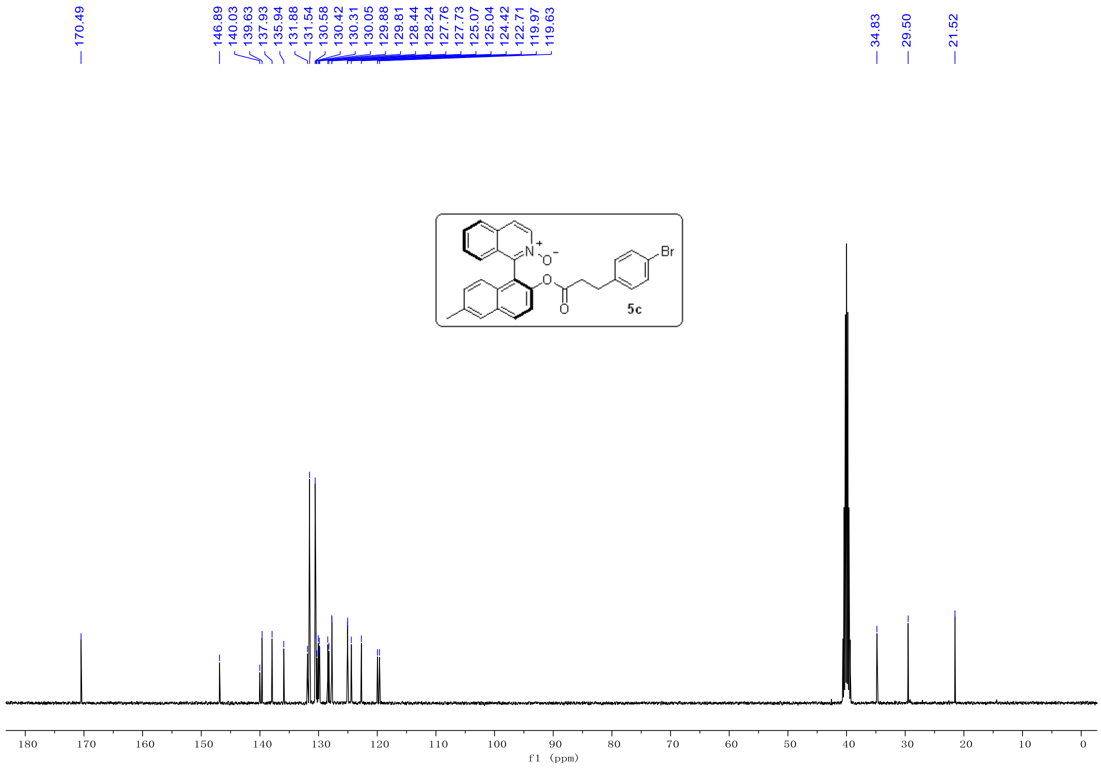

Supplementary Figure 94. ^{13}C NMR spectrum of **5c**

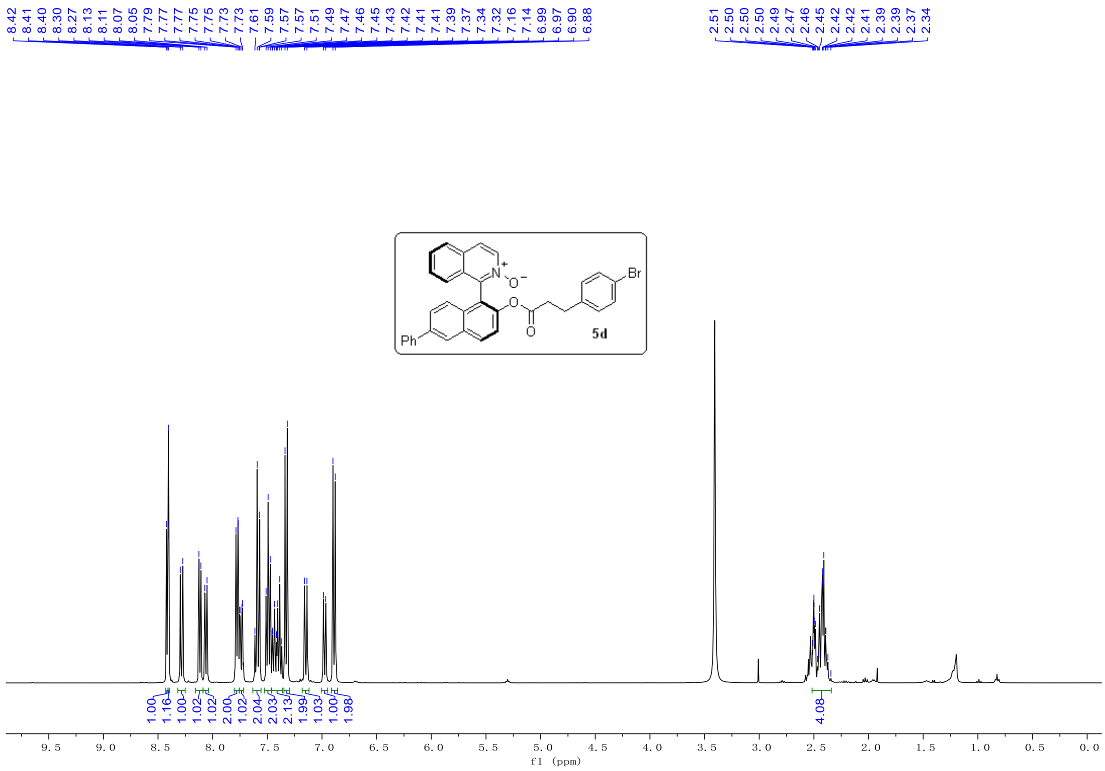

Supplementary Figure 95. ^{1}H NMR spectrum of **5d**

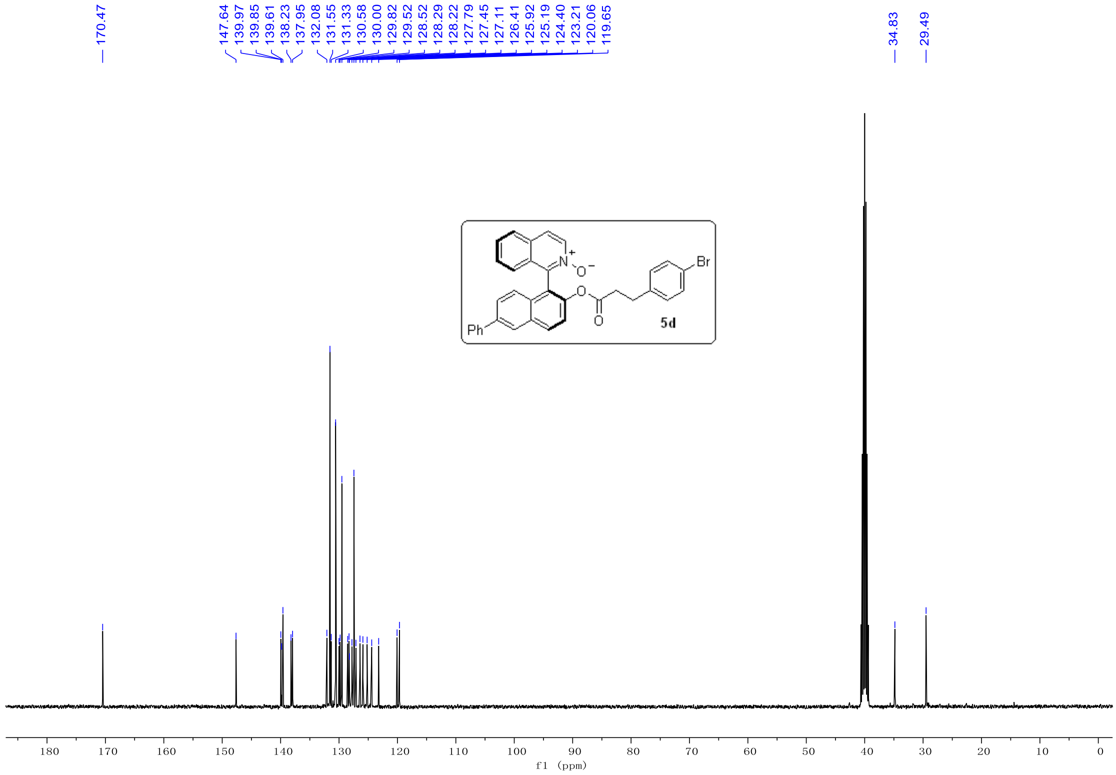

Supplementary Figure 96. ^{13}C NMR spectrum of **5d**

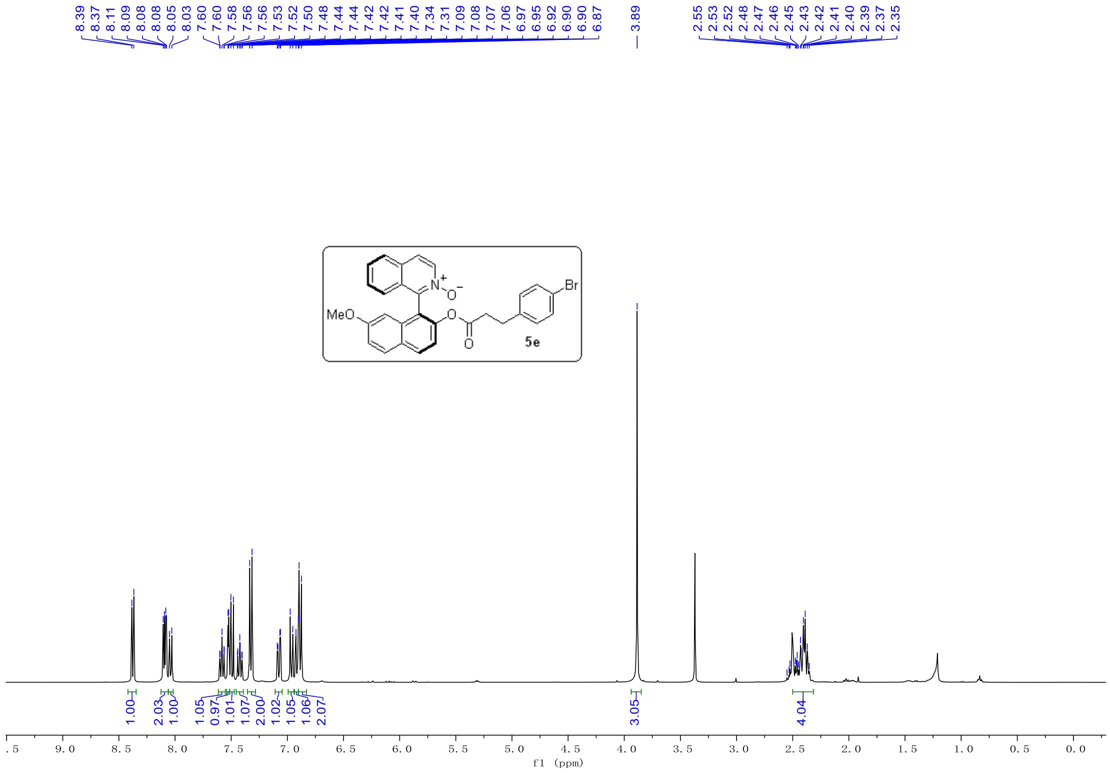

Supplementary Figure 97. ^{1}H NMR spectrum of **5e**

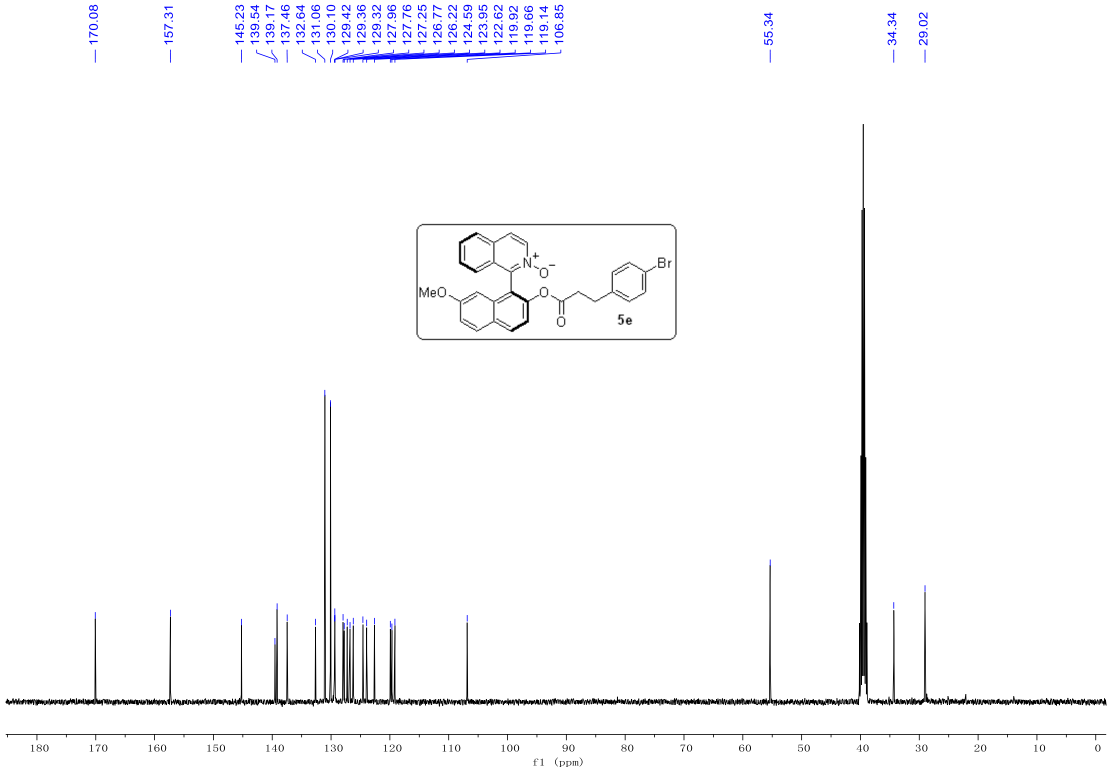

Supplementary Figure 98. ^{13}C NMR spectrum of **5e**

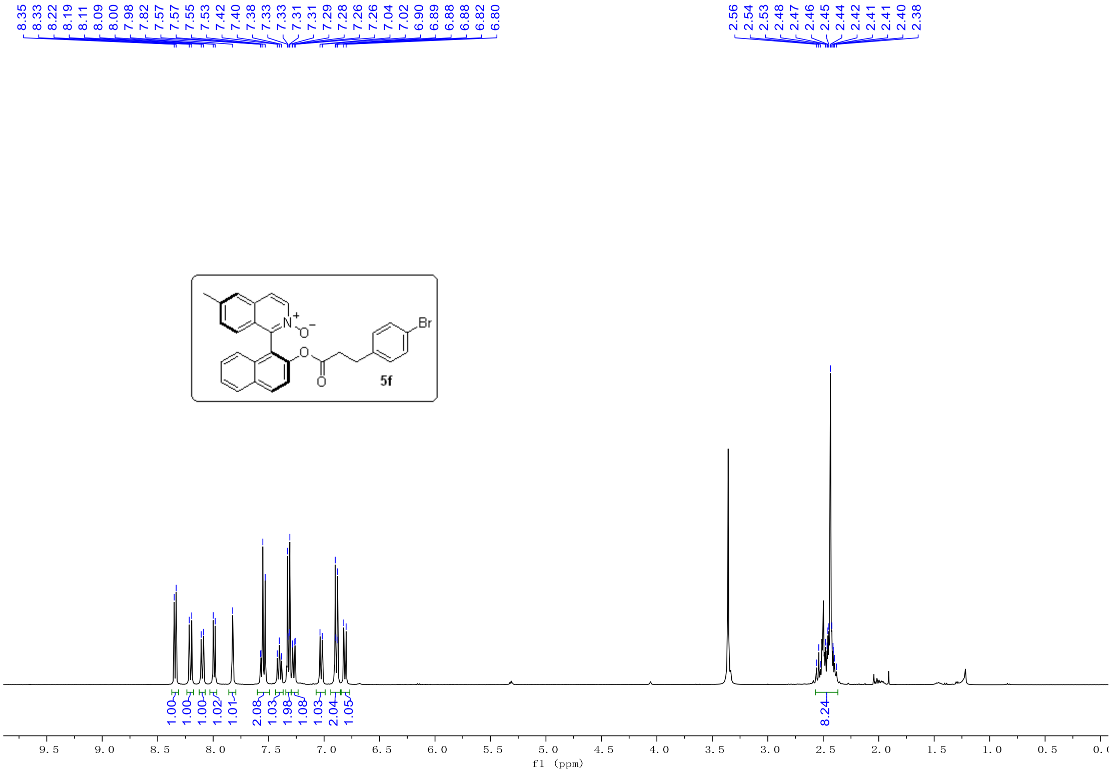

Supplementary Figure 99. ^{1}H NMR spectrum of **5f**

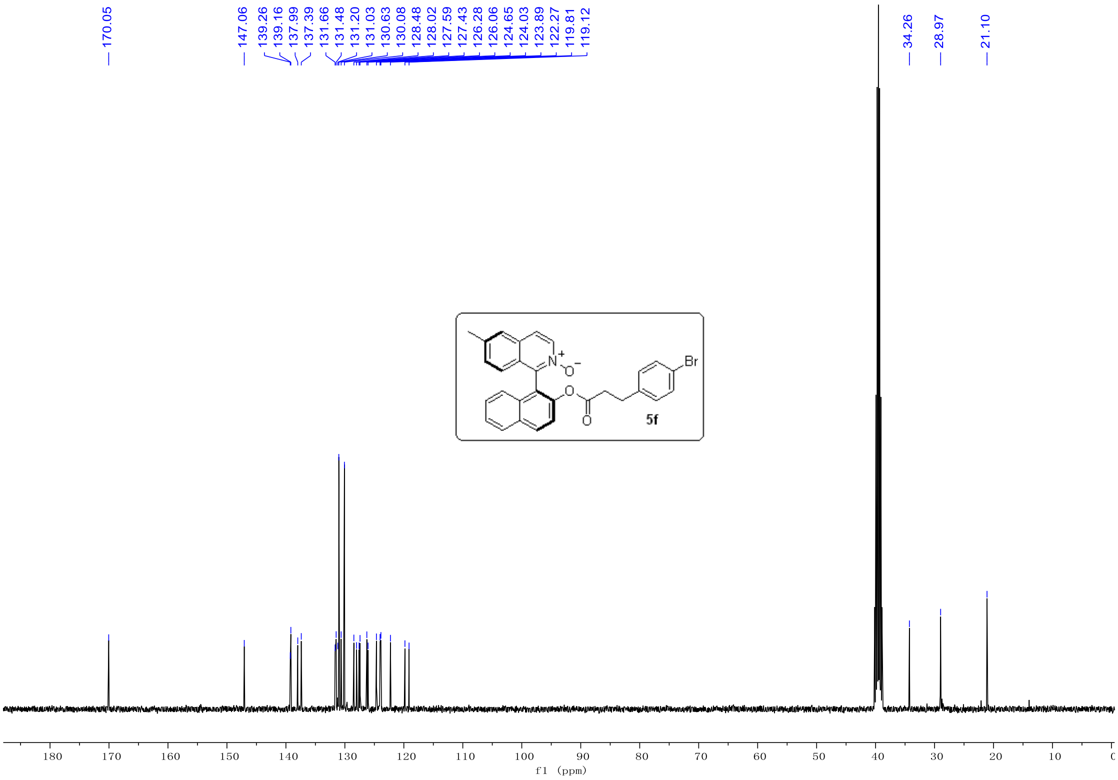

Supplementary Figure 100. ^{13}C NMR spectrum of **5f**

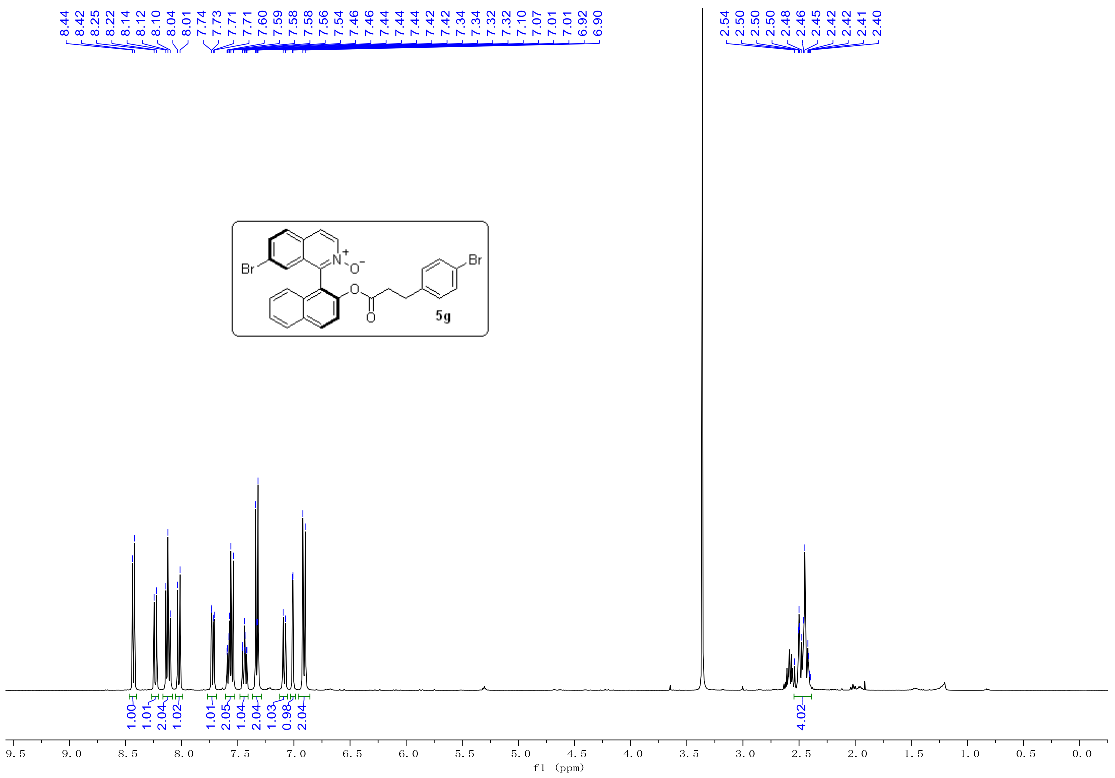

Supplementary Figure 101. ^{1}H NMR spectrum of **5g**

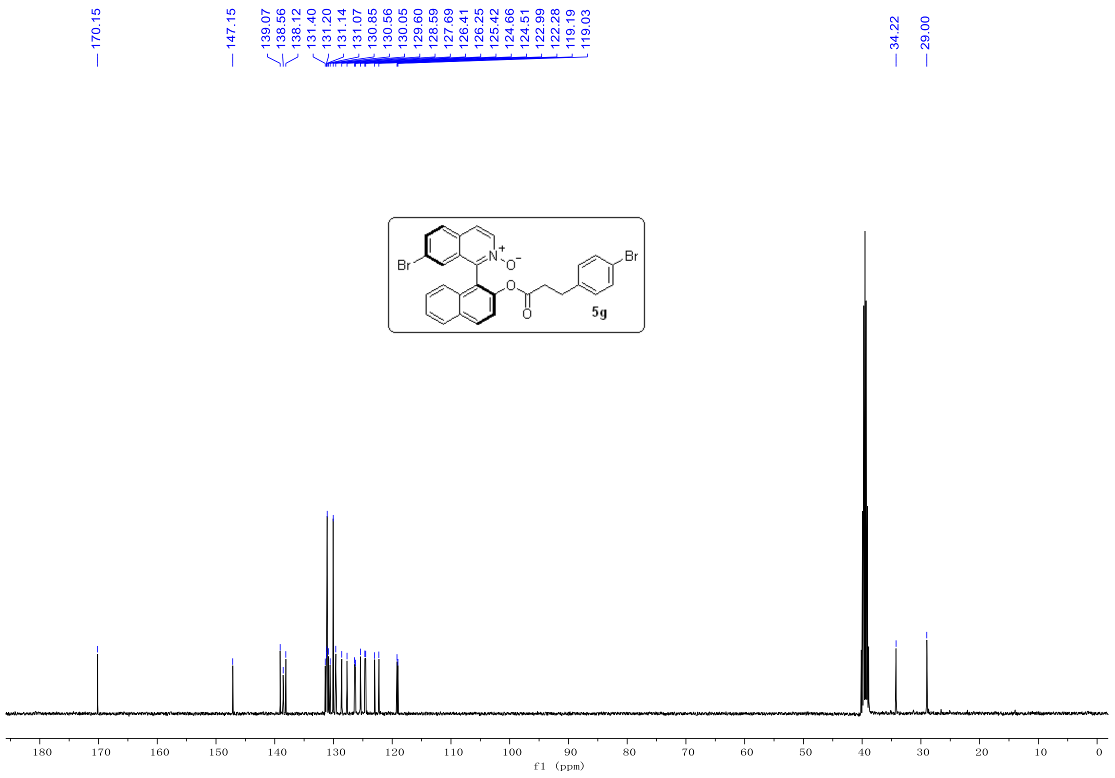

Supplementary Figure 102. ^{13}C NMR spectrum of **5g**

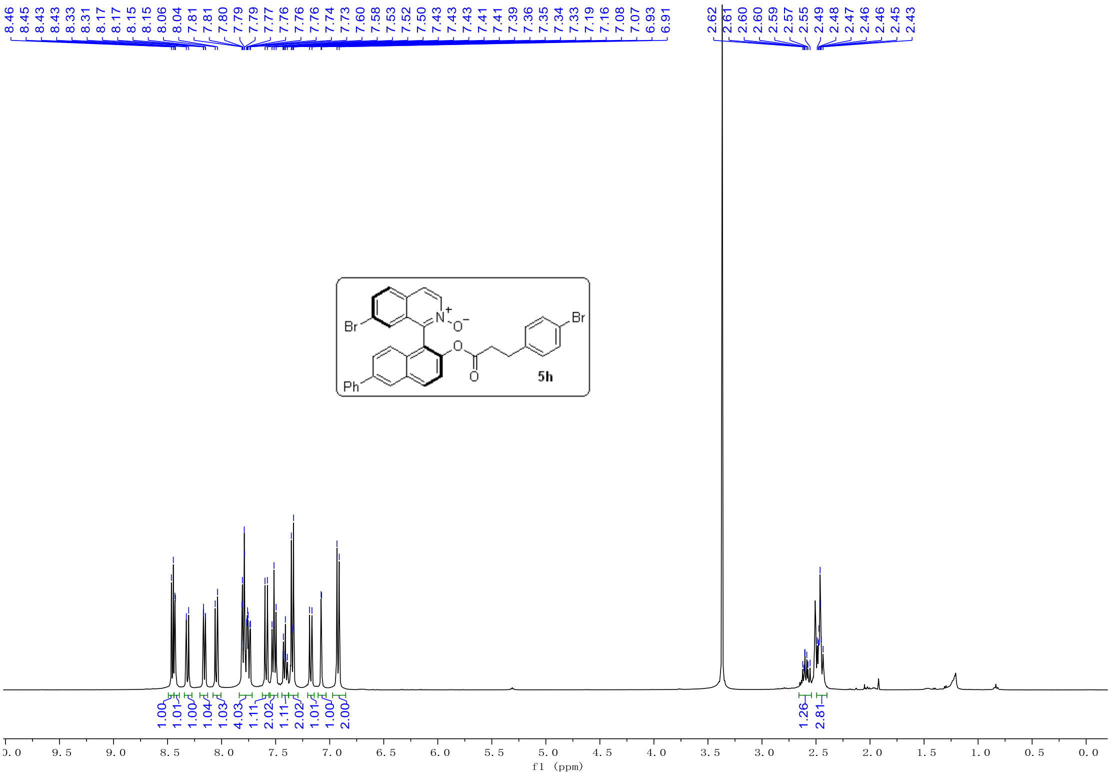

Supplementary Figure 103. ^{1}H NMR spectrum of **5h**

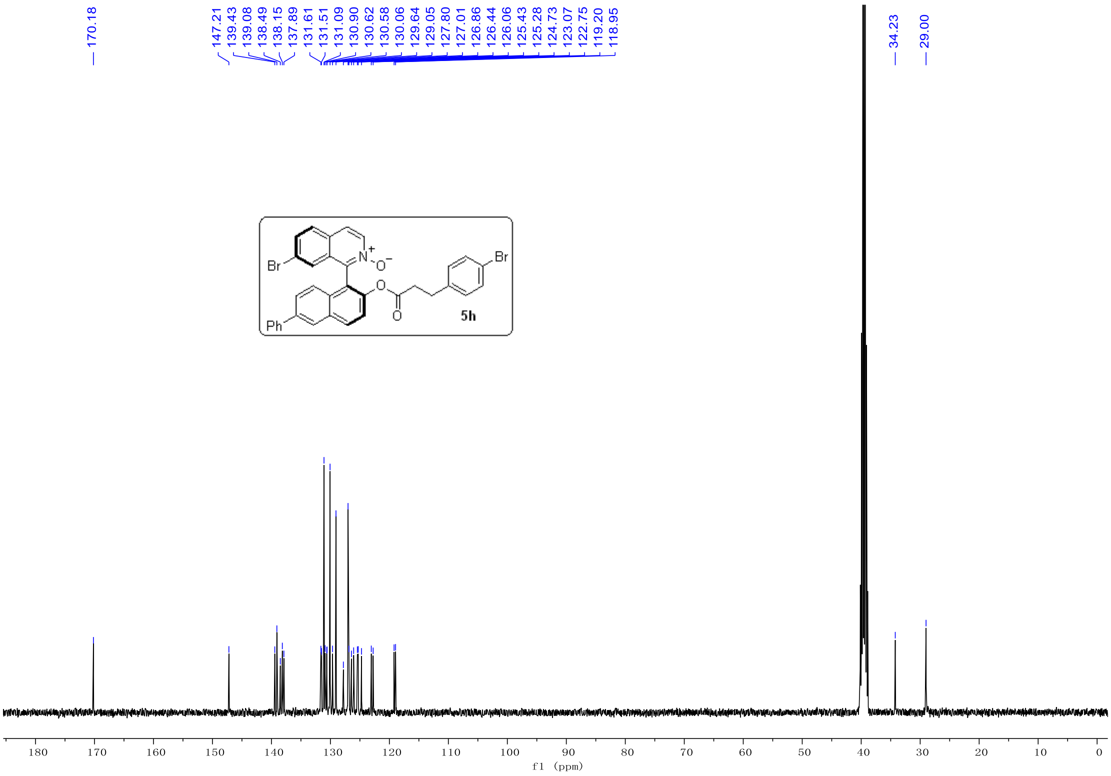

Supplementary Figure 104. ^{13}C NMR spectrum of **5h**

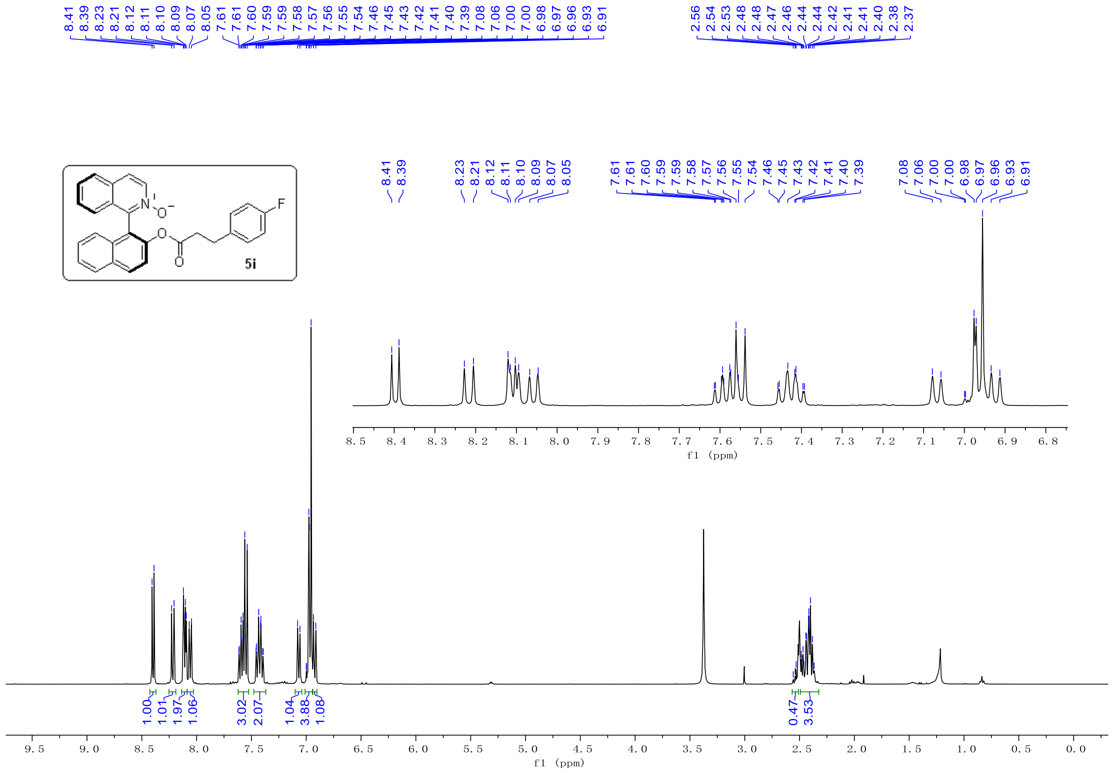

Supplementary Figure 105. ^{1}H NMR spectrum of **5i**

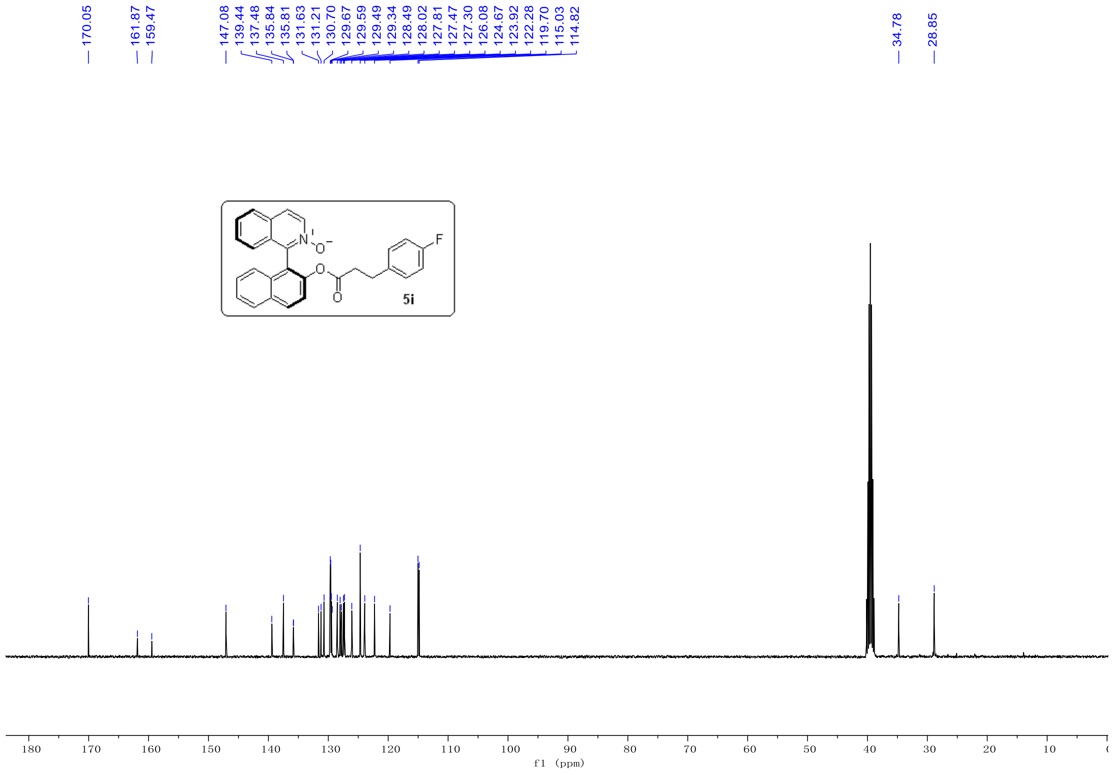

Supplementary Figure 106. ^{13}C NMR spectrum of **5i**

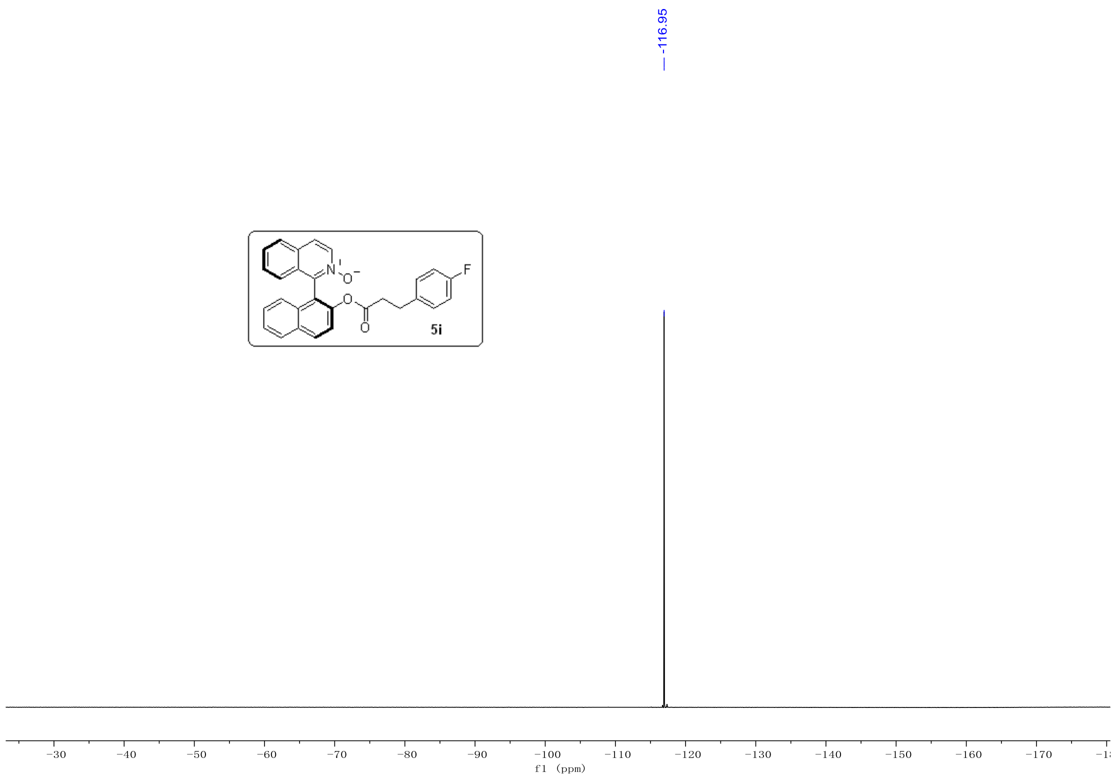

Supplementary Figure 107. ^{19}F NMR spectrum of **5i**

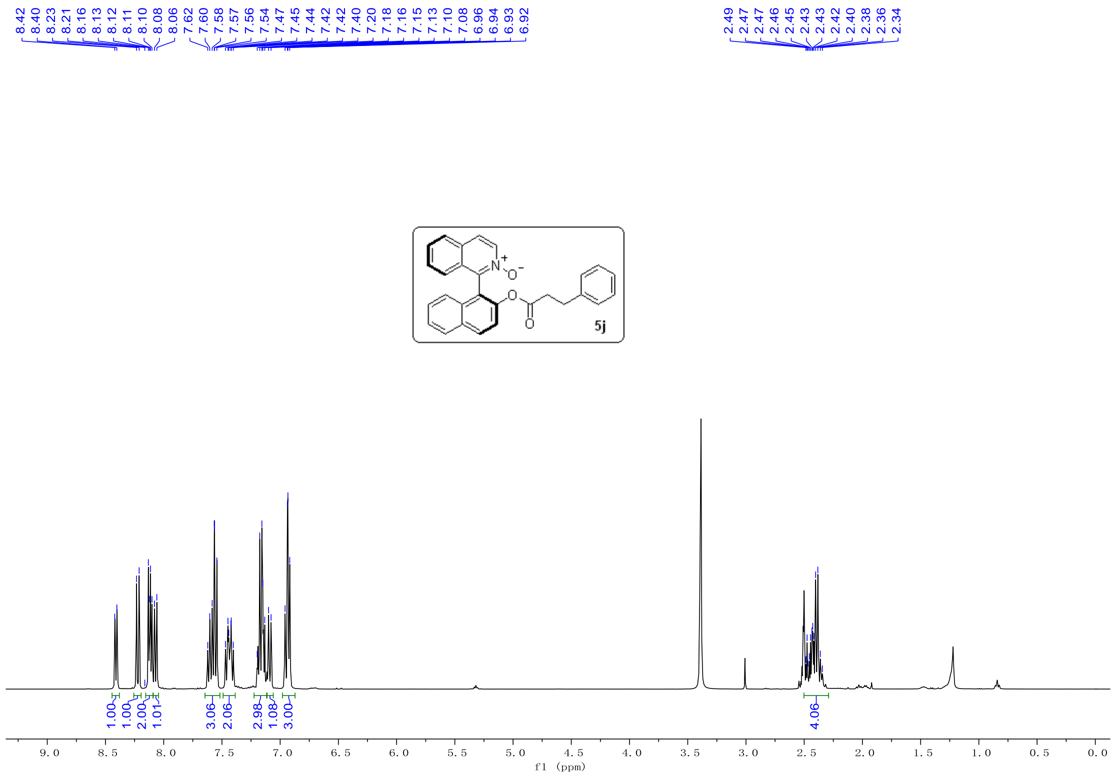

Supplementary Figure 108. ^{1}H NMR spectrum of **5j**

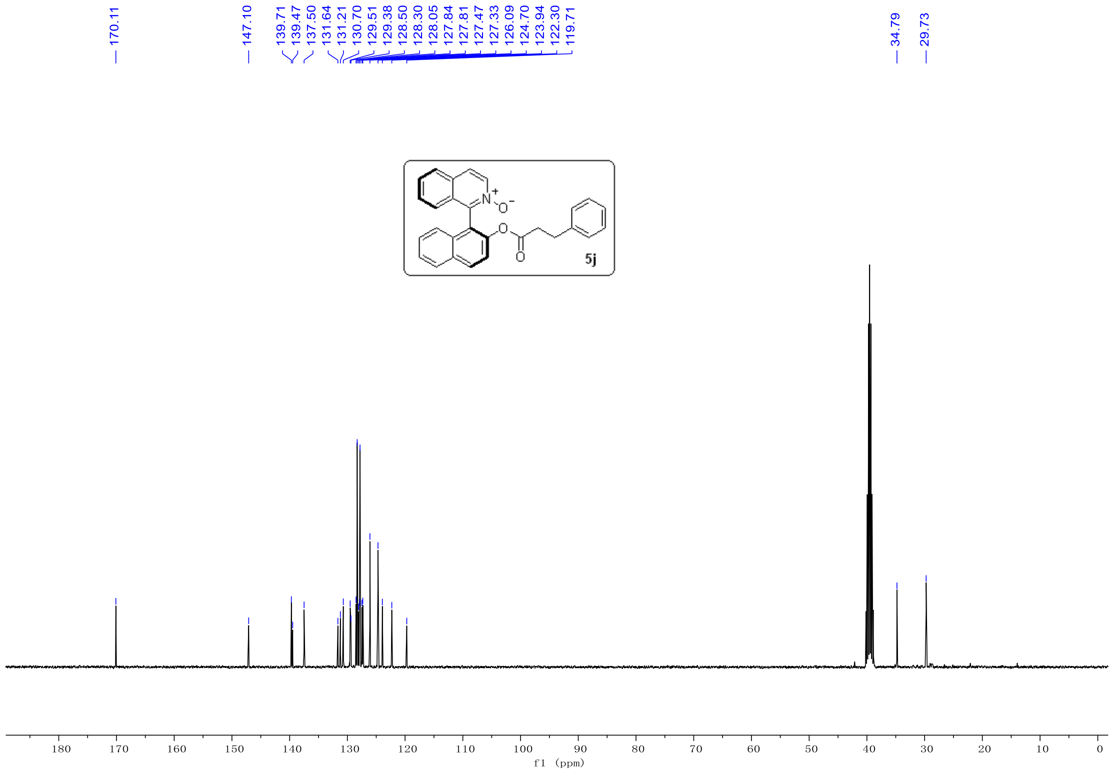

Supplementary Figure 109. ^{13}C NMR spectrum of **5j**

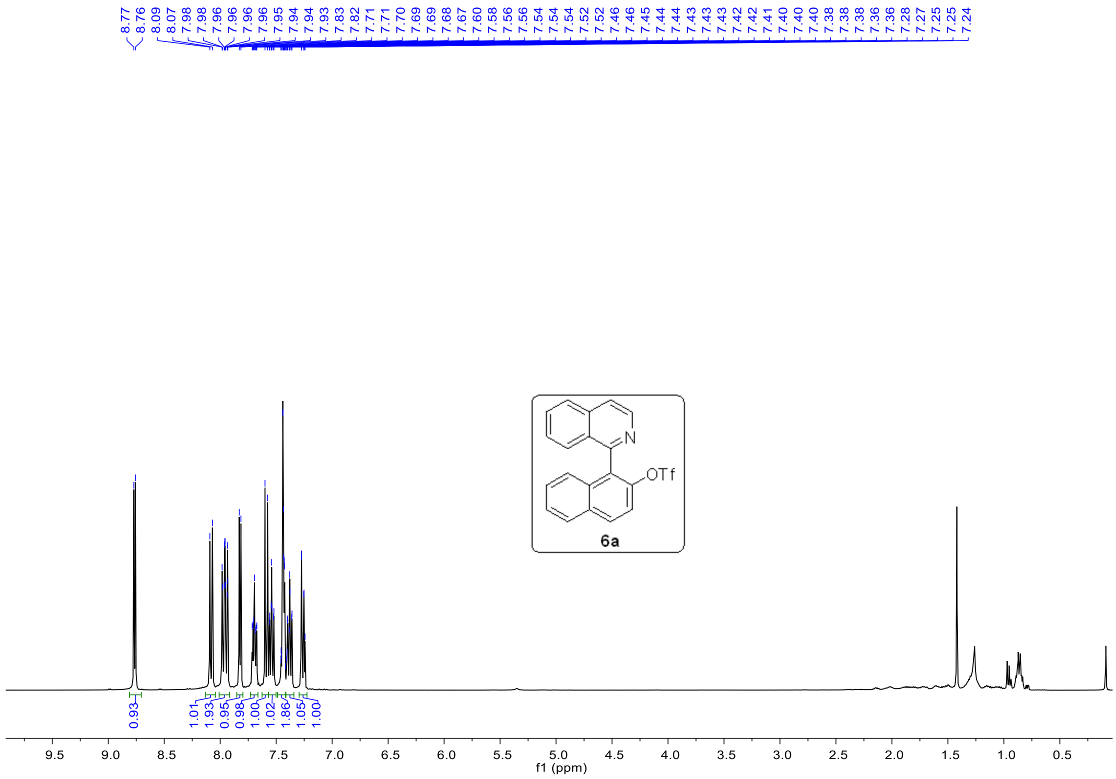

Supplementary Figure 110. ^{1}H NMR spectrum of **6a**

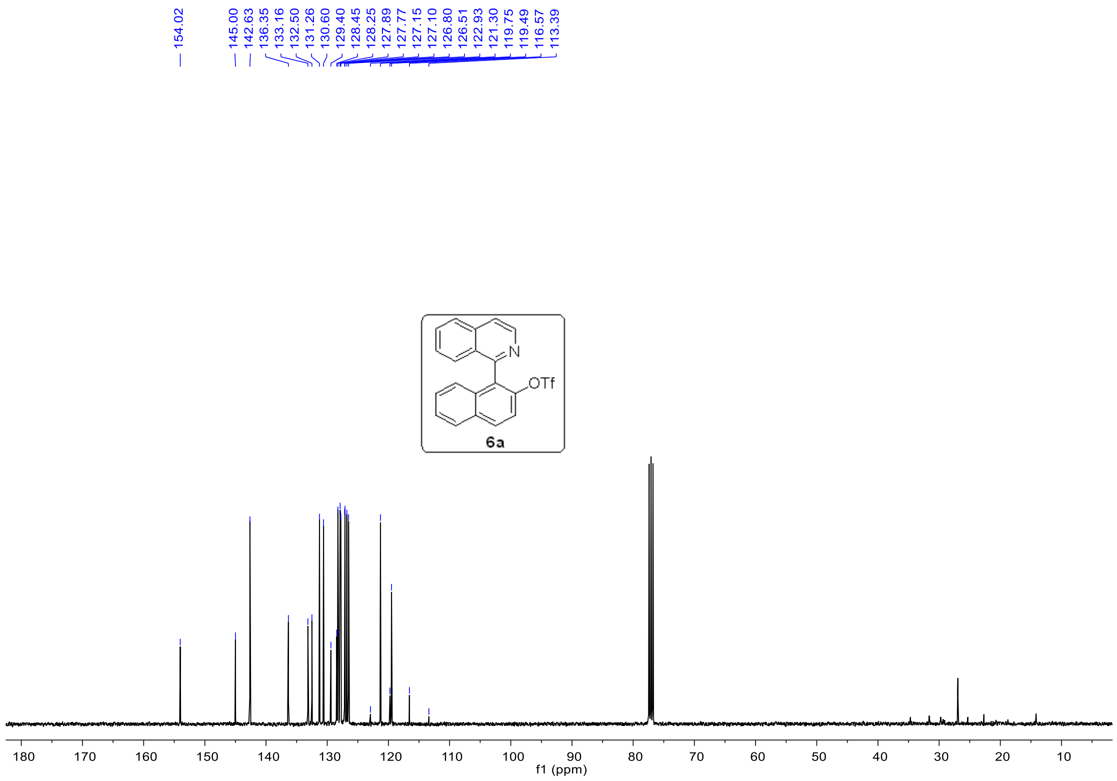

Supplementary Figure 111. ^{13}C NMR spectrum of **6a**

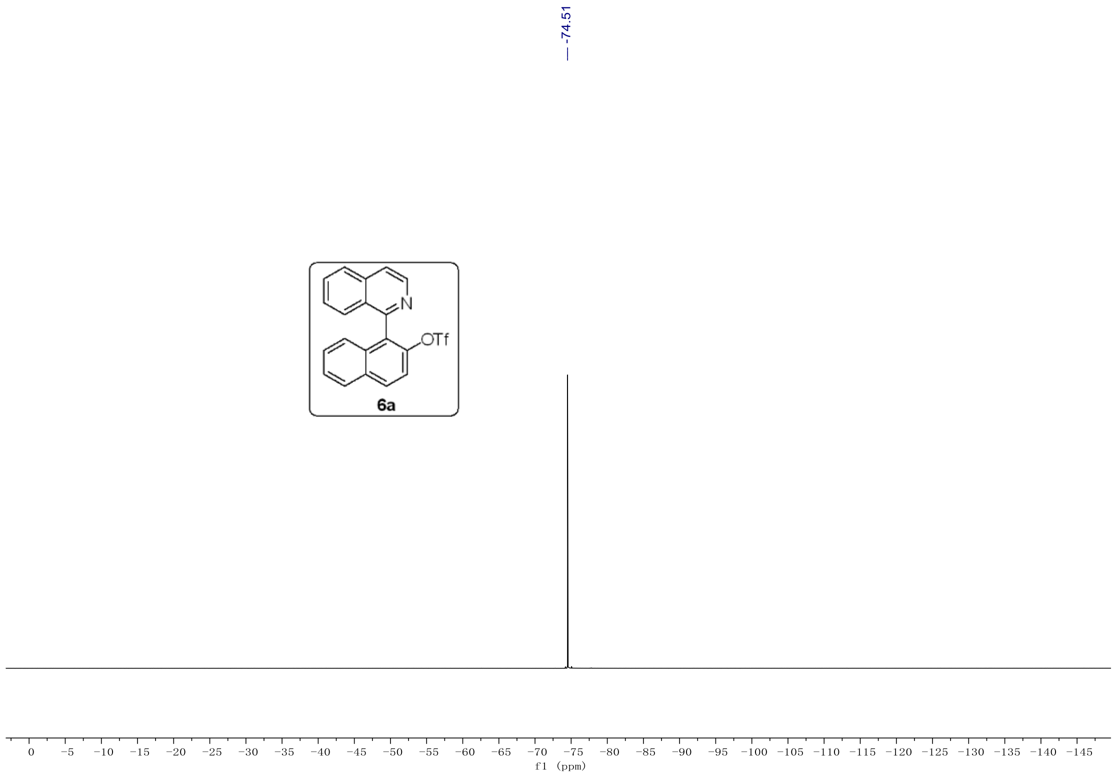

Supplementary Figure 112. ^{19}F NMR spectrum of **6a**

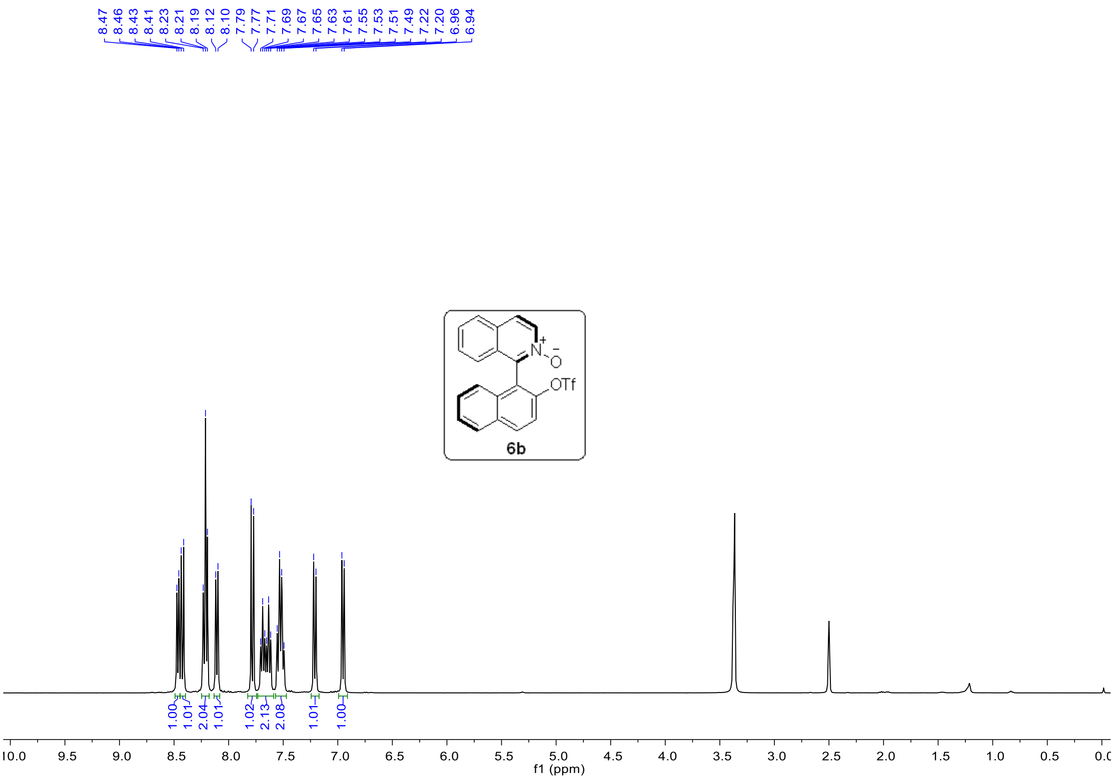

Supplementary Figure 113. ^{1}H NMR spectrum of **6b**

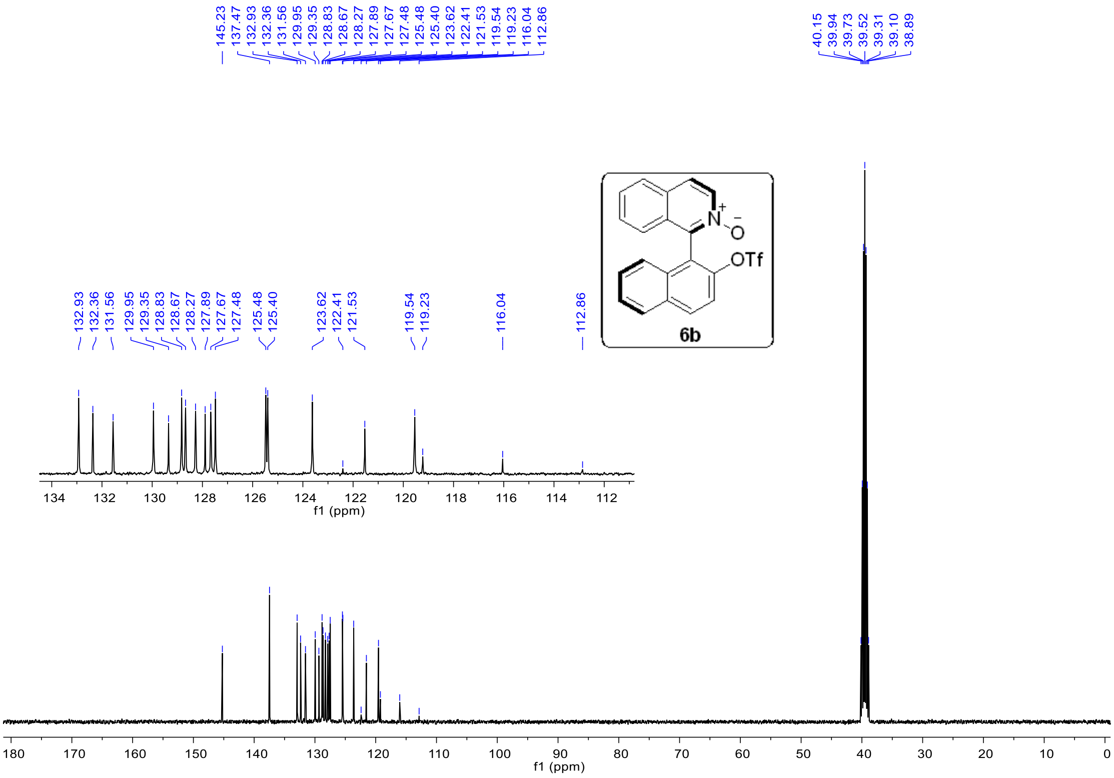

Supplementary Figure 114. ^{13}C NMR spectrum of **6b**

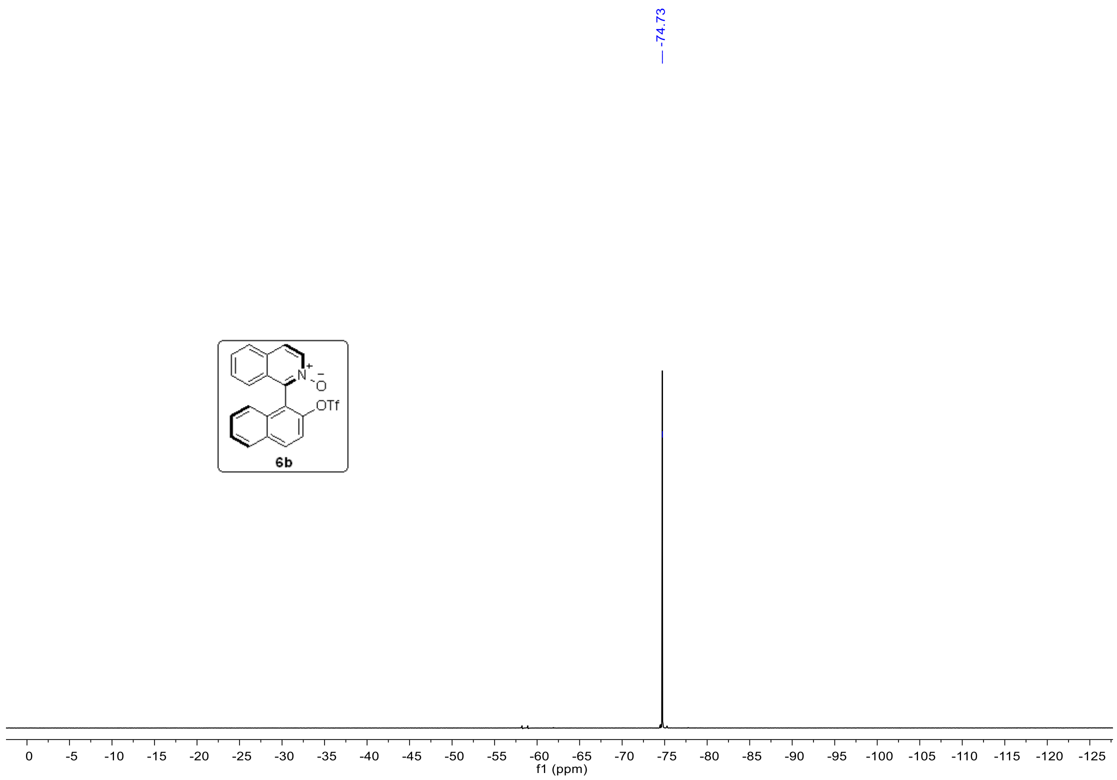

Supplementary Figure 115. ^{19}F NMR spectrum of **6b**

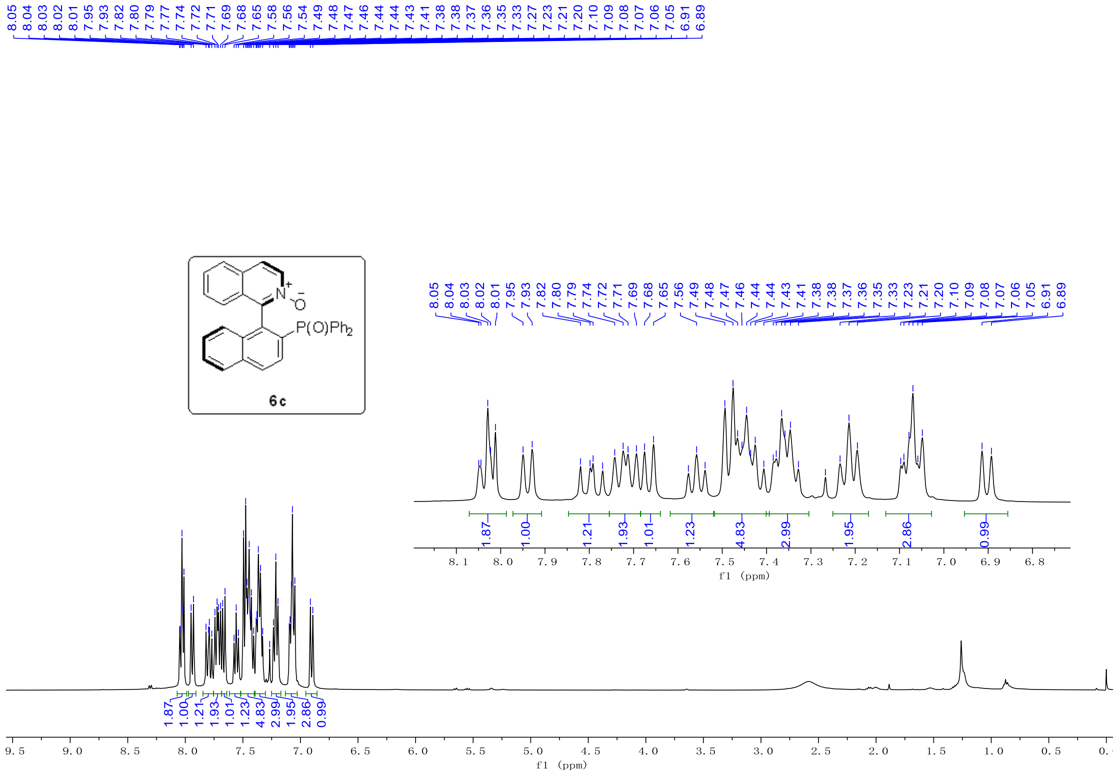

Supplementary Figure 116. ^{1}H NMR spectrum of **6c**

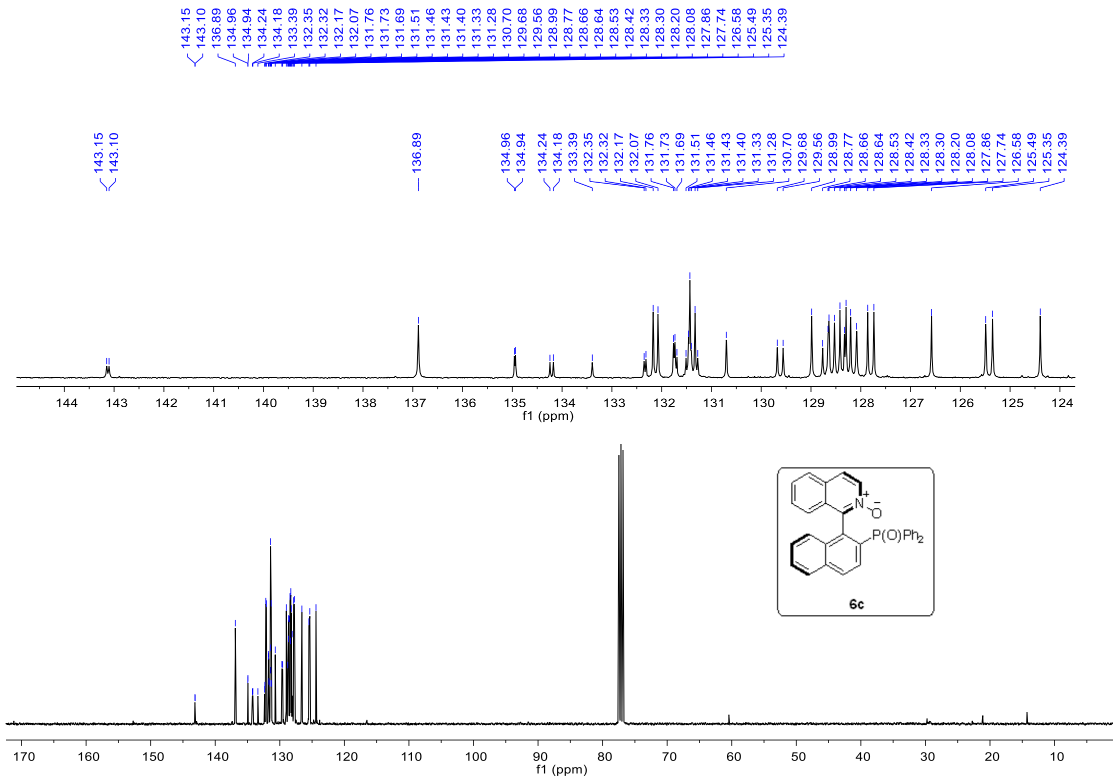

Supplementary Figure 117. ^{13}C NMR spectrum of **6c**

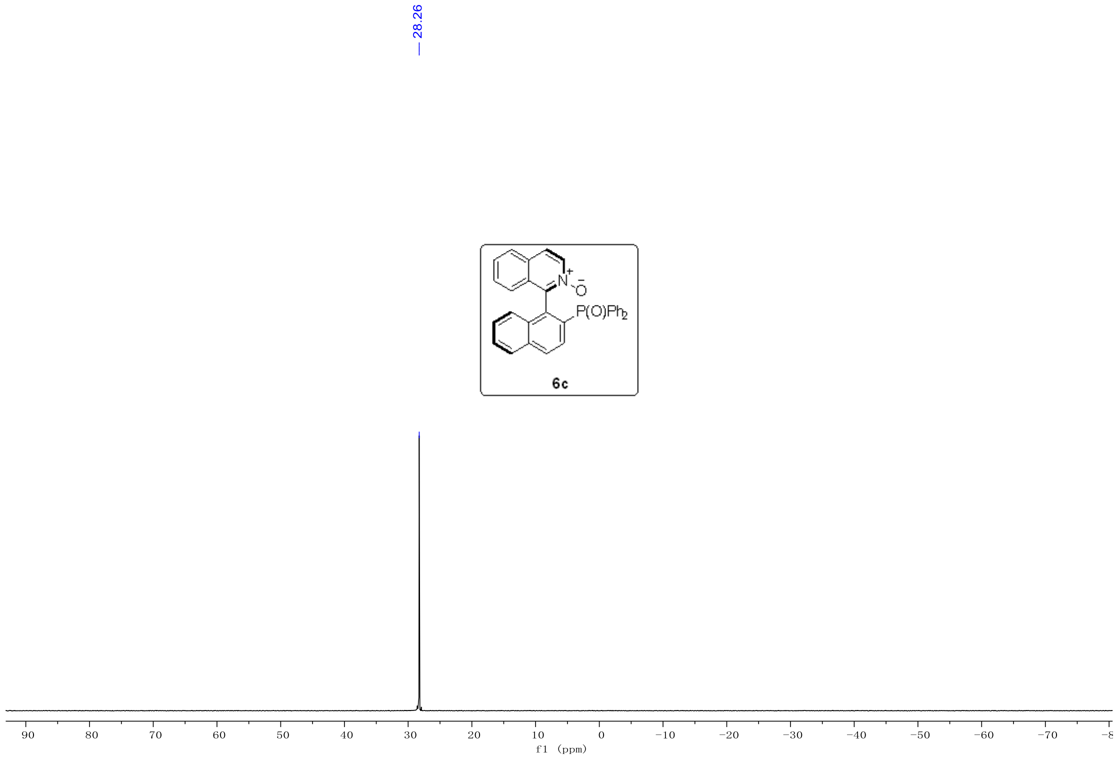

Supplementary Figure 118. ^{31}P NMR spectrum of **6c**

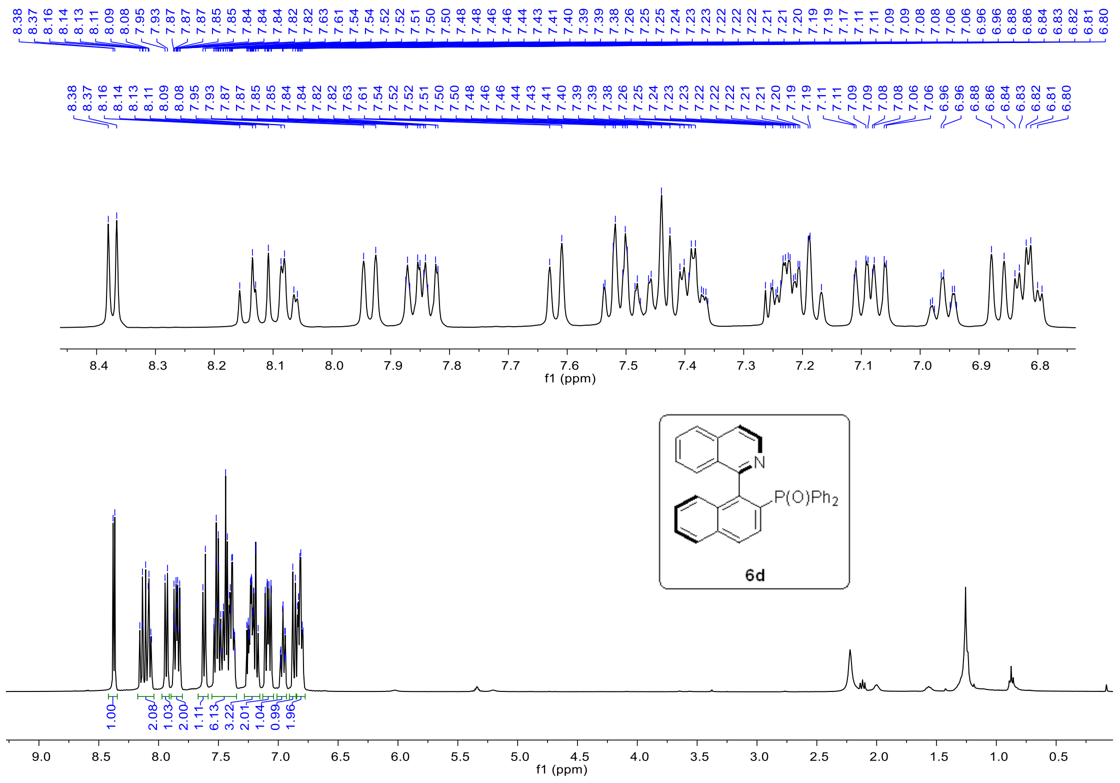

Supplementary Figure 119. ^{1}H NMR spectrum of **6d**

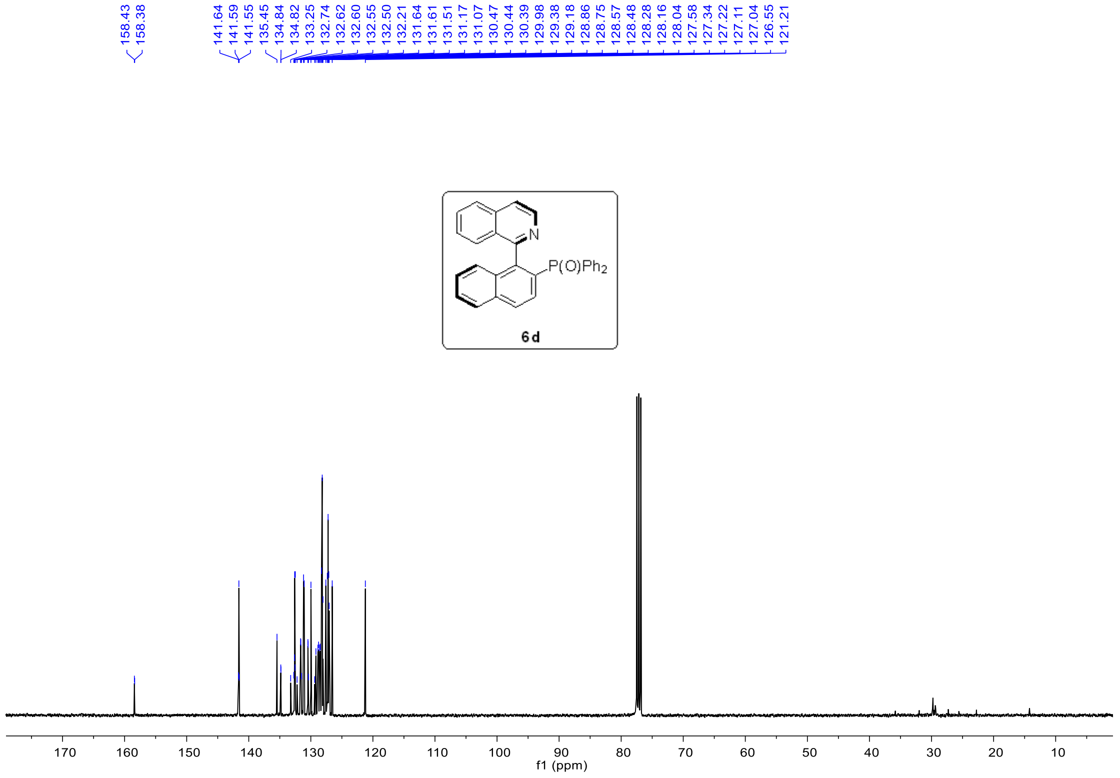

Supplementary Figure 120. ^{13}C NMR spectrum of **6d**

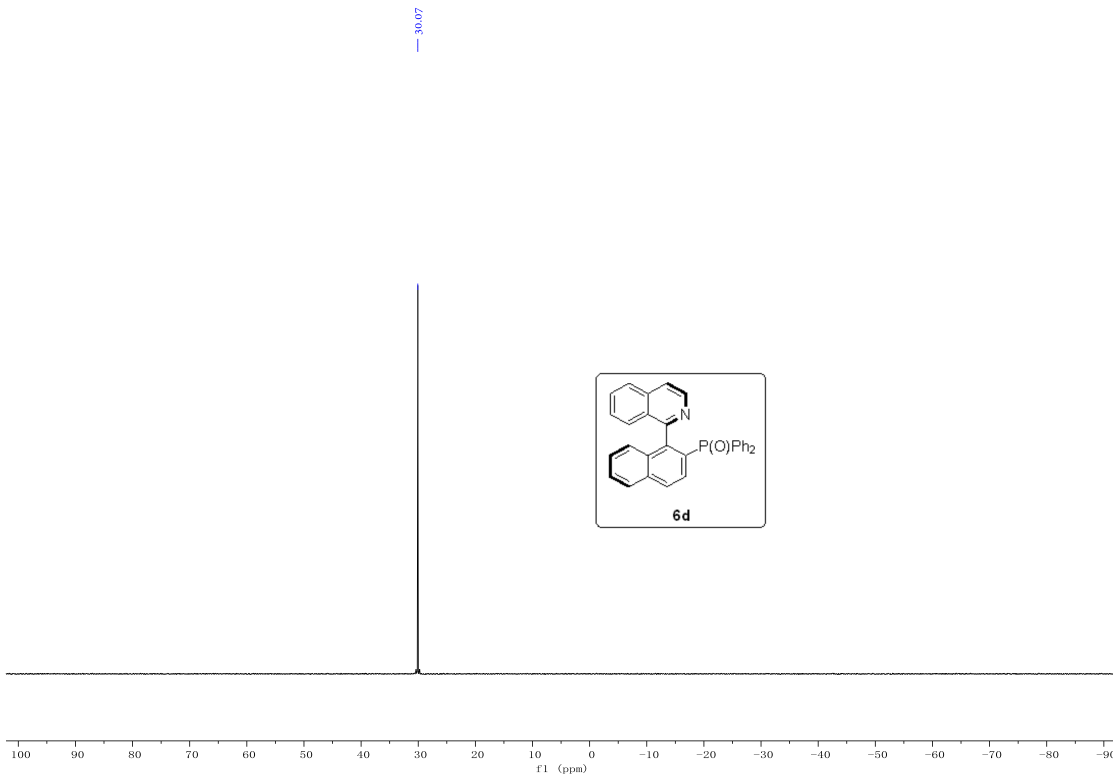

Supplementary Figure 121. ^{31}P NMR spectrum of **6d**

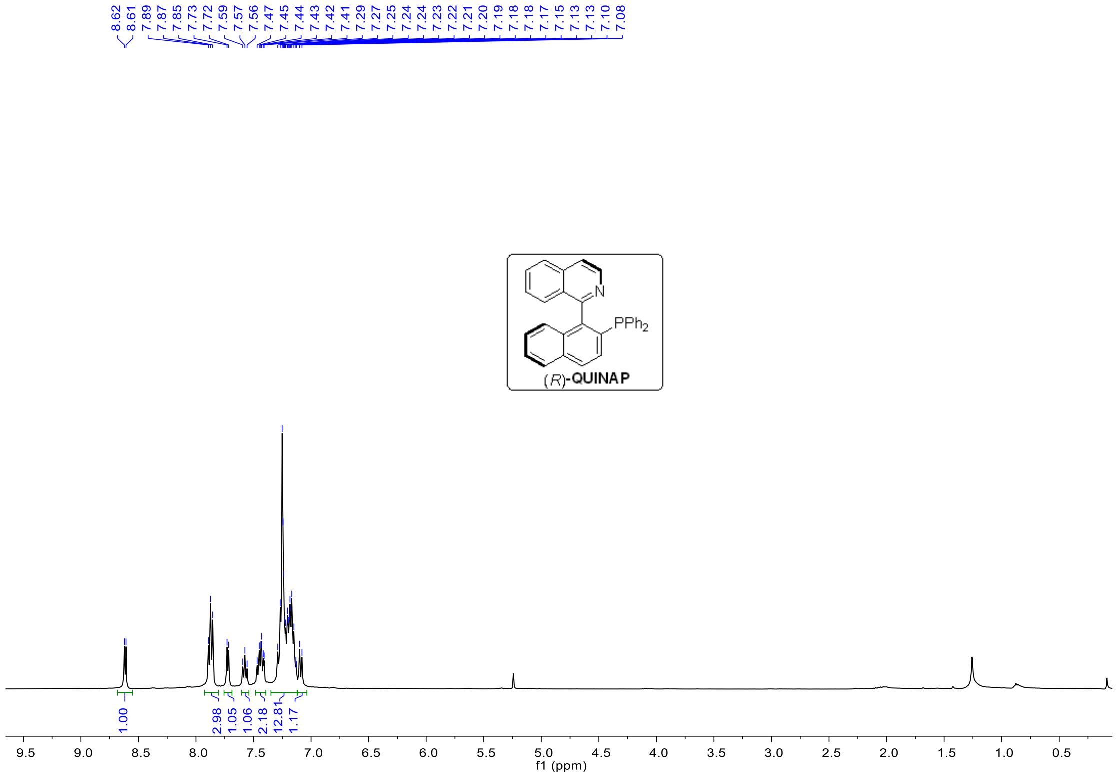

Supplementary Figure 122. ^{1}H NMR spectrum of (*R*)-**QUINAP**

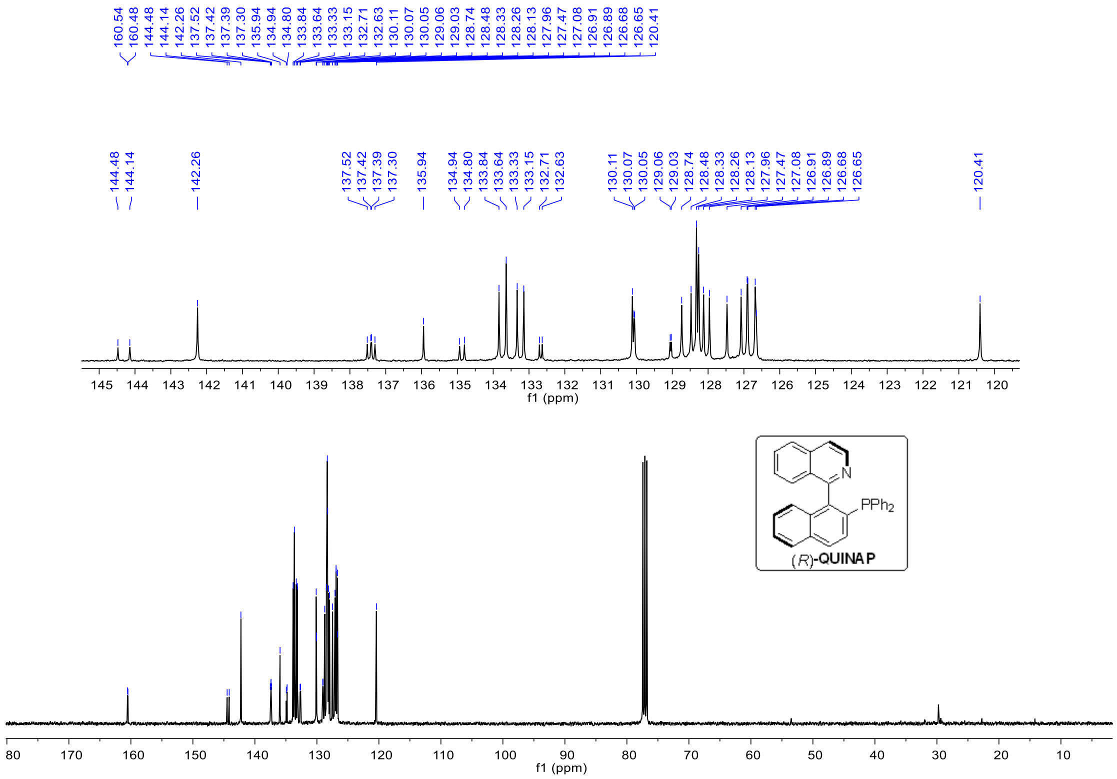

Supplementary Figure 123. ^{13}C NMR spectrum of (*R*)-**QUINAP**

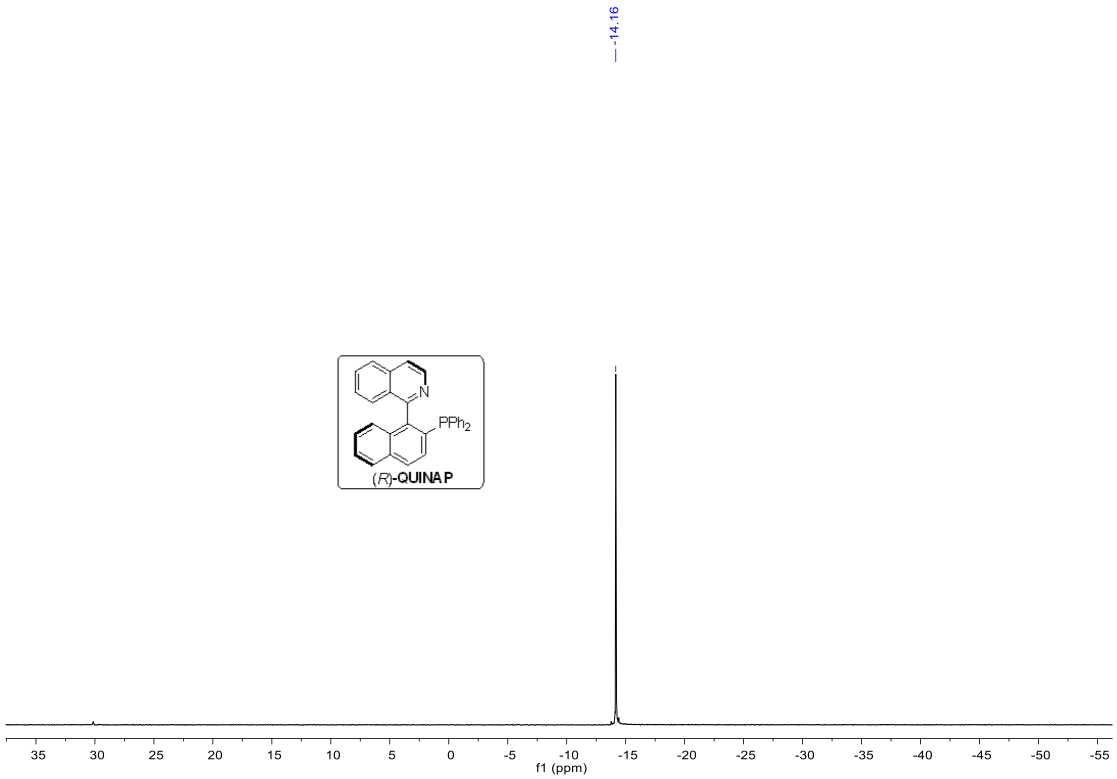

Supplementary Figure 124. ^{31}P NMR spectrum of (*R*)-**QUINAP**

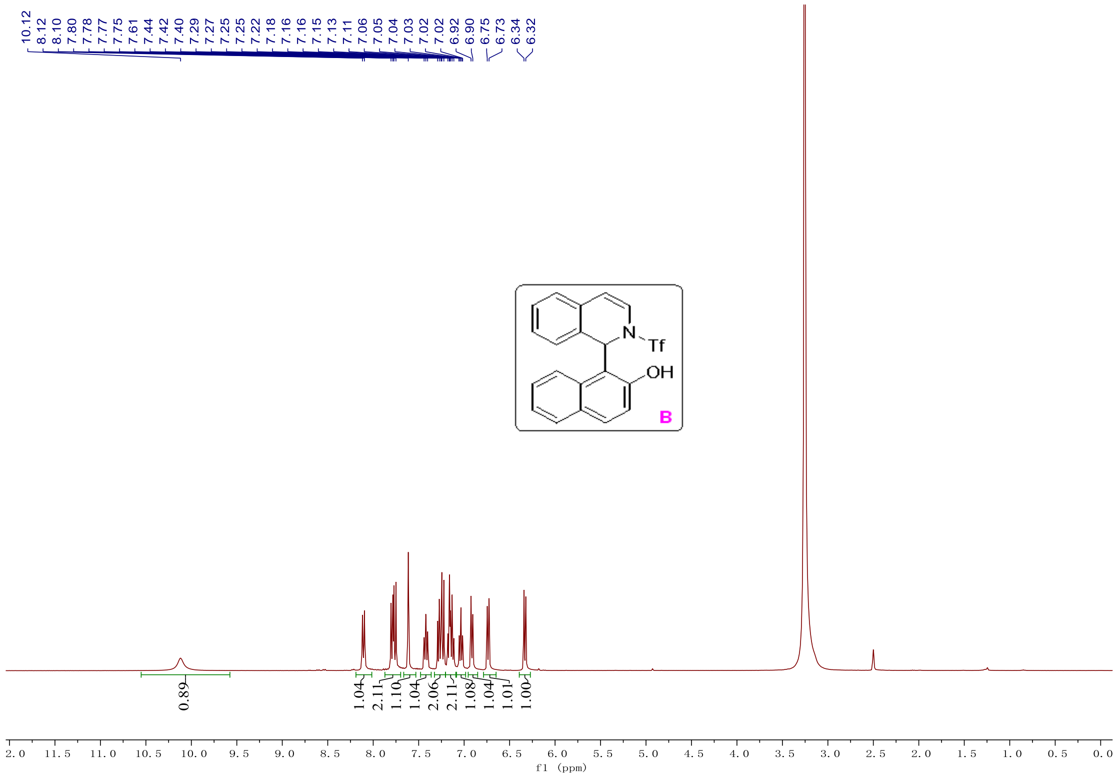

Supplementary Figure 125. ^{1}H NMR spectrum of intermediate **B**

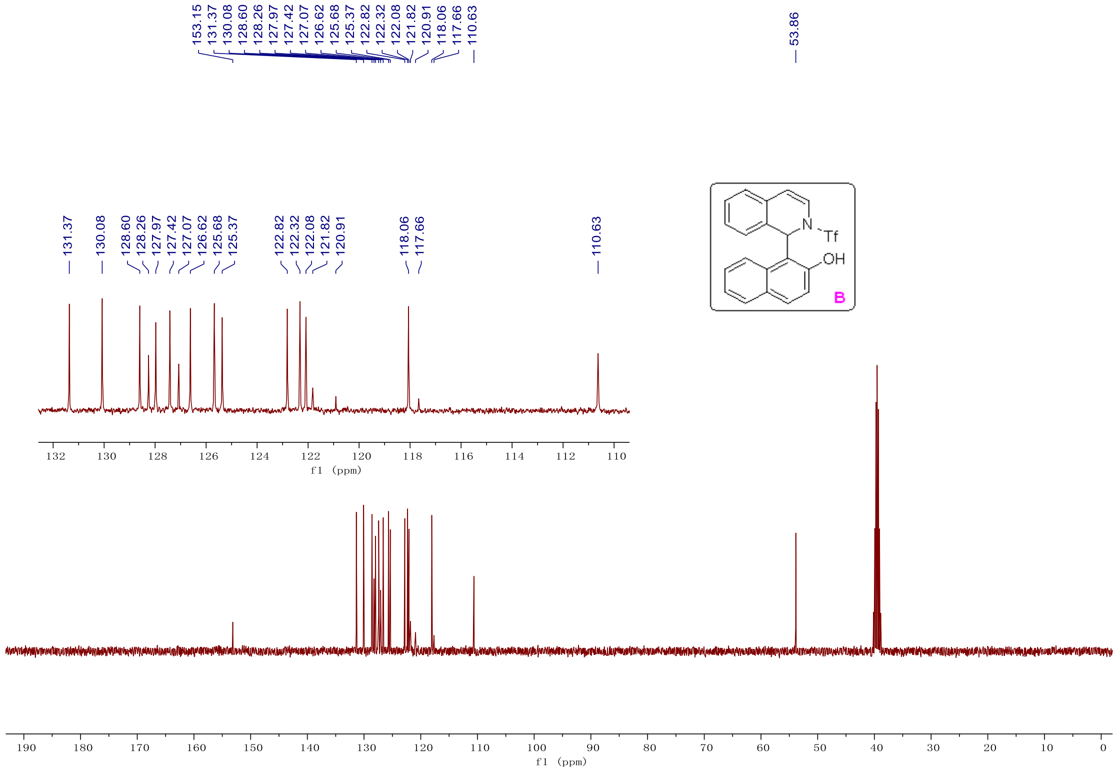

Supplementary Figure 126. ^{13}C NMR spectrum of intermediate **B**

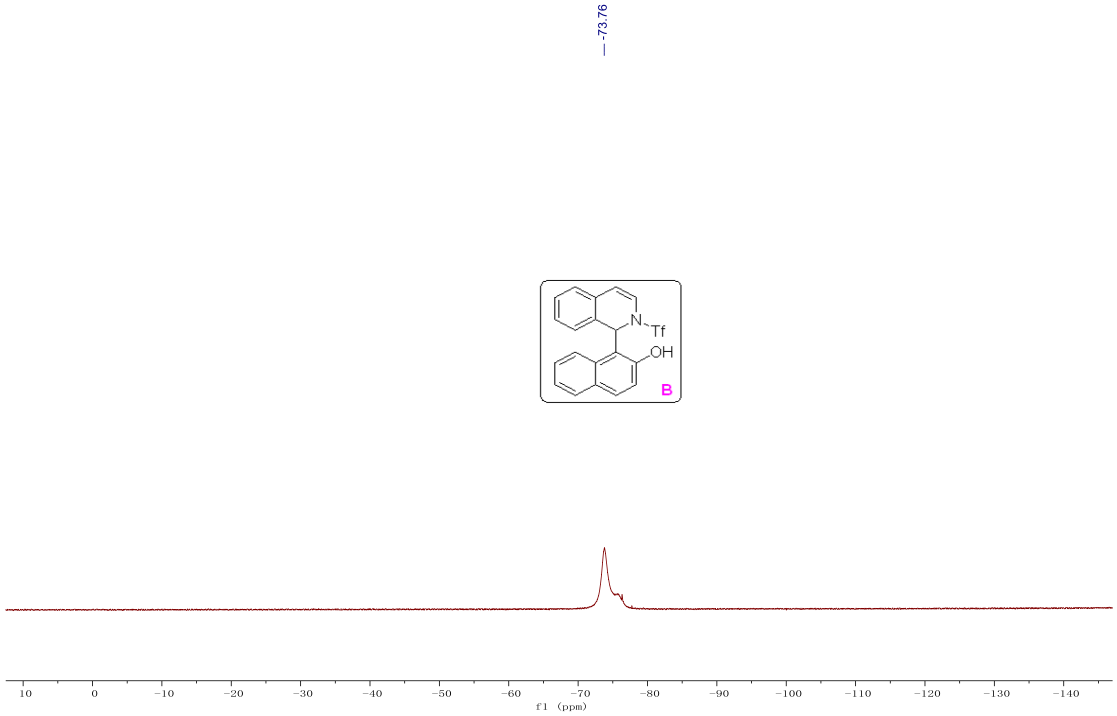

Supplementary Figure 127. ^{19}F NMR spectrum of intermediate **B**

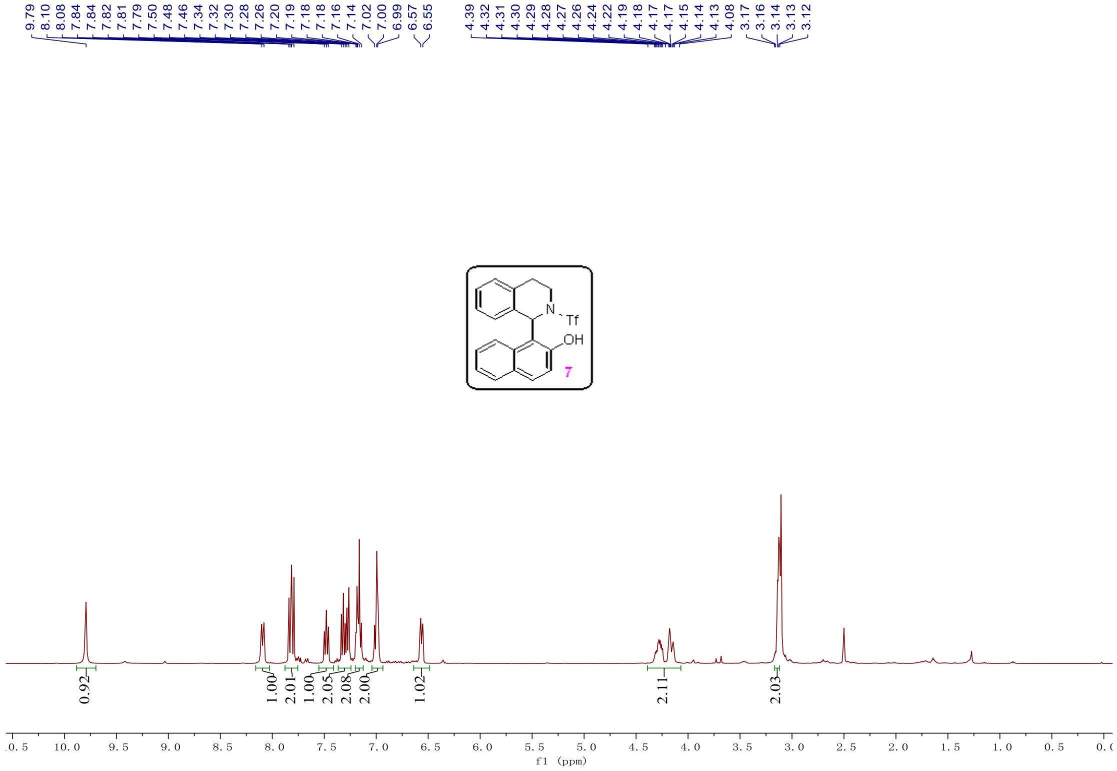

Supplementary Figure 128. ^{1}H NMR spectrum of **7**

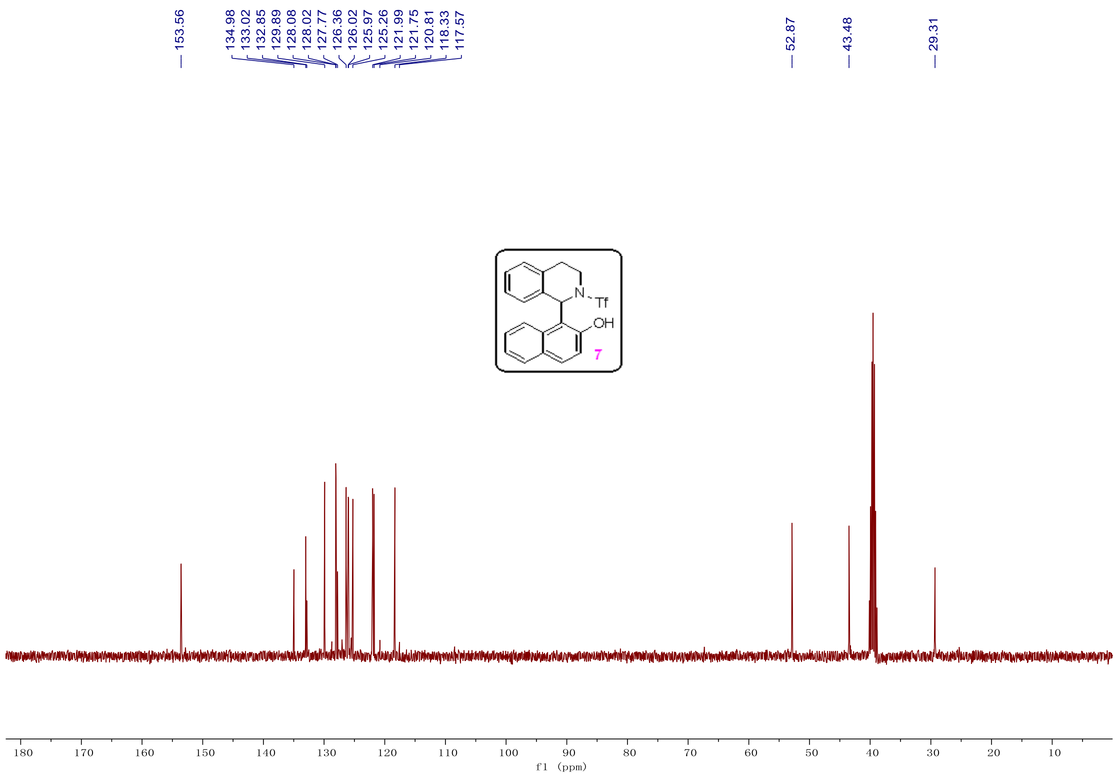

Supplementary Figure 129. ^{13}C NMR spectrum of **7**

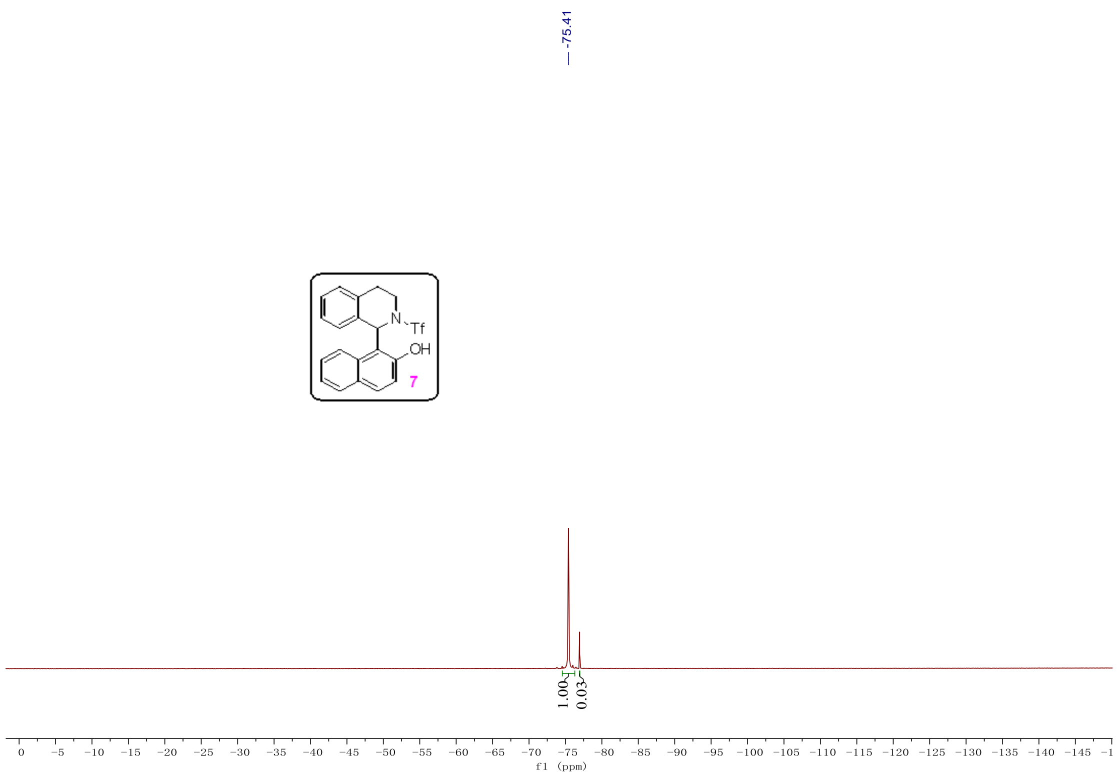

Supplementary Figure 130. ^{19}F NMR spectrum of **7**

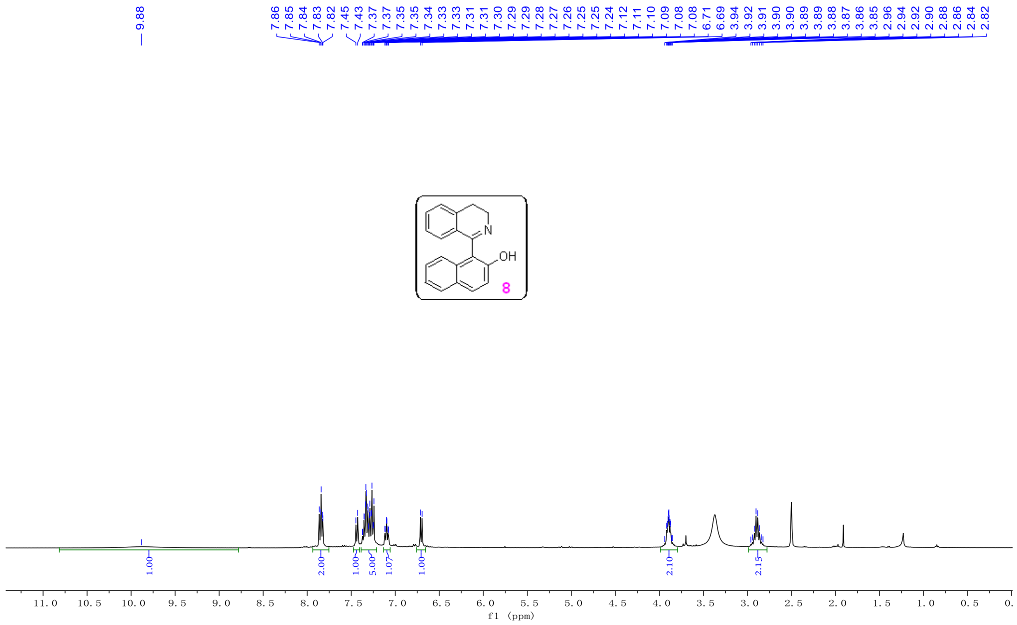

Supplementary Figure 131. ^{1}H NMR spectrum of **8**

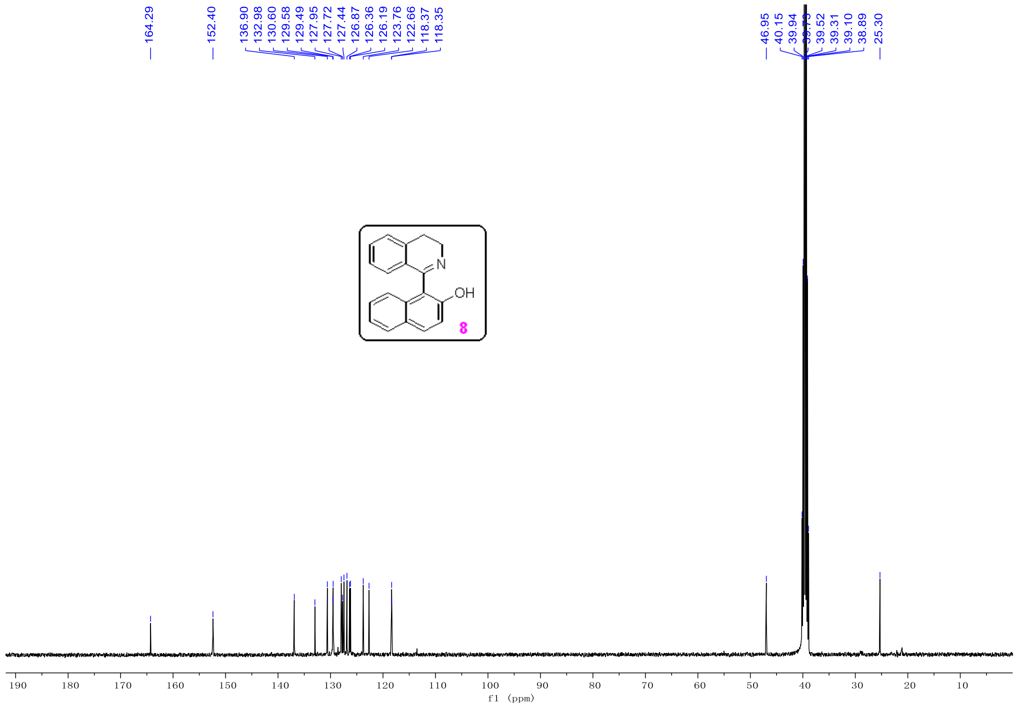

Supplementary Figure 132. ^{13}C NMR spectrum of **8**

11. Supplementary References

1. Meng, S.-S. Tang, W.-B. & Zheng, W.-H. Catalytically Enantioselective Synthesis of Acyclic α-Tertiary Amines through Desymmetrization of 2-Substituted 2-Nitro-1,3-diols. *Org. Lett.* **20**, 518-521 (2018).
2. Kokatla, H. P. Thomson, P. F. Bae, S. Doddi, V. R. & Lakshman, M. K. Reduction of Amine N-oxides by Diboron Reagents. *J. Org. Chem.* **76**, 7842-7848 (2011).
3. Ramírez-López, P. et al. Synthesis of IAN-type N,N-Ligands via Dynamic Kinetic Asymmetric Buchwald–Hartwig Amination. *J. Am. Chem. Soc.* **138**, 12053-12056 (2016).
